# Identification of the Biosynthetic Gene Cluster of Thermoactinoamides and Discovery of New Congeners by Integrated Genome Mining and MS-Based Molecular Networking

**DOI:** 10.3389/fchem.2020.00397

**Published:** 2020-05-21

**Authors:** Gerardo Della Sala, Alfonso Mangoni, Valeria Costantino, Roberta Teta

**Affiliations:** ^1^Laboratory of Pre-clinical and Translational Research, IRCCS-CROB, Referral Cancer Center of Basilicata, Rionero in Vulture, Italy; ^2^Dipartimento di Farmacia, Università degli Studi di Naples Federico II, Naples, Italy

**Keywords:** genome mining, non-ribosomal peptides, molecular networking, metabolomics, antiproliferative activity

## Abstract

The putative non-ribosomal peptide synthetase (NRPS) gene cluster encoding the biosynthesis of the bioactive cyclohexapeptide thermoactinoamide A (**1**) was identified in *Thermoactinomyces vulgaris* DSM 43016. Based on an *in silico* prediction, the biosynthetic operon was shown to contain two trimodular NRPSs, designated as ThdA and ThdB, respectively. Chemical analysis of a bacterial crude extract showed the presence of thermoactinoamide A (**1**), thereby supporting this biosynthetic hypothesis. Notably, integrating genome mining with a LC-HRMS/MS molecular networking-based investigation of the microbial metabolome, we succeeded in the identification of 10 structural variants (**2–11**) of thermoactinoamide A (**1**), five of them being new compounds (thermoactinoamides G-K, **7**–**11**). As only one thermoactinoamide operon was found in *T. vulgaris*, it can be assumed that all thermoactinoamide congeners are assembled by the same multimodular NRPS system. In light of these findings, we suggest that the thermoactinoamide synthetase is able to create chemical diversity, combining the relaxed substrate selectivity of some adenylation domains with the iterative and/or alternative use of specific modules. In the frame of our screening program to discover antitumor natural products, thermoactinoamide A (**1**) was shown to exert a moderate growth-inhibitory effect in BxPC-3 cancer cells in the low micromolar range, while being inactive in PANC-1 and 3AB-OS solid tumor models.

## Introduction

Microbial genome mining has represented a revolutionary approach to search for novel secondary metabolites as drug leads. The ground-breaking idea behind this strategy is deciphering genetic information to depict the chemical structure of a natural product, without having it to hand.

Genome-directed discovery of natural products is pursued through the identification of biosynthetic gene clusters, displaying homology with genes involved in the production of known secondary metabolites. Indeed, although secondary metabolites cover a wide and heterogenous chemical space, the biosynthetic routes for several classes of compounds, such as non-ribosomal peptides and polyketides, are outstandingly conserved across microbial species: at the molecular level, this translates into high sequence similarity of many core biosynthetic enzymes (Ziemert et al., 2016).

Since the discovery of novel secondary metabolites by David Hopwood and coworkers through the whole genome sequencing of *Streptomyces coelicolor* (Bentley et al., 2002), genome mining has made great strides and has become a well-established strategy to access metabolomes of macro- and microorganisms (Della Sala et al., 2013; Wilson and Piel, 2013). Indeed, if natural product discovery is usually driven by bioactivity and/or dereplication techniques, the advent of genome mining has accelerated the chemical workflow, as prediction of secondary metabolite biosynthetic pathways may provide a rationale for targeted isolation of natural products from complex crude extracts (Bachmann et al., 2014). In addition, the screening of bacterial genomes unveiled a huge, unexplored biosynthetic potential, revealing that the number of compounds detected through analytical chemistry approaches is lagging well-behind the putative chemical entities identified by genome mining (Challis, 2008). Taken together, these observations explain why genome mining has contributed to the renaissance of natural product chemistry, giving renewed energy to drug discovery from natural sources.

With the advent of high-quality throughput sequencing methods and the drop of costs, an ever-expanding amount of bacterial genome sequences have become publicly available. The current challenge is handling and mining these sequence data, to detect biosynthetic gene clusters and connect them with the chemical structures of known secondary metabolites or putative novel chemical entities to be isolated from the microbial producer. The emergent need to screen and manipulate a huge amount of sequence data has led to the development of powerful and efficient *in silico* platforms, such as antiSMASH 4.0 (Blin et al., 2017) and PRISM 3 (Skinnider et al., 2017), which are specifically addressed to genome-wide detection of biosynthetic gene clusters and structural prediction of the relevant secondary metabolites.

In the frame of our ongoing research program focused on the isolation of antibacterial and antitumor secondary metabolites from marine organisms and microorganisms (Teta et al., 2012, 2019; Schirmeister et al., 2017), the lipophilic cyclopeptide thermoactinoamide A (**1**) was recently isolated from the Icelandic thermophilic bacterium *Thermoactinomyces vulgaris* and shown to exert antibacterial activity against *Staphylococcus aureus* ATCC 6538 with a MIC value of 35 μM (Teta et al., 2017). Dissection of the chemical structure of thermoactinoamide A suggests that it is assembled by non-ribosomal peptide synthetase (NRPS) machinery, which is based on modular mega-synthetases displaying a standard set of catalytic domains, namely condensation (C), adenylation (A), and thiolation domains (PCP, also known as peptidyl carrier protein). The acknowledged collinearity between the modular architecture of NRPS with their encoded metabolites and the high homology within the key biosynthetic NRPS domains, together with the chance to predict *in silico* substrate selectivity of adenylation domains, has made thermoactinoamide A (**1**) an optimal candidate for a genome mining approach.

Herein, we report new insights into the biosynthesis of the bioactive cyclic peptide thermoactinoamide A (**1**). In light of our observations about the predicted assembly line of **1**, we retrieved from GenBank the whole genome of a *Thermoactinomyces vulgaris* strain (*Thermoactinomyces vulgaris* DSM 43016) and performed a bioinformatic analysis leading to the identification of a putative operon encoding the biosynthetic pathway of thermoactinoamide A. To support this hypothesis, chemical analysis of the organic extract from *Thermoactinomyces vulgaris* DSM 43016 revealed this microbial strain to produce thermoactinoamide A. Notably, combining biosynthetic gene cluster analysis with a mass spectrometry- and molecular networking- based investigation of the microbial metabolome, we succeeded in the identification of 10 structural variants of **1** from *Thermoactinomyces vulgaris* DSM 43016, five of them being new compounds (thermoactinoamides G-K). In addition, *in vitro* evaluation of antiproliferative activity of **1** against three different cancer cell lines is discussed.

## Materials and Methods

### Bioinformatic Analysis

To compare bacterial genomes, average nucleotide identity (ANI) calculations were performed by using the Pairwise ANI tool (Chen et al., 2019), available at the DOE Joint Genome Institute website^1^ Secondary metabolite biosynthetic pathways were identified by genome mining software programs, namely antiSMASH 4.0 (Blin et al., 2017) and PRISM 3 (Skinnider et al., 2017). For the antiSMASH search, the antiSMASH bacterial version was used, setting detection strictness to “relaxed” and selecting the following extra features: KnownClusterBlast, ClusterBlast, SubClusterBlast, ActiveSiteFinder, Cluster Pfam analysis, and Pfam-based GO term annotation. For PRISM analysis, parameters were set as follows: (a) structure limit was set to 50; (b) window was set to 10,000; (c) as optional searches, searches for all families of biosynthetic domains were enabled; and d) for open reading frame prediction, all methods were selected. Substrate selectivity of NRPS adenylation domains was predicted by NRPSpredictor2 (Röttig et al., 2011), while identification and classification of condensation (C) and epimerization (E) domains were accomplished by NaPDoS analyses (Ziemert et al., 2012). The thermoactinoamide (*thd*) gene cluster from *T. vulgaris* DSM 43016 was split on two distinct contigs (contig Ga0070019_105 - accession: REFP01000011; contig Ga0070019_114 - accession: REFP01000005). These two contigs were re-assembled using as a template the contig containing the intact *thd* gene cluster from *Thermoactinomyces* AS95 (contig NODE_4; accession: LSVF01000006). Contigs from *T. vulgaris* DSM 43016 were aligned and re-assembled using the blastn suite (Altschul et al., 1990) and the sequence analysis software SeqMan (DNASTAR v.5.00). Reference-guided assembly led to the integration of contig Ga0070019_114 (accession: REFP01000005) into contig Ga0070019_105 (accession: REFP01000011) (Figure S5). The *de novo* assembled contig sequence is available as Supplementary Material (denovo_assembled_contig.fasta).

### Data-Dependent LC-HRMS/MS Analysis

A 500-mL culture of *Thermoactinomyces vulgaris* DSM 43016 (DSMZ)^2^ was grown for 24 h at 50°C in CYC-medium (Czapek Dox agar 48.0 g/L, yeast extract 2.0 g/L, casamino acids 6.1 g/L, tryptophan 0.02 g/L, sterile Milli-Q H_2_O), and thereafter lyophilized after freezing. Then, the freeze-dried culture was rehydrated with 4 mL of distilled water and sonicated for 5 min. The suspension was extracted with a mixture of MeOH/CHCl_3_ (2:1, 6 mL), paper filtered, and dried to afford 20.6 mg of crude extract, according to a previously reported procedure yielding all low molecular weight metabolites (Costantino et al., 2012). The extract was resuspended in MeOH at a concentration of 10 mg/mL for LC-HRMS/MS (Liquid Chromatography - High Resolution Tandem Mass Spectrometry) analyses. Experiments were performed using a Thermo LTQ Orbitrap XL high-resolution ESI mass spectrometer coupled to a Thermo U3000 HPLC system, which included a solvent reservoir, in-line degasser, binary pump, and refrigerated autosampler. A 5-μm Kinetex C18 column (50 × 2.10 mm), maintained at room temperature, was eluted at 200 μL·min^−1^ with H_2_O (supplemented with 0.1% HCOOH) and CH_3_CN, using a gradient elution. The gradient program was as follows: 30% CH_3_CN 5 min, 30%−99% CH_3_CN over 30 min, 100% CH_3_CN 3 min. Mass spectra were acquired in positive ion detection mode. MS parameters were a spray voltage of 4.8 kV, a capillary temperature of 285°C, a sheath gas rate of 32 units N_2_ (ca. 150 mL/min), and an auxiliary gas rate of 15 units N_2_ (ca. 50 mL/min). Data were collected in the data-dependent acquisition mode, in which the three most intense ions of a full-scan mass spectrum were subjected to high resolution tandem mass spectrometry (HRMS/MS) analysis. The *m/z* range for data dependent acquisition was set between 610 and 800 amu. HRMS/MS scans were obtained for selected ions with CID fragmentation, an isolation width of 2.0, normalized collision energy of 35, Activation Q of 0.250, and an activation time of 30 ms.

### MZmine Processing and Molecular Networking

Processing of LC-HRMS/MS data was performed using a method similar to the feature-based molecular network described by Nothias et al. (2019). Raw files were directly imported into MZmine 2.53 (Pluskal et al., 2010). The mass detection was performed on raw data and exact masses with mass level 1 and centroided masses with mass level 2, by keeping the noise level at 1,000. Chromatograms were built using an ADAP module (Myers et al., 2017) with a minimum height of 1,000, and *m/z* tolerance of 0.05 (or 20 ppm). For the chromatogram deconvolution, the local minimum search algorithm was used with the following settings: chromatographic threshold = 5%, minimum retention time range = 0.50 min, minimum relative height = 30%, minimum absolute height = 10,000, minimum ratio of the peak top/edge = 1.3, and peak duration range = 0.0–6.0 min.

Peak alignment was performed using the Join aligner algorithm (*m/z* tolerance at 0.05 (or 20 ppm), absolute RT tolerance at 0.6 min). [M+Na–H], [M+K–H], [M+Mg−2H], [M+NH_3_], [M-Na+NH_4_], [M+1, ^13^C] adducts were filtered out by setting the maximum relative height at 100%. Peaks without associated MS/MS spectrum were finally filtered out from the peak list. Clustered data were then exported to.mgf file for GNPS, while chromatographic data including retention times, peak areas, and peak heights were exported to a .csv file. The mass spectrometry data were deposited on MassIVE (accession number: MSV000085201).

A molecular network (Wang et al., 2016) was generated on GNPS' online platform,^3^ using the Metabolomics workflow with the following parameters: the parent mass tolerance and MS/MS fragment ion tolerance were set at 0.02 Da and 0.2 Da, respectively, the cosine score at above 0.71, and matched peaks above 6. Spectra were retained only if the nodes appeared in each other's respective top 10 most similar nodes. The spectra in the network were then searched against GNPS spectral libraries using a cosine score above 0.7 and at least 7 matched peaks. Once the molecular network (https://gnps.ucsd.edu/ProteoSAFe/status.jsp?task=a365d81e928f4d0dbc5c76b0dff4515b) was generated, chromatographic data in the .csv file were mapped to the relevant nodes using Cytoscape 3.7.2 (Shannon et al., 2003), which was also used for network visualization and analysis. The fragmentation spectra of Thermoactinoamide A–K were deposited to GNPS library (CCMSLIB0000572020, CCMSLIB00005720245 and CCMSLIB00005720251–CCMSLIB00005720259).

### Isolation and Structural Determination of Thermoactinoamide D and E

The crude extract of *Thermoactinomyces vulgaris* DSM 43016 was subjected to reversed-phase HPLC using a 10 μm Kinetex C18 column (250 × 10 mm) [eluent A: 0.1% HCOOH in H_2_O; eluent B: MeOH; gradient program: 60% B 5 min, 60% → 100% B over 17 min, 100% B 13 min; flow rate 5 mL min^−1^, wavelength 280 nm], thus obtaining pure thermoactinoamide D (**4**), (0.063 mg, t_R_ 15.5 min), that was subjected to NMR experiments and Marfey's analyses.

The fraction eluted at t_R_ 9.5 min containing thermoactinoamide E (**5**) was further purified by reversed-phase HPLC using a 5 μm Kinetex C18 column (250 × 4.6 mm) using the same elution method [eluent A: 0.1% HCOOH in H_2_O; eluent B: MeOH; gradient program: 60% B 5 min, 60% → 100% B over 17 min, 100% B 13 min; flow rate 1 mL min^−1^, wavelength 280 nm]. The amount of thermoactinoamide E was not sufficient to perform NMR experiments.

Both the compounds were hydrolyzed with 6 N HCl/AcOH (1:1) at 120°C for 12 h. The residual HCl fumes were removed under an N_2_ stream. The hydrolysate of **4** was then dissolved in TEA/acetone (2:3, 100 μL) and the solution was treated with 100 μL of 1% 1-fluoro-2,4-dinitrophenyl-5-d-alaninamide (d-FDAA) in CH_3_CN/acetone (1:2) (Marfey, 1984). The vial was heated at 50°C for 2 h. The mixture was dried, and the resulting d-FDAA derivatives of the free amino acids were redissolved in MeOH (200 μL) for subsequent analysis. The hydrolysate of **5** was instead treated with 1-fluoro-2,4-dinitrophenyl-5-l-alaninamide (l-FDAA) in the same conditions as above.

Authentic standards of l-Tyr, l-Val, l-Leu, l-Ile, and d-*allo*-Ile were treated with l-FDAA and d-FDAA as described above and yielded the l-FDAA and d-FDAA standards. Marfey's derivatives of **4** and **5** were analyzed by HPLC-ESI-HRMS, and their retention times were compared with those from the authentic standard derivatives. A 2.6 μm Kinetex PFP column (100 × 4.6 mm) maintained at 25°C was used for compound **4**. The column was eluted at 200 μL min^−1^ with 0.1% HCOOH in H_2_O and MeOH. The gradient program was as follows: 60% MeOH 5 min, 60% → 100% MeOH over 30 min, 100% MeOH 15 min. A 5-μm Kinetex C18 column (50 × 2.10 mm), maintained at room temperature, was instead used for the analysis of Marfey's derivatives of **5**. The column was eluted at 200 μL·min^−1^ with H_2_O (supplemented with 0.1% HCOOH) and MeOH, using a gradient elution. The gradient program was as follows: 5% MeOH 3 min, 5–70% MeOH over 30 min, 70–100% over 1 min, and MeOH 100% 6 min. Mass spectra were acquired in positive ion detection mode, and the data were analyzed using the Xcalibur suite of programs. NMR experiments were performed on Varian Unity Inova spectrometers (Agilent Technology - Cernusco sul Naviglio, Italy) at 700 MHz in CD_3_OD; chemical shifts were referenced to the residual solvent signal (CD_3_OD: δ_*H*_ 3.31, δ_*C*_ 49.00). All ^13^C chemical shift were assigned using the 2D spectra, therefore, mono-dimensional ^13^C NMR spectra were not recorded (see **Table 4**). For an accurate measurement of the coupling constants, the one-dimensional ^1^H NMR spectra were transformed at 64 K points (digital resolution: 0.09 Hz). The HSQC spectra were optimized for ^1^*J*_*CH*_ = 142 Hz and the HMBC experiments for ^2, 3^*J*_*CH*_ = 8.3 Hz.

### Cell Viability Assays

In order to evaluate the growth-inhibitory effects of thermoactinoamide A (**1**), the xCELLigence System Real-Time Cell Analyzer (ACEA Biosciences, San Diego, CA, USA) was used for real-time monitoring of cancer cell proliferation after drug exposure, as previously described (Caso et al., 2019).

Data from biological assay represent the mean (± standard deviation, SD) of three independent experiments. Two-group comparisons were performed using Student's t-test. *p* < 0.05 were considered to be statistically significant. Statistical analysis was performed using the GraphPad Prism Software Version 5 (GraphPad Software Inc., San Diego, CA, USA).

## Results

### Identification of Thermoactinoamide Biosynthetic Gene Cluster

The genome of *Thermoactinomyces vulgaris* strain DSM 43016 was retrieved from GenBank (Accession: NZ_REFP00000000). The published genome from this strain consists of 15 contigs, with an overall size of 2.56 Mb and GC content of 47.9%.

Interestingly, using ANI (Average Nucleotide Identity) calculations, we discovered three further bacterial genomes that definitely affiliate with *Thermoactinomyces vulgaris* strain 43016 (ANI value ≥ 99.56%), although were deposited in the GenBank database as belonging to three different type strains, namely *Thermoactinomyces* AS95 (Accession: LSVF00000000), *Thermoactinomyces* sp. Gus2-1 (Accession: JPZM00000000), and *Thermoactinomyces* sp. CDF (Accession: LFJU00000000) (Table S1). Therefore, these bacterial genomes can be assigned to the same species as the ANI value is higher than the cut-off score (95%) currently accepted for taxonomic inference (Figueras et al., 2014).

The focus of our study was to detect the biosynthetic gene cluster encoding the thermoactinoamide family of compounds. d-Amino acids containing cyclic peptides (such as thermoactinoamides) are either synthesized through the classical NRPS assembly line or produced via post-translational epimerization and cyclization of a ribosomally synthesized precursor peptide (RiPP pathway). Therefore, a preliminary screening was performed to mine the draft genome of *T. vulgaris* DSM 43016 for secondary metabolite biosynthetic pathways, using the publicly available bioinformatic tools, Antismash (Blin et al., 2017) and PRISM (Skinnider et al., 2017).

The genome of *T. vulgaris* DSM 43016 contains 5 putative biosynthetic pathways: a type-III-polyketide synthase gene (contig Ga0070019_104 - accession: REFP01000010); a small NRPS system, consisting of A, PCP, and E proteins, which are encoded by separate genes (contig Ga0070019_101 - accession: REFP01000007); a multi-modular NRPS gene cluster, split between two contigs (contig Ga0070019_105 - accession: REFP01000011; contig Ga0070019_114 - accession: REFP01000005); a lassopeptide; and a siderophore gene clusters (contig Ga0070019_101; accession: REFP01000007). As expected, genome mining of *Thermoactinomyces* AS95, Gus2-1, and CDF revealed the presence of the same secondary metabolite biosynthetic gene clusters as in *T. vulgaris* DSM 43016.

Owing to the collinearity between the NRPS domain architecture and the thermoactinoamide chemical structures, the biosynthesis of thermoactinoamides was predicted to be directed by the multi-modular NRPS pathway, which was designated as “*thd*.” Prior to bioinformatic analysis, we manually assembled the two distinct contigs the NRPS gene cluster was split on, using the contig from *Thermoactinomyces* AS95 (contig NODE_4; accession: LSVF01000006) containing the intact *thd* gene cluster as a template. Putative genes identified in the *de novo* assembled contig from *T. vulgaris* DSM 43016 are summarized in Table 1.

**Table 1:** Putative genes identified on the *de novo* assembled contig containing the thermoactinoamide gene cluster (highlighted in green and bolded) from *T. vulgaris* DSM 43016.

**ORF**	**Protein ID**	**Position [nt]**	**No. of aa**	**Putative function**	**Closest homolog (accession#) organism**	**expect value**	**identity/positives [% aa]**
ORF1	RMB00104	24,613–26,286	557	ribonuclease J	WP_037996450, *Thermoactinomyces*	0.0	99/100
ORF2	RMB00105	26,442–26,999	185	hypothetical protein	WP_037996506, *Thermoactinomyces*	2e−134	100/100
ORF3	RMB00106	27,109–28,356	415	peptidoglycan amidohydrolase	WP_052186787, *Thermoactinomyces*	0.0	100/100
ORF4	RMB00107	28,668–28,895	75	hypothetical protein	WP_037996451, *Thermoactinomyces*	3e−45	100/100
ORF5	RMB00108	28,979–29,302	107	multidrug efflux SMR transporter	WP_037996453, *Thermoactinomyces*	9e−68	100/100
**ThdA**	RMB00109	29,719–41,271	3,850	NRPS (C*-A-PCP-C-A-PCP-E-C-A-PCP-E)	WP_049720208, *Thermoactinomyces* sp. CDF	0.0	99/99
**ThdB**	KYQ85937	41,347–51,942	3531	**NRPS (C-A-PCP-C-A-PCP-C-A-PCP-E)**	WP_138614946, *Thermoactinomyces vulgaris*	0.0	99/99
ORF8	RMB00111	51,988–52,482	164	hypothetical protein	WP_049720207, *Thermoactinomyces*	2e−118	100/100
ORF9	RMB00112	52,923–53,867	314	dihydroxyacetone kinase subunit DhaK	WP_037996514, *Thermoactinomyces*	0.0	100/100
ORF10	RMB00113	53,869–54,501	210	dihydroxyacetone kinase subunit L	WP_037996459, *Thermoactinomyces*	2e−153	100/100
ORF11	RMB00114	54,498–54,884	128	dihydroxyacetone kinase subunit DhaM	WP_037996461, *Thermoactinomyces*	9e−86	100/100
ORF12	RMB00115	54,971–55,693	240	ATP-dependent protease ClpP protease subunit	WP_037996464, *Thermoactinomyces*	2e−173	99/100
ORF13	RMB00116	55,690–55,911	73	YlzJ-like protein	WP_037996465, *Thermoactinomyces*	3e−46	100/100

C*, truncated condensation domain, probably inactive.

**Figure 1:**
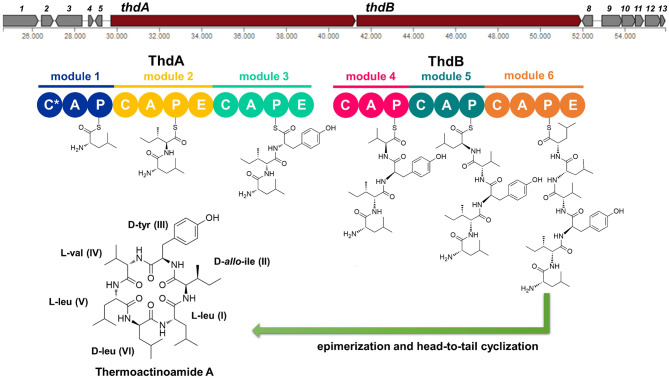
Putative biosynthesis of thermoactinoamide A (abbreviations: C, condensation domain; A, adenylation; P, peptidyl-carrier protein; E, epimerase; C*, truncated condensation domain, probably inactive).

The putative *thd* gene cluster is 22,223 bp long and encodes two NRPSs, ThdA and ThdB, each comprising of three modules. As shown in Figure 1, biosynthesis of thermoactinoamide A starts with the loading module of ThdA, which activates an l-Leu residue by adenylation. Selectivity for leucine was predicted based on the specificity-conferring code of A domains proposed by Stachelhaus et al. (1999); in addition, an NRPSpredictor2 search (currently incorporated in Antismash) also predicted leucine specificity based both on the sequence of the A domain and on structural features of the active site (Röttig et al., 2011) (Table 2). After activation and its ligation to the first PCP of ThdA, the l-Leu residue is forwarded to the first extending module of ThdA, displaying a ^L^C_L_-A-PCP-E domain organization and catalyzing condensation between the l-Leu residue and one l-Ile. The A domain showed an amino acid-specific structural motif selective for isoleucine (Table 2). NaPDoS analyses (Ziemert et al., 2012) as well as detection of the down-seq signatures reported by Caradec et al. (2014), supported identification within this extending module of one ^L^C_L_ (condensation of two l amino acids) domain and one E (epimerization) domain (Table S2; Figures S1, S2). The presence of an epimerization domain suggests conversion of the l-Ile residue to the corresponding d-*allo*-Ile, which is in agreement with the absolute configuration of this amino acid in thermoactinoamide A (Teta et al., 2017).

**Table 2:** Signature motifs of adenylation domains from the Thd synthetase.

	**Antismash prediction**	**Binding pocket signatures (residue position^a^)**								
		**235**	**236**	**239**	**278**	**299**	**301**	**322**	**330**	**331**
ThdA_A1	Leu	D	A	W	F	L	G	N	V	V
ThdA_A2	Ile/Val	D	G	F	F	L	G	V	V	F
ThdA_A3	Tyr	D	A	A	T	I	A	A	V	C
ThdB_A4	Ile/Val	D	G	F	F	V	G	G	V	F
ThdB_A5	Leu	D	A	W	F	L	G	N	V	V
ThdB_A6	Leu	D	A	W	F	L	G	N	V	V

a *as reported by Teta et al. (2015)*.

The last NRPS elongation module of ThdA extends the dipeptidyl intermediate l-Leu-d-*allo*-Ile with a tyrosine residue, because the ThdA_A3 domain was found to be specific for tyrosine according to NRPS predictor2 (Stachelhaus code match: 90%) (Table 2). As expected, this module starts with a condensation domain featuring signatures of ^D^C_L_ domains, to form a peptide bond between d-*allo*-Ile and l-Tyr. Then, a second epimerase within the last module of ThdA is consistent with occurrence of an l-tyrosine in thermoactinoamide A (Table S2; Figures S1, S2).

The growing chain is then transferred to the NRPS enzyme ThdB, which includes three modules each adding one amino acid, namely valine, leucine, and leucine. The first adenylation domain (ThdB_A4) of ThdB was predicted to activate either valine or isoleucine, thus accounting for a relaxed substrate selectivity (Table 2). Residues lining the binding pocket of ThdB_A4 are essentially hydrophobic, in accordance with the lipophilicity of the substrate sidechain of valine or isoleucine residues. Considering that (a) the specificity conferring code partially matches both with Ile and Val activating enzymes and (b) thermoactinoamide variants include either Val or Ile at that position, it can be argued that the A-domain binding pocket allows accommodation of both amino acids.

The first condensation domain of ThdB was classified as a ^D^C_L_ domain in an NaPDoS search and is involved in the amide bond formation between d-Tyr and l-Val (or Ile) (Table S2). Then, the last two modules of ThdB extend the peptide growing chain by the sequential addition of two l-leucine residues. Indeed, ThdB_A5 and ThdB_A6 share a 100% sequence identity and were predicted as specific to leucine (Table 2). The relevant condensation domains (ThdB_C5 and ThdB_C6) within the last two modules feature conserved ^L^C_L_ motifs as expected. Isomerization of the terminal l-leucine to d-leucine is in charge of ThdB_E3 epimerase (Table S2; Figures S1, S2).

Unexpectedly, the *thd* gene cluster lacks a typical thioesterase/cyclase, which is usually required for hydrolytic release and head-to-tail cyclization of the linear peptide (Kopp and Marahiel, 2007). Moreover, neither a trans-acting cyclase, such as SurE in surugamide biosynthesis (Kuranaga et al., 2018), nor a terminal condensation domain (C_T_), such as those responsible for cyclization in fungal NRPSs (Gao et al., 2012), could be detected within the thermoactinoamide NRPS. Despite the lack of usual chain termination domain, the observation that cyclic peptides are produced by *T. vulgaris* DSM 43016 implies the existence of unique off-loading and macrolactamization mechanisms. Intriguingly, the NRPS involved in the biosynthesis of acetylaszonalenin, namely AnaPS, lacks a canonical thioesterase/cyclase and terminates with an epimerization (E) domain, which has been proposed to promote a specific conformation of the linear intermediate to undergo cyclization (Gao et al., 2012). Similarly, ThdB_E epimerase could position the N-terminal Leu amine group next to the C-terminal Leu thioester carbonyl, thereby allowing the head-to-tail macrocyclization to take place. Moreover, considering that epimerases and condensation domains share both sequence and structure features (Keating et al., 2002), it could be hypothesized that ThdB E catalyzes the last condensation step, thus resulting in the hydrolytic cleavage and macrolactamization of the final thermoactinoamide.

### Structure and Biosynthesis of Thermoactinoamide A Congeners

The actual production of thermoactinoamide A and its congeners by *T. vulgaris* DSM 43016 was confirmed by chemical analysis. The strain was extracted with a MeOH/CHCl_3_ mixture and the crude extract was subjected to liquid chromatography high-resolution tandem mass spectrometry (LC-HRMS/MS) on an LTQ Orbitrap instrument. LC-MS data were processed using an implementation of feature-based molecular networking (Nothias et al., 2019). The raw LC-MS data were pre-processed using the MZmine program (Pluskal et al., 2010) and the .mgf MS2 data file generated by MZmine was submitted to the online platform at the Global Natural Products Social Molecular Networking website^3^. Mapping of chromatographic information exported from MZmine and visualization of the network were performed using the Cytoscape program (Shannon et al., 2003).

**Figure 2:**
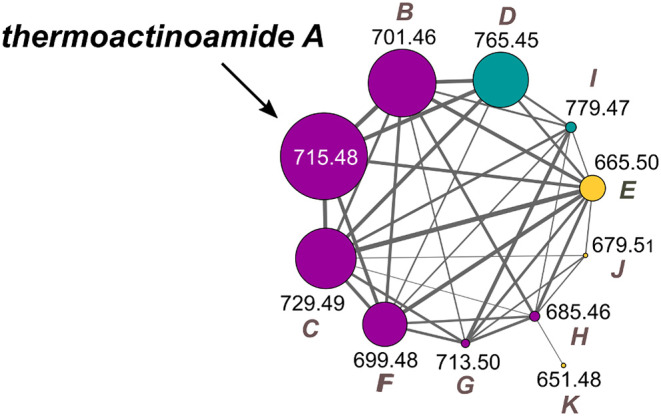
Molecular cluster of thermoactinoamides. Nodes for thermoactinoamides containing one aromatic residue are labeled in purple, while thermoactinoamides containing two and no aromatic residue are marked in blue and orange, respectively. Node size is relative to the areas of the relevant peaks in the extracted-ion chromatograms from the LC-MS run and edge thickness is relative to the cosine score similarity.

The thermoactinoamide cluster (Figure 2) contains eleven nodes, indicating the presence of ten compounds closely related to thermoactinoamide A (**1**, *m/z* 715.48), and is represented so that the area of the nodes is proportional to the amounts of the relevant compounds. This makes it immediately clear that all the congeners of thermoactinoamide A are far less abundant than the parent compound. Because no other related NRPS clusters were present in the genome of *T. vulgaris* DSM 43016, all these minor thermoactinoamide variants (Table 3) must be produced by the same multimodular NRPS gene cluster that synthesizes thermoactinoamide A.

**Table 3:** Thermoactinoamide variants from *T. vulgaris* DSM 43016.

**name**	***m/z* [M+H]^**+**^**	**molecular formula**	**aa-1**	**aa-2**	**aa-3**	**aa-4**	**aa-5**	**aa-6**
thermoactinoamide A (**1**)	715.4751	C_38_H_62_N_6_O_7_	l-Leu	d-*allo*-Ile	d-Tyr	l-Val	l-Leu	d-Leu
thermoactinoamide B (**2**)	701.4596	C_37_H_60_N_6_O_7_	l-Leu	d-Val	d-Tyr	l-Val	l-Leu	d-Leu
thermoactinoamide C (**3**)	729.4909	C_39_H_64_N_6_O_7_	l-Leu	d-*allo*-Ile	d-Tyr	l-Ile	l-Leu	d-Leu
thermoactinoamide D (**4**)	765.4545	C_41_H_60_N_6_O_8_	l-Tyr	d-*allo*-Ile	d-Tyr	l-Val	l-Leu	d-Leu
thermoactinoamide E (**5**)	665.4960	C_35_H_64_N_6_O_6_	l-Ile	l-Leu	d-*allo*-Ile	l-Val	l-Leu	d-Leu
thermoactinoamide F (**6**)	699.4804	C_38_H_62_N_6_O_6_	l-Leu	d-*allo*-Ile	d-phe	l-Val	l-Leu	d-Leu
thermoactinoamide G (**7**)	713.4958	C_39_H_64_N_6_O_6_	l-Leu	d-*allo*-Ile	d-phe	l-Ile	l-Leu	d-Leu
thermoactinoamide H (**8**)	685.4646	C_37_H_60_N_6_O_6_	l-Leu	d-Val	d-phe	l-Val	l-Leu	d-Leu
thermoactinoamide I (**9**)	779.4705	C_42_H_62_N_6_O_8_	l-Tyr	d-*allo*-Ile	d-Tyr	l-Ile	l-Leu	d-Leu
thermoactinoamide J (**10**)	679.5117	C_36_H_66_N_6_O_6_	l-Ile	l-Leu	d-*allo*-Ile	l-Ile	l-Leu	d-Leu
thermoactinoamide K (**11**)	651.4801	C_34_H_62_N_6_O_6_	l-Val	l-Leu	d-*allo*-Ile	l-Val	l-Leu	d-Leu

*Color code relates to the presence of one (purple), two (blue), or no one (orange) aromatic aminoacid residue in the structure*.

*All the thermoactinoamides MS/MS spectra have been annotated on GNPS*.

**Figure 3:**
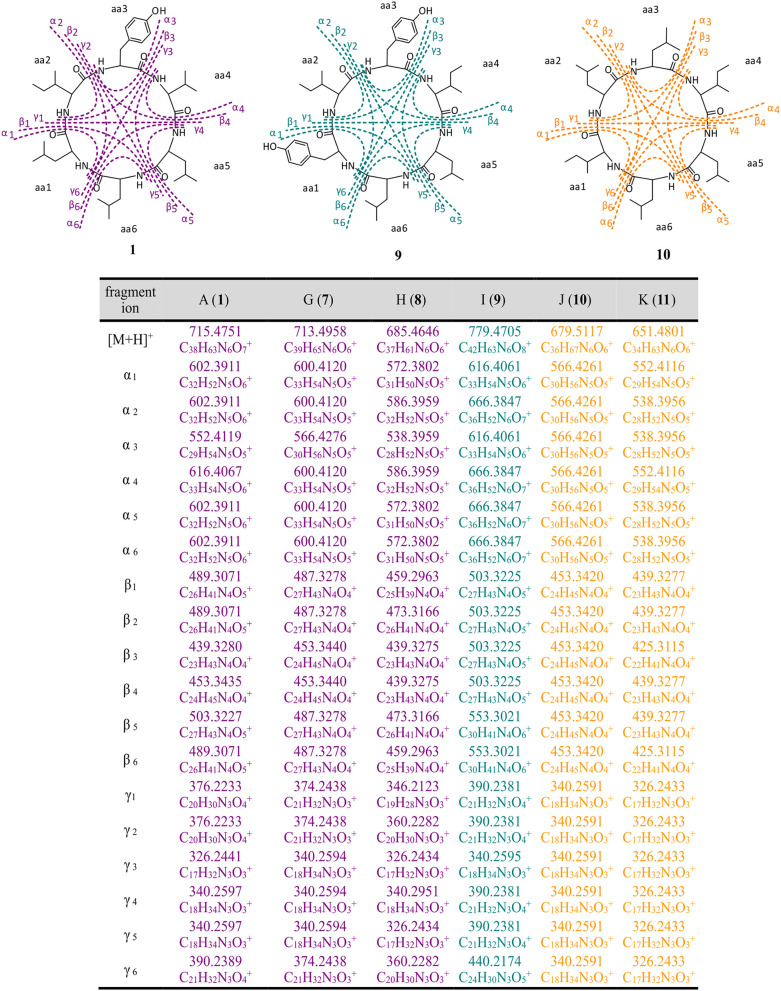
High-resolution MS/MS fragmentations of new thermoactinoamide variants. Thermoactinoamides A (**1**), G (**7**), H (**8**) containing one aromatic residue are labeled in purple, while thermoactinoamides containing two (**9**) and no aromatic residue (**10** and **11**) are marked in blue and orange, respectively. Fragmentation scheme is depicted for thermoactinoamides A (**1**), I (**9**), and J (**10**), as representative of each class of compounds. Fragment α, β, and γ are the losses of one, two, and three amino acids from the parent ions.

The structure of thermoactinoamide A (**1**) was confirmed by comparison of its HPLC retention time, MS/MS spectrum, and ^1^H NMR spectrum with those reported (Teta et al., 2017). The high-resolution MS/MS fragmentation pattern of **1** was taken as a model to study in detail all the thermoactinoamide congeners (Figure 3). As observed for most cyclic peptides (Ngoka and Gross, 1999), the typical fragmentation mode of thermoactinoamides consists in the cleavage of two amide bonds with the loss of one, two, or three amino acid residues from the parent ion (called ions α, β, and γ, respectively, in the following discussion). These fragment ions provide information on the amino acid sequence in the peptides and in the previous study (Teta et al., 2017) identified correctly the amino acid sequence of compound **1**, except for discrimination of isomeric Leu and Ile and all the stereochemical aspects. As a side remark, elucidation of the latter structural aspects required an extensive spectroscopic and chemical analysis, while a previous knowledge of the biosynthetic cluster of thermoactinoamide could have provided the same information immediately.

Monoisotopic masses, retention times, and fragmentation patterns of peptides **2–6** identified them as the thermoactinoamides B-F reported in Teta et al. (2017). In that study, compounds **2**–**6** were not isolated and their structures were putatively assigned based on their MS/MS fragmentations and of the assumption that they differed from thermoactinoamides A (**1**) only in a single amino acid. Therefore, structures **2**–**6** were re-examined in the light of the newly acquired knowledge of their biosynthetic cluster.

Besides fitting MS/MS fragmentation data, structures of thermoactinoamide B (**2**), C (**3**), and F (**6**) fit well with the biosynthetic cluster. They show, respectively, an Ile → Val substitution at aa-2, a Val → Ile substitution at aa-4, and a Tyr → Phe substitution at aa-3. These substitutions are among the most common for minor congeners of NRPS products. Putative isoleucine-activating adenylation domains of NRPSs have been shown to also select valine to a similar extent, and conversely valine-activating enzymes are able to recruit isoleucine as well (Challis et al., 2000). Furthermore, based on the aromatic substrate selectivity of ThdA_A3, it is not surprising that a Phe residue may replace a Tyr residue in thermoactinoamide F (**6**). In contrast, the reported putative structures of thermoactinoamide D (**4**) and E (**5**) did not obviously match up with the modular architecture of the *thd* gene cluster. Therefore, these two congeners were isolated to determine their structure by spectroscopic and chemical means.

**Figure 4:**
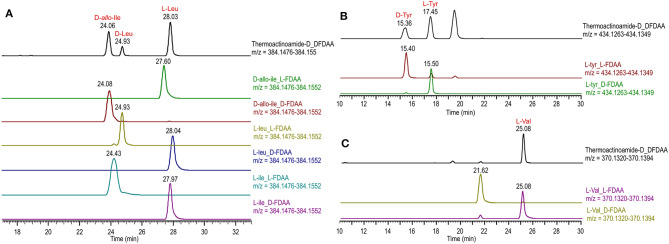
Advanced Marfey's analysis of compound **4** using a pentafluorophenyl (PFP) bonded-phase column. **(A)** Extracted-ion chromatograms at *m/z* 384.1514 of the d-FDAA derivatives from the hydrolysis of **4** and of d- and l-FDAA derivatives l-Leu, l-Ile, and d-*allo*-Ile. **(B)** Extracted-ion chromatograms at *m/z* 434.1306 of the d-FDAA derivatives from the hydrolysis of **4** and of d- and l-FDAA derivatives l-Tyr. **(C)** Extracted-ion chromatograms at *m/z* 370.1357 of the d-FDAA derivatives from the hydrolysis of **4** and of d- and l-FDAA derivatives l-Val.

Thermoactinoamide D (**4**) displays two Tyr residues, with the additional Tyr located on aa-1, a position that in the other variants is held by a strikingly conserved l-Leu. This would imply an extended substrate specificity to Tyr of the Leu-specific ThdA_A1 loading module. Even though the location of the additional Tyr residue could be unequivocally deduced by MS/MS data, this unexpected substitution prompted us to perform a full 2D NMR characterization of thermoactinoamide D (**4**), whose results are summarized in Table 4. Consistently with MS data, the HMBC correlations of the l-Tyr CO carbon atoms with the d-*allo*-Ile H-2 proton and of the d-*allo*-Ile CO carbon atoms with the d-Tyr H-2 proton indicated that the d-*allo*-Ile residue lies between the two Tyr residues. Marfey analysis (Figure 4) revealed the configuration of the two Tyr residues of **4** to be l and d, respectively, and confirmed the identity and the configuration of the other amino acids. Finally, the l-Tyr residue was assigned to position 1 according to gene-based stereochemistry prediction. Therefore, the hypothesized structure of thermoactinoamide D (**4**) was fully confirmed by NMR and Marfey analysis, and the relaxed substrate selectivity of ThdA_A1 therefore confirmed. Indeed, multi-specificity of adenylation domains is not unusual and has been extensively documented in the literature (Meyer et al., 2016). It is worthy to note that the N-terminal truncated ^*^C domain could influence substrate selectivity of the downstream A domain within the starter module of ThdA. It has been demonstrated that C-terminal subdomains at the interface with the A domains exert a gatekeeping function and modulate substrate selection by the A domain (Meyer et al., 2016).

**Table 4:** NMR data of thermoactinoamide D (**4**) (700 MHz, CD_3_OD).

**amino acid**	**Pos**.	**δ_C_, mult**		**δ_H_, mult (*J* in Hz)**	**HMBC^*a*^**
l-Tyr	1	174.0 (C)		–	
	2	56.1 (CH)		4.56 (ovl)^*b*^	1,3, d-Leu-1
	3	35.5 (CH_2_)	a	2.95 (dd, 13.7, 6.3)	1,2,4,5/9
			b	2.85 (dd, 13.7, 6.3)	1,2,4,5/9
	4	126.5 (C)		–	
	5/9	129.9 (CH)		7.06 (d, 8.2)	5/9,6/8,7
	6/8	115.0 (CH)		6.72 (d, 8.3)	4,6/8,7
	7	156.3 (C)		–	
d-*allo*-Ile	1	172.1 (C)		–	
	2	57.0 (CH)		4.11 (d, 3.9)	1,3,4,5, l-Tyr-1
	3	35.8 (CH)		1.94 (m)	
	4	12.9 (CH_3_)		0.63 (d, 7.0)	2,3,5,
	5	25.5 (CH_2_)	a,b	0.71 (m)	3,4
	6	10.8 (CH_3_)		0.71 (m)	3,5
d-Tyr	1	173.2 (C)		–	
	2	55.0 (CH)		4.85 (ovl)	1,3, d-*allo*-Ile-1
	3	38.0 (CH_2_)	a,b	2.95 (m)	1,2,4,5/9
	4	127.3 (C)			
	5/9	130.0 (CH)		7.07 (d, 8.2)	5/9,6/8,7
	6/8	115.0 (CH)		6.71 (d, 8.3)	4,6/8,7
	7	156.1 (C)			
l-Val	1	172.4 (C)		–	
	2	61.5 (CH)		3.66 (d, 5.4)	1, d-Tyr-1
	3	29.3 (CH)		1.95 (m)	1,5,6
	4	24.2 (CH)		1.47 (ovl)	
	5	18.0 (CH_3_)		0.82 (d, 6.9)	2,3,6
	6	17.1 (CH_3_)		0.88 (d, 6.9)	2,3,5
l-Leu	1	172.6 (C)		–	
	2	51.3 (CH)		4.45 (dd, 10.9, 4.3)	1,3,4, l-Val-1
	3	38.7 (CH_2_)	a,b	1.76 (m)	1,2,4
	4	24.4 (CH)		1.60 (m)	
	5	21.1 (CH_3_)		0.97 (d, 6.4)	3,4,6
	6	19.6 (CH_3_)		0.90 (d, 6.4)	3,4
d-Leu	1	172.3 (C)			
	2	50.5 (CH)		4.58	1, l-Leu-1
	3	39.9 (CH_2_)	a	1.72 (m)	1,2
			b	1.64 (m)	2
	4	22.1 (CH)		0.97 (ovl)	3
	5	13.2 (CH_3_)		0.92 (d, 6.4)	4
	6	13.2 (CH_3_)		0.91 (d, 6.4)	4

a *Selected HMBC correlations from proton stated to the indicated carbon*.

b *Overlapped*.

**Figure 5:**
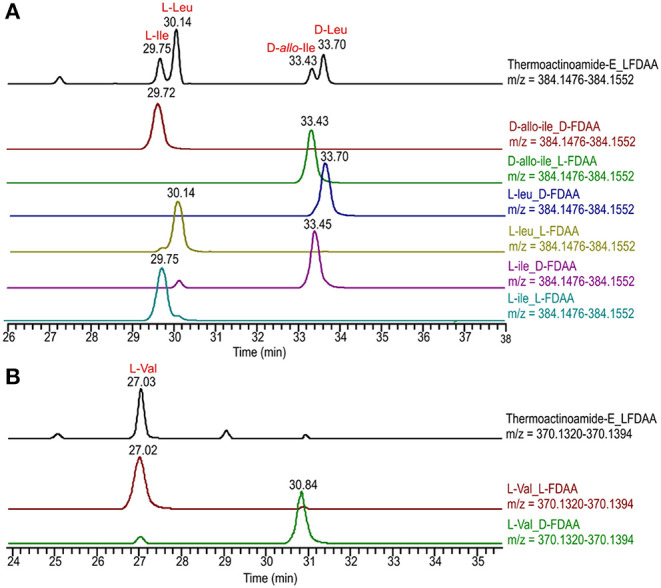
Advanced Marfey's analysis of compound **5** using a C18 reversed-phase column. **(A)** Extracted-ion chromatograms at *m/z* 384.1514 of the l-FDAA derivatives from the hydrolysis of **5** and of d- and l-FDAA derivatives l-Leu, l-Ile, and d-*allo*-Ile. **(B)** Extracted-ion chromatograms at *m/z* 370.1357 of the l-FDAA derivatives from the hydrolysis of **5** and of d- and l-FDAA derivatives l-Val.

The isolated amounts of thermoactinoamide E (**5**) were too small for 2D NMR-based structure elucidation, but advanced (MS-aided) Marfey analysis could still be performed and allowed for distinction between Leu, Ile, and *allo*-Ile residues and between their respective d and l enantiomers. This way, thermoactinoamide E (**5**) was shown to contain 1 × l-Ile, 2 × l-Leu, 1 × d-*allo*-Ile, 1 × d-Leu, and 1 × l-Val, an amino acid composition that did not agree with the reported putative structure (Figure 5). More interestingly, the determined amino acid composition is different from that of all the other congeners (**1**–**4** and **6**) discussed so far, in that only two d amino acids are present in the sequence. Thermoactinoamide E (**5**) contains only aliphatic amino acids and could derive from cyclo-dimerization of the tripeptidyl unit l-Ile/Val-l-Leu-d-Leu/*allo*-Ile. Therefore, it can be argued that it is synthesized by the sole ThdB module acting iteratively. ThdB possesses only one epimerization domain and if it functions twice to produce a cyclic hexapeptide, the resulting peptide is expected to contain two d amino acid residues, as thermoactinoamide E (**5**) does. Based on these considerations, and taking into account the substrate selectivity of the A modules of ThdB discussed in the previous sections, the structure of thermoactinoamide E (**5**) was revised to cyclo (l-Ile-l-Leu-d-*allo*-Ile-l-Val-l-Leu-d-Leu).

In addition to the congeners **2**–**6** discussed so far, the thermoactinoamide node cluster contained additional nodes of five new compounds, which were named thermoactinoamides G-K (**7**–**11**). They were present in even smaller amounts than **2**–**6**, and none of them could be isolated. Therefore, their structures, including absolute configurations, were assigned combining spectral information provided by MS/MS fragmentation with biosynthetic information provided by the bioinformatic analysis of the gene cluster as well as the structures of the congeners **2**–**6** discussed above.

The molecular formula of thermoactinoamide G (**7**) (C_39_H_65_N_6_${\text{O}}_{6}^{+}$, *m*/*z* 713.4958) contained one additional CH_2_ and lacked one O compared to thermoactinoamide A (**1**), suggesting concurrent Tyr → Phe and Val → Ile/Leu substitutions. This was confirmed by the HR-MS/MS spectrum of **7**, which contained a fragment ion at *m/z* 566.4276 (ion α, C_30_H_56_N_5_${\text{O}}_{5}^{+}$) composed of five Ile/Leu residues and originating from the loss of a Phe residue.

Thermoactinoamide H (**8**) (C_37_H_61_N_6_${\text{O}}_{6}^{+}$, *m*/*z* 685.4646) lacked one CH_2_ and one O compared to compound **1**, suggesting Tyr → Phe and Ile/Leu → Val substitutions. The tripeptide fragment ion at *m/z* 346.2123 (ion γ_1_) showed that the molecule contains three Ile/Leu residues in a row, and a complementary Val_2_Phe fragment ion at *m/z* 340.2951 (ion γ_1_) was also present in the MS/MS spectrum. The fragment ions β_1_/β_1_, both at *m/z* 439.3275, and the absence of a fragment ion from loss of two Val residues showed that Phe is located between the two valine residues.

The fragmentation pattern of thermoactinoamide I (**9**) (C_42_H_63_N_6_${\text{O}}_{8}^{+}$, *m/z* 779.4705) closely resembled that of thermoactinoamide D (**4**), differing only in a CH_2_, therefore in the substitution of the Val residue (aa-4) with an Ile/Leu residue. As for thermoactinoamide D, the fragment ion at *m/z* 440.2174 is (Leu/Ile)_1_(Tyr)_2_ and specifically is a Tyr-Leu/Ile-Tyr fragment, because there is no Tyr-Tyr fragment, nor a fragment due to the loss of Tyr-Tyr.

Finally, thermoactinoamide J (**10**) (*m/z* 679.5117, C_36_H_67_N_6_${\text{O}}_{6}^{+}$) and K (**11**) (*m/z* 651.4801, C_36_H_67_N_6_${\text{O}}_{6}^{+}$) belong, like thermoactinoamide E (**5**), to the series of thermoactinoamides entirely composed of aliphatic amino acids and putatively synthesized by ThdB alone. Thermoactinoamide J (**10**) is composed of six isobaric amino acids and MS/MS fragmentation cannot provide any information on its sequence. However, on the basis of the results of this study, a putative but reliable structure, including absolute configuration, can be proposed for **10**, which involves a ValIle substitution at aa-4 compared to **5**. Likewise, thermoactinoamide K (**11**) is a lower homolog of thermoactinoamide E (**5**) and is expected to differ from the latter only for the IleVal substitution at aa-1.

### Evaluation of Antiproliferative Activity of Thermoactinoamide A (1)

**Figure 6:**
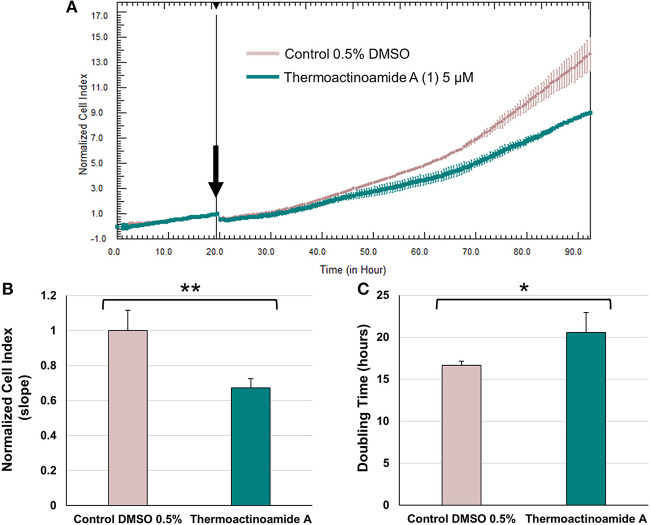
Effects of thermoactinoamide A (**1**) on the proliferation of BxPC-3 cells monitored in real time using the RTCA platform. **(A)** Normalized cell index (NCI) kinetics of the BxPC-3 cells exposed to 0.5% DMSO vehicle and 5 μM of thermoactinoamide A. Arrow shows the starting point of treatment of the cells. Each cell index value was normalized to this starting point. **(B)** Decrease of the slope of BxPC-3 proliferation curve describes the rate of change of NCI after 72 h drug treatment. Slope values of NCI curves are relative to controls treated with 0.5 % DMSO vehicle. **(C)** Doubling times of NCI of BxPC-3 cancer cells after 72 h treatment with 5 μM of thermoactinoamide A and 0.5% DMSO. Doubling time is the time required for a curve cell index value to double. Data are presented as mean ± SD; *n* = 2. Each experiment was performed in triplicate. *p < 0.05; **p < 0.005.

Potential growth inhibitory effects of thermoactinoamide A (**1**) were evaluated at a single dose exposure (5 μM) for 72 h against three different cancer cell lines, namely BxPC-3 (pancreatic carcinoma), PANC1 (pancreatic carcinoma), and 3AB-OS (osteosarcoma cancer stem cell line). As previously described (Teta et al., 2019), cell proliferation was monitored by the xCELLigence System Real-Time Cell Analyzer (RTCA), based upon dynamic measurements of electronic impedance alterations which are indicative of cell viability and morphology. At 5 μM concentration, thermoactinoamide A (**1**) was shown to exert selective, moderate antiproliferative activity against BxPC-3 cancer cells, as inducing a) a significant reduction in the slope of the real-time proliferation curve and b) a concurrent increase in tumor cell doubling time exclusively in this cancer cell type (Figure 6). On the other hand, PANC1 and 3AB-OS carcinoma cells were not sensitive to drug treatment (Figures S3, S4).

## Discussion

Thermoactinoamides are lipophilic cyclopeptides, putatively assembled through a non-ribosomal peptide synthetase system consisting of two distinct trimodular NRPSs, namely ThdA and ThdB. Notably, the Thd synthetase lacks a typical thioesterase/cyclase, underlying the existence of unique off-loading and macrolactamization mechanisms to yield the final product.

Integration of genome mining and LC-MS/MS-based molecular networking has provided a powerful approach for a comprehensive investigation of the NRPS metabolome of *T. vulgaris* DSM 43016. The LC-MS/MS-based molecular networking represents a robust and fast technique for dereplication of complex extracts (Esposito et al., 2019) as well as for detection of novel variants of known natural products (Teta et al., 2015; Grauso et al., 2019) thereby giving an exhaustive metabolic profiling of a certain bacterial strain (Teta et al., 2016; Paulo et al., 2019). Molecular networking performed on the extracts of *T. vulgaris* DSM 43016 led to the discovery of eleven different members of the thermoactinoamide family, five of them being new compounds.

Bioinformatic analysis of the *thd* gene cluster significantly supported detection and stereo-structural determination of thermoactinoamides. Prediction of substrate selectivity of adenylation domains was useful to infer possible amino acid combinations within the thermoactinoamide cyclo-hexapeptide ring and, therefore, allow targeted detection of novel congeners. Moreover, genome mining allowed for the settling of some doubts about structure and configuration of those compounds either (a) present in amounts too small for structure elucidation by NMR, or (b) showing 2-fold symmetry and isobaric amino acids, hampering unambiguous interpretation of MS2 data. For example, bioinformatic analysis of the *thd* gene cluster was used to locate l-Tyr and d-Tyr residues in positions 1 and 3, respectively, within thermoactinoamide D (**4**), as well as to determine the amino acid sequence of thermoactinoamides E (**5**), J (**10**), and K (**11**).

Combining the relaxed substrate selectivity of some adenylation domains with the iterative and/or alternative use of specific modules, the thermoactinoamide synthetase is able to generate a wide chemical diversity. Detection of fully aliphatic thermoactinoamide congeners, containing only two d-configured amino acids, implies that thermoactinoamide synthetase may deviate from the collinearity rule and adopt an alternative strategy for their assemblage. Indeed, it appears that ThdB works as a standalone, trimodular iterative NRPS to create peptides E (**5**), J (**10**), and K (**11**). However, how Thd NRPS is programmed to assemble these compounds still remains unclear. Two possible biosynthetic models can be proposed for the ThdB-driven biosynthesis of **5**, **10**, and **11**: the parallel model and the combinatorial model. In the parallel model, reported for iterative bacterial NRPS (Yu et al., 2017), such as gramicidin S synthetase (Hoyer et al., 2007), ThdB catalyzes condensation of two tripeptidyl monomer intermediates simultaneously tethered to the enzyme. As TE domains play a crucial role in the cyclooligomerization mechanism by iterative bacterial NRPS (Yu et al., 2017) a homologous enzyme should be present in the *thd* gene cluster too, but it could not be identified by *in silico* prediction.

In the combinatorial biosynthetic model, proposed for the first time for vatiamides biosynthesis (Moss et al., 2019), the ThdB could choose as a cognate partner either a ThdA or another ThdB NRPS via specific intermodule interaction motifs. Therefore, this combinatorial capacity results in the formation of two distinct assembly lines, namely ThdA-ThdB and ThdB-ThdB megasynthases, with the latter being involved in biosynthesis of variants E (**5**), J (**10**), and K (**11**). Decoding the intriguing biosynthesis of thermoactinoamides, as well as elucidating the non-canonical head-to-tail cyclization mechanism of the linear peptide, will be the aim of our future work.

Beside antimicrobial activity, thermoactinoamide A displays a moderate antiproliferative effect against a pancreatic tumor model, in the low micromolar range. Due to a chemical scaffold endowed with promising biological properties, our efforts will be addressed toward sustainable production of thermoactinoamides by microbial fermentation, in the native strain or in heterologous hosts, aiming to perform a deeper investigation of their pharmacological activity.

## Data Availability Statement

All information and datasets for this study are included in the article/Supplementary Material.

## Author Contributions

RT, GD, and AM: conceptualization. RT and GD: data curation, investigation, and writing—original draft. GD, AM, VC, and RT: funding acquisition. AM, VC, and RT: supervision. AM and VC: writing—review and editing.

## Conflict of Interest

The authors declare that the research was conducted in the absence of any commercial or financial relationships that could be construed as a potential conflict of interest.

## Footnotes

^1^https://img.jgi.doe.gov

^2^Leibniz Institute DSMZ-German Collection of Microorganisms and Cell Cultures GmbH; https://www.dsmz.de/.

^3^Global Natural Products Social Molecular Networking: http://gnps.ucsd.edu//.
