# Supplementary material for: Identification of the Biosynthetic Gene Cluster of Thermoactinoamides and Discovery of New Congeners by Integrated Genome Mining and MS-Based Molecular Networking
Source: Front Chem. 2020 May 21;8:397. doi: 10.3389/fchem.2020.00397 (PMC7253712; doi:10.3389/fchem.2020.00397)
Supplement: Supplementary file 1 [file Data_Sheet_1.PDF]

Supplementary Material

Identification of the Biosynthetic Gene Cluster of Thermoactinoamides and Discovery of New Congeners by Integrated Genome Mining and MS-based Molecular Networking

Gerardo Della Sala[1], Alfonso Mangoni[2], Valeria Costantino[2], Roberta Teta[2*]

[1] Laboratory of Pre-clinical and Translational Research, IRCCS-CROB, Referral Cancer Center of Basilicata, Rionero in Vulture, Italy.

[2] Dipartimento di Farmacia, Università degli Studi di Napoli Federico II, Napoli, Italy

Table of Contents

1. Supplementary Tables .... p. 3

Table S1. Average Nucleotide Identity (ANI) calculations .... p. 3

Table S2. NaPDos phylogenetic analysis of condensation and epimerization domains .... p. 3

2. Supplementary Figures .... p. 4

Figure S1. Signature sequences of DCL and LCL domains of the ThD synthetase........ p. 4

Figure S2. Signature sequences of E domains of the ThD synthetase .... p. 5

Figure S3. Effects of thermoactinoamide A (**1**) on the proliferation of 3AB-OS cells........ p. 6

Figure S4. Effects of thermoactinoamide A (**1**) on the proliferation of PANC-1 cells........ p. 7

Figure S5. Reference-guided *de novo* assembly of *thd* gene cluster .... p. 8

Figure S6. MS/MS spectrum of thermoactinoamide A (**1**). .... p. 9

Figure S7. MS/MS spectrum of thermoactinoamide G (**7**). .... p. 9

Figure S8. MS/MS spectrum of thermoactinoamide H (**8**). .... p. 10

Figure S9. MS/MS spectrum of thermoactinoamide I (**9**). .... p. 10

Figure S10. MS/MS spectrum of thermoactinoamide J (**10**). .... p. 11

Figure S11. MS/MS spectrum of thermoactinoamide K (**11**). .... p. 11

Figure S12. MS/MS spectrum of thermoactinoamide D (**4**). .... p. 12

Figure S13. MS/MS spectrum of thermoactinoamide E (**5**). .... p. 12

Figure S14. ^{1}H NMR spectrum of thermoactinoamide D (**4**)..p. 13
Figure S15. COSY spectrum of thermoactinoamide D (**4**). ....p. 14
Figure S16. HSQC spectrum of thermoactinoamide D (**4**). ....p. 15
Figure S17. HMBC spectrum of thermoactinoamide D (**4**). ....p. 16

1 Supplementary Tables

Genome 1 - Accession	Genome 1	Genome 2	ANI (%)
NZ_REFP00000000	*T. vulgaris* DSM 43016	*Thermoactinomyces* sp. AS95	99.65
NZ_REFP00000000	*T. vulgaris* DSM 43016	*Thermoactinomyces* sp. CDF	99.56
NZ_REFP00000000	*T. vulgaris* DSM 43016	*Thermoactinomyces* sp. Gus2-1	99.67
LSVF00000000	*Thermoactinomyces* sp. AS95	*Thermoactinomyces* sp. Gus2-1	99.71
LFJU00000000	*Thermoactinomyces* sp. CDF	*Thermoactinomyces* sp. AS95	99.61
JPZM00000000	*Thermoactinomyces* sp. Gus2-1	*Thermoactinomyces* sp. CDF	99.58

Table S1. Average Nucleotide Identity (ANI) calculations show that *Thermoactinomyces* sp. AS95, *Thermoactinomyces* sp. CDF and *Thermoactinomyces* sp. Gus2-1 affiliate with *Thermoactinomyces vulgaris* DSM 43016 as the ANI value is higher than 95%. ANI values have been calculated using the Pairwise ANI tool available at the DOE Joint Genome Institute website (https://img.jgi.doe.gov).

	Query id	Database match id	percent identity	align length	e-value	pathway product	domain class
☐	ThdA_C*	tyroc3_C1_DCL	24	275	1e-18	tyrocidin	DCL
☐	ThdA_C1	tyroc3_C3_LCL	47	419	2e-112	tyrocidin	LCL
☐	ThdA_E1	surfa5_C4E	44	446	2e-113	surfactin	epim
☐	ThdA_C2	bacit3_C1_DCL	40	426	4e-98	bacitracin	DCL
☐	ThdA_E2	tyroc2_C4E	48	451	9e-106	tyrocidin	epim
☐	ThdB_C3	grami2_C1_DCL	42	430	2e-104	gramicidin	DCL
☐	ThdB_C4	tyroc3_C2_LCL	48	419	1e-113	tyrocidin	LCL
☐	ThdB_C5	tyroc3_C4_LCL	50	419	6e-117	tyrocidin	LCL
☐	ThdB_E3	grami1_C1E	42	442	3e-94	gramicidin	epim

Table S2. NaPDos phylogenetic analysis allowed detection and prediction of condensation and epimerization domains within the *thd* gene cluster. A specific domain class has been assigned to each domain. An LCL domain catalyzes a peptide bond between two L-amino acids; a DCL domain links an L-amino acid to a growing peptide ending with a D-amino acid; an E domain catalyzes inversion of stereochemistry in amino acids.

2 Supplementary Figures

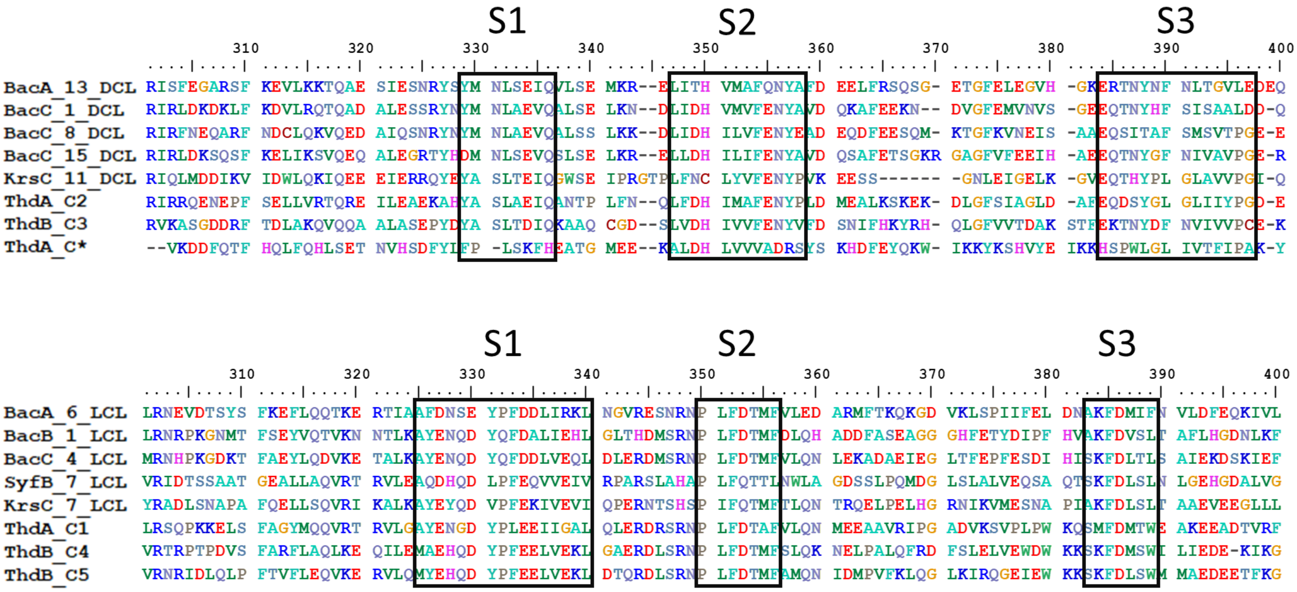

Figure S1. Signature sequences of DCL and LCL domains of the thermoactinoamide synthetase**.** As reported by Caradec *et al.*,[1] C and E domains can be divided into 2 sub-regions called Up-Seq and Down-Seq. Down-Seq regions of DCL and LCL domains have been aligned and signature sequences have been surrounded by black squares. The Down-Seq regions are specific to the enzymatic activity of the domain and their signature motifs (S1-S2-S3) can be used to predict domain class. Protein sequences were aligned with BioEdit.[2]

---

1. Caradec, T., Pupin, M., Vanvlassenbroeck, A., Devignes, M. D., Smaïl-Tabbone, M., Jacques, P., and Leclère, V. (2014). Prediction of monomer isomery in Florine: a workflow dedicated to nonribosomal peptide discovery. *PloS one*, 9(1), e85667. doi:10.1371/journal.pone.0085667.

2. Hall, T.A. (1999). BioEdit: a user-friendly biological sequence alignment editor and analysis program for Windows 95/98/NT. *Nucl. Acids. Symp. Ser.* 41, 95-98.

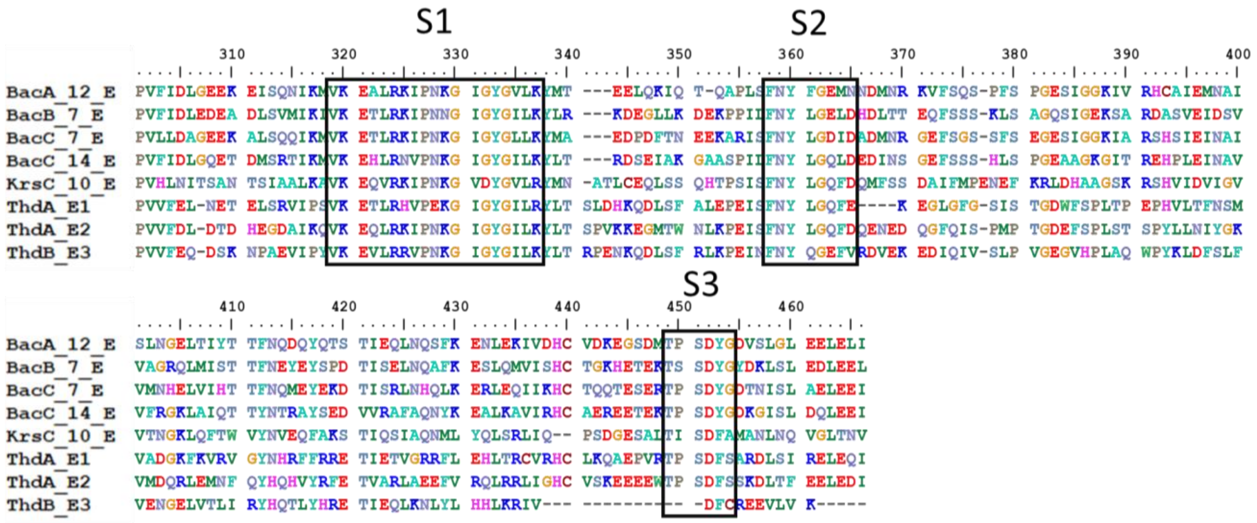

Figure S2. Signature sequences of E domains of the thermoactinoamide synthetase. Down-Seq regions of E domains have been aligned and signature sequences have been surrounded by black squares. The Down-Seq regions are specific to the enzymatic activity of the domain and their signature motifs (S1-S2-S3) can be used to predict domain class. Protein sequences were aligned with BioEdit.

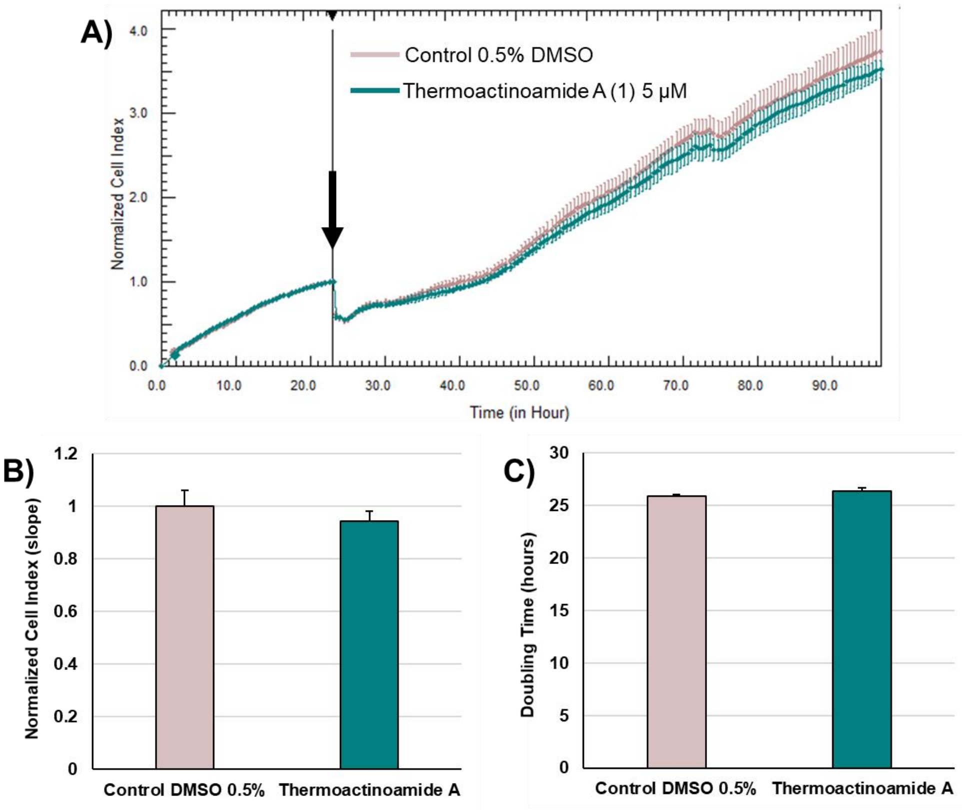

Figure S3. Effects of thermoactinoamide A (**1**) on the proliferation of 3AB-OS cells monitored in real time using the RTCA platform. A) Normalized cell index (NCI) kinetics of the 3AB-OS cells exposed to 0.5 % DMSO vehicle and 5 µM of thermoactinoamide A. Arrow shows the starting point of treatment of the cells. Each cell index value was normalized to this starting point. B) Slope of 3AB-OS proliferation curve is unaffected after 72h drug treatment. Slope values of NCI curves are relative to controls treated with 0.5 % DMSO vehicle. C) Doubling times of NCI of 3AB-OS cancer cells after 72h treatment with 5 µM of thermoactinoamide A and 0.5% DMSO. Doubling time is the time required for a curve cell index value to double. Data are presented as mean ± SD; *n*=2. Each experiment was performed in triplicate.

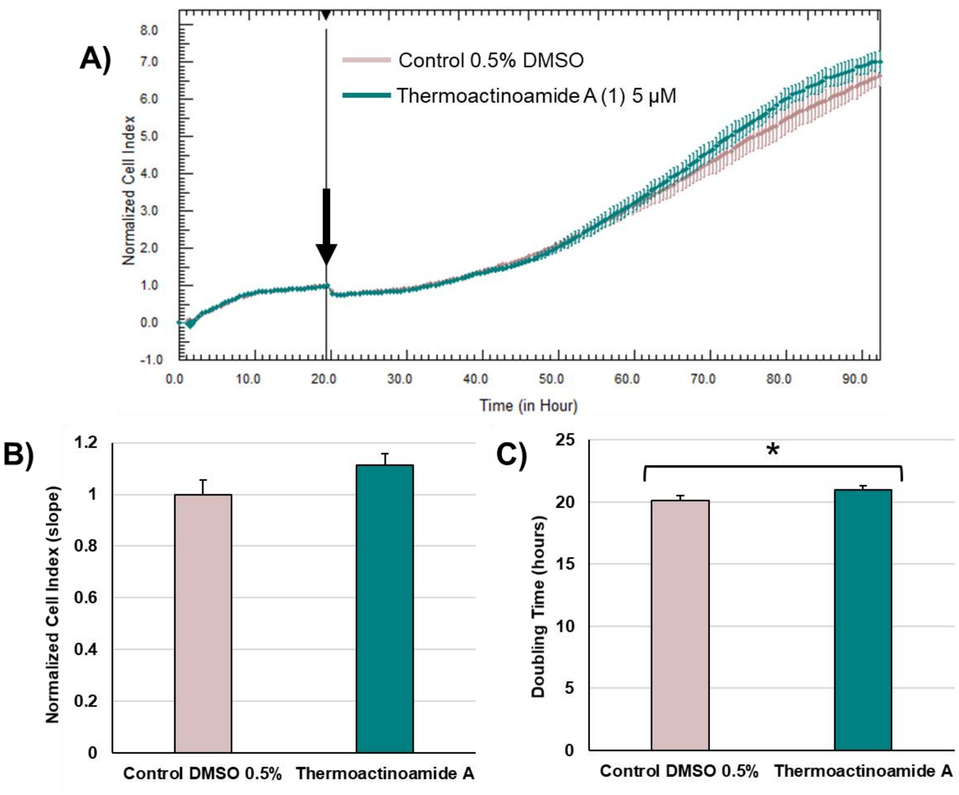

Figure S4. Effects of thermoactinoamide A (**1**) on the proliferation of PANC-1 cells monitored in real time using the RTCA platform. A) Normalized cell index (NCI) kinetics of the PANC-1 cells exposed to 0.5 % DMSO vehicle and 5 μM of thermoactinoamide A. Arrow shows the starting point of treatment of the cells. Each cell index value was normalized to this starting point. B) Slope of PANC-1 proliferation curve is unaffected after 72h drug treatment. Slope values of NCI curves are relative to controls treated with 0.5 % DMSO vehicle. C) Doubling times of NCI of PANC-1 cancer cells after 72h treatment with 5 μM of thermoactinoamide A and 0.5% DMSO. Doubling time is the time required for a curve cell index value to double. Data are presented as mean ± SD; *n*=2. Each experiment was performed in triplicate. * $p < 0.05$.

A)

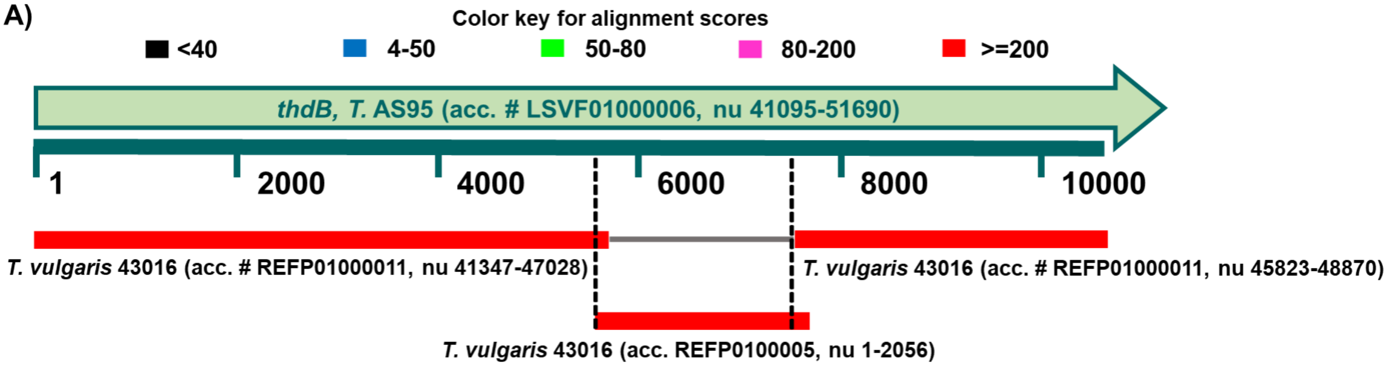

B)

Sequence ID	Nucleotides	Score (bits)	Expect value	Identities (%)	Gaps
REFP01000011	41347-47028	11216	0.0	5676/5682 (99.89%)	0/5682
REFP01000011	45823-48870	5971	0.0	3039/3048 (99.70%)	0/3048
REFP0100005	1-2056	4052	0.0	2053/2056 (99.85%)	0/2056

Figure S5. Reference-guided *de novo* assembly of *thd* gene cluster from *T. vulgaris* DSM 43016. The *thd* gene cluster from *T. vulgaris* DSM 43016 was split on two distinct contigs (contig Ga0070019_105 - accession: REFP01000011; contig Ga0070019_114 - accession: REFP01000005). These two contigs were re-assembled using as template the contig containing the intact *thd* gene cluster from *Thermoactinomyces* AS95 (contig NODE_4; accession: LSVF01000006). (A) Contigs were aligned and re-assembled using the blastn suite and the sequence analysis software SeqMan (DNASTAR v.5.00). Reference-guided assembly led to the integration of contig Ga0070019_114 (accession: REFP01000005) into contig Ga0070019_105 (accession: REFP01000011). Particularly, the smaller contig Ga0070019_114 was integrated into the ThdB encoding region, as shown in the sketch above. (B) Quality and significance of blastn alignments using *thdB* from *Thermoactinomyces* AS95 as query and contig Ga0070019_105 (REFP01000011) and Ga0070019_114 (REFP01000005) as subjects. Abbreviations: *acc.#*, GenBank accession number; *nu*, nucleotides; *T.*, *Thermoactinomyces*.

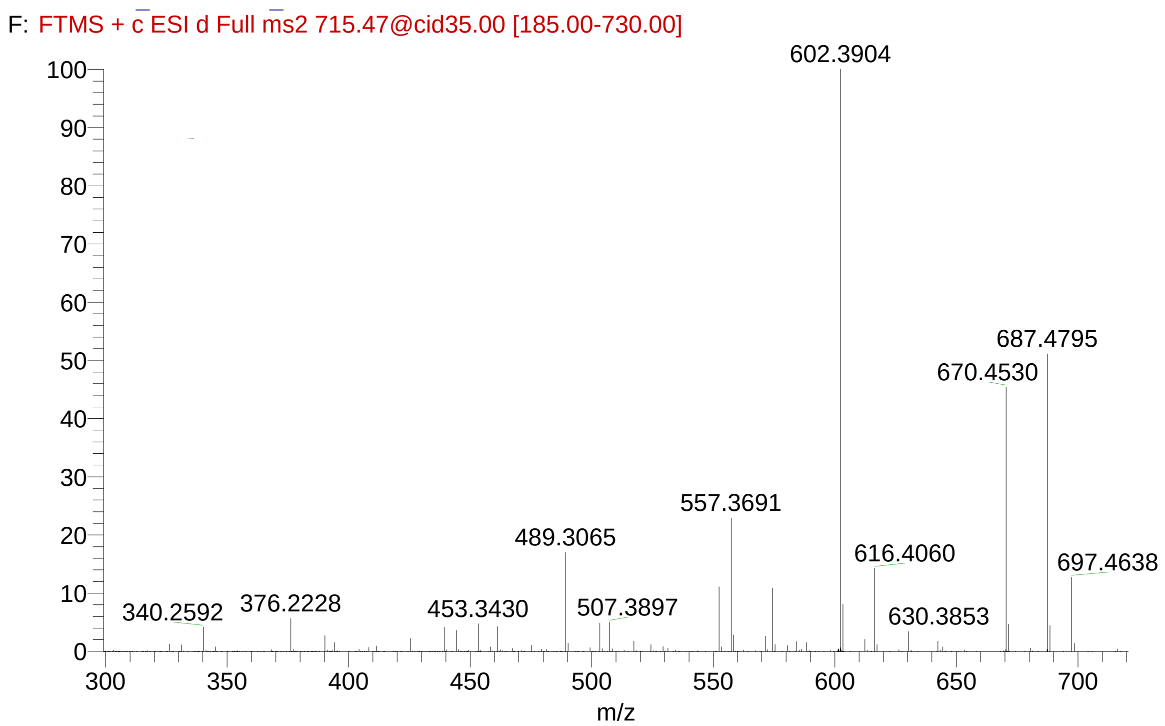

Figure S6. MS/MS spectrum (parent ion at *m/z* 715.47) of thermoactinoamide A (**1**).

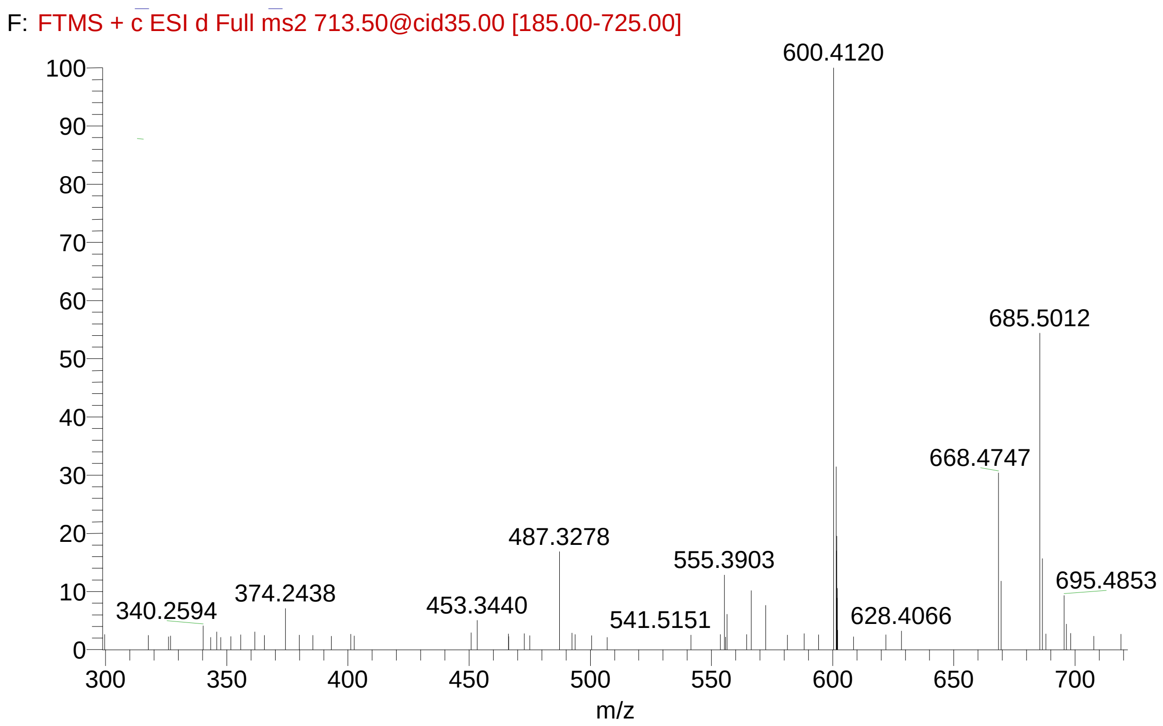

Figure S7. MS/MS spectrum (parent ion at *m/z* 713.50) of thermoactinoamide G (**7**).

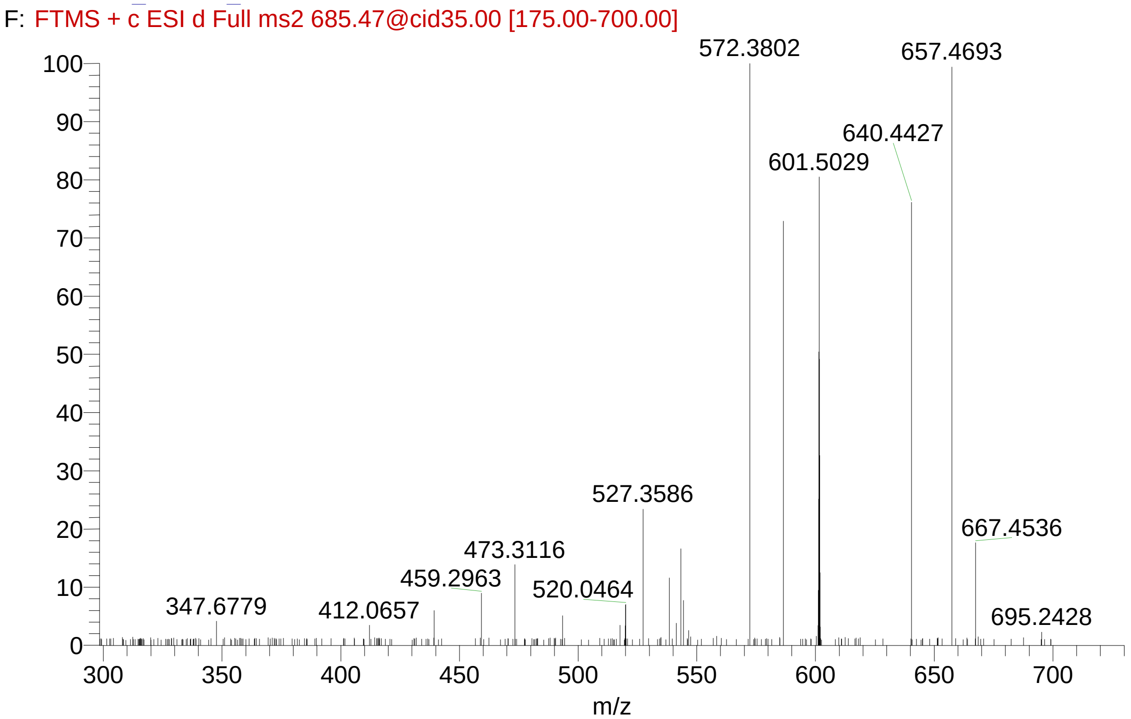

Figure S8. MS/MS spectrum (parent ion at *m/z* 685.47) of thermoactinoamide H (**8**).

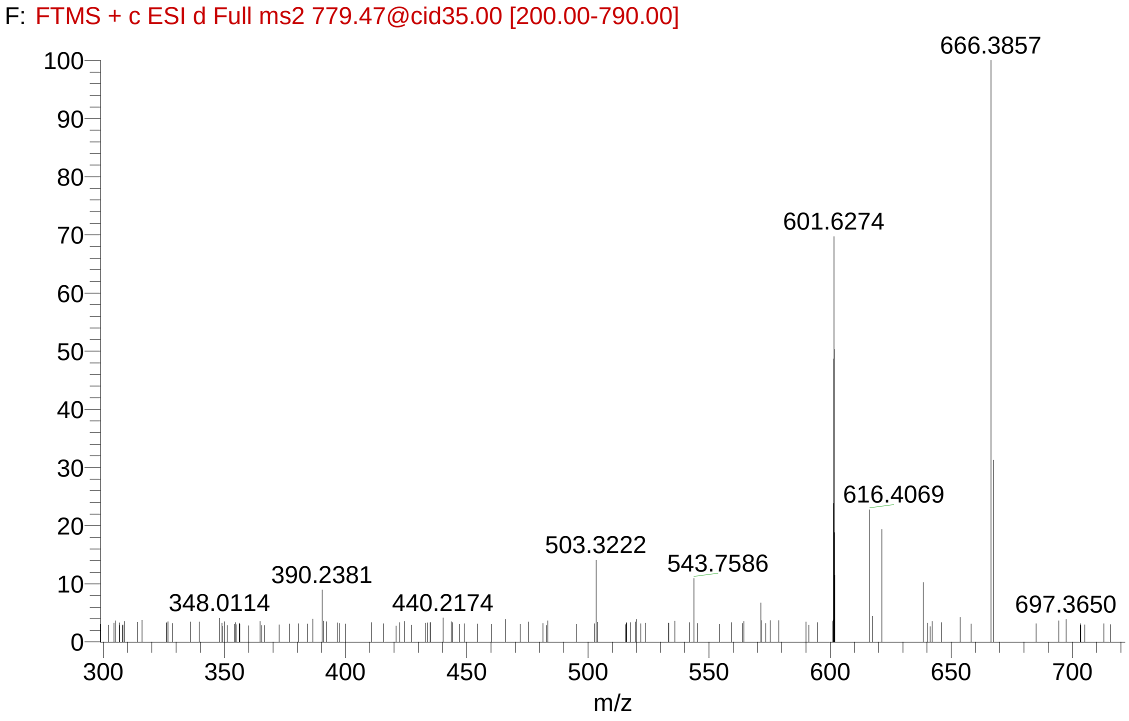

Figure S9. MS/MS spectrum (parent ion at *m/z* 779.47) of thermoactinoamide I (**9**).

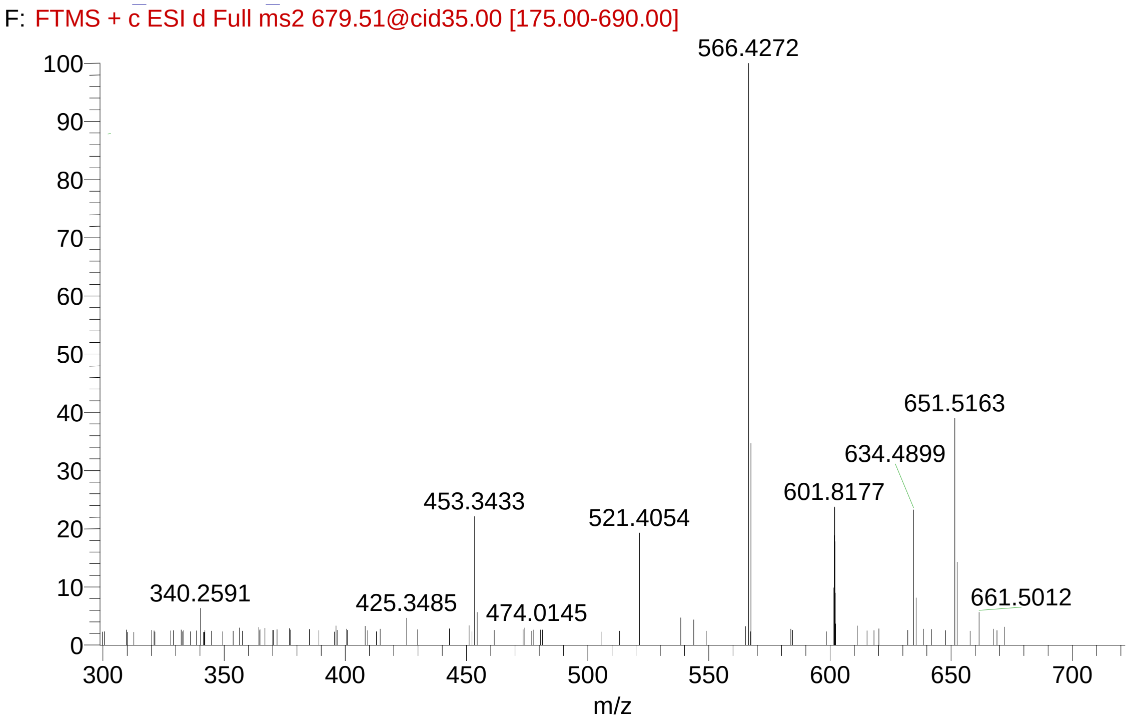

Figure S10. MS/MS spectrum (parent ion at *m/z* 679.51) of thermoactinoamide J (**10**).

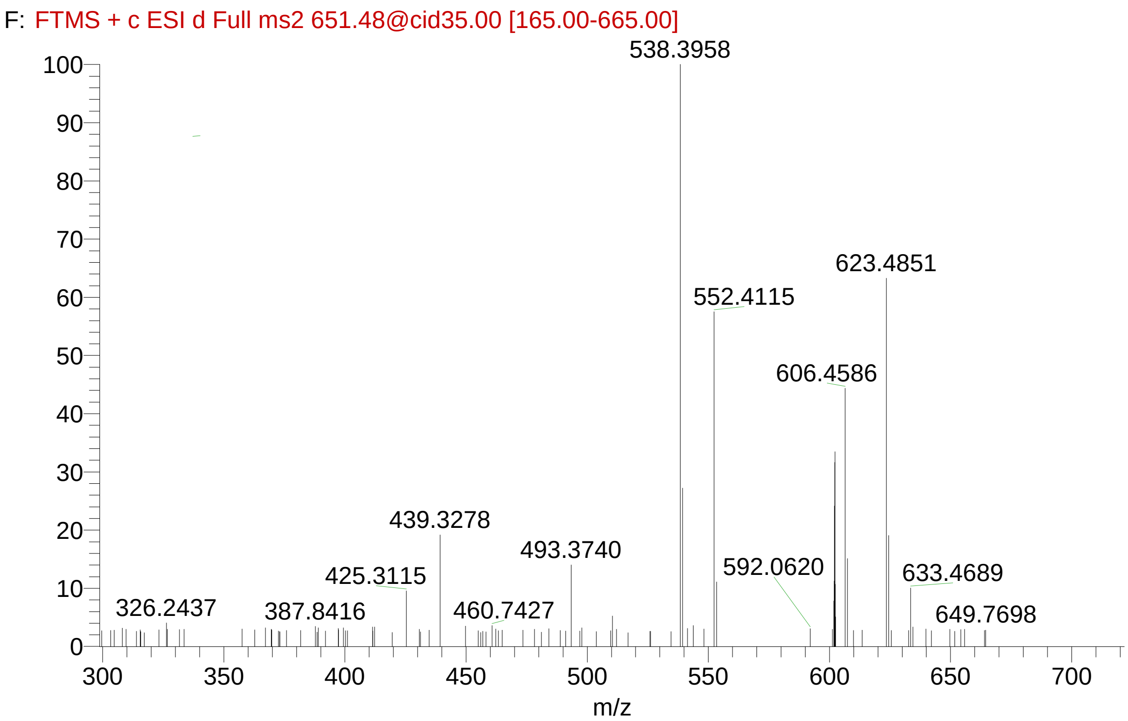

Figure S11. MS/MS spectrum (parent ion at *m/z* 651.48) of thermoactinoamide K (**11**).

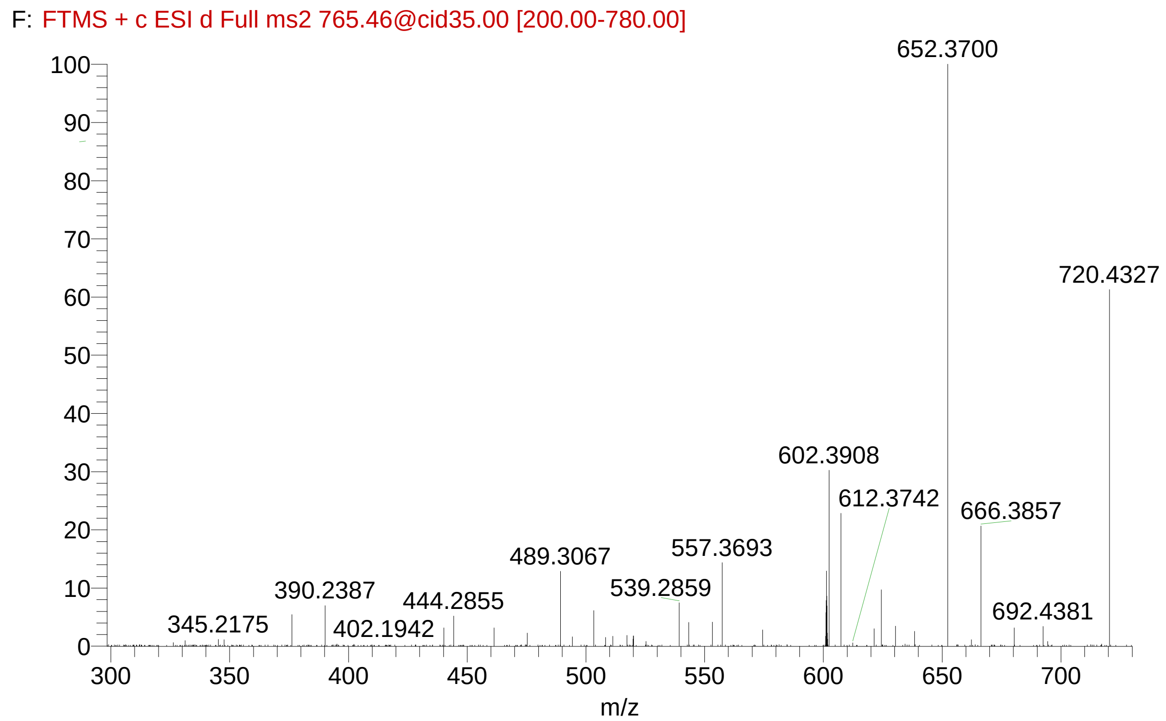

Figure S12. MS/MS spectrum (parent ion at *m/z* 765.46) of thermoactinoamide D (**4**).

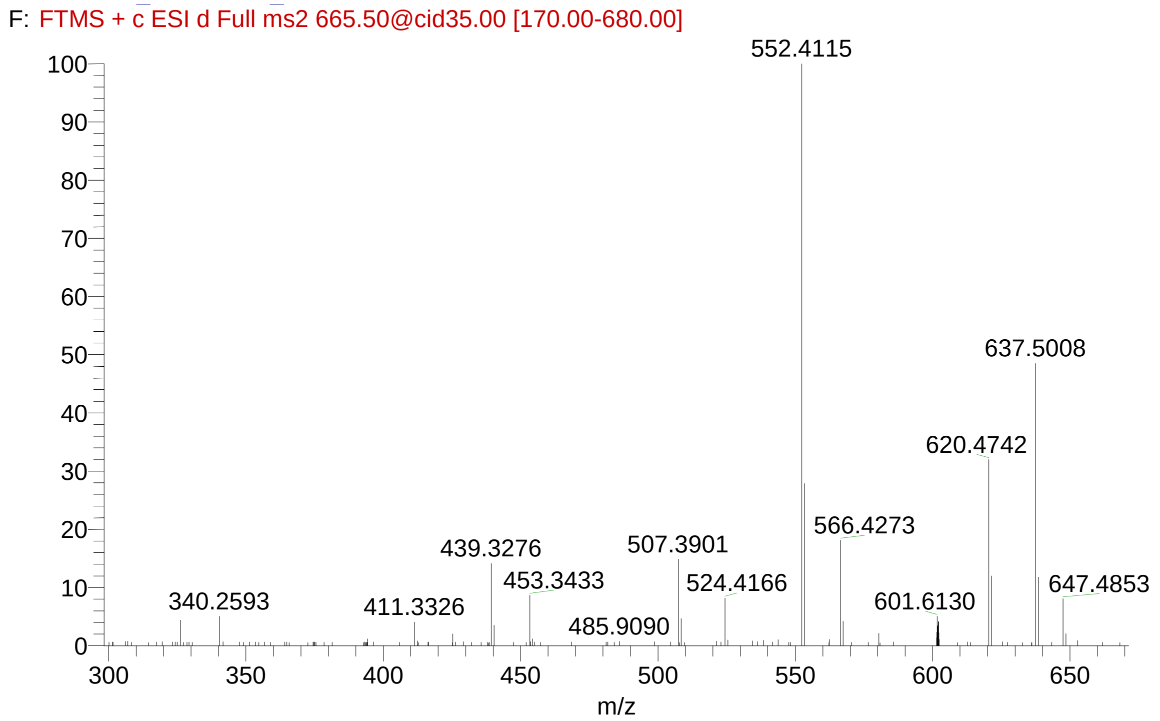

Figure S13. MS/MS spectrum (parent ion at *m/z* 665.50) of thermoactinoamide E (**5**).

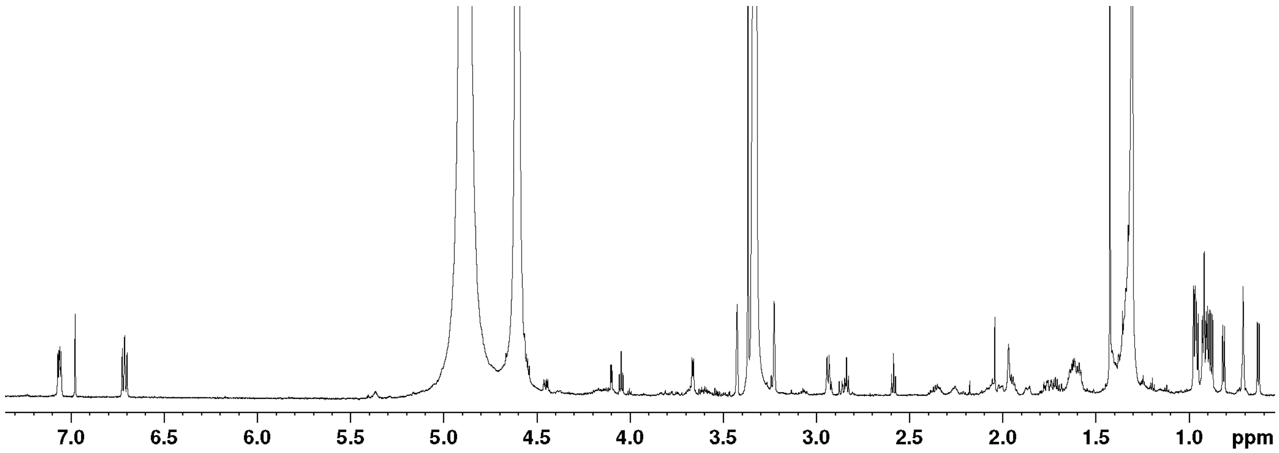

Figure S14. ^{1}H-NMR spectrum of thermoactinoamide D (**4**) (700 MHz, CD_3OD)

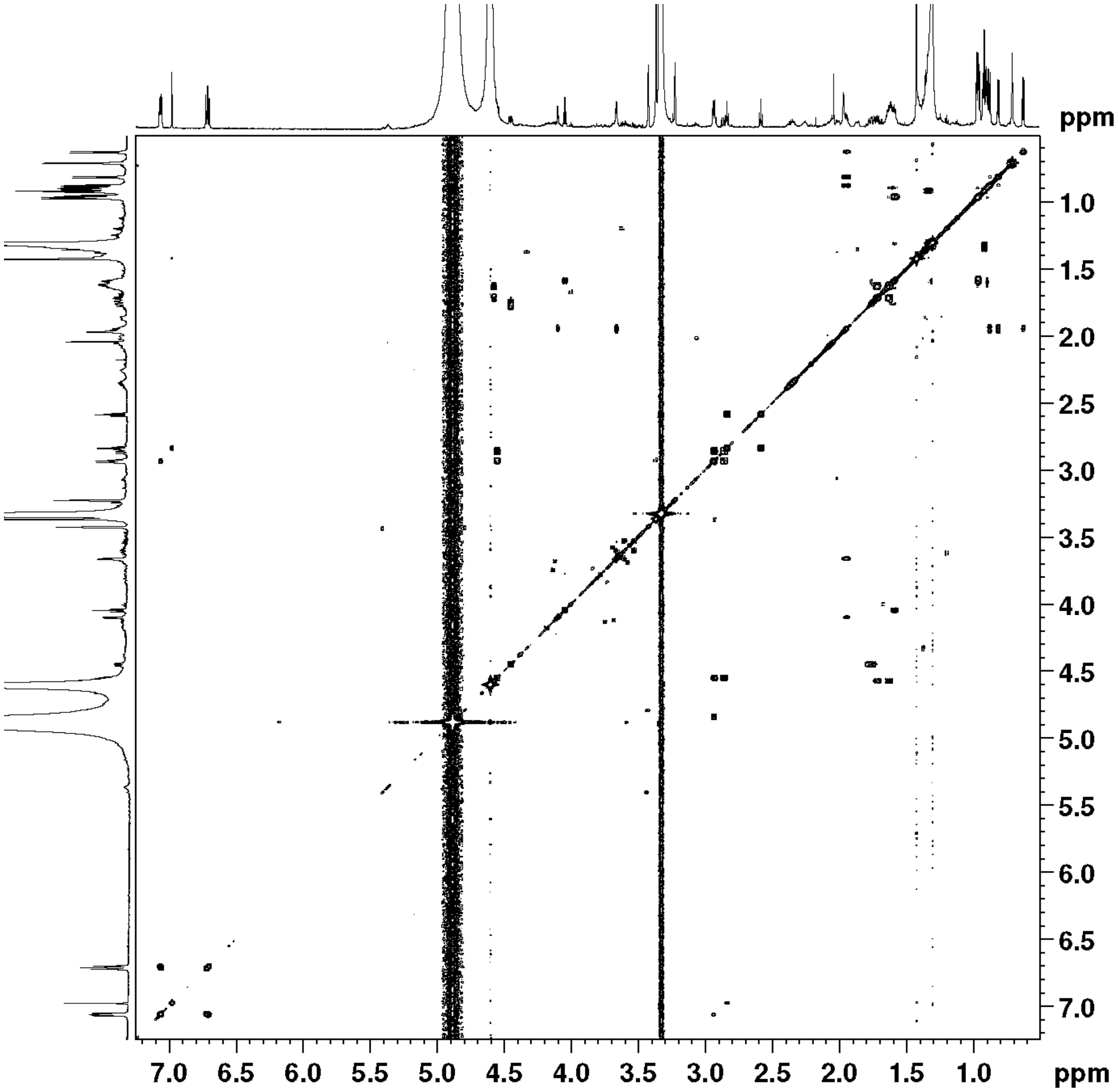

Figure S15. COSY spectrum of thermoactinoamide D (**4**) (700 MHz, CD_3OD)

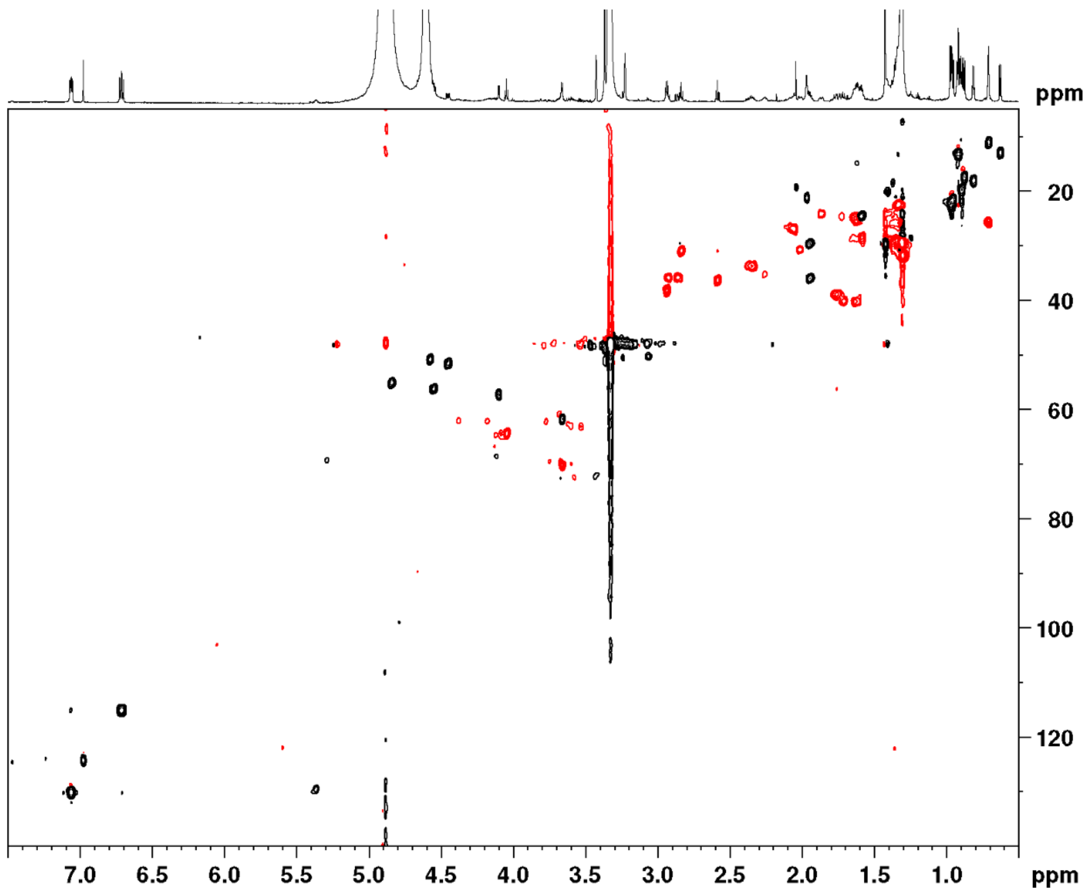

Figure S16. HSQC spectrum of thermoactinoamide D (**4**) (700 MHz, CD_3OD)

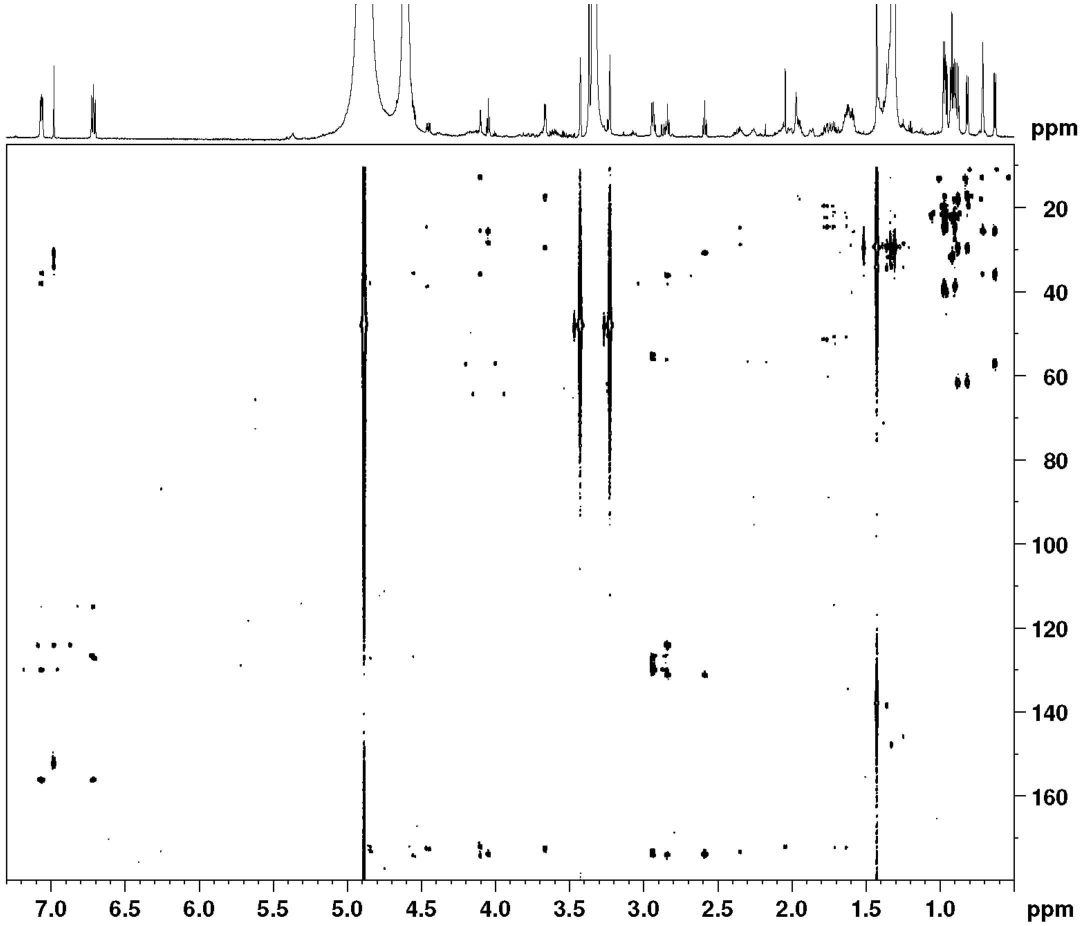

Figure S17. HMBC spectrum of thermoactinoamide D (**4**) (700 MHz, CD_3OD)
