# Supplementary material for: Identification of the Biosynthetic Gene Cluster of Thermoactinoamides and Discovery of New Congeners by Integrated Genome Mining and MS-Based Molecular Networking
Source: Front Chem. 2020 May 21;8:397. doi: 10.3389/fchem.2020.00397 (PMC7253712; doi:10.3389/fchem.2020.00397)
Supplement: Supplementary file 2 [file Data_Sheet_2.docx]

>de novo assembled contig_Thermoactinomyces vulgaris DSM 43016

AGGGGGAGCCAATCCCAAAAAGCCAGTCTCAGTTCGGATCGCAGGCTGCAACTCGCCTGCGTGAAGCCGGAATCGCTAGTAATCGCGGATCAGCATGCCGCGGTGAATACGTTCCCGGGCCTTGTACACACCGCCCGTCACACCACGAGAGTTTGCAACACCCGAAGTCGGTGAGGCAACCTTTTAGGAGCCAGCCGCCGAAGGTGGGGCAGATGATTGGGGTGAAGTCGTAACAAGGTATCCCTACCGGAAGGTGGGGATGGATCACCTCCTTTCTAAGGAGCTCATTTCGAGCAAAAACGTTTCAAAGAGTCATGTCTCACACTCTTTAGTTTTCAGGGAATACTGGAAAAATCCCCGCAGACCACGGTCTGCGGGGATTTTTGTTTGCGACGGTTGCATTTTGAAACAAAAAAGGGAAGATACCCCCTGAAACGGGGTATGCTAAAATGAAAAGATGCCGGGAAAATGATTGGAAGATGAGGTGTTTCAAATGGTCAGGAGGGCAAGAAGAGAAAAAAAAGAACAAGAAGAACAGAAAAAAAAGTTATCCCCGCGTCAATATCACGTCACTCAGGAAAATGGAACCGAACCTCCTTTTCAAAATGAATACTGGGATCATGAAGAAGAAGGAATCTATGTGGATGTGGTTTCGGGTGAACCGCTTTTCAGTTCATTGGACAAATTTGATGCCGGATGCGGTTGGCCCAGTTTTACCAAGCCGATTCACGAAGATCAGATTGTGGAAAAGGAAGATTACAGCCACAACATGATCCGTACCGAAGTGAGGAGCCGGGAAGGAGACTCTCATTTGGGGCATGTGTTTAATGACGGGCCGCTGCCGACAGGGCTCCGCTATTGCATCAACTCGGCGGCTTTAAGGTTTATCCCGAAAAAAGATTTGGAAAAAGAAGGTTATGGGGAATATCTGTCCCTGTTTGATAAAAAGGATTCATGACAAAGAGAAAAGCGTTTGCCGGGTCGGCAAACGCTTTTTTTATGGCAGGCATTAAGATTGGGCCAGAAGTTTTTCCAATTCATCCAGTTTCTCCTCAAAAACGTTCAGGGCTTGCTGAATCGGTTCCGGTGAAGTCATGTCGACGCCGGCTTTTTTCAATACTTCAATGGGATAGTCGGAACTTCCGGCTTTCAAGAAGGAAAGATAACGGGAGACGGCCGGTTTTCCTTCGCGCAGAATTTGTCCGGACAATGCCGCCGCGGCACTGAATCCTGTCGCATATTGGTAAACATAATAGTTGTAATAGAAGTGGGGGATGCGTGCCCATTCCAAACCGATTTCCGGATCGCTTATCATGTCGTTGCCGTGATACTTTTTGTTCAAATCATAGTACAACGAGGTGAGCAGTTCCGGTGTCAGCGCTTCTTTTGCCTGCGCTTTTTGATGAATGAGATGTTCAAATTCGGCAAACATGGTTTGGCGGAAAACCGTTGCCCGGAAGCCGTCGAGATAATGGTTCAAGAGATACAGACGCTTTTGTTTGTCGTCGGTTTGTTTGAGCAAATAATCATTGAGCAAGGCTTCATTGCAAGTCGATGCCACTTCCGCAACAAAAATGGAATAGTTGCCATAGACAAACGGTTGGTTTCTGTTGGTGTAATAACTGTGGAGGGAGTGCCCCAGTTCATGTACCAGTGTGAACAAGCTGTCAATGCTGTCCTGCCAGTTCATCAGGATGTAGGGGGCCGTTCCGTAAGCTCCTGAAGAATAGGCGCCGCTTCGTTTTCCTTTGTTCTCATAAACATCGATCCACCGATTGTCAAAAGCCTCTTTCAGGATGGATACATATTCCTCGCCCATGACACTTAACGCATCGAGCACAATTTTTTGTGCATCCGGGTAGGAAATTTTAAAAGAAGATTCTTCCACCAACGGGGTGTAAATGTCGTACATGTGCATTTCTTCCAAGCCCAGTGCTTTTTTGCGGATGCGCATATACCGGTGCAGCAAGGGCAAATTTTGGTTGACGGTATCCACCAATTGATCATAGACGGTTTCAGGTATGCTGTTCCGGCTTAAAGCGGCATGCCGTGCCGAATCATAGCCGCGGACTTCTGCATAAAAATTATTTTTCTTGATGGCCCCGCTTAAGGTGCTGGCAAAAGTGTTTTTATATTTGTCGTATGTGGAGTATACGGCATGAAACGCGTCTTTTCTGACGCGGCGGTCCGGGCTTTCCATCAATTTCGTGTACCGGCCGTGTGTCACTTTGACTTCTTCCCCGTTTTCATCCCGGATCGAGGGAAACTCCAAGTCTGCATTGTTCAGCATGCCGAAGGTGTTGGCGGAGGCCCCCAGGACTTCACCGGCTTGGGCTAGGAGTTTTTCTTCTTTTTCCGATAAAACATAGGGACGCTTTTTGTTTAACTCTTCCAGAGCGTGTTTGTATACCTGCAATTCTTCGTTTTCTTCCAAAAAACGCGTGATTTTCTCTTCGGGGATGGAAAGGATCTCCGGAGTGATGAAGGACAAGGCTCCGGAAACTTCCGTATACAAACTTTTTGCGCGGTCATTGAGGGCTTGATAAAAGGATTGGGTGGTGTCCTGATCAAACCGCATATGCGCATATGTATAAAGTTTGTCAAGTTTGTAGAGCAATTGATCCTGGAATTTCAGCGCTTTTAACAAATCCTCAGCAGACCGTCCGAGCTTCCCTTTGAACCGGCTGATCTCAGGGATCATTTGTTTGATGTTTTGATATTCTTCTTCCCATTTCTCATCGTTTTCAAAAATATCTTCAAGACGCCATTTATATTCATCCGGAATTTCTGAGCGAAGAGGAAGTTTGGATGAGGATTGGGTCATGATTATACCTCCTTAATCAGTTCTGTATTCTCCATTATACTACTCGGCCGGGGAATTCAGAAACCATTCCTTTTATTGGGTTGTGTATAATTTTTTTATTAATTTGCATAATATTGGGGTGATCATCATGAAAGGGAGGTACAAGAAGTGAGCAATCTGGAGCGTTTCAGAAGCCGGAGAAATCTTTACAACCCGGTACATCCTTTGTCACAAATCCATTCGGAAATCAACCGCACATTTGAACGTTTTTTTGAAAATCCGATCTGGAATGAGTCGATGCCAAAAATGCCCGCCATCAACATGAAAGAGGAAGGGAACCAGTATGTCATCGAGGCGGAAATGCCCGGCCTCAGGGATGAAGATGTTGAAATAGAGGTGCACGGCGGACAATTAACGATCAAAGGGGAACGGAAACAAGAATCGAAAAAAGAAGGAGAGCACATGTATATTGAAGAGAGGAGTTATGGTTCGTTTTACCGGACCGTCACCCTTCCGCAGGATGCCAACTTGGATCATATCACAGCCGATTATGAAAACGGGCTTCTCATAATCACGATTCCGAAGGATCCGGAAAAAGAACCGAAGAGAATTAAAATCAACCGGAAACCTTCTTCATGATCGTGCGGGAAAAATGAAAAAAACCAACGGGGCCGTATGGGACACGGCCCTTTTTTTTTGGAAAGAAGCCGGCACCGGGTTCCTCCTAATTTTTCCCGGATGGAGATGTGAGTAATCAGAGAAGGATGATCGCATACTATCACCAAATCCATTTCAATTTCAGGAAGGTGTAAGGATTATGCAAAATGTTGAAAACATGGAAAACGTTCTTTCCTACATTCATTCCGAGCTGAACCGCATTGAAACGATGGCGGGTACATTGGCCACAATTGAACAGGACCATTACCGGAAGCTGACGAATTTCGATCATCGCAAATTGGTGGATATTGCGGTCGAAGAACAGAATGCCGCCCGTCAGTTGGGAACGGTCAAGCAAATGTGCTTGTCGATGGCGCAAAAAATTGAAGAACTTCAAAATTCGTTGGGGCAGGGTGAAGCCAAGGAGCGTGTCCATCGTGCTGAAGTTCATTGAAAGGATGATCCGGTCCGCCATTGACCGGTCTGTCGATCAAGCGTTGACCAGGATGATCCGGGACCAATATACCGAGAATTTGTTTGAAATGGTGCCGGCCTCGAAAAAGGTGGGGGTGACCAACCTCATGGAAATCACCATGAGGGCCAATCAGGGCACACCCATCTCCCGACCCTTGGGAACCCCTGTTCCTATGTCCCCGTGGGAAAAAATCTTGTTCAATCCGGTTCACCTGTTCCGCTTTCCCACTCCGGAAAACGTCGGAATCAACACTTCGGTCACGATTGGACAGCGGGCCAAAAAACCATTGACGATTTCCATTCCGATCATGATTGCAGGCATGTCCTTCGGCGGTGCGTTAAGCAAAAAAACCAAAATTGCCCTCGCCAAAGCTGCCACCAAGGTGGGGACCGCGACCAATTCGGGGGAAGCGGGGCTCATGAAAGAGGAAAGGGAAGCGGCCGGTTTGTTTATCGGACAGTATAACCGGGGCGGATGGATGAACGATCCGAAAAAGTACAAAGCCTTGGATGCGATCGAGATTCAACTGGGGCAGGGGGCCCAAGGATCCGCTTCGCAGCGGACGGCCGCCCATAACATCGGCGAGGACTTCAGGGAAGTTTATGAGTTGCAACCCGGGGAAGATGCGGTGATCCATTCTCGGCTGCCCGGGGTCAATGGCAAGGAAGAGTTTGTCCAACTGGTCCGCAGACTGAAAGAGGAAACCGGGGTGCCGGTGGGACTGAAAATTGCTGCGACGCATCATCTTGAAAAAGAGTTGCAAATCGCCGTGGAAGCCGGAGTGGATTTTGTCACCGTTGACGGAGCCGAAGGCGGAACGCACGGGGGTGCACCCACTTTGCAGGATGATGTCGGATTGCCCACCCTCTTTGCCATTACAAGAGCATCCGAATTTCTTGCCCGAAAAAAGGTGAAACAGGATATTCAACTGATTGCCACAGGAGGATTGGTCACTCCCGGACGAATGCTGAAAGCCATCGCCTTGGGAGCCGATTGTGTGTATATCGGAACGGCGGCCTTGATGGCCTTGGCCAGCGAACAAATGGTCAAATCCGTACCCTTTGAACCTCCCACCAGCTTGTTGGTTTATTCGGGGAAAATGACCGACCAATTGGATGTGGACCAGGCGGCCATGAATCTGGTCCGTTATTTGAATGCTTGCGTGCGGGAAATGGAGCTCGTTTTGATATCGCTTGGAAAAACGGCTGTTGCCGATTTGACCAAATCGGATTTATGTACCATGGATCCTTTTATTGCCAAAGCAACGGGCATTGAGTTGGGCTATGTTGCCCCCGAAGACCAATCCCGCTTTTTTGAAGAAACCAACCCCTTGTTTTCCGTTCCACGGGACCGGGAGTTTGAAACCGGGCCGTTTGTCAGCCATTGAGAGATAAAAAATGCATCCCGGGGATACCGGCTTTTTCTGCCCCGGTCCCCGGGAAGAGGGACAAAAGCGGTTCGTTAAGGAAAAAGGAGCCGGCAAAGGCAATTGCTTCTTTTTTGGTGATTGTGTTATAATCACTTTGGCTATACTTATTGTAGATACATCTTGCGAGTGCAAAGAGTGGGTGGAAGCCCACTCTTTGCGTTTTGTTGCAGGTGTTTGAATTAGCCAAATAAGGAGGGTTGGCTTTGAGCCGACAAATTACAGAAGCCGTACAACAACTTGCCCGGCCGATTGTGGAAAAAGAAGGTTTGGAACTGGTCGATATCGAGTATAAAAAAGAAGGGCCAAATTGGTTTTTGCGCATTTTCATCGACCGGGAAGACGGGGGAGTTGATTTGGATGATTGCACCCGGATCAGTGAGATAGTGGGGGAAAAGCTGGACGAAGAAGACCCGGTGCCAGGGGCGTATATTCTGGAAGTGTCATCACCGGGTGCGGAAAGGCCGCTGACCAAGGAGAAGGATTTCCAAAAAGCTGTCGGTAAAAATGTATATATTACCACGTACGAACCCATTGAAGGCCAAAAAGTGTTTGAAGGGGTTTTGCACCAATTTGACGGTACCACCCTTACGGTCCATGAGAAGAAGAAGACATTTGAGATTCCACTCGAGAAGGTGGCCAAAGCACGATTAAGCGTCGTTTTTTAACGCGAAGGGAGGATTAGGCCGATGAATGCCGAGTTCATCGAGGCCCTTGATCAATTAGAAAAAGAAAAAGGGATCAGTAAAGATATTTTGATTGAAGCGATTGAAGCTGCATTGATATCTGGATATAAGCGCAACTTTCATTCTGCACAAAACGTACGGGTAGATGTGAACAGACAGACCGGACAGGTGCGTGTGTTTGCCCGCAAAACCGTCGTGGATGAAGTCGTCGACTCCCGCTTGGAAATTTCATTGGATGCGGCCCGGGAAATCGACCCCAATTATCAATTGGACGATATTGTGGAGATCGAAGTCACGCCGGCAGACTTTGGCCGGATTGCGGCTCAAACAGCGAAGCAGGTGGTGACGCAACGGATCCGGGAAGCGGAACGGTCAGCCATTTACGAAGATTTTGTTGACCGGGAAGAAGACATTGTCACCGGAATTGTGCAAAGAGCCGACAACCGTTACTATTACGTGGACCTTGGCCGGGTGGAAGCCTTGCTGCCCCACAATGAAGTCATCGCCGGAGAGCGGTTCAGCCACGGGGATCGCGTGAAGGTTTACATTACCCGGGTGGAAAAATCGACGAAAGGTCCGCAGATTTTCTTGTCCCGCACCCATCCCGGACTGTTGAAAAGGTTGTTTGAGTTGGAAGTTCCCGAAATTTATGATGGCGTTGTGGAAATTAAATCGATTGCCCGTGAAGCGGGTCAACGTTCCAAAATTGCCGTGGCTTCGCACAATCCGGATGTGGATCCGGTCGGAGCCTGTGTGGGTCACCGCGGATCCCGCGTGCAAACGGTGGTCAATGAACTGCGCGGTGAAAAAATCGATATCGTCCGTTGGTCTGACCGCATGGATGAATTGATTGCCAATGCGCTCAGCCCGTCCAAAGTGATTTCCGTCACCATTTATGAAGAAGAAAAAATGGCCCGGGTGATTGTTCCCGATCACCAATTGTCTCTGGCGATCGGAAAAGAAGGACAAAACGCACGGCTGGCTGCAAAGCTCACCAATTGGAAGATTGACATCAAAAGCGAATCGGAAGCCGAAGAATTGGAAGAAGAAGCTGAAGATGAAATTTTGGATGAAGTCGAAATTCCGGATGAAGATGCTTTATTCAATACCGATGGCAGTGAAGGGACCGAATAATTTCTTCCAAGGGGAGGGTTTTTGGCATAAGATGAAACGGCGAAAGGTACCGATGCGAAAATGTGTGGCATCACAAGAAATGTTTCCGAAAAAGGAACTGATCCGGATCGTGAGAACGCCGGAAAACGAACTGGTCATCGACCCGACCGGAAAGAAATCCGGGCGCGGAGCGTATTTGTGCGCAAAGATGGAATACATTGATTTGGCACAAAAAAAGAAAGCATTGGATCGCGCCCTCAAAGTGCAAGTTCCAACCGAATTGTATGATCAACTTCGTGATTATGTAAGAGAGCATCAGGGGAATGAATAAGTTTTTGCAACTGCTGGGGCTTGCGATGAGGGCCGGAAAAGTCGTTTCCGGAGAAGAATTGGTGATCCGGGAAATTCGCTCGGGAAAAGCGCAGTTGGTGATTCTGGCCGAAGATGCAAAAAAAAACACGGAAAAAAAGGTCAGTGACAAATGTGATTCTTACAACGTTCCTTTGTTGCGATACGGTGCCCGAAAAGAGTTGGGAAATGCGATTGGAAAAGAGACACGCGTGGTCATTGCAATCACGGACCGGGGGTTTGCCCGGTCATTCATGAAGCTCGGCAGTAAGTAACGGGGGTGAATGGATTTGGCGAAAATGCGTGTATATGAATACGCGAAAAAAGTGAATAAGAGTAGTAAAGAAATATTGCACAGTCTGAAAGACTTAGGCATTCAAGTGAATAACCATATGAGTGTGTTGGATGAAGGAATGATACAAAAATTGGATCAACGTTTAAATCAACAAGAAAAATCTGCAAAAATACAAGATCGTCCGAAAAGCGAAGAAAACAAGAAAGCGACGTCCAACGGAAATCATCGGAATAAACAAGAATCTTCCGCTCACAACAAAAAGCGTGCCTCTTCCGGATCAAACCGCAACAGGGGCGCCCGCGGGCAGGAAGAGCGGAAAGCAAACCATGAGACAAGCCAGCAGGATAAGCGCCAACGCAAAGGAAAGAAAGGAAAGGCCTTCCAGAAAGGAAAAGACGGCGCCCGCCGGGACAATGTGAACCGGAAAGGAAAAGGCCGTCAAAAAGGCGAGAAAAAGAAAGAGGCCAAAGAAAAACAAGCTCCGGTACTGCCAAAGGAAATCACCGTTTCCGGTCCCATGACCGTTGGGGATTTTGCCAAGCTGTTGCGCAGGGAATCTTCCGCTGTCATTAAAAAGCTGATCACGCTTGGAATCATGGCCACCATCAATCAGGAAATTGATGTGGAAACGATGACCATCTTGGCAGAAGAATTTGACGTTACCATCCATGTCAAAGAAGAAGTGGATGAAAGCAACTTTGAAGAGATCGAAGAAACGGACGCACCGGAGGATTTGGTTGAACGCCCGCCGGTGGTTACGATCATGGGACACGTGGACCATGGAAAAACCACGCTGCTTGACAAAATCCGCCAAAGTAAAATCACGGAAGAAGAAGCCGGAGGAATCACACAACACATCGGTGCTTATCAGATCGAAGCCAATGACAAAAAAATCACCTTTTTGGATACGCCGGGGCATGCTGCATTCACCACCATGCGTGCGCGGGGAGCCCAAGTGACGGATATCACGATTTTGGTGGTCGCCGCCGATGACGGCGTCATGCCGCAAACGGTCGAAGCCATCAACCATGCCAAAGCAGCCGATGTCCCGATTATTGTTGCCGTCAACAAGATGGATAAACCCGAGGCAAATCCGGACCGGGTGAAACAACAATTGACCGAATACGGATTGGTTCCGGAAGAATGGGGCGGGGACACCATTTTTGTCCCGGTATCGGCCTTGACAGGAGACGGCATTGACGAATTGCTGGAAATGATTCTGTTGGTGGCCGAAGTTCAGGAACTGAAAGCCAATCCGGATAAACGCGCCCGCGGGGTGGTCATTGAGGCGGAACTGGACAAAGGCCGCGGAGCTGTTGCCACCGTGCTGGTGCAAAACGGTACGCTGCATGTCGGAGATCCGGTGGTGGCCGGAAATTACTTTGGTAAAATTCGTGCCATGATCAACGATCAAGGGCGTCGTGTCAAAACGGCACCGCCATCGACACCGGTCGAAATTCTGGGGCTTTCTGATGTGCCGAATGCCGGCGATGCGTTCATGGTGTTTGAGGATGAGAAAAAAGCGCGTTCCATCGCCGAGAAACGCCAGACGAAACAAAAAGAACAAGAACGCGGAGCCCATTCCCGTGTATCGCTGGATGATTTGTTCAAACAAATTAAAGAAGGCGAAGTCAAAGAATTAAATGTTATTATTAAAGCAGACGTCCATGGTTCTGCAGAAGCCATGAAACACTCGCTTGAAAAAATTGATGTCGAAGGCGCACGCGTAAAAATCATCCATGCGGCTGTCGGAGCCATCACGGAATCGGACATCATTTTGGCTTCTGCCTCAAATGCCATTGTGATCGGGTTTAATGTCCGTCCCGAACCCAATGCCCGGGAGATGGCCAAACAGGAGAAAGTGGACATCCGCTTACACCGGGTCATTTATAAAGCGATTGAGGAAATCGAAGCGGCCATGAAAGGTCTTTTGGATCCCGAATATGAAGAGGTCATGGCAGGCATTGCAGAAGTTCGCCAAATCTTTAAAGTCTCCCGGATCGGAACCATTGCCGGTTGTTATGTCATCGAAGGGAAAGTGGTTCGTGACGGAAAAGCCCGGGTGATTCGTGACGGTGTCGTGATCCATGAAGGTGAAATTGACACGCTGAAACGTTACAAAGATGATGCCAAAGAAGTGGCACAAGGGTATGAATGCGGGATTACCTTGCAAAACTATAATGACATCAAAGAAGGAGACCAAATCGAAGTTTACACCATCCAGGAAGTGGAGCGGAAGTAACGTGCACATCGGTGTACTGGAGTTTAAGGGGCGGGTGATCGCCTCTTTCTCCCTCAAAGACAAACGCAGGGTGATTCAAAGCGTCCAGGCAAAAATCAGGAACCGGTTTAACCTTTCCATTGCAGAAGTGGCGGATCAAGACGATCGTCAGTTTGTTTCGCTTGCAGTCGTGGGGGTCGGAAGCAACCGGATGGTGGTGGAAAAAGAACTGGAACAAGCCCTTCGGCTGCTTGAACAAACGGATGGTTTGGAGATCATTGCGGCAGATATCACATTTTAGGTTGACGAGGAGGTTTGATCCGTGGCACGGATACGTAACAGTCGTGTGGGTGAACAGATCAAAAAGGAATTAAGCCAACTTATCCAGCAAGAGTTAAAAGATCCCCGTATCGGATTTGTGACGGTGACTGGTGTCGAAATGAGCGGAGATCTGCAAATTGCCAAAGTTTACATCAGCGTGATGGGAAGCAAGGAGCAAAAACAAGAAACTTTGACCGGACTGGAAAAAGCCAAAGGTTACCTTCGTTCTGAAATCGGGCATCGTGTTTCGTTGCGTCATGTTCCCGAACTCATTTTTTCGATTGATCAATCCCTTGATTACAGTGAAAACATTGAACGTCTGCTGCGGGATGTTCATGCACGGGAGAATGAGAAACAATGAGCCGTTTTGATGAGCTGCTTGCTTTTTTGGAAAAGGCCGACCGGATTTTGGTGGTATCTCATCTTCACCCGGATGGGGATGCGATCAGTTCCACTTTGGCGATGGGATATATGTTAAAACAATCCGGCAAGCAAGTGACGATGGTCAACGAAGACCCGGTTCCCCGGAAATTTTCTTTTCTGCCCGGAGTGGAAGAGATTCAGCCGGCGGAACAGGTTTCCGGCTCCTTCCAATATGTGATTGCTTTGGATTGCGCCGACAAAGAGCGGATGGGCGCCTGCCGTTTCCTGATCCGGGATGATGCGGTGATCGTCAACCTGGACCATCATGCGACCAATGACCGGTTTGGGGATATCAACATTGTGGTTCCGGAAGCGGCGGCAACCGTGGAAATTATTTTTGATTGGGCGGAAGAAAACAACTTCCCCATTTGCGAACCACTGGCGGCATGCCTTTACACCGGTTTGTTGACCGACACGGGGGGATTCCGCTATTCCAATACTTCCCCGAAAGTGTTGAGACAAGCCGCCAAGTTGGTGGAGACCGGCATCGAAGCGCATAAGATTGCCGACCGGGCTTTGGAAACCGTAACCATGGAGCAGTTAAAATTGCTGCAAGGGGCTTTGTCCACCTTGCAGAAATCAGACGACGGACTCATTGCATGGATGTTTTTAAGCAAAGAGGAAATGGAAAAAGCGGCGAATTCGTACGAAGCTTTGGATGGCATCGTCAATTATGCGCGAAATATTTACGGGGTGGATGTCGGCATTTTGTACCGGGAAGAGGATGACGGAACCATCAAAGTCAGTTTCCGTTCCAGAGAACAGGTTGATGTGGGCCGGGTGGCCAAGGAATTTGGCGGAGGCGGCCATGCCCGTGCGGCGGGATGCAGCATGCCCGGGACACTCCGCGAAGTTCACGAGCGCGTTTTCAAAGCAGTTCAGTCCAGGTTGAAGCAGGGGTGTGAAGTTTAAATGGGGTGCCATGGGATTTTGCCCGTTTTTAAACCAAAAAACTATACATCCCATGATATTGTCGCCATCGTCCGCCGCTTGACAAAGCAAAAAAAGGCTGGTCATACAGGCACGCTGGATCCGGAAGTGGAAGGCGTGCTGCCTGTTTGTCTGGGACAAGCCACCCGGTTGGTGGAATATTTGCAGGACCGCCCCAAGCGTTATCAAGGAACCTTGAAATTGGGGATTGCCACCGAAACGGAAGATCAGACCGGCGCCGTCATTGCAGAGGCCGAACGGGTGGAACCGGTGGAGCCGGCACAAGTGGAGGAGGTTTTCTCCCGGTTTGTCGGGGAGATTACGCAGGTTCCACCCATGTATTCGGCCGTGAAAGTGAACGGACGCCGTTTATATGAGTGGGCCCGCGAAGGAAAGGAGATCGACCGGCCGAAAAGGAAGGTCCACATTTATGAATTAAAATGGACGGGGATGACGCCGGGGGAATATCCGGAAATTCATTTTGAAGTTCTTTGTTCCAAAGGGACTTATATCCGCACCTTGTGTGTGGACATCGGAAAAGCTTTGGGGTATCCGGCGCATATGGCTTCTCTGGTGCGGGTGCAAAGTGGCCCGTTTCACCTGGAAGATTGTTACACGTTGGATGAGTTGAAGCAAATCGGGGAGCAAGATGCTTGGGAAAATCATGTCACCCGGTTGGATGAGGGGATTTCGTTCATGCCCGGCATGATGGTGAATCATGAAGATCTTCAAAAAGTTTATGATGGTTGGAAAATAGAGATTCCTGTTGATGATCAATTGTTCAATGAGAATCAACTGGTTCGTGTGTATACGGACACCGGTGTTTTTTGCGCTGTATACCGGGTGATTGAAAAGGGCTTGGCGAAACCAGAAAAAGTATTTCGGGATGTGGAGTAGATGGAAACGATACATTTAACCTATCCGATGATCGATCAACCAACCGATCAGGAACCGATTGCACTGGCGATTGGTTTTTTTGACGGTGTCCATTTGGGGCATCAAGCCGTGATCCGAAAGGCGATGGATTTGGCCCGGGAACTGAAGGCGGTTCCGGCGGTCATGACTTTTGATCCGCATCCACGGGAAGTGTTGGGGCAAGAGGCGATTCACCGTTATCTGACCCCGTATCCCGACAAGTTGGAGCAGTTTGCCCGTTTGGGGGTCAGGAAAGTTTATGTTGTCCGGTTTGACCGGAATTTCGCTTCTTTGTCAAAAGAAGAATTTGTCACCGACATATTATTGCCGCTTGGTGTCAAAGGCGTGGTGACAGGGTACAATTTTACGTTTGGACACAAAGCGGAGGGGAAAGCAGAAGACCTGACCGAATTGGGAAAAGGGCATTTCGTCACCGAAATCGTCTCTTTGATCCAACAGGATCAGGGAGTGATCAGCAGCACCAGGTTGCGGCGGGCGTTGGCCGAAGGGGATGTCAAAACCGCCCGGGACATCCTGGGGCGCCCGTATTCGCTGCAAGGAGAGGTGGTCCGGGGAGATCAGCGGGGGCGGTTGATGGGGTTTCCCACAGCCAATCTGGACCTGAAGGCTCCGTATTTTGTTCCCGCCCGAGGCGTCTATGTGGTCAAAGCAACCATTGATGAGGTTTCTTCCTATGGCATCATGAATATCGGAGTGCGTCCCACTTTTAAAAGCGATCAACCGAGTGAAAGGTTGGAAGTGCACCTGCTCGGGCAATCCGGCAACTATTACGGCAAGAAATGCAAAGTGGAGTTTTATCATTTCTTGCGCGAAGAGAAAAAGTTTCCGAGCGTGGAGGCGTTAATTCAGCAGATTGAAAAAGACCGGCAAGAAGCAGAGCGTTGGTTGAAGTTATTTGCACAATAAGGCTTGTTGTACTCATCTTTTTCTTATGGTATACTGATTCGGTAACAACCCTGGCTCGGATCACCGATGCCTCGACGGTGTTCTGGGTTATGGGCGATTGAGCAAAAGGAGGATTTTTCGATGGCATTAACCAATGAACGCAAAAGAGAAATCATCAATGAATTTAAAACACACGATAATGACACGGGTTCCCCCGAAGTGCAAATCGCGATTTTGACTCATCGGATCAATGAGTTAAACGAGCACTTAAAAGATCATAAAAAAGATCATCATTCTCGCCGTGGTCTTTTGAAAATGGTTGGTCAACGCCGGAACTTGTTGAACTACTTGAAGAAGAAAGACATTGTTCGTTATCGTGAACTGATCAAAAAACTCGGATTGCGTCGATAAGAAAAGCGGGGCGGCATGCCCCGCTTTTTTATCATCTCATCTTTTAAAAGAGAAGTGGAAGGAACATACTGTAAATGTGGAGAAGTATGCTATATTTGTGTTTTTGAGAGGAGGAAATGATTGAAGATGGAACCTCAAACATTTGAGACAGATCTGGCTGGACGAAAATTAAAGCTGGAAATCGGAAAATATGCCAATCAAGCCAATGGTGCGGTGATGGTGAGATATGGAGACACTTCGGTGCTGGCTACCGCGGTTGCATCCAAGGAGCCGAAAGACACGGACTTTTTTCCGTTGACGGTAAATTATGAAGAACGTTTGTATGCGGTTGGGAAAATCCCGGGAGGCTTTATCAAGCGTGAAGGCCGCCCGAGCGACAAAGCCATTTTGGCTTCCAGGCTCATTGACCGCCCCATTCGCCCGCTGTTTGATGAAGGGTTTCGCAATGAAGTGCAAGTGGTGACCACTGTCATGTCGGTGGATCAGGATTGCTCTTCGGAAATTGCCGCCATGATCGGCGCATCCGTGGCACTTTCCATTTCGGACATTCCTTTCAAAGGACCCATCGGAGGCGTGGTGGTTGGCCGGGTTGATGGTCAATTGGTCATTAATCCGACGGTGGAACAAATGGAAAAATCCGATCTGCATCTGGTGGTGGCTGGTACGAAACACGGGGTGAACATGGTGGAAGCGGGTTCGGATGAAGTGCCGGAAGAAACGATGCTGGAAGCGATTTTGTTTGGCCACGAAGTGATCAAGGAGCTGGTGGCTTTTCAGGAACAGATCGTGGAAAAAGTGGGACTGCCCAAAATGGAGCCCGAATTGCATCAGGTCGATCCCGGACTGGAAAAACGCGTGCGGGAACTTGCTACCGATTCTTTGATCCAAGCCGTTCAGGTCATTGACAAACAAGAGCGCCAGGATGCGATCGATCAGGTGAAGGAAGAAGTCTTGGCACAGATGGCCGGGGAACTGACGGAAGAAGAATGGGCCGAGCAAGAAAAAGACATGAATACCGTCTTGGATCAAATCGTGAAAGAAGAAGTGCGCCGGTTGATATTGGAAGAGGGCAAACGTCCGGATGGACGGGCTGTCGATGAAATCCGCCCGCTTTCCAGCGAAATTGATGTCCTGCCGCGGACACACGGTTCCGGATTGTTCCGACGCGGACAAACCCAGGTGTTAAGCGTTTGTACGTTGGGGGCACTCGGCGATGTGCAAATCCTGGATGGTTTGGATTTGGAGGAATCCAAACGCTTTATGCACCACTATAATTTCCCTCCGTTCAGCGTCGGTGAAGCAAGACCGATCCGGTCACCGGGACGCCGGGAAATCGGACACGGGGCTTTGGGGGAAAGAGCACTCGAACCGGTGATTCCTTCCGAAGATGTTTTCCCGTACACCATCCGGTTGGTCTCCGAAGTGCTTGAGTCCAACGGTTCCACTTCGCAAGCAAGTATTTGCGCGTCGACGCTTGCCTTGATGAATGCGGGGGTTCCGATCAAAGCACCCGTTGCAGGCATCGCGATGGGACTGGTCAAAGAAGAGGATAATGTTGTCGTGTTAACCGATATCCAAGGCATGGAAGACCATCTTGGAGATATGGATTTTAAAGTGGCCGGTACACGAAAAGGAGTTACCGCCCTTCAAATGGATATCAAAATCGAAGACATTGACCGGAACATTCTCCAAAGAGCATTGGAACAAGCGCGGAAAGCCAGAATGGTGATCCTGGACAACATGGAAGCCACCATCAAGGAACCGCGAAAACAATTGTCCCCATATGCTCCGAAGATTACCACCTTACGGATTCATCCGGATAAAATCCGTGATGTGATCGGACCGAGCGGACGAGTCATCAATAAAATCATCGAAGAAACCGGAGTCAAAATCGATATCGAACAAGATGGCCGCATTTTCATCGCTTCTCCGGAAACGGAACAAAACGAACGGGCGAAAAAAATCATCGAAGATTTGGTTCGTGAAGTGGTGGTTGGCGAAACGTATCTCGGAACGGTCAAACGGATTGAAAAATACGGAGCATTTGTGGAAATCATTCCGGGCAAAGAAGGACTTGTTCATATCTCTCAACTGGATTTAAACCGGGTCGGAAAAGTCAGCGACGTGGTGAAAGTCGGAGACTCGATCATGGTAAAAGTGACGGAGATTGATGACCAAGGACGGATTAATTTGTCCCGCAAAGCTGTGTTAAAAGATGAAGCAGCGAAGAAAAAAGAAAAAATTTAAAGGAAGAGGCAAAGGAAAAGAGTCAGAGCGAATCTGCCTCTTTTTTATATTTATACGACGGTCATAGGGAGTTGTCCGGGAGCATATGGTAGGAAGGAAAGGAGGCGGCAAACTTTGTTTCCCGTTCGTATGGCGACACTGTTCTTATCGGTTTGTTTTATTGTTTTGCTGGTCCAACTTCCCACCATTGAAGCTTATGTGACGGCCGTGAAGAGCGGAGCGGAGGTGTCGGTGATCTCTTCCGGCTTCAAGACAGACTCCCCGATTGTCGAAAAGATCAAAAAAGAGGCGCCCAAACATCAGATTGCCCCCATCGATGCACGGCTGGATCCGGTTTGGAAAGCCATTCCGGGGCTGAACGGGCGAAAAGTCGACCTGGAAGCAACGATCGAAAGCACACTCAAGCAAAAAGACAAGCACCGGATTCATTGGGTGTATCAGGAAATTCCCCCGAAAATACAACTGGAAGATCTGGGACAGGCACCGGTTTACCGGGGGAACGAAAAAAAACCGGCGGCCGCGCTGATGGTGAATGTGGCTTGGGGAACGGAATACATACCGGAAATGCTCAAGATTTTCAAACAGGAAAAGGTGAAAGCGACCTTTTTTTTGGATGGTTCCTGGCTGAAAAAGAATCCCGAAATGGCCAGGCAGTTGGTGAAAGAAGGGCATGAAGTGGGAAATCATGCTTATTCCCATCCTTTGATGAGCCGGATCGGCACCGGGCAAATCGAAGCCGAGATCGGAAAGACGGAGTCTTTAATCCAAGAAACCTTACAGGTCAAAAGCAGGTGGTTTGCCCCGCCGGCCGGCGATTTTGACAACCGCGTATTAAAAATAGCCGAACAATTTCAAATGAAAACCATATTATGGACATTGGATACCGTCGATTGGAAAAAGTCCGTTTCGCCGGAGATGATGGTGAACAAGGTGGAAAAAGGGATTGCACCCGGCACCTTGTTGTTGACTCATCCGACGGACCGGACGGTCAAAGCGTTGCCGGGGATGATCCGTGCAGGGAAAAACAAGGGCTTGAAGTGGATGACGGTCAGCGAAATGCTCTCTTCAAAACGAGTGGATTCCATTGAGCAATGATTTATGATTTGATATAGTAGTGTTGCTTTTTTGTAGTTTATCAGAGAATTAAGGAGGATCTTATTTGATCGTTAAACATACGCTGGAGAATGGTGTTCGGTTGGTTGCTGAAAAAATCCCCCACGTCCGTTCAGTCGCTTTGGGGATTTGGGTGGGAACCGGTTCTGAAAATGAATCGCTTGCCAATAACGGGATTTCCCATTTTATCGAACACATGATGTTTAAAGGAACCAAAACGCGCACCGCGAAACAGGTTGCGGAATCCTTTGACGCAATCGGCGGACACGTGAATGCCTTCACCTCCAAAGAAATCACTTGTTATTATGCCAAAGTGCTGGACGAACATTTTTTAACGGCACTGGATGTATTATCGGATATGTTTTTTGAATCGGTGTTTGATGAAGGCGAGATCGAAAAAGAGAAAAAAGTGGTCTTGGAGGAAATTTACATGGTGGAAGACACGCCGGATGACCTGGTTCATGACATGCTTTCCGAAGTGTCGTCAGGCGGCCACCCCTTGGGATACCCTGTATTGGGCAATGCCGAAACCGTGACCGGTTTCCGCCGGGAGGATTTGTTCCGCTATAAAAATCAATTTTATGTCCCTTCCAATGTCGTGATTGCCATTGCCGGCAATTTGCCGGATGATTACAAGGAAAGGGTCGAGGAAGCTTTTTCCGGCCATCAGGGCGAGATGCCGGTACGAAGCAGGGAGGTTCCGGTTTTTACGCCGGATGTCAAAGTGAAACAAAAAGAAACCGAACAAACCCATTTATGTTTCGGATTGCCGGGACTGGCCGTCGGTGATGAAGATTTTTACACATTGGTTTTGTTGAATAATGTGCTTGGCGGCAACATGAGCTCCCGTTTGTTCCAGGAAATCCGCGAAGAGCGCGGGCTTGCCTATTCGGTGTTTTCTTATCATTCCGCCCATCGTGACACCGGCCTTTTTGCCATCTATGCCGGAACGAAACACGGGCAGGAGAATGAAGTGGTTGAATACATTTATCAAATTTTGGATGATCTCAAAACCAAAGGATTGACGACGGACGAATTGCACAAAGCCAAAGAACAATTGAAAGGGAGCTTAATGCTTGGACTGGAAAGCACCAACAACCGCATGAGCCGGTTGGGGCGCAATGAATTGTTGTTAAATCGTCAGTTCACTTTGGATGAAATCATCGAAAAGGTGGATAGAATCACTTTGGATGGCGTGAATCGTCTGGCGCGGAAAATTTTTTCATCACCCATGTCGTTGGCGCTGATTTCGCCAGACGGAAAAATCCCTTCCAGTTTTAGGAGGAACTCCATTGCATAATGTCAAAGTTTTTCACATGCCAGGAAACGAAGATCTGCCTCTTCCAGTGAAAATGTCGACGGGTTCCAGTGGTTTTGACCTCCATGCCGCAGTGATGGATCCAGTGGAAATCAAACCGGGCCGGTGGAAGTTGATTCCTTCCGGAATTGCCTTGTCGATGCCGGCAGGGCTGGAGGCCCAAGTGCGTCCGAGAAGCGGTCTTGCTTTAAAACACGGAATCACCGTGCTCAACACACCAGGGACAATCGATGCAGATTACCGTGGAGAAATCGGGGTGATTTTGCTGAACCTGGGGGAACGGCCTTTTCGGGTTGAGCGGGGAGACCGTATTGCACAGTTGGTGTTTATGCAAGTCCCTGCGGTTTCCCTCAAAGTTACGGATTCGTTGGATGAAACGGAACGTGGGTCTGGAGGCTTTGGTCATACGGGTAAATAGGAGAGGTGATCTTCATGTCCGAGCGGCATCGGATCAGAAGGTTACAGGAGGAAATGGAACATCTCCGCAAGGAATTGTACCAATTGGTGAACGGTGAACCCGAACGATTGATGGATGCCCGTGTTTTGCCTTTATCCGAGCAATTGGATGTACTGATTTTGGAAATGCAAAGGATCCGATTGGAACATCGTTAAATAAAGAGGATGAACGCCAAATCCGAAAGTCCGGCAAAGCCTGCCGGGCTTTTTGTTTTTTATAAAAGTGTTGACTGAATCATATGGATAAAGGGAGAGAAAAAATGGAACGTCGGGGGGGAAGGATGTTGCGCTGGAGTGAACTGGCAGAAAAGGAATGCATCGATTTGGTGGGAGGAGAACGGTTGGGGGATTTGTTGCATGCAGACATGTCGTTTGATCCGCAAACCGGAAAAATCCATGCCATCTTGTTACCGACGGAATCTTCCTGGTTTAAAAAGAAGCACAGGTTTATGGAATTGACGTGGAGCATGATCCGGAAAGTCGGGCCGGAAATGTTGATCGTGGATTCGTTGGACCGGAAATCTTCCAAAAAACAATTTTGACAGAAGGGAACCGAATTTCATTTTTTTTCATATGATGCAAACAGATGTTGATCATGGTAAGGATTCTTCCGGGGAAAGGAGTGAAGAAAATGCTGACAGGCAAACATGTTGTCTTCCTCGGTGGAGATGCACGGCAATTAGAGGTGATTAAAAGCTGTACAGAGATGGGAGCCCGGGTTTCATTGGTCGGATTTGACAATTTGCAAAGTCCGGTCAGCGGGGCTTCGTTAAAGGAGCTTTCGTCTGATTTGTTAGAGACGGCGGATGTTTTGGTGCTCCCGATCATTGGGACCGATGAAAAAGGGCAAATCAGCAGTGTTTTTACGTCCAAAAAATTGTTTCTGACCCCGGAGTTGTTTCAATCTCTTCCAAAACATTGCCTCGTTTTTGCCGGGATGGCCAAGGAGTATTTGCGAAAGTTGTGCCGGGATTTCGGGCTTCAACTTATTGAACTCTTAAACCGGGATGATGTGGCCATTTACAACTCCATCCCGACAGTGGAAGGTGCGCTGATGATGGCCATCCAACACACCGACATCACCATTCACGGTTCCGAAAGCATTGTGCTGGGCATGGGAAGATGCGGATTGTCGCTGGCCCGTGTGCTTCATGCGATTGGAGCCCGGGTGAAAGTCGGGGTGAGAAGTTCCGAGCATAAAGCCCGCGCATTTGAAATGGGGATTCAGGCGTTTGATACATCCGAATTAAAAGACCATGTGAAAAATGCGGATTTGATTTTTAACACCGTTCCGTCCTTGATCATTGATTCAACCGTTTTGGCCAACGTGCCTCATGATGTGGTCATCATTGATCTGGCATCCAAACCGGGAGGCGTGGATTATCCGTTCGCTGAAAAAAGAGGAATCAAAGCCATCTTGGCACCCAGTCTGCCGGGAATCGTGGCTCCGAAAACAGCCGGACAAATTTTGGCGGAGACCATCACCCGGATCATGAAAAATGAGTTATTCAGGGAGGGAACCGTTCAGTGAATTTTCAAAACTTGACGATCGGCTTCGGCATGACGGCATCCCACTGTACGCATGATGAAGTGCTCCCGCAAATGAAAAGATTGGTGGAACTGGGGGCAAACGTGATTCCGGTGATTTCCCATACGGTGGCGACGGTGGACAGCCGGTTCGGCACCGCGGAAGATTGGAAGAAAAAAATTAAAGAAATCACCGGTCGGACCCCGTTGATGACCATACCGGAAGTGGAACCGTTCGGTCCTGAAAAAAGTTTGGATTGTTATGTGATTGCTCCCTGCACCGGCAATTCCCTGGCGAAACTTGCCAATGCATTGACAGACAGCCCGGTTTTGATGGCGGCAAAAGCGCAAATGCGCAATCACCGGCCGGTGGTGGTGGCCATTTCCACGAACGACGCACTCGGCTTAAATGCCGTCAACCTGGCAAAATTGCTGGCAGCCAAAGACATTTATTTTGTTCCGTTTGGCCAAGATGCTCCCGAAAAAAAACCCAAGTCCATGGTCGCACGAATGGAATTGATCCCGGAAACCTGTCTTGCCGCCATCGAAGGGAAACAGTTGCAACCATTAATAGTTGAAAAATACCGCGACTTGACTTCATAATAGAAAAGAGCTCGGATAGATGATTCATTCGTCCCCCGTTGATGAGATACAGGGGGCTCACTTCATATGGGAGGGGACTTCATGAGTGCAAAGAAATATCATGTCGCAGTGGTAGGAGCAACCGGAGCTGTCGGGCAAGAAATATTGAACACCCTCGATGCAAGAAATTTTCCTGTCGAAGAATTGCGCTTGCTTTCTTCCAAACGTTCCGCAGGCAAAAAAATCACATTCCGCGGTCGGGAAATCACGGTTCAGGAAGCAGTGCCGGAAGCATTTGAAGGCGTCGATTTCGCTTTATTCAGCGCCGGCGGCAGTGTGAGCAAGGCATTGGCAAAAGAAGCGGTGAAACGCGGGGCGGTTGTTGTGGATAATACCAACGCCTTTCGAATGGATCCGGAAGTCCCGTTGGTGGTTCCGGAAGTGAATCCGGAAGCGTTGGATCAGCATAAAGGAATTATTGCTAATCCCAACTGTTCCACCATTCAAATGGTTGTTGCATTAAAACCGTTGGCGGATCGGTACGGAATCGAAAGAATCATTGTCTCGACATACCAAGCCGTCTCCGGTGCGGGATGGAAAGCGATCAACGAATTGGAAGAACAGACGAAAGCCGCGATGGCCGGTGAAGAAGTCCAAAAAAATGTTCTTCCCGTGGGCAAACTTGACAAACATTATCAGCAAGCGTTCAATGTGATTCCGCAAGTGGATGTGGCGGAAGAAAACGGATTTACACTTGAAGAAATGAAAATGGTGCGCGAAACGAAAAAAATCTTCGCGGATGATTCCATCGGCGTGACCGCCACTTGTGTACGGGTCCCGGTTGTACGGGGGCACAGTGAATCGGTTTATGTGGAATTAAAATCGGATTATGATCTTGATGAAGTCCGAACCCTCTTAAATGATGCACCGGGTGTGGTGGTGCAGGATGCGATTGAGGAGCAGATTTATCCCATGCCGTTGGATGCATCCGGCCGGACGGAAGTGTTTGTCGGACGTCTCCGTCGCGATTTGACCCATCCGCGCGCGCTCAATATGTGGGTGGTTTCGGATAATCTTTTGAAAGGAGCCGCAACCAACACGGTTCAAATTGCAGAAACTTTGATTGAAAGAGAATGATCATAAAGAGAAGGGATAGGCATGAGCATTATTGTACAGAAGTTCGGAGGAACCTCCGTGGCAACCGCTGAACGGCGTCAACGTGTCCTTCGTCATATTTCCAACGAGCTGGAGAAAGGAAACAAACTGGTTGTCGTTGTTTCAGCGATGGGAAGAAAAGGGGAACCATACGCAACCGATACATTGCTCGGTTTGATTGATCAATATGATCAGCAGATCCTTCCCCGTGAAAAAGATTTGTTGTTAAGCTGTGGAGAGATCATCTCTGCAGCTACCCTGTCCAGCATGCTGACCCAGGCAGGGATCCGGAGCTGTGTATTGACCGGTGGACAAGCGGGGATCATTACGAACGATCAGCATACCAATGCCCAGATTATCACCGTCAATCCCAAACGCATTTTGCATGAACTGGAAAACAACCGGGTCGTTGTCGTGGCCGGTTTCCAAGGGCAAACAACCGATGGGGAAATCACCACATTGGGACGCGGCGGCAGCGATACCACCGCAACGGCGTTGGGAGCGGCTCTTGATGCCGAATATGTAGATATTTTTACAGATGTAGAAGGGATTATGACGGCTGATCCAAGAATTGTCGAGGATGCCGCCCAATTGGAGCAGGTCACCTATGTTGAAATGTGCAATCTCGCTTTTCAAGGAGCCAAGGTGATTCATCCCCGGGCCGTGGAGATTGCCATGCAAAACAGCACCCCGATTCGGATCCGTTCGACCATGTCAGATCATCCGGGAACGTTGGTGACACACCGGAGCGAAATTGACCGGTTGGTTCGGGAAGTGCGGGAAAACACCATCATCGGGGTGACCCAGATGCCCAATGTGACTCAGGTGAAAATCCGGCTGAGACAAGGGCAATACCATACACAGTTGAAAGTTTTCAAAGCGATGGCGGATCATGGGATCAGTGTCGATTTTATCAGCGTCAACCCGAGCGAAATTGCCTATACGGTTTATGATGATCTGGCGGATCAGGCGGAACAAATATTATATGATCTGGGACTGGAACCGGAATTATCCAGAGGTTGCAGCAAGGTATCCGTTGTCGGTGCAGGCATCGCCGGCGTTCCGGGCGTGATGGCCAAAATTTCGGAAGCACTGACGGAAGAGGATATTCAAGTGTTGCAATCAGCGGATTCCCATACGACCATTTGGGTGCTGGTCCATGGAAAAGACATGGAGAAAGCTGTGCGTGCTTTGCATAAAAAATTTCATCTGAATAAACTCTACTGTTCATAAAGGAGGGAAGTGAGGATGTTGTTTGGCCGATTAATCACGGCGCTGTTTACGCCGTTTACCGAAAAAAACGAGATTGATTGGCCCTGTTTGGAAGCCAATATCGAGCGTTTGATCGAGGACGGGAATGATTCAATTGTTGTTTCCGGAACGACCGGAGAGTCTCCCACCCTGACCCTCTCCGAAAAGCTGGAATTATATCGTTTTGCCAAAGAAAAAGCAAACGGCCGGATCAAAGTGATCGCAGGGACCGGAAGCAATAACACCCTGCAAAGCCTGGAATTGACCAAGAAAGCGGAGGAAATCGGCGTCGACGGGGTGATGCTGGTCGCCCCCTATTACAACAAACCGTCGCAGGAAGGGTTGTATCAGCATTTCAAAACGATTGCCGAATCGACTTCACTGCCGGTGATGCTGTACAATGTCCCCGGACGCACCAGTGTCAATATGACGGCTGAAACCATGGCCCGGCTCGCTCAATTGGATAATGTGGTCGCTATCAAAGAGGCAAGCGGGGATCTCAATCAAATCGGGCGGCTGATTTCGGCGGTTCCCGGCGATGTGGCGGTATACAGCGGAGATGACGGGCTCACTTTGCCGATTTTGTCAATTGGCGGAGCCGGGGTTGTCAGTGTGGCCAGCCACCTGGTCAGCCGGGAAATGAAAGAAATGATGGAAGCTTTCTTCTCGGGGGATGTGAAGAAAGCGCAAAGCTTGCACATTCGTCTGCTTCCTGTCTTCCACGGCGTGTTTTTGACGACGAGCCCGGCACCATTGAAATATGCGATGTCGCAAAAAGGCTGGTCGAAACCTTACGTGCGCCTTCCGATTGTGGAATTGTCGGAAGAAGAAAAAAGGAAAACGGATGAATGGTTGAAAGAACTGTGACGGGAACGGACCTGCCTGTGAAAAAACAGGCAGGTTTTTTGTTTGTCATTGACTTTTTTCAACAAAAGATCCGGTTGCATATCCATTTTTTCATCGTATATAATAAGGGACAAGTGGTAATTGTGCGGTTTCAGTTAAAGACAACAGAATATAGGAGGTAAACAGTTTGACAAAAAACCAGCGAGGCAAATTATCCATTTTTGCACTCGGTGGGTTGGATGAAATCGGAAAAAACATGTATGTGGTGCAGTATGAGAACGACATTGTTGTGATTGATGCGGGCTTGATGTTCCCGGAAGAGGAAATGCTGGGAATCGATATCGTGATACCTGACATCACATACTTGGTTGAGAATAAGGATAAAGTACGCGGGATTCTTTTGACGCATGGTCATGAGGACCATATCGGCGGGTTGCCGTATATTTTGCGTCAGCTCAATGTTCCCGTCTACGCCACGAAATTGACGATGGGACTGGTTGAGCATAAATTGCGCGAGGCTCATCTGTTAAATGACACGAGACGGTACATCATCAACGGTAAATCGGAAATCAAACTGGGTTCTACACTGAAAGCGACCTTTTTTTATACAAATCACAGTATTCCTGATTCCGTCGGTATTTGCATTCATACACCGGAAGGAGCCGTTGTTCACACGGGAGATTTTAAATTTGACATGACCCCGGTGAATGGACACGCTGCGGATATCCATAAAATGGCTGAGATTGGAAGAAAAGGGGTATTGTGTTTATTATCTGACAGCACCAATGCGGAGCGCAGCGGATTTACCGGATCGGAAAGTTCGGTCGGTGAAGGGATTAACGAAGTTTTCCGTAAAGCAAAGCAACGGGTGATTGTAGCTACTTTTGCGTCCAATATTCACCGCATCCAGCAAGTGGTGAACGCATGCCATATGTACGGTCGAAAGCTGGCTGTGGTGGGACGCAGCATGGTCAATGTGGTCAATATTGCATCGGAGTTGGGGTATTTGCATGTGCCCTCTGATTTGATCATTGACCCGGATGAGATCAATCGTTTGCCTGCCAATCGGGTGGCGGTGATTTCCACAGGAAGTCAGGGGGAATCGATGTCAGCGCTGACACGCATGGCGAACGCATCTCATCGCAAAGTGGAAATTTTACCCGGTGATACCGTGATCATTGCCGCCACACCCATTCCGGGCAACGAAAAATTGATCGGCCGCACCGTCGATCAATTATTCCGGATTGGGGCGGATGTTGTGTATAGCACGACTTCGCATGTACACGTCTCGGGGCATGGGTCTCAGGAAGACCTGAAGCTCATGCTGAATTTAATGAAACCCAAATACTTTATTCCGATTCATGGTGAGTATCGTATGTTGCGTGCTCACGCCCAACTTGCCGAATCTGTAGGAGTAAGCCCGGAAAACATTTTTATCTGTGACAACGGAGATATGGTTGAAATTTCAGGTGGTCGTGCCCGATACGGGCCCAAGGTTCAGACCGGGAAAGTATTGGTTGACGGTTTGGGCGTTGGTGATGTTGGCAATATTGTATTAAGAGACCGCAAATTACTATCTCAGGACGGAATTTTGGTCGTGGTGCTGACCTTAAGCAAAGAAAACGGCACCATCGTATCAGGGCCGGATATCATTTCGCGAGGCTTTGTTTATGTTCGGGAATCCGAACAGTTATTGGATGAAGCGAACCGGATTGTGGCATTGACCATGGAAAAATGCATGAGAGAACATGTCAGCGAATGGGCTTCGCTCAAAACGAGCATCCGGGATGCATTAAGCAAATTTCTGTTTGAGCGGACCCGTCGTCGTCCGATGATTTTGCCAATTATCATGGAAGTTTAACCGGAGCTAAACCGCACAGAAAACCGCAGCTTTTGAATTTTCAAGGCTGCGGTTTTTTTATCATGTGAAAGAGAAACCGTCTGGTCGAAGTTCATTTATTGTGGATGGGGCGGCGAGCCTTTTTGAAAAATAAACGGGTTGAAAGAAGGGGACGAATGAAATTTGTTTTGATATTCGGGCCGCAGGCCGTCGGCAAAATGACAGTCGGTCAAGAGCTGGAAAAAATCACAAAACTGAAGCTTTTTCATAATCATATGACACTGGAGTTGCTTTATCCGTTTTTTGGCTTTGGTCCTGAAACATGGAGATTATCGGACCGGTTTCGCCAAGAGATCTTTGAAGCGGCGTTGGAAAGTGATTTATACGGAATCATTTTCACGTATGTCTGGGCGTTTGATCAACAAAGAGACTGGGATTATGTTGATCACATTTGTTCCATTTTCGAATCCAAGGGCGGGATTGTTTATTTTGTGGAACTGGAAGCGGATCTTGAAGTAAGGCTTGAACGGAAGAAAACGCCCCACAGACTTGAACAGAAACCGTCGAAAAAAAATATAACCCAATCGGAACTTGATTTAAAACAGACTGCTGAAAAACATCGTCTGAACTCTCTGAAAGGAGAGATCAGAAAGAAAAACTACATTAGAATAAACAATCCAAATTTGGAAGCTGAAAAAGTCGCCAAAATGATTAAAGACAAGTTTCGGTTATGAGCATGATGCAGCTTTCTTTTCAAAGTATATTTTTATCGGGAGATGATCCCAAAAAGCATCCCGGTTTTCTTATGCAAAAACCGGGATGCTGAAGCCGGTTATTAGCCTATTATTCTTTGATATGGATAAAATTGTCAATCCGGTCGACCGGAACTTGGCGTGTTCCGTAGCCGTGACGGATGTCCCAGTTATACTCTTCGACCGTTACCGTTTTCTGGTCGCTGCTCACTTCGGCGACAAAGGCCACATGGTTGAGGTTTCCTTTGTTCCATTGGGCAACGGCTCCGACTTTTGGAGTACGGGTCACTTTGTAACCGATCTGTCTTGCGTTTTCATCCCAATGCCTGCCGTCTCCCCAATGTCCTTTTTTCCCGTTGGGACCGGTCATGTCATTGGTGAACGGAATCCCGAGATTGTTATTGATGCGCCAAGCGACAAAGGAGACGCATTCCCTGTAATAAAAACCCCATGGGTCCACATCGCCGGGATTGTTGTTCTTAAAAGGGTAATCATCGATCATTTCGTGCTTGGGTTCCGGTTCCGTGTCGGGATCGGGTTCCGGTTCCGGGGAAGGTGCGGGTTCTTTGTCTGAATTTCCTTTTTTGAATTGGAAGCTGATATTGACAGATCCTGAACCGGAGGCGATCAGTTCAACGTGCCAGTCTTTGTCGTCTTCCGGGGATTGTGGCGGGTTGGGCGGTTCTTCCGGCGGGGGGTTGTCCGATCCTTTTTTGCTTTTCAGTGCTTTGAGCGATTTGGCTCTATCCATATCCACGTCCCCCATGATTCCCGAAACACTCCCTTTGTCCGTATACTGCCAGCAGGTGATTTCTTCCCAGCCGCCTGTTGAGGGCAACTTTGGATTGTCCACTCTCCAATGCGCCAACCATAACGGCATGTCCTTCAAACGGTTGTTAAAGCCGTTAAACCGGTTGATATAATCGGGGAAAGTATATAACAGAACTTGTTCGCCCGTTTCTTTTTTGACATGCTCAGCGAAGGCAAGCACCCAATCGGCGATTTCGTCCGCTGTCATATCGGTGTTTTTTTCTTCCAGGTCGAGAACAGGCATGAGATCCAGTTGGTGTTGTTTCAGTTTATCAACAAAGTTCTGCGCTTCTTTCTCTGCTTTTCCTCTTAACAACTTGGCGTAATGATAAGCACCCGCTTGGATTCCTGCGGCCTGCGCCCCTTTTACATTATTGGAGAATTGTGAATCGGTGAATGTTTCTCCCTCGGAGGCTTTGATGAAAGCGAATTGAATGTTTTGAGGATCTTCTTTGACTTTCTGCCAATCAATGACCGATTGATGATGGGAAACATCAATCCCGGTGACACTGCCAAATTGATCAGACATGCCATCCACCCCTTAAAGATGATGATCAAAGTCCCCCGGGTTCCGGTTGAAAAACGGTTTTGGATGTGGCGGTTGAACCCCCCTTTTTTTCATATTCCACATTTAATTATACCAAAAATAGAGAAAATTCTATATATTTTTATATTCGTTCGGCGCCGGAATATGGGGAATTTTGTTTGGAAGTGCCGGCCGATGCGATCTTTAATCATTAAAAATTGGTGAACTTATTCAAACGGATTATGTAAAAGGGATGAGATTTGATATAATATCCATCAATTCTTGTGATTGATGTTCGATTGGGAGGGTAAAAATTGCTGCAAGAACCAGATTTGATGAACTCACTCGTCATACCAGTGGCTGTTGAATTGTTGATTTTCATTATCAAGGCATTGGTGGTGACACTGTTTGCCGGTTTCTTTGTAAAACGATACCTGGCACCGCTGGTAAGAAAGGATCAAAAACCGGCTGAGCCGCGTGTTCCGGCCGGTGAAGACAAGGAAAGCAATGAAGAAGAAGTGTATGACAAAAAAGAAAGCTGATTAACGGAGTGAATAAAAAACCGTATCCTGTTCAAGGATAACGGTTTTTTTATTTTGCGCGGCTAAGAAAAAACGGCTTTTTGCTATGAACCGGCGGATGTCACTTTCAAGCCGACGATTCCGGTCAGAATCAGGAACAAAAAAAATAAACGCATCCAGTCATGGGGTTCATCAAACAAAAAGATCCCCATCACTGCCGTGCCCAAAGCGCCGATCCCTGTCCAGACCGCATAGGCGGTGCCGATGGGCAGAGCTTTGAGGGCGAGGGACAAAAAATAAAAACTGAAGATCATTCCCGCAATGGTGACAAGGGATGGAAACCACCGGGTGAATCCTTGAGTGTATTTAAGTCCGATGGCCCATACCACTTCAAAGATTCCGGCGATGATCAAATAAGCCCATGCCATGGAATCTCCTCCTCCCGTCAAAACGGAATGTCTGGATTCATACTCAATTAGTATAACAATTTTCAAATCAAAAGCAATCTCTTTCGATATCCGGAGTGCAATGAGAAATTTGTATGAATCAGGAAGGAGAATTCAAAGTGATGGAAAAGAATTAGTATGATATTTCATAATCCCCAAATGCTGTATATTTAAAAATATAAGTATACCATTTTTTAAAGGATACATACCTCGGTATTATAAGTGAAATACCTGCATCTTATCCTAAAAAAACATTTTTTATTAATATAATTTTATATTATATTTTGTAATTTCATGATTGTCTCATTGACAAGAGGTGGATGTTTGGATAAAATGAGTTTTGCCTTTTTAAAATAATTGAAATAGATTATAAGTTTTATATAAATTGGAGGTTAATCATGACAAAGGCAAATGTTAAGGAACGATGGAGTGAGCACAGGAAGTATTATGATGAATGGTTAAGCGGCTATGACGGGGGCGTGTTCATTCCCGCCAGGAAAACATGGGCCGGCAAAGAAAACATGGGGGACAAGACCGAAGCAACGGAGCATTATGAAATCAGACTGAGCCGGTCCGTCACGGAAAGTTTTTATGCGTTATCCAGAAGGTATCAAGGGGCTTTGTCTTCGTTGCTTCAAGGGGTATGGGGCAGTTTGTTGTTGAGATACAGCGGAGAGAAAGAAGTTTTGTTTGCATTTGCCGACCATTCCGGAAAGAAGGTTTCTGCTTGCGATCCGGAAATCCGATTGCTCCGTGTCAAAGATGATTTTCAAACCTTTCACCAGCTCTTTCAACATTTATCTGAAACGAATGTCCATTCTGATTTTTATTTATTTCCGCTTTCCAAGTTTCATGAAGCGACGGGGATGGAAGAAAAAGCCTTGGACCATCTGGTGGTGGTCGCTGACCGTTCCTATTCCAAGCACGATTTTGAATACCAAAAATGGATCAAAAAATATAAAAGCCACGTTTATGAAATCAAAAAGCATTCGCCCTGGCTTGGATTGATTGTCACCTTTATTCCTGCCAAATACCTGACCATCCGGATATCATATGATCCGAACCGGTATGATGGCGGGTTGGTCCAAGCGACCGGTCATCATCTGAAACAAATCATTGAAAGTGTCACCAGCCAGCCCCGTTTGGAAATTTCCAAGCTGCAAATTGTAACGGCACAAGAAAAGAAATTGTTGAAAAAGTTTAATGAAACAACCCGGGCGTTTCCGGACGGCCAAACGGTACACTGGCTGATTGAGCAGAGGGTCATGAAGTCGCCGAACCGGACCGCCGTTGAATTCAAAGGGGTCAGCCTGACTTATCAGGAGTTGAACAGGCGTGCGGATCTGTTGGCGATGGCATTAAGGGATCACGGGATTGAAAAAGGGGATATTGTGGCCATCATGGTCCCGCCTTCTTTGGAAATGATCGTGGGGGTTTTGGGGATTTTGAAAACCGGAGCCGCTTATTTGCCGGTGGACCCCAATTATCCCCCGGAGCGGGTTTCGTATATGTTAAGCGACAGCCATGCCAAAATGTTACTGTCTTTGAAAACGGCGTCCGGCGATTTGGCGGCGGATGTCCCGGTATTTGATTTGGCGGGAATGGATTGGAGTGTAACCAGGGAAGTGCGCCCCTTGCCATCGACGGTCCGGCCCGATGATTTGGCTTATGTGATTTATACATCGGGTTCCACTGGAAAACCCAAAGGTGTGATGATCGAACATTCTTCCTTGGTTAACTTGGTTTATTGGCATAATCAACAATTTCACATCACCGAAAAAGACCGGTGCACCAAATATGCCGGTTTTGGATTTGATGCTTCCGTGTGGGAGATTTTCCCGCCGTTGGTGGCCGGGGCGACGTTATATGTCATTGAAGAGGAATTGCGTTACGACATCCGGCAGCTCAATGATTTTATGCAGGAAAAAGGCGTAACGGTAAGTTTTCTGCCGACGCAGGTGGCAGAGCAATTTATGGAGCTCAAAAACCATTGCTTAAAAACCCTGCTGGTCGGTGGAGACCGGCTGCAAAAAGTGGTTCCGCAAACGTATCAAATCGTGAATAACTACGGGCCGACGGAAAACACCGTTGTCACCACCAGCGGGACCGTCCGTCACGGCGAACCGGTCATGATTGGAAAACCCATTGCCAATCACCGTGTTTATGTCTTGGATCAAAATCACGAGTTGCAACCGATTGGCGTCCCCGGGGAGTTATGCATCAGCGGGGCCGGTTTGGCACGCGGTTATTTGAACCGGGACGACCTGACATGGGAAAAGTTTGTCCCCAATCCCCATGAACCTGGTGAAATCATGTACAAAACCGGCGATTTGGTCCGTTGGCTTCCGGACGGGAGGCTCGAGTTTTTGGGGCGGTTGGATGATCAGGTGAAGATCCGTGGATTCAGGATTGAGCTGGGGGAAATCACACAACGCCTCATGGAATATCCTTTGATTCAAGATGCATTGGTCTTGGTGCAAAAGGATGAATACGGCGAAAATCGGCTGGTCGGCTATTTCACGGCGCGGGAAGGATGGGAGGAAGAAGACATCCGCAAACACTTGGCGCAGGAATTGCCCGACTTCATGATTCCATCCCATCTGATTCATTTGGAAGCTTTTCCGCTGACTCCCAACGGAAAAGTGGATAAAACAGCCTTGCCGTTGCCGGAAGCGGAGGTCGCCGCCACCCGGGAGATGGTGAAAACCACCGATGAGACTGAACAAACGTTGATCCGGCTTTGGAGAGAGATTTTGCACGTGGAGCCCGGAACCGGGGACCATTTCTTCCATTTGGGAGGGGATTCCCTCAAGTTAATGCGCCTGGTGTCAAGAATCCGGGAAGAGTTTCAGATTCAGCTTTCGATTCAGGCTTTGTTTGAAAATCCGGTTTTACGGGAAATGGCCGTTCAAATCCGGAAAGCGGAAGGGAGAATCCCGGATGAATGGGAGGGGGCAGAGGAAAAAGAGTATTATCCGGTGTCTTCCGTCCAAAAACGCTTGTATGCAGTGGAACAGATGGAAAACATCGGCACCACTTACCACACACCGGTGCTGTTGGAATGGAAGGGAAATGTTGACACAAAGCGGTTAAAATCGGCGGTGGAACAATTGGTTCAGCGGCATGAGGCGTTGCGGACTTCTTTTCACTGGGTGGATGAAGAACTGGTGCAGAAAGTTCATGCCGAAGCAGAGATAACGTGGGAGATGTTCCACGCGGATTCGGAGGAAGAAGTCAAACGGATTGCCGCAGATTTTATCAGGCCGTTCGACTTGGGACAAGCTCCTTTGCTTCGTGCTGCCCTGATTCATGCCAAAGAGAGCTGTTACCTGTTGTTGGATATGCACCACATCGTTTCGGACGGAATCTCGGTGGAAGAAATCTTGTACCGTGAACTGGGTGCGCTGTATCAAGGACAGGAGCTTCCGCCGCTTTCCAAACAATACAAGGATTATGCCTTGTGGCAACAACAATGGCTAGGTTCGGAAGAGTGCCTTCGCCGCAAAGAGTATTGGCTCAAGCAGCTGGAAGGAGAGATTCCGGTCCTTGAATTGCCGGCCGATTATCCGAGGCCCCCGGTTCAGGAGTTTGCCGGAGATAAAGTTTCTTTTGAAGTGAAACCGGCATTGGTGGAACGCCTGAAGCAAATCGGGCAAAGAGAAAATGCCACCTTGTTTATGATGCTGTTTTCGGCATACAAGGTGTTGATTTCCAAACTGTCAGGACAAGAAGACATCATTGTCGGAATCCCGGTGGCCGGACGTGCAAAATCCTGGATGGAACCGGTTTTCGGAATGTTTGTCAATACCTTGCCGCTTCGTTCACAGCCCAAAAAGGAACTTTCTTTTGCCGGTTACATGCAACAAGTCAGAACCCGGGTGCTGGGAGCTTATGAAAACGGCGATTATCCGTTGGAAGAAATCATCGGGGCGTTGCAATTGGAGCGGGACCGGAGCCGCAATCCGCTCTTTGACACCGCGTTTGTTTTGCAAAACATGGAGGAAGCCGCCGTCCGGATTCCCGGAGCGGACGTGAAAAGTGTGCCGTTGCCCTGGAAACAATCGATGTTTGACATGACATGGGAAGCAAAGGAAGAAGCTGACACCGTCCGCTTTCATGTGGAGTATTGCACGCGTCTTTTTGAACGGAAAACGATTCAACGATATATCCGATCCTTTTTATACATTTTGGAACAAATCGCCAAGGATCCGGGGCAGTGCCTAGCAGACGTGGAATTGCTCCCGCCGGAAGACCGCCATCAGCTTGTCACCGAATTTAATCGGACGGATGCTCCTTATCCCGGAACGAAGACCATTCAGCAGTTGTTTGAGGAGCAAGTGCTGAAAACGCCGGATCGGCCGGCGGTCAAAATGGGCGATCAAGTCCTGACTTATCAAGCGTTGAATGCAGAAGCCAACCGGATGGCCCGTTGGTTGCGGAAACAAGGGGTCACCAGGGGAAGTGTCGTCGGTTTGATGGTGAACCGTTCCCCCTTGATGATGGTCAGCCTGTTGGCGATTGTAAAGGCGGGAGGAACTTATTTGCCGATTGATCCCGAATATCCGGATGAGCGGATCCGGTTTATGCTGGAAGACAGTCAGGCATCCATGTTGTTGGTGGAACCCGGGATTTCCGCTCCCGTTTCTTATCAAGGGACGGTGGCCGGATTGTCTCCGGACGTTTGGAAAGATGAGGATCCCGGAAATTTGCCGAATATCAACGCCCCGGAAGACGTCCTGTATATCATCTACACTTCCGGTTCCACGGGTACGCCCAAAGGCATCGAAACCATGCACTATAACGTGATCCGGACCATGTTTAATAACGGATATATCGAAATGGATCCTTCGGATCGCGTGTTGCAATTGTCCAATTATGCTTTCGACGGATCGACTTTTGACATTTACATTTCGTTGTTGCACGGAGCGCAACTCACCTTGGTCTCCACAGAAGCTTTATTGGATTTGAATCAATTATCCCGGCTGATCCGGGAAGAACAAATCACGGTCACTTTTATCACGACGGCGTTGTTTAACACCCTGGTTGACTTGGATTTGGAATGTTTGCGAAACGTCCGGAAAATTTTGTTTGGCGGGGAAAAAGTCTCCTTTTCCCATGTGAAAAGAGCGGTGGAGTTTTTGGGTGAGGACCGCGTTGTTCATGTGTACGGGCCGACAGAAACCACCGTGTTCGCTACCTATTGTCCGGTTGGGTTCAAGCATATCGAGCGGGGAATTATTCCGATCGGGAAACCTCTCCATAACACCCGGCTTTATGTGCTGAATTCCGAGAACCAATTGCAGCCGGTCGGGGTGCCCGGCGAACTGTGCATCAGCGGAGACGGGGTGGCCAAAGGATATTTGAACCGCCCCGAACTGACGGAAGAGCGGTTTGTACGGGAGCCTTTCGCACCGTACGGGCGCATGTACAAAACCGGCGATTTGGTGCGTTGGTTGCCGGATGGCACGATTGAGTATTTGGACCGTTTGGATGATCAGGTCAAAATCCGGGGACATCGCATTGAATTGGGCGAGATCCAACGGAAGTTGCTGGAGGAAGAAGCGGTCCGGGAAGCCTTTGTGATGATGGACCGGGATGAAAAAGGGCAATCTTACTTATGCGCTTATTGCGTGGCCGAAGGTGAAATCGATGTTTCCAAACTGCGGCAAAACTTGCAGAAAAAACTGCCGGATTACATGGTGCCCGGATATTTTGTCCAAATGGATTCGCTGCCGTTAAATGCCAATGGGAAAGTCGACAAACGCGCATTGCCAAAACCGGATTTGCGCAAGGCAGTTTCGGCCGGATATGTGGAGCCTTCAACGGAAACCGAAGCCTTGTTGGCCGAAGTGTGGAAGGAAGTTTTGGGGGCCGAACGGGTCGGAATCCATGACAATTTCTTTGAGCTGGGCGGTGATTCAATCAAGGCGATCCGGATTGCGGCCCGGTTGAACCAGCAGCAAATGAAACTGGAAACCAAGCAGTTGTTCCGTCATCCCACGATCGCGGAACTCGCTCCTTCTATCCGGCGCGGTGTCTCCCGGATGGAGGAAGAGGAAGTGGTTTCCGGCGACGTCCGGTTGACTCCGATTCAAAAATGGTTCTTCTCCCTTCAATCCGAGGAGCCGCACCACTTTAACCAAGCCATGATGCTCTTCTCCGGTGAAGGATGGAACCCGGCTTGGGTCGGTCAGGCATTCCAGGGGTTGGTCCGGCATCATGATGCGCTCCGGATGACGTATGATTTCACAAACGGTGAAATTAAACAGACGAATCAACCCTTGGAACATCAGGCGTTTACCTTGGATGTATTCGATTTGACCGGATCGGAACCGGCAAACATCGAAGCATATGCGAACAAGCTGCAGAAGGGGATCGATCTAAAAAACGGCCCGCTGGTCCGGTTGGGGGTTTTTCACACCCGTGAAGGGGATTACTTGCTGATGGTGATTCATCATTTGGTGGTGGACGGCGTCTCCTGGCGCATTCTGTTGGAAGATTTCCAAACGGCTTATGATCTGGCATCCAAACATGAGGAGATCCGGTTGCCCCAAAAAACCAGTTCCTTTAAAACTTGGGCAAAACAACTCTTTCAATTTGCAAACAGTAAAAAGATGTTGAAAGAAAAAAGTTATTGGAAGCGGGTTTGTTCACAAACCGTGCCGGCTTTGCCCAAAGATGAGGAATGGACCGGGGAAAAACTCCATCGGGACATGCGGGAGATCGAATTGGAGCTGGATGAAGAGCTCACCAGGAAACTGCTGACCCAAGCACATCAGGCTTATCACACGGAAATGAACGACCTGTTGTTGACCGGACTTGCGTTGGCTGTTCATGAATGGACGGGACAATCGAAAGTGGCGCTTCATCTGGAAGGACATGGCCGGGAAGAATTGTTTGACGGTGTGGATTTAAACCGGACAGTGGGTTGGTTTACCACCATGTATCCCGTTGTTTTCGAGCTGAATGAGACGGAGTTGTCCCGTGTGATTCCAAGCGTCAAGGAAACGTTGCGCCATGTTCCGGAAAAGGGAATCGGTTACGGAATCCTGCGTTATCTGACCTCTTTGGACCACAAACAAGACTTGTCCTTTGCGCTCGAACCGGAGATCAGCTTTAACTATCTCGGGCAGTTTGAAAAAGAAGGATTGGGATTTGGCTCCATATCCACTGGAGACTGGTTCAGCCCGCTGACGCCGGAACCGCATGTGCTTACGTTTAACAGTATGGTGGCAGACGGCAAATTCAAAGTCCGGGTGGGATACAATCACCGTTTCTTCCGCAGGGAAACCATTGAGACGGTGGGACGCCGCTTTTTGGAACATTTGACCCGGTGTGTCCGTCACTGTTTAAAGCAAGCTGAACCGGTTCGCACACCAAGTGATTTCAGCGCCAGGGATCTGTCGATTCGTGAACTGGAACAGATTTGTTCGGTGTTGGATCCGAAGAACATTCATGATATTTATCCGCTCTCTTCTTTGCAAAAAGGGATTTTGTTTCATGCGATCCAAACACGGATCCAATATTTTGAGCAGTTTTACATCGATTTGGACGGAGAAGTGGACTTTGCCGCTTTTGAAAAGAGCTTGGATGATTTGGTTCAAAAGCATGATGTGTTGCGAACGGTTTTCTTGTACCAAAAATTGAATCAACCGTTCCAGACCGTGTTGAAAGAGGTTGAAGTTCCGTTCCGGTTTGTGGATTTAAGCCATCAATCCAAAGAGGAACAAGAAGCTTTCTTAACCCGGTTTAAACGGGAGGACCGGAAAAAAGGGTTTGCCACCTTGCATGAGCCGTTGATCCGCTTTGCGTTGTTCAAATTGGGCGATCGACGTTACCACTTTGTGTGGAGTTTTCACCACATCTTGTTTGACGGCTGGTGCATCGGCCTGTTGTTGGAAGATTGGCTGGACATGTACCGGGCGCGGATGAAGCATGCCACGCTCAAAGTGGAAAAAATGGCTCCTTACCGGGATTATATCCGGTGGCTGGAAGAACAAGACCATGAAGAAGCCAAAGCGTTCTGGAGAGATTATTTGCACGGTTATGAACAGACAGCCAGCGTTTTGAGCAAAGGCATCGGGAGGAAGACCGATTTTGAACTGGAAAAGAAAGAGGTCAGTTTCCGCCTTCCCCGCCATTTGAGCGACCGGTTGTCGATGTTGGCGCAAAAATATCATGTGACACCGAGTACGTTCTTCCAGGCGATCTGGGGAGTGTTGTTGCAGAAATACAACAATACGGATGATGTTGTGTTCGGAACCGTGGTATCCGGAAGGCCGTCGGACCTGCCGCAAGTGGAAAAAATGGTCGGGCTGTTTATCAATACGGTCCCGGTGCGGATCCGCCGGCAGGAAAACGAACCGTTCTCCGAATTGTTGGTGCGGACCCAACGCGAAATTCTGGAGGCGGAAAAAGCCCATTATGCGTCGTTGGCGGAGATTCAGGCCAACACCCCATTGTTTAATCAGCTGTTCGATCACATTATGGCCTTTGAAAACTATCCGCTGGACATGGAGGCTTTAAAAAGCAAAGAGAAAGATCTCGGATTTTCCATCGCCGGTTTGGATGCGTTTGAACAGGACAGCTATGGGTTGGGATTGATCATTTACCCCGGGGACGAATGGTTGATCAAGCTGAAGTATGATACGGAAGTGTATACAGAGTCCGACATGCAGCATGTGATTTTGCATCTGCAAACCTTGATGGAACAAATCATGGAGAATCCGGAAACGCCTTTGGACCGGTTGGAAATTGTCTCGGAAAAAGAGAAACAGCAATTGCTTGTGGACTTTAACCGCACAAAGACGGACTATCCGAAACAAGCCGTGATTCAGCAGTTGTTCGAACAACAGGTGAATAAAACCCCCGATCACCCGGCGCTGGTTTACAAAGAAGAAGTCTGGACTTACAAAAAGTTGAATGAACAAGCCAACCAACTGGCGAGAAATCTCCGCAACAAAGGAATCGGCCGCGAACAGATTGTCGGGATCATGATGAACCGGTCGCCGGAGTTGATCGTCTCCATTTTGGCGGTATTAAAAGCCGGTGGCGCATATCTCCCCGTGGATCCGGATTATCCGGCCGGGCGAATTCAGTATATGTTGGAGGACAGCCAGGCCAAATTGTTGTTGGTGCAGCCGGGATTGCAAGTGCCGGCCGGTTACCGGGGGAATGTTTTGGTGATCAGCCCGTCACTGATGAGGGGAGACGCCTCCGATTTGCCGGTCATCAACCAGCCGGGCGATCTGGCTTACATGATCTATACCTCCGGCTCGACGGGAAAACCCAAAGGGGTGATGATTGAGCACCGCAACCTTTGCAATCTGGTGCAAATTGCCCAACCTTACGAGATCCGTGAGCACAGCCGTGTTTTACAGTTTGCATCCATCAGTTTTGATGCATCGGTTGCCGAAATCTTCCCCACCTTAACCGTCGGGGCGACCCTGTATCTGGAAGAAAAAATGGTGCTCTTGAATGACCTGGTGAAATATTTGAAAGAAAAAAGGATCAGCAATGTGACATTGCCGCCGTCGGTATTGCAGTCCGTCCCGCATGAAGAATTGCCGGATCTGGAAACGATCATCAGTGCAGGCGAAGCATGCAGCCCGGGGGTGGCTGGTAAATGGGGAGCCGGCCGCACATTTATCAATGCCTACGGGCCGACGGAAGCGACGGTATGCACGACCTACGCCAAGCTTGACGATTCTTCGGATAAAACACCGATTGGAAAGCCTTTTTTCAATCAGCAGGTGTACATTGTCAACAAAGACCATCAACTGCAGCCGGTCGGGGTTCCCGGTGAACTTTGCATCAGCGGGGAAGGGCTGGCCAGAGGTTACTGGAACCGTCCGGAGTTAACTGCGGAAAAGTTTGTGGAAAATCCGTTTGTCCCCGGGACGAAAATGTATAAGACGGGGGACATGGCAAGGTGGCTGGCGGATGGAAACATCGAGTATCTGGGCCGGTTTGATGAACAGGTGAAAATTCGCGGACACCGGGTGGAACTGGGCGAAATTACAGACCGGTTGCTGAAACACCCGAGTGTGGAGAAAGCCGCGGTGGTGGCGCAGACCAATGAAAACGGGACGTCGTATTTGTGTGCTTACTTCACGGCCAAGGATTCCTGGTCGGTTCCGGAATTGCGCCGGTATATGATGGAAGAGTTGCCTGAGTACATGGTTCCTGCGTTTTTTGTGGAACTGGAACAACTCCCGCTGACCCCCAATGGAAAAATTGACAAGAAAGCGTTGCCCAAACCGGACGGGATGGTTCAAACCGGTAAGAAACACACTTCACCCACCAATGAAGCGGAGAAAAAGCTGGTTCAAATCTGGAAAGAAGTGCTCGGTTTGGAAAGGATCGGGATTCATGATGACTTCTTTGAACTGGGCGGGGATTCGATCAAAGCCATTCAGATTGTATCCCGGTTGCATCAATGGGATTACAAACTGAAAATCAACGACCTGTTCGACCATCCCACCATTTATGAACTGGCACCGTATGTACAAACGGTCGAATCGGCGGATGATGAGGGGATCGTCGAGGGGGAAGTCCCGCTGACCCCGATCCAGCATTGGTTCTTCGAGCAGGAGTTTATGAATGCCCATCATTGGAATCAATCGATGATGCTTTATCATCCGGATGGCTGGCAAAAAGACCGGGTGCATCAAGCTTTCTCCAAGCTGGTGGAACATCATGACGCGCTGAGAATGCGCTTTGAGCAGGATGGCGAAAAATGGGTGCAGATCAACCGGGGTTTGGCAGAAGGGGAGTCTTTCAACCTTTATGAATTTGATTTGCGGCATGTCAACGATCCGGAATCGCACATTAAGTCCATGGCCAATGAATTGCAGCGGAACCATGATTTCAACCAGGGCAAATTGATCCAATTGGGATTGTTTAAAACCTGCCAAGGGGATCATTTGTTGATCATTCTTCACCATTTGCTGGTGGACGGTGTGTCGTGGCGTATTCTGCTGGAGGATTTTGCAACCGCCTATCAACAGGTTTCCGCAAACCAGGAGGTTGTTTTGCCCAAGAAAACCACATCTTTTAAAAAATGGAGCAAAAAGTTAACCGACTATGCCGATTCACCACAAGCCTTGTCCGAACTGAATTATTGGAAGCAGGTGGAAAATACGGATGTTCCGGCCCTGCCGAGGGATTTCACCCTGCCGACAGAGCGGCGCCTCATGGATCACGCCACCATCAAAGTGGAACTGACCGAAGAAGAAACGAAACAATTGCTTACCGAAGTGCACCATGCATACCATACCGAAATCAATGACCTGTTGTTGACCGCACTGGTCTTGGCTGTCCGCAAGTGGACGGGGCAGGAACGGGTGGCCGTCAATCTGGAAGGGCACGGCCGGGAAGAGATTGTGCAAGATGTGGATTTAAGCCGCACCATCGGTTGGTTTACGACGGCGTTTCCGGTTGTTTTTGATCTGGATACAGACCATGAAGGGGATGCCATCAAGCAGGTGAAAGAGCAATTGCGGAAGATTCCCAATAAAGGAATTGGTTACGGGATTTTGAAATACCTGACTTCCCCCGTAAAAAAAGAAGGGATGACCTGGAACCTGAAACCGGAGATCAGTTTCAACTATTTGGGGCAGTTCGATCAGGAAAATGAAGACCAAGGGTTCCAAATTTCCCCGATGCCGACGGGCGATGAGTTCAGTCCGTTGTCGACCAGCCCTTATCTGCTGAATATTTACGGTAAAGTGATGGATCAACGATTGGAGATGAACTTCCAGTATCATCAGCATGTTTACCGTTTCGAAACCGTGGCCCGTTTGGCGGAAGAGTTTGTTCGTCAGTTGCGCCGGCTCATCGGGCATTGCGTATCCAAAGAAGAAGAAGAATGGACCCCCAGTGACTTTAGTTCCAAAGATTTGACGTTTGAGGAGCTGGAGGATATTTCCGACTTGCTTTCCGATTTGTAAACGCCATGCCAAGAGTCCTTTGCACGAAGGGCTCTTGGCTTAACATCCTTGTCTGACTATTGGAGGAGATCGGTTATGGATAAAAAACAGGTGGACATCTATTCTTTGTCTCCCATGCAAGAGGCCATGCTGTTTCACTCGTTATATGACCAGGGGAATTCTTATTTCAAACAATTGTTGATCCAGGTGGAGGGGGATTTTGATCCCGGACTGTTTGAAAAGAGTTTAAATGTTTTGATTGAACGATATGACATTTTCCGGACTGTGTTTATTTATAAAAAGCTGAAAAAACCACGTCAGGTGGTTTTGCAAGAGCGCGAAGCAAAGATTCAGTATCAGGATTTTTCCCATCTTCCGGAAGATGAGCGGAATCAAAAGGTTGAAGCATGGATCCGAAAAGATCAAACCAAACCGTTTGATTTGTCCAAAGATTTGTTATTGCGTGTTTCCGTGTTGAAAACAGGGTCCCAACAGTATCAAATCGTGTTCAGCAGCCACCACATCCTTTTGGACGGATGGTGCATGGGGATCGTGTTTGAAGATTTGTTCGAAATTTATGGGAAGCTGAAAAATCACGAGCCCGTTCCCCACAAAAAAGTGACGCAATATCGTGAATTGATCCGTTGGCTGGAAAAGCAGGATCCCAAAGAAACAGAACAATATTGGAAACAGTATTTGGCCGGGTATGAAGGACATGCCAAAATTCCGGGAGCGAAGACCGCCCGCGGCGATGATTACAAGTTGGAGGAAGTCCGTTGTTCACTGGATGAAACGTTGACCAGGCAATTCAATCAATTTGCCCGTGAACATCATCTCACCCTGAACAATGTATTCCAAACAATCTGGGGATTGATGTTGCAACGGTACAATCACACGGACGATGTGGTGTTCGGTTCCGTGGTCTCCGGAAGGAACAAAGACATTCCCCGGATCCAGGAGATCGTCGGTTTGTTCATCAACACCATTCCCGTGCGGGTCAAAGCAAGTGGCGATGACCGTTTTACAGACCTGGCCAAACAAGTTCAACAAGCGGCGCTGGCCTCGGAGCCGTATGATTATGCTTCGTTGACCGATATTCAGAAAGCGGCGCAATGCGGCGATTCATTGGTTGACCATATTGTTGTGTTTGAAAACTATGTGTTTGATTCGAATATTTTCCATAAATACCGGCATCAATTGGGGTTTGTCGTGACGGATGCCAAGAGCACGTTTGAAAAAACCAACTATGACTTTAATGTGATTGTGGTGCCCTGTGAGAAATTCACCATTTTGTTTCAATACAACGGACATGTTTACCAAAGAGAAGACGTGGAATCCTGTTTCCGCCACTTTGAACGGATTGTCCGCCAAGTCATTGGAAATCCGGATTGCCGCTTGAAAGACATTGAACTGGTTTCCCCTTCGGAAAAGCAGCAGTGGTTTGCATGGAATCAAACGGATGCGGATTATCCGCGCGATAAAACCGTTCAGGAGATTTTTGAAGAACAGGCGGAAAAATCCCCGCAGCAAATCGCCTTGAAATTCCGGGGAAATACGATGACTTACAAAGAGTTGAATGAACAAGCCAATCAGCTGGCGAGAGTGTTGAGGAAACAAGGCGTCCGGCGCGGGGACATTGTCGGATTGATGACGGAACGCTCCTTTGAAATGATGATCGGCATGCTGGCGGCCGTCAAAGCCGGTGGGGCTTATATGCCGATTGATCCGGAATATCCCGCTGGACGGAAGGAATTTATGCTGGAAGATGCCGGTGCTCCGGTTCTCATGGTGCAGCCGGGACTGAAAGTGCCTGCCGGTTATAAAGGGCGAGTGGTTGAACTGGCTCCCGGCGAATGGGCGGGGGAGGAAAAAAGCAACCCGGAGAATGTCAATCAGCCGGATGATCTTCTTTACGTCATGTATACTTCCGGATCGACCGGCAAGCCCAAAGGCATTTTGACCATGCATTACAATGTGATGCGGACGGTCATCAACAACGGGTACATACAGGTGCATGCCGGGGACCGGCTGTTGCAGTTGTCAAACTATTGTTTTGACGGATCCACGTTTGATATCTACAATGCCTTGTTGAACGGAGCGACGCTGGTTTTGGTGCCCAAAGAGGCTTCCAAAGACTTTGCGGAGCTGACCAAATTGATCCGAGAGGAAGAGATTACCGTCACCTTTATGACCACTTCCCTGTTTAACACGGTTGTGGATTTGGATCTTGATTGTTTAAAGAGCTTGCGCAAAATTGTCTTTGGCGGGGAAAAGGCTTCGGTCAAACATGTTCAAAAGGCAGTGCAAGCACTGGGCGGAAACCGACTGATCAACGGGTACGGACCGACGGAAACCACGGTTTTTGCCGCGACTTATTCCATTGATCCGTCAGTGTTGGCCACCCATCGTGTACCGATTGGAAAGCCGCTGAACAACACCAAGCTGTATGTGTTGGACCCATGGGGAAATCTTCAACCGCCCGGCATTCCCGGTGAACTTTGCGTCAGTGGCGACGGAGTGGCCAAAGCGTATTTAAACCGTCCCGAATTAAATGAAACCCGCTTTGTTCCGGATCCGTTCCATCCCGGTCAACGCATGTACCGCACCGGGGATTTGGTTCGCCTGTTGCCGGATGGCCATTTGGAATATTTGGAGCGGATGGATAATCAGGTGAAAATCCGGGGACACCGCATTGAGACCGGTGAAGTGGAAAAGAGGTTGTTTGCCCATCCCGATGTGCTGAATGCGGTGGTTTTGGCCGATAAAGATCAGAACGGGCATTCCTATTTGTGTGCTTATGTTGTGTTGAAAAAGGATGTTTCACCTTCTGCATTGCGTGAGCACATGACCAAAGCGTTGCCGGAATACATGATCCCCGATTACTTTGTAAAATTGGATCAACTTCCGTTTACGCCCAATGGAAAAGTGGACTTGCGGGCGCTTCCCAAACCGGCCGAAGCGTTGGAGAACAAAAGGGATTATGTTGCCCCCACGAACCCCGTGGAAGAAAAGCTGGTAAAAGTCTGGCAGGTAGTTTTGGATGCGGACCAAGTCGGGATTCATGACAACTTCTTTGACCTGGGGGGACACTCCCTGAAAGCGATGATGCTTTCTTCGCGGGTGAGCAAGGAATTGAATGCAGACTTGCCGCTAAGAGAGATTTTTGCCCGGCCGACGGTGCAGCAACAAGCGGCCTTTTTGCAGAATAAGGGCAAAAAAGAATACACCGGGATTGCCCCGGCACCGGAGATGAAGGATTATCCGGTGACTTCCGCGCAGAAGCGCTTATATATGGTCAGCCAACTGGAAGGAAGCGGCACCAGTTACAATATGCCTTATGTATTCCGGATTCACGGCGATTTGTCCGTTCCCGGTTTAAAGAGCGTCTTGCAATCTTTGGTGGACCGGCATGAATCGTTGAGAACGTCTTTCCACATGATTAACGGGGAATTAAGGCAGAAAATTCATCCCGATGCCCGATTGGTCGTCGAGCAGTACCAAGCCGGCGGGAAACGGCAAGAAAAACGTGTCATTCAAGAATTTATCCGCCCGTTTGATTTGCAAAAGGCGCCGTTGATGCGTGCCGGGGTGTTGGAACGTACGGACACCCGCGAGAAGTATCTGTTATTGGATTTTCATCATATCGTGGCTGATGGTGTTTCGATCCGGCAATGGTTTTCCGATTTTTCGCGGCGGATGCAAGGTGAAGAGTTGCCTTCGCTTCCATTGCAATACAAGGATTATGCTTATTGGCAACAACATGAGAAACAAAAAGACCGTTGGAAAAAACATGAGGAGTACTGGTTGAATGAACTTTCCGGCGAGTTGCCCGTTTTGCAATTGCCGACCGACTTTCCGCGCCCGAAAGTTCAGCGTTTTGACGGGGATCAGCTTCCTTTTGAAATGGATGCGGGATTGTCGGATCAATTAAGGCAGCTGGCCGATGAGCAGAATGCAACGATGTATATGGTGTTGTTTGCCGCTTATCATGTCCTGCTCTCCAAGTACGCGGGGCAAGAGGATTTGATTGTCGGGTCCCCCATCGCCGGAAGGCCGCATGCGGACCTGGAGACCATGGTGGGCATGTTTGCCAACACGCTGGCGGTCCGAACCCGGCCGACACCGGATGTATCGTTTGCCCGGTTTCTCGCGCAATTGAAAGAACAAATCCTGGAGATGGCAGAGCATCAGGATTATCCCTTTGAAGAACTGGTGGAAAAATTGGGGGCAGAACGGGATTTAAGCCGGAATCCTCTTTTTGACACCATGTTTTCTTTGCAAAAGAATGAATTGCCGGCCCTTCAGTTCCGGGATTTTTCGCTGGAACTGGTTGAATGGGATTGGAAAAAATCCAAGTTTGATATGAGCTGGATCCTGATTGAAGATGAGAAAATCAAAGGGATCGTGGAATTTGCCACGCATCTGTTCAAGCCGGAAACGGTAAAAAGAATGGTTCGCCATTATCTTCATATTTTGAAGCAAATCACGGAGAATCCGGACCTTCGCCTGTCTGACATCAAATTGATCACGCCGGATGAAGAGCATCAGGTGTTAAACAATTTCAATGCGTATCGTCTGGAGTATCCGCGGGGAAGAACGATTCCGTCCTGGTTTGAAGAACGGGTGGAAAAGCAACCGGACCGGGTGGCGGTGGTTTGCGGCGAAGAACGATGGACTTATGAAGAGTTAAACCGGCAGGCCAACCGCCTGGCCCATGCACTGAGGAAGAAAGGGATCGGCCGCGAGCAACTGGTCGGCATCCTGATGTCCCCGTCCAAAGAGATGATGGCGGCGGTTCTCGGTGTCTTGAAAGCAGGGGCGGCGTACGTTCCGATTGATCCGCAATATCCGGCGGACCGGATCCAATATATGTTGAAAGACAGCGGGGCTTCCCTGCTTCTTACGGATCGTCCGGCCGTTCCCGGCAGTTCCGGCGTGGAAGTTTGGGATATGAAAACCAAAGATTTCCGGGGCGAATCCGATGACAACCCCGCACCGGTGAACAAAGAACGGGATTTGGCCTACGTGATCTATACGTCCGGTTCCACCGGAAAACCCAAAGGCGTGATGGTGGAACACGCCTCCCTGATCAATTTGTGCGAGTGGCATATCCGTGAATTTCAGGTGACACCGGAAGATAAGAGCATCAAATACGCAGGGGTCGGATTTGATGCCTCGGTATGGGAGATTTTCCCGACTTGGATGGCGGGAGCGTCGCTTCACATCATTGAAGAGGCCATGCGTTATGATCTGGAGGCACTGAACCGGTACATGGAGGAACAAGGGATCACGGTTGCTTTTCTGCCGACGCCGGTGGCGGAGCAATTTATGCAGCTGGAAAACCGCAGCCTGAGAGTGTTGCTGACCGGAGGGGACCGGTTGGGCCGGGTGTTGCCGCAGAACTACACGGTGGTCAACAATTACGGTCCGACGGAGAACACGGTGGTGACGACGAGCACCCCGGTGCGTCCGGATGAACCCGTCACGATTGGAAAACCGGTGGCCAATAACCAAGTGTATGTGTTGAATGAACATCAGCAATTGCAGCCAATCGGCGTGCCGGGCGAGCTGTGCGTCAGCGGGGAAAGTCTGGCCCGGGGATATTTGAACCGGCCGGAGCTGACGGCGGAGAAGTTTGTGCCCAACCCTTATGTGCCGGGGCAAAAGATGTACCGGACCGGAGACCTGGTGCGGTGGCTGCCGGACGGGCGCTTGGAGTTTCTGGGGCGGATGGATGAACAGGTGAAAATCCGCGGTTACCGCATTGAGCCCGGGGAAATCGCCAACCGTTTGTTGGAGCATGCTTCCGTCCGGGAGGCCTTTGTCACCGTGCAGCTGAATCCTTCTCAAGAACCGGAGTTATGCGCATATTTCGCAGCGTCGTCTCCTTGTTCCGCGGAAGAATTACGGGAGCATTTAAGTTTGAAATTGCCTGACTATATGGTTCCGGTTCACTTTATCGAAATGGAGAAACTCCCCTTGACCCGAAACGGCAAAGTGGACAAAAAAGCATTGCCGTTGCCGGAAGCCGACACAACCCGAAGCAAGGATGACGCACCGGCCACTCCCGCCGAGGAGCAGTTGATCGAAATCTTGAAGGAGATTCTCCATACCGAACAAATCGGAGTCCATGACAGCTTCTTTGAAAAAGGCGGCAACTCCCTCAAAGGGATGATGTTGTCGGCGCAAGTCCACAAGAGGATGCATACGGAACTGCCTTTGCGGGAGATTTTTGCCCGTCCCGTGGTCAAGGAGATGGCCGCATACATCGAAAATCAAAAAACGGCTGAACATGTCACCATCGCTCCGGCACCCAAACAAGCTTATTATCCGGTTTCATCCGCACAGAAACGGTTGTATGTGGTCGGGCAACTGGAAGGAATGGGCACCGGTTACAACATGCCCCATGCCTTTTCCATCCGAGGAAATTTGCCGGTTTCCCGTCTCGAAGAAGCATTCCGGGCACTGGTGGAACGGCACGAATCCTTGCGTACTTCGTTTCATCTCATTGATGGCGAATTGATGCAAAAAATTCACACCGATGTGGAGGTCCGGGTGGAGTGTTATCAAGCCCAAGACCGTCCGGATGTGAAACAAAAAATTCAACAGTTCATCCGTCCGTTTGATTTGGAGAAAGCTCCGTTGATCCGGGTCGGAATGATCGAACATGCCAAGGAAGAAGAACAGATCTTGGTCATCGACATGCATCACATCATTTCCGACGGTGTCTCGATGAACATCCTGTTTCAGGATTTGATGAAATGCCTGCAGGGAGAAACGTTGCCGCCGGTGTCCCTGCAATACAAGGATTATGCCGTCTGGCAACAAAAGGAAAAACAAAAGGATACATGGAAAAAACACGAGCAGTATTGGATGAATGAACTTTCCGGCGAACTTCCGGTTCTGGATTTGCCCACGGATTACCCGCGACCTTCCGTACAACGGTTTGACGGGGACCATATGACTTTCGAAATCGAGCACCGATTAACCCGGAAATTGAAACGGCTTGTGGATGAGAAAAAAGCGACTCCGTATATGGCACTCTTGGCCGCGTACAATTTGCTGTTGGCGAAATACACCGGCAAAAACGACATCATTGTCGGCTCGCCGATTGCCGGAAGATCGTATGCCGGTTTGGAGTCCATGGTGGGGATGTTTGCCAATACGCTTGTGGTTCGCAACCGGATTGATTTGCAACTTCCGTTCACAGTCTTTTTGGAACAGGTGAAGGAACGCGTCCTGCAAATGTATGAGCATCAGGATTATCCGTTTGAGGAATTGGTGGAAAAGTTGGATACCCAGCGCGATTTGAGCCGCAATCCCCTTTTTGACACCATGTTTGCCATGCAGAATATCGACATGCCGGTTTTTAAACTGCAAGGATTGAAAATTCGCCAAGGGGAGATTGAATGGAAAAAATCGAAATTTGATTTGAGCTGGATGATGGCGGAAGATGAGGAAACATTCAAAGGGGTTGTCGAATACAGCACTTGCCTGTTCAAGCCCGGGACCATCCGGCGTATGATTGGCCACTTTGTTCATATTCTCGAACAGATTGTGGATGATCCCGGAATTCGGCTGTGCGAGATTGAGCTTCCCACGTTGGAAGAGAAATGGGAGATATTGCAAACCTTTAACCAAACCCGTCTGGAGTATCCGCGGGGAAGAACGATTCCGTCCTGGTTTGAAGAACGGGTGGAAAAGCAACCGGACCGGGTGGCGGTGGTTTGCGGCGAAGAACGATGGACTTATGAAGAGTTAAACCGGCAGGCCAACCGCCTGGCCCATGCACTGAGGAAGAAAGGGATCGGCCGCGAGCAACTGGTCGGCATCCTGATGTCCCCGTCCAAAGAGATGATGGCGGCGGTTCTCGGTGTCTTGAAAGCAGGGGCGGCGTACGTTCCGATTGATCCGCAATATCCGGCGGACCGGATCCAATATATGTTGAAAGACAGCGGGGCTTCCCTGCTTCTTACGGATCGTCCGGCCGTTCCCGGCAGTTCCGGCGTGGAAGTTTGGGATATGAAAACCAAAGATTTCCGGGGCGAATCCGATGACAACCCCGCACCGGTGAACAAAGAACGGGATTTGGCCTACGTGATCTATACGTCCGGTTCCACCGGAAAACCCAAAGGCGTGATGGTGGAACACGCCTCCCTGATCAATTTGTGCGAGTGGCATATCCGTGAATTTCAGGTGACACCGGAAGATAAGAGCATCAAATACGCAGGGGTCGGATTTGATGCCTCGGTATGGGAGATTTTCCCGACTTGGATGGCGGGAGCGTCGCTTCACATCATTGAAGAGGCCATGCGTTATGATCTGGAGGCACTGAACCGGTACATGGAGGAACAAGGGATCACGGTTGCTTTTCTGCCGACGCCGGTGGCGGAGCAATTTATGCAGCTGGAAAACCGCAGCCTGAGAGTGTTGCTGACCGGAGGGGACCGGTTGGGCCGGGTGTTGCCGCAGAACTACACGGTGGTCAACAATTACGGTCCGACGGAGAACACGGTGGTGACGACGAGCACCCCGGTGCGTCCGGATGAACCCGTCACGATTGGAAAACCGGTGGCCAATAACCAAGTGTATGTGTTGAATGAACATCAGCAATTGCAGCCAATCGGCGTGCCGGGCGAGCTGTGCGTCAGCGGGGAAAGTCTGGCCCGGGGATATTTGAACCGGCCGGAGCTGACGGCGGAGAAGTTTGTGCCCAACCCTTATGTGCCGGGGCAAAAGATGTACCGGACCGGAGACCTGGTGCGGTGGCTGCCGGACGGGCGCTTGGAGTTTCTGGGGCGGATGGATGAACAGGTGAAAATCCGCGGTTACCGCATTGAGCCCGGGGAAGTGGCCGACAAGTTGTTAAGTTACCCGACCGTGAAAGAAGCCGTGGTGGTGGCGCAAACCGACGAACAAAATCAGTTGCATTTGTGTGCATATTTCACGGCATCGGAGTCCTGTTCCATTCCTGCATTGCGGCAGTACCTGGGGAAAGAATTGCCTGATTACATGATTCCGGCTTACTTCATGCAGATGGAGCAACTGCCGTTGAATGCCAACGGTAAAGTGGACAAAAACATGCTTCCCAAACCCAAACAGATGCAAACCGGCGTGGAATATGCGGCGCCCCGCAATGAAAAGGAACGCATCCTGGCGGAGCTGTGGCAGGAAATTTTGCATCTGGACCGGGTGGGGATCCATGATCAGTTTTTTGAGCTTGGTGGCGATTCCATCAAAGCGATGCAAATTGCTTCCCGCTTGAAACCGTACCGGCTGAAAATGAGATTGAAAGATTTGTTCCAACATCCGACCATTGAAGAATTGGCACCGCATGTTTTCCCCATCGATGAAGACCGGGAAGATGAAGTGGTGACGGGGGAAGTTGCGCTGACCCCGATTCAGCGTTGGATGTTTGAGCAGACGGATTATCCGGATCAATGGAACATGGCCATTGTCCTGTACCGGAAACAAGGATGGGATGCCGACGTGTTAAAACCGGTTTTCCATGCATTGGTGACCCATCACGACGCCTTGAGGATGTCGTTTAAGCGGGAAGATCACCAGGTGAAGGCATGGAACCGGGATGTGGAAGGGGAATTTTTCTCCATCCACGAGTTTGATGTCAACAGCGAAGACCTTTCGGAAGTGATGAAAAGAGAAGCCAACCGTTTGCATCAATCGCTTCGCATCCATGGCGGGCCGTTGATCCGCCTGGGGATTTTCCATACCGGCGAAGGGGATTACTTGCTGATCATTATCCACCATTTGGTGATGGATGCCGTTTCCTGGAGGGTGCTGTCCGAAGATCTCCATACTGCGTACCGGCAACAATTGGACGGGGAACCCTTGGCTTTGCCGCAAAAAACGACTTCGTTTCAATCATGGAGCCATCAATTGCAAGAGTTTGCGAACAGTCCGGCGTTGCTTGCTGAAATTCCGTATTGGAAAGCGACCGACCGCCTCCAGGTTCCAAAACTTCCGGTCGATAAAAAAGAAACGTCGGATTATGCCAATCAGCATATCGGCATCGTTTGGGGACGCCTCCCGAAAGAGGAAACCGGTGACTTGTTGTCCGGGGCGCATCGCGCTTACCAGACGGAGATGAATGATTTGTTGTTGTCGGGCTTGGTGATGACCTTGAACAGTTGGACCCATGGCAAGGTGGCGGTCCAATTGGAAGGGCACGGCCGGGAGGAAGTGATTGACGGGGTGGATTTGACCCGGACCGTCGGATGGTTTACATCCATGTATCCGGTCGTGTTTGAACAAGATTCCAAAAATCCGGCCGAAGTCATTCCGTATGTGAAAGAAGTCTTGCGGCGGGTTCCCAATAAAGGAATCGGATACGGGATTCTCAAATATTTAACCCGTCCCGAAAACAAACAAGATCTTTCGTTCCGGCTCAAACCTGAGATCAACTTCAACTATCAAGGGGAGTTTGTCCGGGATGTGGAGAAGGAGGACATCCAAATCGTGTCCCTGCCGGTGGGAGAAGGGGTTCATCCCCTGGCTCAGTGGCCGTATAAACTGGATTTCAGTTTGTTTGTTGAAAACGGGGAACTGGTCACGCTGATTCGCTACCACCAAACGCTGTATCATCGGGAGACGATTGAACAACTGAAAAACCTCTATCTCCATCATCTGAAAAGAATTGTGGACTTTTGCCGGGAAGAAGTCTTGGTGAAATAAAGACAATACATCCTGTCAGGGTGTCCAGGCAGGATGTATTGTCATTTACAGAGCCGGAGTTGGCGTCGGTTCGGCAAAATCGTTGCCATATTTTTGGTCGATAAATTCGCGGATTTTTTGATCAGACGTTCCGGATTTTTTCATTTGTATGGATTCTTTGGCAATCTCCAGACAAACCCCGCAAGTGGTGGCATGGGAATCCCATGTGATTTGGTCTTTGGATTGTTGGTGGATAAAGCAATCAAGATTATTGCGATGCCCCACACTTTGTCCGCATCCGCAGAAACACGGGATCCGGGCCAGCAAATCACGGTGTTTGGAGACTTCTTGATAAGTTTGAGCCACCTCCGCGGGATGATCCCGCAAAAAAGAGGGTAATTCATGGGGCGCGGTGGTCTCCCGGACATCTCCCGTTACATGGGAGGATTCACCGGGACCGGCCTCCTGATGCGGATCCGGCTGGCAACCGGCTGTAAATGACAAGCCAAACAGAAACAGAAAGACGGAAAAAAATCGAATCGTTTTTTGATGCATGATCATATGCCCCCCCCTTTTTCCCCACTCATCATAGCAGAAAAAAATGATTTGGTGTGGAGAAGGGGAAATCCGTTTCAAAAAGATAAAAAAAGAGACCGTGTAAACGGTCTCATAGATCCGGGGAACAAAATGATAAAACCACTTGATTGAGATAGCTTGCGTAAGCCAGAGTGGCTATCTCATTTATTATTATAAAGCAATTTGTGAGTTTTTGTTAAGGAAATAAAAGGGAGAAAAAACTTTATTTGGGTGACTTATTCCGTTTTTTAAGTGTTTTATTTTTTTAGATATAATGAAGTGTGAAAAACCATTCTTTACCCGATGGATTATCTGAATGATCCGGCTTGAAAAAGTGGCGATTTTTGCAAAAGGGAGATGAGAGAAAGTTGAAAAAATTGATCAATCATCCTGATGAGGTAGTGGATGAAATGTTGGAGGGCATGGTGGCCGCGTATCCAAAGAGATTGTCCAGGATTCCGGATTATTCCGTCATCGTCCGCAATGAAGCGCCTGTTCAAGGAAAAGTGGGGTTGGTCAGCGGCGGCGGTTCCGGGCATGAACCGGCTCATGCCGGGTATGTGGGAAAAGGCATGCTGGATGCCGCCGTGCTTGGCGAGGTGTTTACCTCCCCCGCGCCCGATCAGATCTTAAGAGCGATCCAGGCAGCCGACGGAGGCAAGGGTGTCTTGTTGATCATCAAAAATTATGCCGGGGACGTCATGAATTTTGAGATGGCGGCAGATATGGCGATAATGGGAGGAATCCCCGTGAAATGCGTGGTGGTCAACGATGATGTGGCCGTTGATGATCCGTCCCGGAGAAGGGGAATTGCCGGAACGGTTTTCGTCCATAAAATTGCCGGAGCTTGGGCCGAAGCGGGGGCGGGTTTGGAGGAGGTTCACCGGATTGCGGAAAAAACCGTGCGAAATATGCGGAGCATGGGAGTGGCTTTAACCGCCTGCACGGTGCCAGCCGTCGGAAAGCCAGGCTTTGAGATCCAAGAGGATGAAATGGAAATCGGAATGGGGATTCATGGGGAACCCGGCATCCAAAGAACGGATCACTTGTCCGCAGATGAAACTGCTGGTGTGTTGTTGGATGCCATTTTTAAGGATTTGCCACTCCAAAAAGGGGACGAGGTTGCGGTCATGTTGAATGGTTTGGGGGCCACGCCGCTGATGGAGTTGTATATTTTGAACAGGAAAGTATCGGAATATTTACAAGATAAGGGGATCCGTGTGTTCTCCACCAGTGTCGGCGAATACATGACTTCGTTGGAGATGGCCGGTTGTTCACTCACCCTCTTGAAACTGGATGATGAATTAAAAAAAGGATTGAAAGAACCATCCGACACCGTATCATGGGTTCAAGCCGAAGGAGGAAGATAAGATGGAATGGACGGTTTTGCAGGTGAAGCATTGGTTATCGGATTTAAGCCGGATCATGCAAGAAAAAAAGGAAGAGCTTTCCCGGTATGATCAGGCGATTGGAGACGGGGATCACGGTGTGAATATGGCTCGCGGCTTTAAGGAAGTGGAGAAGGTCCTGGCGCAAGAACATGATCCTCACGATATCGGGTGGTGGTTCCAAACGGTTTCCCGGATTCTGTTAAGCAAAGTGGGCGGGGCATCCGGCCCGTTGTACGGCACTGTTTTTCTGAAAATGGCTTCGGTTTGCAAAGGAAAAAGCGTGTTGACGCATGAAGAATGGGTAACGGCTTTTTCCGAGGCTCGTCAGGGATTAAAAATGAGAGGAAAAGCGGAGCCCGGCGACAAGACCATGTTGGATGTCTGGGAACCCGTTGTCCGGTACATGGAACAGGCGAAAGAGCAAATCTCCTGGCAAATGGTCAAAGACATTGCCGAACAATCCATGATCGACACCCGGGCGATGGTGGCAAAGAAAGGGCGTGCTTCTTACCTTGGGGAACGCTCGAGAGGTCACTTGGATCCGGGGGCGGTGTCTTCTTATTACTTCTTTTGGACATGGGCAGAACAACCTTGGAAGGGGGACGGACAATGAGCTGTGTCGGATTGGTGCTGGTCTCGCACAGTGAAAAGCTTGTTGAAGGATTGGCGGAATTTCTCTCGCAAATACAAAAAGGCGTACCGGTCGCCATTGCCGGCGGAACGGATGATGGAGAGCTGGGAACCAGCCCGTTTAAGATCAAAGAAGCCATCGAACGCGTTTATTCAGATTGCGGCGTTTTGGTTTTATTTGATATCGGAAGTGCGGCTATGTCCGTCGAAATGGCCATCGAGATGTTGGGGGACGAAAAGAAGATACGCGTGGCGGACGCGCCGTTGGTGGAAGGTGCCTATGCCGCCGCCGTGGAAGCTTCGGTCCGAAGCGATTTGGATAAAGTGGCGGCATCGGCGCAAAAAGCAAGAAATTGGCGCAAATAGGAACATAAAATTCTCCCGGGTTCCTCACAATAAAAAAAGAGAGATGCGCATACTAACTCGTAGTCCATACAAAAGGAGTGAACCCGGTGAAAGAGGATCGAAATGTCCGACCCGATACAGAATCGAATGAAAAAAAGAAGAATGCTGCCGCCCAGATGATCCAGCAGTTGGGGCAAACCAATGTGCCTCAACTGGAAAGCAATATTTTTTGTTTGAGCATCATCGGGCAAATCGAAGGCCATCTGGTGATGCCTCCCCAAAATAAAACGACGAAATATGAACATGTGATTCCGCAGATCGTCGCGGTGGAACAAAACCCGAAGATTGAAGGAATGGTGATTGTTTTAAATACCGTGGGCGGTGATGTGGAAGCCGGTTTGGCCATTGCGGAAATGATTGCGACGATGCGAAAGCCGACGGTTTCGATTGTTCTCGGGGGAGGCCACAGCATCGGGGTTCCCATTGCGGTTGCGGCGGATAAATCCTTCATAGCCGAAACAGCCACCATGACGATTCATCCGGTCCGTCTGAGCGGATTGGTCATCGGCGTGCCCCAAACCTTTGAATATATGGAAAAAATGCAAGATCGGGTGATTCATTTCATTTCCACCCATTCGAACATCACGGAAGAAAAGATCCGTGAGCTCATGTTCCAGACCGGGGATCTGGCCCGGGACATTGGAACCAATGTGATTGGAAAAGATGCGGTGAAATACGGATTGATTGATGAAGTGGGCGGCATTTCCGAAGCGCTTGCCGAATTGAACAAAATGGTGGAGGCCAACAAGCAAAAGGAGATTTTGCAATGATTTACTATTCCACGATCCCGATCGAAATGGCTTTTTACCGCCCGGATGAGTTGAAGCTGAAACAAATTCAAGTGGATGGCGTGAAGATGATTGTCCATGAAGGAGAGGAAGGAGAAGCGACCATTGTCCAAGTGCTGAGCGGAGATCCCGGTCATTTTTTGGATCCTCGTTTTCAGCCGGGAAACACCATCAAAAACGTTCCGACCGTGACAGAGTAAACTGCCGGCCATTGCCGGCAGTTTTTTTGGAGCCGGAAGAAGAACAAAAAGAAAAAATCTTTAAAATCCGGTAGGAGACTTTATGTTGTTTTGGGAAATAAATATCGATAAGATAATACCGACTGGTTATCATTTCCATTATTTACTTTCGGAACGGATGGGTTGAAAAGGTGGTTTCAATGACAAAAAAGAGAAATAAGAAAAAACAGTCGATTGGAACAAAAAAAGAAATTTTGTTTGAGATTTACGGCATTGTCTTATTGGCGTTGTCTTTGGTGACGATTGCCAGTTATGGAGCCATTGGCAGAAATTTGACGTATCTGGCCCGTTTTGTGGTGGGGACATGGGATTTTATCATTCCGCTTTTGGGAATGGGATTGTCCGTGTATGTGATGGCGCATCACCGGTGGCCGGATCATTGGTCGTCGAGGTGGACGGGAGTCGCTTTGGTGTTCATGGCGCTGATCACTTTTAATCATTACTTGACCTTTCACACCATTCAAACGGACAAAGGAATCATTGATCAGACATGGTACATGTTGTTGGAGGAAAGAAATTCAGCGGTTCCACTGGATGTCGGCAGCGGGATGCTGGGGGCTTTTTCCTATGCTTTGCTCCATTTTTTATTCGATGACACTGGCGCTTTGATTGTGCTGGCTGCCGTCGTGTGTTGCGGTTTTTTGCTGATCGCCGACTTTTCATTTACCAAAGCTGCGCTGATCTCTAAGGCCGTTTACAAAAAAGCAAAACAGAAGGGAAAAGATTGCCTGATCGATTGGATCAGGCAATATGACCGCCGCCGGAAGCGGCCATCGAAGCAAAGACGTGTGCAGAAAAAAACCGTCCCTGATGTGGAAGTGAGCAAAGAGCCCATCGATCCGGTGGTTTATGACTTTGAAGATGTGGCTTACCAAGACAGCCGGGAGCAACCGGAGGACGGGGAAAAGCCGGTCCATGAACAAATGAAATTGAAATTGCAGCCATCGGAGCCGGATAGGAAACGACCGAAACAACCCGAACAGGAAGAAGGTTCCGACGAGCCGATTGAAGTCCATTTTCAATCGCAACAAAAGATGGATCACTATGTATTGCCACCGGCCACCTTGTTGAATCCGACAAAGAAAAGGCAGGGCCGGGAGCAGGAAAACGTCCATGAAAAAGCAAAAACGCTGCAGGACAAACTGGAAAGCTTCGGAGTCAAAGCAACGGTGGGGGAAATCCACCGGGGACCGGCTGTCACCCGTTATGAAATCCGTCCGGAAAAAGGGGTGAAAGTGAGCCGGATTGTCAACCTGGCGGATGACATTGCCCTGGCGCTGGCGGCCAAGGATATCCGGATTGAAGCCCCGATCCCGGGAAAATCGGCGGTCGGGATCGAAATCCCCAATTCCAAAGTCGCGCTGGTGGGGTTAAGAGATGTATTGGAGAGTGCGGAATATTTGGGATCGCAATCCAAGTTGAGCATTGCCCTGGGGCGTGATGTGTACGGCAAACCCATTGTGGATAATCTGGCCAAGATGCCGCATCTGCTGGTGGCGGGTGCAACCGGATCGGGAAAAAGCGTTTGCATCAACGGGATCATTGTCAGCCTGCTGTACAAAGCGCGTCCGGACGAAGTGAAATTGATGATGATTGATCCCAAAATGGTTGAATTGAATGTATACAACGGAATTCCCCATTTATTGATTCCGGTTGTCACGGAGCCGCGCAAAGCGGCCATGGCATTGAAAAAAGTGGTGGCGGAAATGGAAAAGCGCTATGAATTGTTTGCCAAGCACGGGGCCCGCGACATCGAACGCTTCAATCAATTGGTGAAAGAAAAGGGATTGGAAGATACAGACCCTCTCCCTTACATTGTTGTCATTGTGGATGAGTTGGCGGATTTGATGATGGTCGCCCCGGCCGATGTGGAAGATGCGATTTGCCGGCTGGCGCAGATGGCCAGGGCTGCCGGGATTCATTTGATCATCGCCACTCAGCGTCCATCGGTCGATGTGATTACCGGCGTGATCAAGTCCAATATCCCGACCCGGATTGCTTTTGCCGTTTCATCTCAGGCCGATTCCCGCACCATCCTGGACATGGGGGGAGCCGAGAAATTGCTCGGACGCGGGGATATGTTGTATCTGCCGACGGGAGCATCCAAACCGACCCGGATCCAAGGGGCTTTCCTTTCGGATGAAGAAGTGGAGCGGGTGGTGAAATTTGTCAAGGAACAACAAGAACCGGATTACCAGGAAGAAATGATTCCCCAATCCCCTGACAAAAATAAATCATCCGTCGACGACGAATTGTTTCCGCAAGCGAAAAAACTGGTCATTGAAGCGCAAACAGCCTCTGTGTCTTTGTTGCAGCGCCGGTTGCGGATCGGTTACACCAGGGCGGCCCGCCTGATTGACATGCTGGAGGCCAAAGGGATTGTCGGCCCTTATGAAGGGAGCAAGCCCCGCGAAGTGTTGGTTTCATCCTTTGACAATGAAACCGGGGAGAGAAAAAAAGAAGCTGTGAAGTAGTCGCGAAGAGGGAAGCCGCCGAGTTTTCAAAGAAAAGAATTTTTTTGCCGGGCAGACGATCATAATAAATGGTGAACATTTCTAACCGGTGCCGTATTCGTCTATAATAGGGGTAATTGACTTGATGAAAAAAGAGGTGAATACTTTGGGTTTTGCTCAGTTTGAAACAGGCTCGATCGGGAATATCCGTGTCCATGTTTGTAACAGCGAAAAGTTTAAAACCACGATGCTGATGGCATTTATTCAACAGGAGTTGTCCCCGGAAACCGTAACCAAAACCGCATTGCTCCCTCAAGTGTTGCAGAGGGGGACACAGTCCCATCCGACCACCCTTTCCTTCCGTCAGAAATTGGATGAGTTGTATGGGGCCATTTTGTTCGGGGATGTATTCAAGCGGGGAGAACGGCATATCATGCAATTTGGCATGGAACTGGCCAATGAACAATATCTCAAGGAATCCCCTTCCCTGCTTGCGGAAGGAATCCGGTTTTTCAGCGAGGTGTTATTAAAACCGGCGCTGGAAAATCAAGCATTCAAGCCGTCCTATTTGGAAGCGGAAAAGAAAAATTTAAAACAGAAAATTGAAAGCTTAAAAGACGATAAAATCCGCTATGCTTCCCATCGCCTGATTGAGGCCATGTGTCAGGGAGAGCCGTATGCGTTGTTTAACCATGGACGTCTCGAAGACTTGCCAAACATTGACCCGCCATCGTTGTATACATATTATCAAGAGGTCTTGTCCGATTGCCCGGTGGATTTTTATTGCGTGGGCAATGTATCGGTGGACGAAGTGCTGAAATTGCTTGAAAAAGAATTTTCGTCCATTGCGGACAAAACGCGGAAAACGGTACAGACAAAAACGGCTTCCCTTCCGGTGAAGGAAGAACGGATCGTGACCGAACGGATGAATGTCAAACAAGGGAAATTGAACATCGGTTGCCGGACCCAAACCACAATAAAAGATCCGGATTATCCGGCGCTCATGATGTATAACGGCATCCTGGGCGGCTTCCCGCATTCCAAGCTGTTCAGGAATGTGCGCGAAAAAGCGAGCCTCGCTTACTATTGTTCTTCCCGCTTGGAAAGTCATAAAGGCCTGTTGCTCATTCAATCCGGAATTGAAATCGCCAACTATGAGCAAGCGGTTCAAATCATCAAAGAGCAGTTGGAGGAAATCCGGCAAGGAAACATCAGCGATCAGGAACTGGAGCAGACCAAGGCGACGTTGTCCAACCAGCTGCGCTCTCAAATGGACCGGTCTTATGAGATGATCCATTTCCATTACCAATCCGTTTTGAACGGAAAAGATTTGCCGCTGGAAAAATTATTGGAGCAAGTGAATGCGGTGCAAAAGGAAGACGTGCAAAAAATAGCGGAGAAAATCCGGTTGGATGTGATTTATTTCTTACGGGACCGGGGAGGGAATGAACATGCAAAAAGTGGAGTTTGATCAAATTCAAGAATCTCTGTTCTTTGAAGAGCTTCCCAACGGCTTGAGTGTTTATGTCCTGCCGAAGAAGAATTTCTTTAAAACGTACGCCACGTTTACAACCAAATACGGATCCATTGACAACACCTTCCGGGTAAAGGGGAAAGAAAAGCATGAGGTTCCCGACGGAATTGCGCATTTCTTGGAACACAAAATGTTTGAGCAGGCATCGGGGGAAGACGTGTTCCAGGAGTTTTCGCGGCAGGGGGCTTCGGCCAATGCGTTTACCAGCTTTACCCGGACGGCCTATTTGTTTTCCAGCACGGGCCAGGTGGAAAAAAACCTGACAACCTTGCTTGATTTTGTGCAATCGCCGTATTTCACGGAGCAAAATGTGGAGAAAGAAAAAGGGATCATCGGACAGGAAATCCGGATGTACGATGACAACCCGGACTGGCGTGCTTATTTTGGCTTGTTTGAAGCCATGTATCACCATCACCCCGTTAAGATTGACATCGCCGGAACCGTGGAGTCCATATCGAAGATTACCAAAGACTTGTTGTACACTTGCTATGAAACTTTTTACCATCCGAACAACATGGTCCTGTTCATTGTCGGGCCGGTGGATCCGGAAGCCATCATCCAATTGGTGAAAGAAAACCAAAAGAACAAGTCTTTCCCGGATCAGCCGGAGATTGAACGCTTTTTCCCGGATGAGCCAGAGGAGGTGGCCGAAGCCTTCAAGGAAATCGAGTTGAGTGTCGGGATTTCCAAATGCATGTTTGGATTTAAAGAAAAAGCGGACAAGGTGAACGTAAAGGGAAACGAGTTTCTCAAACAAGAAATGGCCACCCAGATCGCTTTGGAAGCATTGCTCGGCCCGGCTTCCAACTTGTATCAATCCCTGTATGACGACGGATTGATTGATGACGGGTTCGGGACGGATTATACCCTAGACAGGGGATTCGGGTTGTCCATCATCGGAGGGGATACGCCGGATCCCGATCAGTTGCTTGAGCGGATCAAAAAGGAATTGCCACCCTTGATTGAAAAGGGATTGGACCAGGAAACTTTTGAACGGATTCGCAAAAAGAAGATCGGTGCGAATTTGCGCTATTTGAATTCTCCGGAATGGATTGCCAACCAATTTACCGGTTACCGGTTTCATGATGCCGATTTATTTAATCTGGTTCCGTGTTTGGAGGCTTTGACGTTAGAAGAGGTGAACCGCCGCATGCAGGAACACATGCAGTGGGACCGGTTTGCCGTTTCCATCGTGCGGCCGTCCCAAGATTAAAGTTACAAATCATAACCGTGAAAGCGTCCTTTTTCAAAAGGGCGCTTTTGTTTGAAAGGATGGAAAACATGCAAGCGTTAAAAGGAAAAACCGCCTGGATCATTGGAGGCAGCGGGGGAATCGGGCGGGACATTTGCATCGAACTGGCCGGATTGGGTGCAAACATTGCCGCCGGTTATCATTCCCGGCGGGAGGAAGTGCAAAAAGTTGTCATGGAATGCAAAGAAAAAGGGGTTCCGGCTTTTGCCTTTCCGGTGGATTTAAGGACCCGTTCTTCTGTCGATGAGGCTTACCGGCAAATTTGCATGTATTTGTCTCCGCCGGATATCGTGATTCATGCGGCGGGTACGTCCAAATGGGGATTGATTCAGGATTTTTCGGATCATGACTATGAATTGCTGATGGATGTTCATGTGCGGGGTGCTTTTTATCTGGCACAAATCGCGCTTCCCCGCATGATCCGCAAAAAAGATGGACGGATTATTTTGGTCTCTTCCATATGGGGGCAGACCGGCGGTGCGGGGGAAGTGTTGTATTCCGCCGCCAAGGGAGCGCAAATCAGCATGGCCAAGGCCCTGGCGAAAGAAGTGGCGCCAAGCGGCATTACGGTGAATGCTATTGCGCCCGGAGCCATTGACACTCCTTTGTTGGATCGGCAATTGCCCGGGGAAGAGAAGCATTCGTTGGCGGAGGACATTCCGGCCGGCCGTTTGGGAAGTCCGGAGGAAGTGGCATCGCTGGCCGCTTTTTTATGTCTTCCCCAAGCTTCCTATATCACCGGGCAAGTCTTTGCTGTTAATGGCGGTTGGTATACATAAAATGAAATCATTCGATTCATATTAGAAGTAGATCCATGTATCAGATAAAAGGAGGAAAAAGAAATGTCTGTTTTAGACAATTTTCAGGACTGGAAAGAGTTTTTGCATAACCGTGTGGACCAAGCCCGCCAGCTGGGGATGAGTGACCAGGATATCAAAGAATTGGCCCATCAACTCGGGGATTATCTGGCCAAGGATGTGGATCCGCAAAACACCCAGGAACGTGTTTTAAAAGATCTTTGGAGTGTGGCGGATGAAAACGAGCAACAAACGATCGCCAATTTAATGTTCAAAATGGTGAACGATGGAAACAGATGAATGTTTGTTTTTATTTTAAAACACCCAAAAACGGGTGTTTTATTTTTTTGATAAATTCAATTCTCAATGTAATACTTTTTTGGCACTTTGCGGTTATGCTATACTAATTAACATATTAATCTTTTGAGTTTGGCGTTTGAAACGGGAGAGGGTGGGGTGCTTTCATGGATCACCAGGAATGGTATCTGGAATATCAGATTCATAAAAATCGCCCTGGTTTAATGGGGGATTTGGCATCCATATTGGGAATGCTTGGCATCAATATCGTCACGATTAACGGGGTGGAGAAAAACCGGCGGGGTTTACTGCTCCGTTCTGATGATAAAGAAAAGATTGAGGCGTTAAGGGGAATTTTGACAAGAGTTGATAACATCACCATTACGGCTTTGCGCCCTCCTCAACTGGTGGACCGGATGGCGGTCAGACACGGCCGTTACCTGGAACGGTGTGTGGCCGATGAAAGAACGTATCGATTCACAAGGGAAGAGCTGGGGCTTTTGGTGGATTTCATGGCACATCTTTTTAAAAAAGAAGGGGCTCAGTTAATCGGTATCCGGGGCATGCCCAGAGTCGGGAAAACGGAGTCCGTGGTGGCTGCCAGTGTTTGTGCCAATAAGCGCTGGACATTTGTTTCGTCCACATTGTTAAAACAGACGGTCCGCAGCCAATTGGCCCAGGATGAACTTTCAGGCCAAAACGTGTATATTATTGACGGGATCGTTTCCACGCTCCGCTCGACCGAACGCCATGACATGCTGGTTCGGGAAATTTTGCGTATGGACGCAACCAAAGTAATTGAACATCCGGATGTGTTTATCAGGGAAACAGAGTATACATTGGATGATTTTGATTATATCATTGAACTCCGGAATCATCCGGATGAAGTGATTTCGTATGATCGATTGGATGAAAATCATGGATTTTAAACGTGGCAAAAGCTTTGCTGCAAGGGGAGGGATCAAAAATGTCGGGGATTGGAGACTATTTGAAACAGGTGCGGCAGCAACGGGGGTATTCTCTGGAAGAAGTAAACAGAATTACGAATATTCACATAAAATATTTGCATGCGCTTGAAAATGACCGTTTTGATCTGCTGCCAAGCCCTTTTTATGCCAAGGCTTTTTTGAGAACTTACGCGAAAAGCCTCGGCGTGGATACGAAACCATTGTTGGACCATTTTGACAAGTTGATGAAAATGCGTCCCAATCAGGAACAGTCCAAAGCCGTCACGCCCCCCAAACCGGCATCCCGGACAAAAATGTTGCCGAAATCAAGCGGGAATTTTCTTCCTCCGAAAGGACAAGCGCAAAGTCCGAGACCAAGCGGGGTGTTGAGCGGGAGTCCGTTCCGCCAGCCAAACAACCCGGAGCCTGAACCGGCCAAAAAATCTTTGTCTTCCGCGCCGAATCCCGGATTGAATTTTCGTCCGGCAAACCAGCAGAATCAGCTTCCGCCGGTTCCGGAAGTAACGAAATCCCCGGAACCACCGGTTGAGCCGCAACCAATGAAACACCCGGAACCGTCAACCCAAACACCCGTACCTCAAACGACGCTGTCTCTGCCTCCGCTTGAAGCCACGCAACAGCATCTGTTAACACCGCGCCGGGTTGCCATGGAATTGAAAAAACAACAAATGAAAGAGAAAAAAGAAAATGTCAAAAAGAAAAAACATACGATGATTGCCATGGTTGTCGGTGCTTTGTTATTGTTAAGCGGAGGAGTGTATGTTTATCTCCATGGTCATCAGACTTCCCAGCCCGAAAAACGCAATGCAGCGGAATATCCTGCCGGAAACGGGCAAATGGTGGATGTCAATGCCGCTTCGCAAAATTTGCCCACTTTGGAGGAAGGGGAAGAATCCTCGGATCCTTATGCCGGACAAGAGTATTTGATCAAAAATGTCGATAAATTAAATGTTGTGTTAAAAGGGAGAAGTGGGGAATCAACGGTTTTGTATGCCCCTACTCCGAAGGATCAGCCCAAAAAATTGACATTGCGTGTGGGGCAAGAAGTCACACTGGATACAGCCGGAAAAAACTATGTTTGGTTCCGATTGGGAACGCCATCTAATGTAGAAATCCTTGTGAATGATCAACAAATTAATACAGATGCGCAAGATGCTGAAAAAAGTTATGTGGTGAAAGTTCAGTAAGCCGTGAAGTTAGTTGGGGGAGGGTGCTTCGTTGTCCGTGGAAATCGGGAGGAGACTGAGAAGGGCGCGTGAGGCGTCGGGCTTGACTTTGGAAGAAGTGGAGAGACGGACCAGAATTTCCAAAAGGGATTTGTTGGCGATGGAACAGGGGCGGTTTCATTTTCACCCCAATCCCGCGTTTGTTCGTTCCTGTGTCCGGGCATATGCAACGGTTGTGGGAGAAAATCCGCAACCGATGTTAAATTTGTTGCAACAGGCACAAGAAAGGACGACGCGCAGGGAACCAACAGTCCGAAGAAATCTTCCGGACGGGCAGGGAAATGGTGGAGAGCTGTCCCGGTCCCGCCGCCGGTCAAACGGATACGGACGTTCTCAATCCCATAAGAAACCGGATTATCCTCAGCAGGATCATCCCCCTCAAAGGCAGGTTTACCGACGATCGGAGAGAGAGGGGATGGAGACGGAATCCGGGGAAGGTGTATACAGCCGCCGGAGCCGCCGGCAGGCTTCCAAAAAGAAAACGTCCGTTTTTGGTAAAATCTACTCGTATTTTTTATTATCGGGTGCAATTTTATTGGTGATTGCAACCATATGTTTTTTATGGTTCCGATGGAGCAATGCCTGATCGGAGAGGCGGTTTCCCGGTTTGGACAGGCCGGGGGACGCCGGTTTTTAAATATAAGAATATACTGAATTTTAACGAGGGGTTTTTCACCGGAAAAATTGACGGTTTTTAAAAAAAAGTTTTAATTTCGGACGGCAACAAGAGGGACTGTAGAAGAAGGCGTTTTTGACACATTTCGGAGACCATGGTATGCTTGAAACAAATTGTTGTTTCTATACAGCGCCGTCAAGATCATTGTTTTTTTATATATATTTACAACAACTCTCAGGCTCTTTTTTGTTTATGGGATGTTGTTTCAAAGAAAAAGAATAGATTTTGATTTCGGTAAAGAGTATGATAGAAGACGGTGTTTCAACTAACGCTAAAAATGTTTACAACCAATCGAAAAGTGCTGGGAAAACAGCAAACACCATGAAAAACCGATAATTTAACGGGTTGCTGAGCAGCATGTCTTTTGTTGTCAAAAGCGTATTGTTCAAACAGCCTTTCAGGAGGTGGCTGGGGTGTCTGTCGAAATTGGCTCATATCTTCGACAAGCCCGAGAAGCGATTGGAATGTCCCTCGAAGAACTGCAAGAAAAAACAAGGATCCAAAAATCATTTTTAATTGCGATCGAAAATGGTGAATTCGATAAATTGCCGAGCCCTTTTTATGTGCGGACATATCTGCGTTCTTATGCCAATTGTGTAAAAATCGAACCGCATCATATTTTGCGTCAATACCGAAAGATGGAACAAGCGGAGAGATTAACCGGGGTACATAAAGCGGTCAATCCGAATGATTTTAATCAAAATCAGCAGACGCAAAAGTTACCGCCGCTTGAAAAATACCAGCAAACGGGCAAAATGCCTGCCGTCAATCCAAGTCCATCGACGCAAAACACAATGCGCATGAGAACGTTGCCGATGAATAACCAGACCAAGGGACGGATCGATGCCCAAACGGCGCTGACGGCTTCCAAGACCATGCCCAACCGGGTGAGCGATCAGGTGAAAGCGCGCCGGGACATGGGTAATCAGCAAGCGGGCATTCTGAATAAGGATAAAGATCAGACGGCTCAGGGAAACGTTGGCGGAATCAATCCGAAAAAAGAATTGTATCCGACGGTTTCACGGAAAAACCAGCACGGACAAACCAGACCGAACCAAAGTTTGTTGCTTTCAAACACCGGGACGGTGCCGCGTGTGTCCGGGCCGTTGAACCGGGTTTCGGATCAGGCAACCCGAAATTCCGGACCGGTGCCCCGGGTTACGGGTGCGATGAAAAAGCTGGATTTAAACAATGTCCCAATTCCATCGGTTTTGCCGGACGACTTGGAAAACGGATCTCAGAAAAAAGAAGGGGAGCGTCAACGGTTGAGTCGCGTTGAACGCTTAAGTCGCAGTGCGTCCAAAAGCCGGAAATCTTTCAAAATATCCGGTTTGAAGCTTAAAAGCCCCATTGCGATGGCGGTTGCCGGTTTAATCTTGTGTGTCCCCCTGGGATGGGCGATTTTGTCGTTTACAGGGGATGATTCCCAACCGGATTCCAACCCGGCTTCACAAACCGCCAATGACCAAACGCTTGAGATTCCGCCTGAGAAAAAAGGAAAACTGCAATTGGCGCAGCAGGATGTGAATGTGGTGCACTATCGTCTCCAGGATACCGATCAAATCGTGGTGATGGTTCATGCTGCCGGTGGAAAATCGCGGGTTGAGCTCAGAACCGATCTTCCTTCCGAGGGCGGAAGTGATCATTTGATTAAAGATGTGACGTTGCCCAAGGACGAAAAATGGAGCTACAAATATACATATGAAGGCAATCGGGACTTTTATGTCGGATTGAGCGCTCCGGAAGATGCGATTGTTTACGTCAACGGACAAGTCGTGAAATCGGCACGGTATATTCACGTCCGCAGAGTCGATTGATTTTTTTTTTTGGAAGCAAGTGCTTGCCCGGCACTTGCTTTTTTGACGTTCATATTCTTCTGGGCTATACTGTAAAAGTTAGAATTGAACAGAACAATCAACGGGAGGGACACGGCAGATGTCCAAGCCTTTTGAAGAATTAAAAAAGGCCAGGGAACAGAAGCAGCTGAGTTTGGAAGAAGCAGCGGAAAAAACCCGGATTGATGAAAAATATCTTGCAGCTTTGGAAAATGCTGAATTTTCCGGGCTTCCGGATCCTCTTTATGTGAGGGGTTTTATCCGCAGGTATTCGAAACTGTTGGGAATCGATGCAAAGCCGATGTTGGATGCATATAAAAAGTGGGAACAAGAAGAGCGTCCCGAAGAGTCCGGGCCGGTCACCACGCTTCCGTCCCGAAGCAGCCGGACACAAGAATCATCCGAACCGAAAAAGAAATGGATTGCCAAGTGGCCGAACGCTCTCTTGGTTTCTTCCATCGCCGTTTTATTGATCGCAGCCGGTGTGGTTACATGGCTGGTTCTGGATCAAGACTCATCCGACGCAGAAACGGTTCAGCTGGAGGAACCGCAAACGGAAACGGCGGATCCGGAGGCCAAACCGGAACCCAAAGAACGTCCGCAGGTGGAATTGATTCAACCGGCTGAAAAAGGAGAACAAGGCGATGTGTACGGTGTTTCAAAAGCCGAAAAAGTGACGGTGAAAGTGGTGGCGGACAAATCAACTGAAATCCAGATCCGTTCGGGGGGACCCACGGGGAAAGTGATTGCTTCCAAAGCATTGTCCCCGAACCAGGCGGAAGAATTTTCCGACCCGGAGTGGATTTCTTTGCGGGTCGATCATCCCCATTATGTCAAACTCTATGTCAACGGGGTTTATATTGACACCTCTTCACAGCAGCAAGTTTCATTGTACCAATTCAAAAAGAAAGAAAGCACGGATGATGAATAAAAGCGGTTCAGCAGAGAGGATCCCTCTTCCTCTCTGCTTTTTTTGACACGCGTCAGATGCTTGGTTTATACTTACGAAAGAATTAAACGAGGAAGGTATGGGTTTAATATTAAAGGATGGATGCGTTTATACTAGAAAAATAAAGTTGATTGACAAAAAAACGAAAAACGGCTTTACAAGATGGTGCCAACCAGGGCATCTTCAAAAGCGACGGCATTTCCTTCTTGGAAAATGCGAAACGCCGGAAATGAATGGTGCGGTGTTTCCGCTGCTCCCGTGCGTGCGGGGCTTTGAAAATCCTCTTCCTAAATTCATGATGATTGTGTGAATGCGGATGAAATTTTTACATCAAAATGGTTATTGCCAGGGAGGTTGGATAGGATGAACCTGGCCAACAAGATTACATTGGCTCGTATTTTTTTAGTCCCCATTGTGATGATCTTTTTGTTGTTTCGCTTTGACATCGGAGAAATCCATGTGGGAGCGGCGAAGTTGACCGCGGGCGAATTGATCGCCACGTTTATTTTTATTTTGGCTGCCGTCACGGACGGGGTGGACGGATACATAGCAAGACGGAAAAAAATGGTGACCAATTTGGGGAAATTTCTTGATCCGTTGGCTGATAAACTGTTGGTGACAACAGCCTTGATCTCATTGGTGGAGTTGCAACGACTGGAGGCGTGGATGGCCATTGTGATCATCAGCCGGGAATTTGCCGTGACCGGCCTCCGGATGATTGCGGTCACCGAAGGCCATGTGATTGCAGCCAGTCCATTGGGAAAACTGAAGACCATCATACAAATTGTGGCGATTGTTTCATTGATGCTCAACAATGTTCCGTTCTCAACCATTTCATTTCCGTTTTCCATGATCGCCACCTGGCTGGCCGTGATCATCACGGTCATTTCCGGAGTGGATTACTTTTACAAAAACCGCAAGATCGTTTCATTGACCAATACACAGTAGTGTCGTCCGGGATTTGGCTAAAGAGAAAGGAAAGATAAAATGCAATCAGAAATCATTACGGTTGGTTCTGAGTTGTTGGCGGGAGAAACGGTGGATTTGCATTCTTCTTATTTGTCCCGGGAACTCCATTCATTGGGGATTGATGTTCAATTTCACACATCGGTCGGGGATGATGCACAAAAGCTGCATGACGTGATTTCACTCGCACGCACGCGATCGCAGTTGATTTTCCTCTGTGGCGGGCTGGGGCCCACCATGGATGACTTGACCAAAGAAACCGTTTCGGAAGTGACGGGGGTTCCGTTGGTTGAGGATCCGGAGGTGAAACGCCGTCTGGAGAAGTATTTTGAAGGACGGATGAAGGTGGTCCCCGCAAATAATTACAAGCAGACTTATGTTTTTTCCGGCGGTGTTGTTTTCCAAAACCATCATGGAACGGCTCCCGGTTTGGCTCTGCAGGTCGACGGGATCACTTATGTTCTGTTGCCGGGTCCTCCGGGGGAATTGGTGCCGATGTTTGAAGAGCAGGTGCGTCCTTTCCTCATGCGCGAAGTGATTCAGGAGGGGCAGGTGATTCTTGCGGAATCCCTTTCTTTTTTTGGATTGGGCGAATCGCTTTTGGAGGAGAAGATTGCCGATTTGATCCGGCAACAGGACAATCCCGTTTTGGCCACCTATGCCAAAGAATCGGGGGTTGTTTTGAGAATCACGGCTTGGGCGCCGGGCGAAGCGGAGGCACGTCAGCTGATTGACCGGCAAAAAGCGAAAGTTTTAACCCGGATCGGGGAATATTGTTACAGCGAACAGGGTGAGACGCTGGAAGAAGTGGTGGTGGCCGGGTTGATTCGCCATCAGAAAACAGTGGCGGTGGCGGAAAGTTGCACCGGCGGAAAACTGGCTCATCTTTTGACTTCCGTGTCGGGAAGCAGCCGGGCGTTTAAAGGCGGATGGGTGACCTATACCAATGAAGTCAAAGAAAAAATCGCCTCGGTGCCGGCGGAATGTTTGAATCAATATGGTGCAATCAGTGCACAAACGGCAGAAATCATGGCCCAAAACACGTTGGAGCGTTTTGATTCGGATTTCGCACTCGGGGTCACAGGGGTCGCCGGGCCGGATCCGGCGGAAGCAAAACCGGTGGGAACGGTATTTATCGGATTGGCCGAAAAAGGACAGCCGGTGCGGGTTTATCAGTTCGCTTTGAAAGGATCGCGCAGGCGTATACAACTGTTGGCGGCGAATTATGCCTTATTTGTTCTTGAGCAATGTTTAAAGAAAGGTGAAACAACAAGATGAATCGTTTTGATCAGTTTCAATTAAGCAATGAAATATTAAACGGAATACACGATATCGGATTTGAAGAACCTTCTCCGATACAAGCGGAGTGTATTCCGGCCGTGCTCCGGGGGGAAGATGTGATCGGGCAGGCGCAGACCGGAACCGGAAAAACCGCGGCGTTCGGCATTCCCATTTTGGAGCGGGTGAATCCGAAACAGCCGCATGTCCAAAGCATCATTTTGGCCCCGACCCGTGAGTTGGCGATTCAAGTGTCGGAAGAATTGCGGAAAATCGGGCGCAAAAAACGGGTGAAAACATTGCCGGTGTATGGCGGGCAATCGATCGGGCGCCAAATCAAGGCGCTGCAACAAGGGGTTCATGTGGTGATCGGGACTCCCGGCCGGCTGTTGGACCATTTGAGACGCGGCACGATCGATCCTTCCCATGTTGAAACCGTGGTGTTGGATGAAGCGGATGAAATGCTGGACATGGGATTTATTGATGACATTGAGCAAGTGTTCCGCTTCATGCCGAAACATCATCAATTGTTGCTTTTTTCGGCCACCATGCCTTCGGCCATTCGCCAATTGGCGAAAAAGTACATGCGCAAGCCGAAGTATATCACCGTCAACCGGGGCGAAGTGACGGTGCCGGTGATCCGCCAGATTTATTATCGCGTGTTGGACAGTTACAAGGTGGATGCCCTGTGCCGGATTCTTGACAGTGAAGAAGTGGATTTGGCGATTATTTTTTGCCGTACGAAAAAAGGCGTGGATGAATTGACGGATTCCTTGCAGGGACGAGGCTATCTGGCCGGCGGACTGCACGGGGATCTGGCCCAGCAACAGCGGGATCGCGTGATGCAATCCTTCCGCAGCGGGGAAATTGAACTTTTGGTGGCAACCGATGTGGCCGCCCGCGGCATTGATGTGGGGAGTGTTTCCCATGTGATCAATTACGACATCCCTCAAGACGTGGAAAGTTACGTGCACCGGATTGGAAGAACGGGCAGAGCCGGACGCAAAGGGGTGGCTCTCACGCTGGTCACCCCCAGGGAAATGAAACAGCTTCGCACGATTGAACAGGAAACGGGCACCAAATTGAAGGCCCGGGAACTGCCCACGTTGGAGGAGGTCGCGGCGAAGCAGCAAGAAAGCTGGATAGCGCAGATTGAAAATACCATCCGTTCCGATGTGGACATGACATTGTTTGAGGAAATGACCGGGGAATTGTCGAAGAAATTTTCCATGGAAAAAGTGGTGGCGGCTTCGTTGTATCTCGCTTTTTCCGACCGGTTTGTCAAGAGCATTGACAACACTTACCATTTCGGGGAAACGGGGGCATCGCCAGGAATGGTCCGCTTTTTTATCAATGTGGGGCGAAATGCCAACCTGCATCCGCAGGAGTTGGCCAAAGCGATTTCGGAGAATGCGGGGATCAGCTTGAGGCAAGTGGGGAAAATCAACATCTACGACCGCTTTTCTTTTGTGGAGGTTCCAGAGGATGTTGCGCCTTTTGTTTATGAGGCGTTCCGGCAATCCAAAATCAACGGTTCCCGGGTCAATATGGAGCCGGCCCGTCCCCGCAACCGTTCATAAAGGGCAAAAAACCCAAAAGAGCTCTTACTTTGTGGATCAACCACAAAGTAAGAGCTCTTTTTTCAGTCGGGAATGCCCAAAGCGATTTTGGCATATCGGGACATCCGGCTTTTGTCCCACGGGGGGTTCCAAACGATTTCCACGTCCACTTTATTGACGCCTTCCACATTTTTCAACGCTTGTTCCACTTCCTGCTGGATGATGCCGGCCAAAGGGCAACCCATGGCTGTCAACGTCATGGTGACCTTGACATTTCCTTCATCATCGATATCTATGTCATAGACCAGCCCCAGATTGACAATGTCAATATTTAACTCCGGGTCATTGACTTGTTCCAGTTGTTCCATGATTTGTTCTTTCATCTGTTCCTTTTGCAATGCGCCTCACTCCTTTTTTGCTTCTACCTATAACGTAACACACTTTTTTTTGCATTACACGAGGATTTCCAAAAAAAGAGTCGAATAAATGTTCGTAAATGATCTTGGCAGATGAAGAAAAACACGATATGATGTTCATATGATTTAATGGGGAAAAAGAGGAGGAACGATACATGTCCGATCGACGACAAGCGTTGGAAATGGCATTGAGACAGATAGAAAAGCAGTTTGGCAAAGGCTCGATCATGAAAATGGGAGACCAGGCCACCCGCCAGGTGGAGACCATTTCAAGCGGCGCGCTGGCCCTTGACATTGCTTTGGGAGTGGGCGGATATCCCCGCGGAAGGATCATTGAAATTTACGGGCCGGAATCTTCCGGGAAAACCACGGTGGCTTTGCACGCCATTGCAGAGGTGCAAAAAAAAGGCGGACAAGCCGCGTTTATTGATGCAGAGCATGCACTTGACCCCGTATATGCCGAAAAACTGGGAGTGAACATTTCGGAGTTGTTGTTGTCACAGCCGGATACCGGGGAGCAAGCGTTGGAAATTGCCGAAGCCCTCGTTCGCAGCGGTGCCGTCGATATCATTGTGGTTGACTCGGTGGCGGCACTGGTGCCCAAAGCGGAAATTGAAGGGGAAATGGGAGACTCTCACGTCGGGTTGCAAGCCCGGTTGATGTCTCAGGCACTGCGCAAGCTTTCCGGAGCGATCAATAAATCCAAAACCATTGCCATCTTTATTAACCAAATCCGTGAAAAAGTGGGCGTCATGTTTGGAAACCCGGAAACGACACCGGGCGGACGCGCACTGAAATTCTACTCCAGTGTACGCCTGGAAGTCCGCCGCGCTGAAACCATCAAGCAAGGAAGCGACATGGTCGGAAACCGCACGAGAATCAAAGTGGTGAAAAACAAAGTGGCACCTCCTTTTAAACAGGCGGATGTCGACATCATGTTCGGAGAGGGCATCTCCAAAGAAGGAAGTGTCTTGGACATCGCTTCGGAACTGGACATTGTCAATAAGAGCGGGGCATGGTATTCCTTCGAAGGAGACCGCTTGGGGCAAGGACGCGAAAATGCCAAACAATTTTTGAAAGAGCATGCGGATGTCTGCCTCAATATCGAAAACCGGATCCGGTCCCATTACAACTTGCCCGTCATTCAGGCAAGTGAACCGGAAGAGGCAAAAGACACAAAAAAGAAGAAAAGTAAAGCGGACAAAACGGAAGAACTTGCTTGAAAACCGGTTCTGAAGAATGGATGAGGCAAGTGGGAGAGAGAAAAAAAATCACCCGGATTGAAAAGCCGTATCTTAACAAGCCGCGATATCATATCTATGTGGATGGAGAGTTTCTCCTCTCCATCCATGAAGATATTTTGGTGAAGTACCGGCTTCACAAAGGCATGGAGCTGGATGAAAGCGAAATTCATGAGTGGCTTGAGGCGGAAGAATACAACAAGGTGAAGCAAAAGGCTTTGCATTATTTATCTTACAAGCCCCGTACAACGCATGAAGTCAAAATGTATTTGGGCCGCAAAGGTTTTGAACGCGAAACCGTCGAACAAGTCATTTTGGAGTTGGAAAAAAGCGGATTTTTAAATGACTTGGAATTTGCCAAAGCCTGGGTGAATGAGCGGCAACATCATAAAGGATACGGAAAAAGGCGTTTGAAACAGGAGCTGTTGAACAAAGGAATGGACCCGGCGGTCGTTGATGAGGCATTGATTCATATCGGGGAGGAAGATGAGCGCCAGTTGGCAATATCCATTGCTGAAAGGCGCTATTTGCGTTTATGCCACGAGTCTTGGCCCCGGATCGAACGCCGCCTGGGCAATTACCTCGTCAGGCAGGGATTTTCCATGGGCGTGGTGTATGAGGTGTTGAATCTTTTCCGGGCCCGTCACGAAGAAGAGAAGGGGAATGGTCCATGAAAATATTGTATATGACCGATACACATATCCGTGGGACATCACCACGAAGCCGTACCGACGATTTCCAGCAGGCGATTCGCGAAAAGATTGAAGAAGTGATTGACATTGCCGAAAGAGAGCGGGTCGATCTGGTCTTGCACGGCGGGGATTTGTTTGACCGCCCCAATCTTTCCCCCGCCATTGTCCGGGAATTCGCCGGGCTGTTTCGCCGTTTTCCGGCCCCCATTTTCGCGGTTGCGGGGAATCATGATATTTACGGACATAATCCCGCAACGGTGGAGCGGACGATGCTGGGGTTGCTCGATGCCTTCGGTGCCATCCGGTTGGTGAACGAAGGGGAACGGATCAAATGGGAGAAAGACGGCATCACCGTTCAGATCAGCGCCCAACCTTTCCATTATGATTTGGACAAACGAAAGCGGCATCTCGATTATACAGTGCCAAATGAATGCGGAGCCGAATATTGCGTGCATATGGTTCACGGCATGTTGGTGGACAGGGCTCTTCCGGACAGCATCCCGCACACGGCGGTTCACGAAGTCTGGAGCGATTCCGTCGATGTTCTGTTGACTGGGCATTATCATGCAGGCTTTCCCGTACAGCACCGGAATGGAAAATATATTGTCAATCCCGGCGCGTTGGCCCGTATCAACAACCACCGGTCGGAAATCAAACGGATGCCCCAAGTCGCCCTTCTGGAATTGAACAAAGAGGGCATCGATGTCCGGCTGGTTCCGCTTCAATGCGCCAAGCGGGGGGAAGAAATTTTGGACCGGTCCTACATTGAAAAAGAAGAATTCCGCGAAAAACAGCTGGGCGAATTCATCCGTGAAGTCAAATCGGTGACCGATTTTCAATCCATCAACATCATGGATATCCTCTTTGAAATGTCCAAGTTGCATGACATCGGGGATGTTGTCACTTTGGAAGCCCAGCGGAGGATTGCGATTGAACAAGAAGCAGAAAGGGAGAGCAGCCATTGAATGGTTTTAAAAAGCTCGTGATTGAAAATTTCCAGTCCCATCAGTATACCGAAATTGATTTCACCGACGGGCTCAATGTGTTTGTGGGACCGTCTGACAGCGGGAAAAGCGCGATTTTGCGCGCATTGAAGTGGGTGTTGTTTAACACTCCGCGGGGAACGGATTTTATCCGGACCGGGGCCAAAGAGTGCCGCGTTCGTCTGGTGTTGACGGACGGGACGGCGATCGAGAGAATCCGCAGCCGTTCGGTCAACCGGTATGTGCTCAATACACCGGACGGAAAGGAACAAATCTTTGAAGGATTTGGAAACTCCGTGCCGGAGGAAATCTCCGACGTGCACCGGATCGTTCCGGTTGAGCTGGACCAAAAAGAGTTGCTGTTGAATTTCGGCACACAATTGGAGAGCCCTTTTTTGCTTTTTGAATCGAATCAAAACAAAGCGAAAACCATCGGCCGGATCAGCGGTGCCCATTTAATCGACAAAGCCTTGAAAAAAACGAGCACCGACCGGCTGGCTTTAAGTGGAACGATCCGCCAGCTGGAACAGGAAAAAGAGCGGTTGGATGAAAAATTAAAGCCGTATGAGAACCTGCAGCAGTTGGAAGCCGATTTTCAAGTGGCGGAACGGGCTTTTCTTGACGTGCAAGAAAAAAAGCGGCTGCAAGCGTCTTTGGAAGCAAAACTGGAAAGCTTGATCGAGATACGGCGGGGAAAAGAAGTGTGGCAGGAAAAAGTTGCGGCATTAAAACGGTTGCCGGATGCCGAACAGCAGCTGCTTCACTTGGAGTATCAAAAAGCGATGCTTTCCCGGTTGATTCGTTTAAACGAAAAGTGGATCCGGCTTCAACAGGAAAAAGAGCGCACGCTGGAAACGGTCAAGCTGGGTGAACACATTCCGGAAGCGGAAAGGCGGCTTGCCGGTTTGTCTAGGAGCCAAAAAAGGTTTGAGCAACTGCTCCGGCTCCAAAACAAGCGGAATCAGATCAAAGAGAAAAAAGCCGGGCTTGAAACCTTTGTGCTCAGGCTTAAAAACGTGCCGCAAGGCATGCGGGAGTTAGAGAAACTGGCCGAAAAGCACGAGCGTTTCAAAGTGCTCCAGCCTTTAAAACGACGGAAAGAAAAGAATGCAAGGGAACTTAAGTACTGGCGGCAGGTGCAAAAGCAAAACCAAGAAGCCTTAGAGGTGTTGGAAAAAGTGGTGCCGAACATAGAAGAGAAAGAAGCGCTGATGAAGAAACTGGACGATTTGCACCGAAGCCGGCTGGATTGCCAAAAACGGATTGCCACCGGAAAAAAATACATCCAGGAAAAAGAACAGGAAATTTTGCGTTGCACCCGGGAATGGATCCGGCTGTTAAAAAAGATCGGAAAGTGTCCGACCTGCGGAACGCCCATCGGCCACTCTGTTTTGCAACATATCATGGAAGAGTATCAAGGAGGAATTTCTCGTGCAGCAGCTGGAAGAGAAGATTAAACAACTCAAAGCGCAACTGGAAGATGCCAAAAGCAAATTGACGGTTGCCAAAGCCCGTCTGGAAGATTTGGAAAAGCAGGAAGAGGAGATTGTCAAAGAAGCCAGGGAGCTGGGAGTGGAACCGGAAGATTTGCCCGGGGAGATTGAGCGGCTGGAAAAAGAGATTGAGGAAGGCATTGAAAAGGCATGGAGCTATTTGCCCAAGGAGTTGAGAGAATGAGATGAAAGACACATCACTTGAGGAATTGCAACAGGCTTTGAAAGAAGCAAGAGATGAACTGTTGCGCCGAAAAGCGGAAAAAGAGCTGCATTTGCGTCAGTTGAATGAGACGGAAACCCGGTTGAAAGAAAGCGTGGAGCACCGTGATCTGTTGGACCGGGTGCGTCTTTTGTTCCAACTGTCATCAGACTATGCCCGCCAACAGGCAAAGCAACAATTGGAAACGCTGGTGACCAATGCCTTGAAGTATGTCTTCGGTTCGACGTTTCGTTTTCAAATTGAGCTCTCGGATCATGGCGGAAATCCGACGGCGGAATTTTATGTGGTTTCGAAGTGGGATGAACAGGAAGTGAAAAACCGTCCACAGGATGCCCGCGGCGGCGGTGTCGTCGACATTGTCTCATTGGCGCTCAGGATTGCGCTCATGGAAACGATCAAGCCCCGTGTCCAAGGGCCGATTATTTTGGATGAACCCGGAAAACATGTGAGTGAAGATTATATCGTCCCCATGATTGAGTTTTTAAAATCGGTCGGGGAAACGTTCGGCCGGCAAATCATTCTTGTCACGCACAATACGCATCTGACGGAATCGGCCGATTCGGCCTATTACGTGCGGTTGTCATCCGGTAAGTCGGAAGTGGAGCGCAGCCGGAATCTTGACAACAATACTGTATAAATAATACAATTTAGTTGTATCTTTGCCTTTTTTAACGGTGGAAGATACCTTCTGACGAAGTAAGTTTGGAATAGAAACCTGAAGGAATTTGAAAATGTTTGCTGGTTTTCCAGCCTTGACCTGGTATAGCAAAAAATGAGTGAGGACGAGCGTGAGAAGACCGAGTTCATCGGTCTTATTTTGTAAACAGAAACAGAGGAGGTGAGCGGTGAATCCAATGGTGTTTAACGGATTGGACATCGTTCTGATGTTTGCCTCTCTCGGTGTCGGAAGCGGTGCGGGATATTGGATACGCAAAATGACGGCGGAAGCCCGGATCGGCAGTGCGGAGAAGGAAGCTGCCGAGATTCTGGAAAAGGCGAACAAAGAAGCCGAAGCCAACAAAAAAGAAAAAATCTTTGAAGCCAGGGATGAAATCCATCAATTGCGCACCGAAGCGGAAAAAGAGATTCGTGAGCAGAGGAATGAATTACAGCGGATTGAGCGTCGTCTTGACCAAAAAGAAGAAACACTGGAACGAAAACAATTAAATTTGGAATCGCGTGAGGAAGAACTGGCCAAACGCGAGAAATATATTGATGAACGTCAGCGGGAGATTGACCGGCTATACCAGGAACAGCTCAAAGAGATGGAACGGATTTCCGGACTTACCACGGAAGAAGCGCGTCAGTTGATTCTGGCTAAAGTCGAGAATGAGATGAAACACGAAACGGCTCAAATCATGAAAGAGATGGAACAACAGGCGATTGATGAAGGAGACCGGCGTGCACGGAAAATCTTGTCTTTAGCCATTCAACGTTGCGCGGCGGATCATGTTGCGGAAACGACCGTATCCGTAGTGAATTTACCCAATGATGAGATGAAAGGCCGCATTATCGGGCGTGAAGGCCGGAATATCCGGACGTTGGAAACGTTGACGGGAATTGACCTGATCATTGATGATACGCCGGAAGCCGTGATTTTGTCCGGATTTGACCCCATTCGCCGTGAAGTGGCGCGGGTGGCGTTGGAAAAACTGGTGGCTGACGGAAGGATTCACCCGGCCCGGATTGAAGAGATGGTGGAAAAATCCCGCCGGGATGTGGACGAACGGATTCGTGAATATGGAGAGCAGGCCACCTTTGAAACCGGCGTGCACGGATTGCATCCGGATCTGATTAAGATCATCGGCAGGTTAAAATTCCGTACCAGTTATGGGCAAAACGTTTTGAAGCACTCCATGGAGGTTGCGCATCTGGCTGGTTTGTTGGCCGCGGAATTGGGTGAAGACATTCATCTGGCGAAACGGGCCGGCTTGCTTCATGACATCGGAAAAGCCATTGACCACGAAATCGAGGGTTCCCATGTGGATATTGGCGTCGAATTGGCGAAAAAATATAAAGAACATCCCGTGGTGATCAACAGCATTGCTTCTCATCACGGAGACTGTGAAGCGACGAGTGTGATTGCTGTCTTGGTCAGAGCGGCGGATGCGCTTTCGGCGGCCAGACCCGGAGCCCGGCGGGAAACATTGGAGGCATATATCAAGCGCTTGGAGAAATTGGAAGAGATTTGCGATTCCTTCGACGGCGTGGAAAAATCATATGCAATTCAAGCAGGGCGTGAATGTCGGATCATCGTGAAGCCGGATGAAGTCGATGATGCGGAAGCCGTCCGTTTATCCCGAGAAATTACCAAACAGATCGAAAGTCAATTGGATTACCCCGGCCATATTAAGGTGACGGTGATTCGAGAGACAAGATCGGTCGAATATGCGAAATAAAGTGGCCTTGCGGCCGCTTTATTTTTTCAAGCATCTTAGCGGAGTTGGAGGCTATTTGTACTGATGAGAATTTTAATGATCGGTGATATTGTTGGACAAGTGGGGCGACAAGCGGTCGCCAATTATTTGCCTAAATTAAACCAAATGTACTCGCCCGATGTGATTGTGGCAAACGGGGAAAATGCCGCGGGCAACGGACGGGGAATTCAACGGGACATCCTGAGGGAACTGATGGATCTGGGAATCCATTGTGTCACATTGGGAAATCATGCTTGGGCGCAGTCCGAGGTGTTTGATTTTATCGATGAGGAGCCGTTTTTAATCCGTCCCGCCAATTATCCGCCGGGGACGCCCGGGAAAGGCCATGTTTTACTGACGGTAAAAGGCGTAAAAGTGGTTGTGGTCAATCTGATGGGGAGAGCGTATATGGGACCATTGGATTGTCCGTTCAGAAAAATGGATGAAATTTTGAGCCGGATTTCAGGAACCCCGTATATATTGGTCGATTTTCATGCGGAAACCACGTCGGAAAAACAAGCATTGGCATGTTACCTGGATGGGAAAGTTTCCGCGGTGATCGGGACGCACACGCATGTGCAAACGGCAGATGAACGAATTTTGCCAAAAGGAACGGGTTATTTGACGGATGTGGGGATGGTCGGCCCGTATGACAGCGTGATCGGGATGAAAAAAGAGGAAGTCGTGAAGCGGTTTATCACACAAATGCCTGTTCGCTTTTCTGTTGCAAAAGGAAGAGAACAATTTAATGCTGTATTGATCGATTTGGACAAACAAACCAAAAAAGCCACGAAATTAACGAGGATTCGTATTGATGCGGAGCATCCGCTTATCGGTTGAAATAGAATAAATTTATTTATGATTTTTTACTCCTATCTATGCAAATTGGAAGGAATTTTTACTTGCGAGTCGAATAGGATAGTAGTGGATTCACTCCAACCATCGAGGAGGTACCTACATGGAAGTATTAAAAGTTTCAGCAAAATCTAATCCGAATTCTGTTGCTGGTGCACTCGCCGGAGTCTTGCGTGAACGTGGTCAAGCAGAGATGCAAGCAATTGGCGCAGGGGCGCTGAATCAAGCTGTAAAAGCGGTTGCGATTGCACGAGGATTCGTGGCGCCTAGTGGGATTGATCTGATTTGTATTCCTGCTTTTACCGATATTATGATCGATGGTGAAGAACGGACCGCGATCAAACTGATTGTGGAACCTCGTTAGGGAAATTGGTCGATATATTCAAAAAAACCTGTTTATTTATATAGACAGGTTTTTTTAAATGGAAGGGTGAACAAGATGCAATGGATGGATGGGCATTGCGATGTGTTAAGCAAAATGTGGCGAAATTTGAAAGACCATTCTTTTTATGACCCCCGCTCCCGGTTGGACAGTTCTTATCACCATCTGAAAGAAGCCTTGGTGGCTTTGCAGGTTTTTGCGATATGGGTACCTGATTCGGTACCCAGAGAAAACCGGTTGTCAGTCGCATTGAAACAAGTGGATTTGTTTTATGAACAGATTATCAGGAAAGAAGAAGCGGTTCATTTTGTCGGTTCGAAACAAGCGTTAAAAAAATGTGCCCCGGATCGGATGGGGGCACTGTTGGCGATGGAAGGGGCCGACGCCTTGCATGGAGAACTTGCCTTCCTGCGGCTGTTTTTCCGCCTCGGTGTGCGCCAGTTGGGGTTGACTTGGAACCATGCCAATGAAGTGGCGGACGGCATTGAAGAGGAACGGAACGGAGGATTGACGAACTTCGGAAAAAAAGTGATCACCGAGATGGAAAGGCTGGGCATGATTTTGGATGTTTCCCATTTGTCGGTGCGAGGGTTTTGGGAAGTGATGGAGTCTGATTTGCCGGTGCTTGCTTCTCACTCCAATTGCCGGAAAATCGCCCCTCATCCGCGGAATCTGGAAGATGAACAAATCCAGGCGCTGATCCGAAAAGGCGGATTGATCGGGATCACCTTTGTTCCATCGTTTACATATCCTTGTGAAAAGGAAGCCACCATCGATCATCTTTTGTTACATATCGAACATGTATGCAGCCTGGGAGGCGAACGGAATCTTTGTTTTGGATCGGATTTTGACGGGATCCGTTTGAAAATTAAAAATTTACATAATTACAGGGATATATACTGGTTGAAAGAAGCGCTTTTGCGCCGTTACCCTGAGTCATTGGTGAGGAAATGGGCATGGGAAAATGCCTGTGCTTTTTACTTAAATCATTTACCGTCATAAAGCCGAAAAAAGTTTAGCAAAAATGATCTTTTTTTTACTTAGGTCTTTTTTTGCAACATTAAAAGAAAAATTGGAAAGATAGAGAACTTAAAACGGTTGCTTTTTATACCATACAGGTCTAGAATGATACATGAAAATGCTATCACTCAGCACAAAGTTGGCTTTATTCCTTTCTTTGTGAAGAGTTGATATCTTTCATAGTTATTTTAATATTGTGTAGTGTGTGGCAGTATTAAAGGAGATGAGTTCATTGGTTAAGCAGCTTTCGTGGAAAGTGGGCGGACAACAAGGGGAAGGTATTGACAGTGCAGGAGAGATTTTGTCAACAGCTTTAAATCGAAAAGGGTATTATTTATACGGGTACAGACATTTTTCTTCACGAATTAAAGGTGGTCACTCCAACACCAAGATTCGTATCAGCATAAACCCAATCCGCTCCATTTCCGACGAGCTGGATATACTTGTTGCATTTGACCAAGAATCCATTGACTTGAATGCTCATGAATTGGTTGTGGGCGGGGTCATCTTGGCCGATGCCAAGTTTAATCCAACCGTTCCGGAAGGAGTAAACGCAAACCTTCTTGCGATTCCGTTTACCAAGATTGCCGAAGAAATCGGAATGTCCCAAATGAAAAACATGGTGGCTGTCGGGGCCTCGGCTGCATTGTTGGGCATTCCGGCGGATGCTTTTAAAGAAGTGATTGAAGAAAGGTTCGCACGGAAAGGCCAAAAAGTGGTAGATAGGAATATGGAAGCGATTACAAGAGGGTTCGAGCATGCGGAAGGCGAACTTGGTGATGGAATAACAAGATTTCGCTTGGAACCTTCTGACGGGAAAAAGCGGTTATATATGATCGGAAATGACGCCATTGCACTGGGGGCGATTGCAGCGGGAGCCCGGTTGATGGCTGCATATCCGATTACCCCATCTTCAGAAATCATGGAGTATTTGATCAAAAAACTTCCGAAATTTGGTGGTTCGGTAGTTCAAACAGAAGACGAGCTTGCAGCGATCACCATGGTGATCGGGGCCAACTATGGTGGTGTTCGTGCTCTCACGGCTTCAGCAGGGCCTGGATTGTCACTGATGACCGAAGCGATTGGGCTGGCCGGGATGACCGAAACGCCGGCGGTGATCGTGAACACGCAGCGTGGCGGGCCCAGTACCGGTCTTCCGACCAAACAGGAACAAAGTGACTTATTGTCCATGCTGTTCAGCACCCACGGTGAAATCCCGAAAATTGTCATTGCTCCGAGCACAGCCGAAGAATGCTTCTACGATACCATTCAGGCGTTCAATCTGGCGGAACAGTATCAATGCCCGGTGATTATCCTGTCGGATTTGGCGATGTCGCTCGGGAAACAAACCGTTGAGCCGCTGGAATACGACCGGATTGAAATTAAACGCGGAAAGCTGGTTTCCTGGGATGAAGAGCTTCCGGAGTTGGAGCCTTCCCAATTATTTTTGCGTTATGAACTGACTGAGGATGGCATTTCCAGAAGGGTTCTTCCAGGACAGAAAAACGGATTGCACCATGTGACCGGAGTGGAGCATGACGAAACCGGACGTCCGTCGGAAAAACCGTTAAACCGCAAAAAAATGATGGAAAAACGGCTCATGAAACTGAAAAACGGCATCGATTTTGACACCCCGGTTTACCTGGATGCTCCGCACGATGATGCCGATCTTTTGGTTATCGGATTCAACTCGACCCGCGGCACGATTTCCGAAGCGAAAGAGCGCGCAGAAGCAGAAGGCTTGAAAATCAACCATGCACACATTCGCCAATTGTTGCCGTTCCCGACGGAAATCATCCGGGAGCAAATGAACAGAGCGAAAAAAGTATTGGTTGTTGAAAACAACGCTACCGGACAATTAACCAATGTCATCAAAATGTATGTAGGAATGGCTGAAAAACTGGTTCACTTTGGAAAATATGACGGAAACCCATTCCTGCCTTCTGAAATTTATCATCAATGTAAGGAGCTGTTGGTCCATGGCCACGTTTAAAGATTTTCGCAATAAAGTAAAACCCAACTGGTGTCCCGGTTGCGGTGACTTTTCCGTGTTGGCCGCCATGCAGCGCGCTTTCGCCAATACCGGTTTGGAGCCGCATGAAGTAGCCGTTGTTTCAGGGATTGGTTGTTCCGGACGGATCTCCGGATATATTAATGCGTATGGATTTCACGGTGTCCATGGCCGAGCGCTGCCGATTGCACAAGGGTTGAAACTGGCTAACCGCGATTTAACGGTGGTTGCAGCCGGCGGGGATGGAGACGGATTTGCCATTGGGATGTCGCACACCATCCATGCCATTCGCCGGAATGTGGATATCACCTATATTGTCATGGATAACCAGGTATACGGATTAACCAAAGGCCAAACTTCTCCACGAAGCGAAAAAGGATTTAAAACCAAAAGCACGCCGCAAGGTGCCATTGAAGCACCCATTCATCCAATGGAAATGGCGCTGACAGCAGGAGCCGGTTTTGTGGCTCAATCAGTTTCCAGTGACCTGAAACAATTAACCCGTGTGATCGAAGAAGGGATCAAGCATAAAGGATTTTCCTTTATCAATGTTTACAGCCCTTGTGTAACGTACAACAAAATCAACACGTATGACTTTTTCAAACAAAATCTGGTCAGCTTGGATGATGATCCGGATTATGATTCATCCGACCGACAAATGGCCATGCAAAAACTGATGGAACATCAAGGATTGGTCACAGGAATCATCTACCAAAACAAAGAGCAACCCTCCTATGAATCCGTGATCAGAGGCTTTAAAGAAGAGCCGTTGGCAAAACAAGAGCTGAAAATTTCCGAACAAGACTTCGAAGAATTGGTTGCCGAATTTGCATAAAGTAAAAAAGCTGTGCAGACTAATGTCTGTACAGCTTTTTTTGTGGAATGGACGGAGAGAGTTTGTTGTGATAATGGAATGCGAAGGAGGATGCCTGATGAGATGGACGGTGCTTGGTTGCCATTCCCCTTATCCGGCGCCGGGAGGTGGGACTCCCGGTTATCTGTTGGAGGCGGAAGGGAAAAAAATCCTGATTGATTGCGGCAGCGGCATACTGGCCTGGCTGGTCGAACGGATCGCCATCGAAGCGTTGGATGCCGTTTTGTTGTCGCATTTGCATCATGACCATATCGTTGATTTTTTTGTTTTGCAGTACGCGGTCATGACCGCCCGAAAACAAGGGAAAAGGAAACACCCCCTTGCGGTCTGGTCACCGGAGAAACCGGCCGGTTGGTTTGAAAAGTTGCGTTATAAAACAGAGATTGACCGAAACGGGATCCGGGAAGGGATGAAAATTCATTTGGGCGGGCGGTTGACCGTGCAATTTTTCCGCACCGATCATGCCGTCCCTTGTTTTGCCATGCGGATCCATGACGGTAAACGCACCATCTTGTACGGGGCAGACAGTGGCCCGGAGACAGATTGGGAAAAGATGGGGAAAAGCCCTGACCTGTTTGTTTGTGAAGCAAGCTTTTTACATAAAGATTTGCCGGATCAACCAACAGGGCATCTGTCGGCCAGACAAGCCGGGCTTGCTGCCGGCCGGCTCAAGGCCAAGCAGTTGATCTTGACCCATTTGTATCCCGGGTATGATCCGGATCACCTGAAGGAGGAAGCCATGGCGGTGTTTGACGGTGTTTGCCAAACCGCTTATTCCGGCATGACGGTCGATTTGTAGCTTTTGTTTGGGCCGGTCATCCATTATCATGGAGAGACAAGCACGGTTTTGAATCAGCCGATGGAAAGAGGGGAGAGCGTTTGCAGAAACTGAAGGAGTATATTGTCAAAGAGGGATTGGTGCTGTCCGATGACATCTTAAAGGTGGATGCATTTTTGAATCATCAAATCGATCCTTTTTTGATCAGGGAAGTCGGGGAAGAGTTTGCCAGGCGTTTCCAAGATGAATCCGTCACTAAAATTTTAACCATTGAGTCGTCGGGCATTGCGCCGGCTATCATGACCGGATTGGCATTGCACGTTCCAGTGGTTTTCGCCCGGAAGAAAAAATCGCTGACATTGAAGGATTCCTATTATGAAGCCAAAGTTTTTTCATACACCAAACAAGAGACGACTTCCGTGGTGGTGGCGAAAAAGTTCTTAACCCCAAAGGACCGGGTATTGGTGATTGATGACTTTCTGGCCAATGGACAAGCGGCAGAAGGTTTGATTCAAGTGGTCCGCATGGCAGGGGCCCGGTTGGCCGGATTGGGGGCGGTGATTGAAAAATCTTTTCAGCCCGGCGGCTCCAATCTTCGCAAGCAGGGAATTCGTGTGGAATCGCTGGTGGAAATTGCATCTTTGGCTGATGGAAAAGTTCATTTCAAAGAAAACTGAGGGTCAAGCCGGATTTCTCTTTATGAGGGAGATCCGGCTTGATTGATTGGTGTTTTGAAATAAACAAGTTTGCAACACAAACAATTCGCTAAAATTCATTTTCCCCGGCTGGGAAATTCGCAACAACATGTTATAATGGGGGAGAATCATCCGAGGATCTTTATTGAACACATGTCACTCATCGTGTATAATGAGAAAATTGCAGTATTCGTGTTCAGCCTTCGAAATCATGGATTTATTCATTATAGAAGCAATCTCACCAGAAAGGGTGGATGAAATGGAACGAAAAGAGAAAGATTACAGCCAATATTTTGTTGCACCCGATCTGAAAGCCGCCAAAAGGCGGGGGAAGTCGGGTGTGGAAGTGATACAATTTGATCAGATTCCCGAAAATATGCGTGGTGTCGGCGCAGGAAAGAAATATTTGATTCAAACTTACGGATGCCAAATGAATGTTCATGACAGCGAAACGATCGCCGGAATTCTGGAACAAATGGGCTATGAACGGACAGAACGGGAAGATCGAGCGGATATTATTTTGCTGAATACGTGTGCCATTCGCGAAAATGCAGAAGATAAAGTGTTTGGCGAATTGGGACGCCTCAAACACCTGAAGTTGGAAAACCCGGATTTAATCCTCGGGGTTTGCGGTTGCATGTCCCAGGAAGAAGTGGTCGTCAACCGGATCTTAAAAAGTTATCAGCATGTGGATTTAATCTTTGGCACACACAATATCCACCGTTTGCCGGTCTTGTTGCAAAACGCCTTATTCAGCAAGGAAATGGTTGTGGAAGTATGGTCCAAAGAGGGCGATGTGGTTGAAAACATGCCCAAGAAGCGTGAAGACGGACTGCGTGCCTGGGTCAACATTATGTACGGATGCGACAAATTCTGCACGTACTGTATTGTTCCTTATACACGGGGGAAAGAACGCAGCCGCCGTCCCGAAGACGTGTTAAATGAGATTCGCGAACTGGCGCGCAAAGGCTATCAGGAAGTGACTTTGCTCGGACAAAACGTGAATGCTTACGGAAAAGATTTCACCGATATCAATTACCGGTTTGGCGACTTGATGGATGATGTCCGTAAAATCGGCATTCCGCGTGTCCGGTTTACGACAAGCCACCCGCGCGATTTTGATGACCATCTGATCGAAGTTTTGGCCAAACGCGGAAATCTGGTCGAGCACATTCATCTGCCGGTTCAATCCGGAAGCTCGCAAATTTTGAAATTGATGGCGCGCAAATATACCCGGGAGTCTTACCTTGAATTGGTCCGCAAAATTAAAGAAGCCATCCCGGATGTGGTGTTGACCACGGACATTATTGTGGGATTCCCCGGAGAAACCGAAGAACAATTTGAAGAAACGCTGTCCCTTGTCCGGGAAGTGGAGTTTGATTCGGCGTTTACGTTTATTTACTCACCGCGTGAGGGAACGCCGGCCGCCAAAATGAAAGATGACGTGCCAATGGAAGTGAAAAAAGAACGACTGCAACGTTTGAACGATCTGCAAAACGAAATCAGCCGCCGGAAAAATGAAGCTCTCCGCGGCCAAGTGGTGGAAGTTTTGGTGGAAGGCACAAGCAAGAAAAATCCGGATGTGTTATCGGGACGGACCCGTACCAACAAGCTGGTGAATTTCACGGGACCGAAGCATCTGATCGGGAAATTTGCCCATGTCCGGATCGATGAACCGCAAACCTGGACGTTAAAAGGAGTTTGGGTGGAAGAGGAACCTCTGACGAAAGTGGGAGGGTAAAATGTACACTTCGTTTGAAAACCATCCCGTGTTGGTGCAAGCCCGGCAATTTGCGGACTTGATTTTAAAAAGCGAAGAAATCCAACGGTTTCGTGCCGCGGAAGAGCAGGTGAAAAAAAGCAAGACCGTCCAAAATTATATTGACACCATCAAGCGAAAGCAAAAAGAATTGGTCCATGCCAAGCATTATCAGAAAACCAAATATGCCCGCATCTTGGAAAAAGAACTGGAACAGTTGAATCAGGAACTGGAGAACCTCCCCATCATCCGGGAGTATCAGCAGACGCAAGTGGGTGTGAATGATCTTTTGCAAACCATCCAGCAAGTGATTGGCACCACTGTTTCCAAAAAAGTTTCCGTTGAAACCGGTGGTGAAATCCGGCAGGGATGTGGAAGTGGCGGGCCTTGTGGTTGTTCGCGGTAAAAAAATTTTCCCCCGGGATGATTTCCCGGGGGTTTTTTTGCACCCTAGACAGTTGCCCTGCATACACTTGTTATTGAATGATGCTTGGAGGAGGGAAGTATGGTGTCATATCCAGATAGAGCCATGGAATACCGACAAATTATTACCAAGGCTGTATGCGGAAAAGGACGCAGATTTTTACAGTCCACGCACACAGTGAAGCCGCCGGAAAATATCCATACGATCTTGGGGGCATGGGTGATTAATCATCAGTACGAATGCACAAAAGTAGGAGAAGCCGTGGAAGTCAGCGGCAAGTACGATGTGAATATCTGGTATTCCACCAAAGGGAACACCAAAACCGATGTGATTAAAGAAACCGTTTATTACACCGAGCAAGTCCCGCTTAGCTATTATGACCGCAACACCCGTGACTCCACCGTTACCGTCAGTGCCACGGTGACGCAGGCTCCCAACTGTGTGGAAGCTTCCATCGCTTCACACACGGACGGAATCGCAGATGGCATTGTCGTCCGGGTTGAAAAAGAATTTGCGGTTGAAATGGTTGGGCAAACGAAGATTTGCGTACCGGTCTATCCGTTAGAATATGCAGAAACTGATGAAAAAGATTTGGCGGGAAGCTATGAAGAACATGTGGATGAAGATTCTTTTGATGAACTGGATCCGGATCTGATCATTGATGACCTGGAAGATTGATTAACGGATATTCCTCGGGAACTGCTGGCACCCAGTCAGCAGTTTATTTTTTGTTTTTGGATCCGAAACAACATTTAACCAATTGAGAAGCCGAGACATAACATAGATCATAATGCTCTTGACGGAAAGTGGGATGCACAGAAAGGGGAAAGGTTATGGGAGCCGTCACCCTTCCATATATGGATTTTAACAATCAGCAATCAATCAAAAATCTTTTGATTGAACAACAAGGAAGACTCGCCTCAACCATTCATCTGGAAAAGAGCGGATCGGTGGCCGTGTCTGAGCCGGTTTTGATTTTAAATGATGAAAGACATGTCCAAAGGACAAAAAATCCTTCGGCTTGCCACCGGATCTTAAGGATGCACGGGGTTCCCGTGCATTCCCATCATCATCTGGTCTTGCGGGAGTACATGGTCGCTGTTTTCCAAACCAATGTGTTGGCGGTTTATTGTTCCCGTCAACAGGGGGCTTGGCTGGCGGAGCAAAAGAGGAATTGGAAAAACAGTTTCCGGCGGGTCTCTTTGCAGGATCCGTCGAGGGAAGTGCGGAAAATCAAAGAATGGGCCGTCCGGGCTTTGTATGCGTTGGGATTGGATTACGGACTGGTGCGACTGGCCGTGGGGCCCAACCGGAAATATTTTGTCCGGCAGGTGGTATGTGATCCCAAGTTAAACAAAGAAATGAAACAAAGCTTCGTGAAAGCCGTACAGCAATATGTGAAAGAGTGTGTAAATCTTCCGGCCATTCCTTGGAACCAGGTGGTGCTGGGAGCCGATCCGGAGTTTATCATGGAAGGAAGGTCCGGGGGATTGCTGATGGCATCCCGATATTTCCCGGTGAAAGGGAGGGTCGGGTGCGATGCGATCTGGATGAACCAAAACCGTTCCTTAAAGCCGCTTGTGGAGATCCGGCCGGAGCCGACTCCGGATCCGCGCGCACTGGCCATCAACATCTTTAAAGGGTTGTTGTACGCAGCCAAAAAAACAGGGCGCGCCCCGGCAAAATGGCTGGCCGGCGCATTGCCGCACCATGCTTTCCCGTTGGGAGGGCATATCCACTTCAGCGGTGTTCAACCAAACTTTAAATTGTTGCGGGCTTTGGACAACTATTTGGCGTTATTTTTGGCAATCGTGGAAGATCCGCAGGGGATCGGGCGCCGTCCCAAGTACGGGTTTTTGGGGGATTTCCGATATCAGGACCACGGCGGGTTTGAATACCGGACGTTACCCAGTTGGCTGATTTCCCCCACCCTGACCAAAGGGGTCTTTGTCGTGGCCAAGTTGATTGCCATCCACTACCGGTATTTCAATTATTATCCGCTGGACGAGGAAGACGTCCAAGAAGCGTATTATCAGGGGGATAAAGAGGTGTTGGCCAAGTGGTTGCCCGTGTTGTGGAGCGAATTGAAGAAGTGCCCTTATTATGGGCGGCACAAGGAATATCTGGATAAATTCTACAAATACCTGACTTCGGGATCGACTTGGAATGAATCGCAAGATATTCGAAAAGTGTGGAAACTGCCGCCTTACCAGAAGAAAAAATAATGGAAAAGTGGGACGGTGCCTCGATTCATGTTAAAATATTGCAGTGAAAAATATTGTAACGGAGGCAGAGAACTTTGGCAAAATATACTCCGATGATTCAGCAATACTTGTCGATCAAGGCACAATATCCGGATGCCTTCTTATTTTTTCGTTTAGGTGATTTTTACGAGATGTTTGTGCGCCCATAAGGACTTATAGGGAATCTGCCCTTGGCAAGGCAGTGGGTGAGGGACGCGGAAAACACCCATCATCGACCAACTGATCCGGAGGGGAAACCCGAAGGGGAGTGTAGCATGTTGGTAAAGTCACGAGTCACGAAGCTGCCATCGTGCGACTGAGTGGCGAGATGAAAGGTTGTGAATGAGGATGAAAGCTGGATTGCTTGAATGACAGCCCGAGCCGGTTGATCGTAGCCCATGGCGGAAGGCAGCTGTATACGCCATGCAGCCGTGATGATTCGCAGAAAGACATGGCGGTCATCGGTGCGTCCTGCGGCTGGGCAGCCTCTGTCAGAGGAAAAGGGCGAACGTCACATCCGACATCACAACGTGCCTGTCTCTATAAGTCTAAATGGGGATCACCTGAGTCGGAATGCCTGCCTGTCGGGCTATGGCCGCAAGGGCCTGAATATCCGATATGGTGACGGAGCCCCCGTAGTAGTCTGAGCCGGGGAAAGCCCGGCACACGGCGAAGGGGGGCAGCTTGCTCGGGTTCCATACGACAAAGGAAGAAGCGTGAGGCTTTATGAGAAATCCTGAGTACGTATTGAACAGTCTGGCCGCGAAGTCCGGCGATTCAACCTATTTATTCCAAAGGATTTACCGAAATTTGTATAACCCGCGGTTTTATCAAAAGGCATATCAGGAACTGATCCGCCGGTTCCCTCAGGTAAGACACCGGAAACTCCTTCCCGGACAGCTGGAATCCCTGATGAAGGAATTGAAAAACGAAACCTATCAGCCGGCACCCTTTCCCGGCGCCGGGGAGACGGAACCGCTTCGGGATTGCTTGGTGGCATGGATTGTCCGCATGCTTCTCGAGGCGATCTATGAGGGTTCTTTTTCGGACTTGTCCCATGGTTATCGTTCTCACCGAAGCTGCCATACCGCTTTGTTGCAAGTAAAGAAAAATTTTTCCGGTGTCAAATGGTTGCTGCACGGAAGCCTGCATTCTTTTTTCCCTTCCATTTCTTTTGAAGTGCTGCGTTCCCTTCTGAGAAAAAGGATTGAGGATGAAAAATTCATCCGGCTCATCAACAAGTTTTTGCGGGCAGGATGGTTAGAGGATTGGCAATTCCACCGCACCTGCAGCGGGGCTCCCGTGGGCGGATGGATCAGCCCGGTTTTGGCCAATATTTATTTCCATGCGTTGGACCGGCATGTGGAAACGCTGATCCATGAGTTTTCCAAAGGAAAGAAGCGCAGGAGGAATCCGGCTTACGAGAAGCTTCTTGCCCGTATCAAACAGGTGGAACAAAGAAAAAGGGATGCAGAAAGCGAAGCGGAAAAAGAGAATTTGAACCGGCTGCAACAACAATGGACGGAACGGTTGAAACAAATCCCCCGGGAAGATCCGTGGGATCCGGCTTATAAACGGCTCGTGTATACGCGTTATGCCGGGGAGATCTTATTGGGGGTGATCGGTTCCAAGAAGGAATCCTCAGCCATCGGGGATGAGGTTGTCCGGTTTATAAAACAGGAATTGAATCTTCCTTTTTCCCGCGAAGCACTTATTTTAAAACATGCCAAAAAACATACGCGTTTTCTCGGATACGACATCACCGTCAAAGAACAGTGCAAACTGCTTTTTCCAAAAGATGTCCGGATCAAAAGACTGCATCAATTCGGGGCGGTGAAAGAACAAAACGGCAAGCTGAAGCCGGTACATAAGAAGGAACTGGTTCCTCTTCCCGATGCGGAGATTCTGCGCGTTTATAACCGGGAGATCCGGCGCATGTACCATTATTACCAATTGGCGGAGAATGTCGGCACTTTGTCACGGTATGCCTATTTGCTGAGATACAGCATGTATAAAACATTGGCCATGAAATACAAGTGTTCCGTGCATCAACTCTTGAAGAAGTATATGCATGCGGGGACCTTTCAGGTGCCGGTTGAAACCGGAGACGGGGAAAAAGCGGTTGCCACATTCCATGACGAAGGGTTTAAGCGCAAGGAAGACCCCGTCTTACATAGCGACGTCGACCGGGTGGATGGATCCGGACCGCCGGACCAAGGCCGGATTTGAGCAAGCGGAGAGCCGTATACGCCGAGAGGTGTACGTACGGTTCGGAGAGGGGTTCAGAGAAACCTGCCGTCAGAAGGCGGCAAGGCGCTTTGTTCCTACTCTACTTCGAAGATGCCGAAAAAGCAGCCGAAGAGCTGGAGATTACATTGACCAGCCGGGATGGTGGCAGTGAACGCATTCCCATGTGCGGGGTTCCTTATCATTCGGCTCACACTTATGTGGAAAAATTGATTGATCAGGGATATAAAGTGGCCATCTGTGAGCAGGTGGAAGATCCTTCCGCTGCCAAAGGGGTGGTCAAGCGGGAAGTGGTTCGCGTGATCACGCCCGGGACCATCATGGAAGAAAATATGCTGGTCGACCAGGAAAATAATTTTTTGGTGACGCTGACCGGAAGCGAAAACCAAATGGCATTGGCGGCAGTGGATTTGTCCACGGGAGAGTGCCACATTACGGAGATGAGCGGTTCGCTGGACGCGATTTTGGATGAAATTTCTTCGTATCGGCCGAAAGAAATCGTGTTGGATGAATCCTTGGCAGATCAAGCGGCGGTAAAAGAACAGATTCAATCCCGCTTAAAATGTTTGATCACGCCGCATGGCATGGATGCCGGTTCCGAAAAGAAACTGTCCGAAGAGTTGCCGGCGCAGTTTCCGCAATACCCCGAGCTGTGCGATACCCCGTTTTTGCGAAAAGTGGTCGCCTTGTTATTCTCTTATCTGAAACAAACGCAAAAGCGGGCTCTGCATCATTTGCAGCGGTTGCACCGGTATGACGCCAGGCAGTATATGATGTTGGATGAAGCGGCGAGAAGAAACCTGGAATTGACGGCAACGCTTGGCGAAGGAAAGAAAAAAGGTTCGCTATTGTGGCTGTTGGATCAAACGGCCACCGCAATGGGGAGCCGGTTGTTGAAAAAATGGCTGGACAAGCCGCTTCTGTCACTGGAAGAGATCACCCGGCGCCAAAATATGGTGGAAGCGTTGCTTTCCGACTTTTTGTTGCTGGATGAAGTGCGGGACCGGATGAAACAAGTTTATGATTTGGAGCGGCTGGCGGCGCGCATTTCTTACGGATCGGCCAATGCGCGGGATTTAAACAGTGTGAAAAGATCGCTTTCCATCATTCCCGAACTGAAAGAGAAATTGTTGCAATCCGACTCCCGGCCGCTGAAGGAATTGGCGGAAAAAATGGATGAGTTGGCGGATGTAAAAAGCCTCATCGACGAAGCGTTGGTGGAAGAAGCGCCGGTTTCCGTCAAGGAAGGCGGCATCATCCGCGAAGGCTTCCATGAAGAATTGGACCAATTGCGGAAGATCCAGAAAGACGGGAAAAGCTGGATTGCCGAGCTGGAGCAGAAAGAAAGAGACACGACGGGAATCAAGTCGCTAAAAATCCGTTATAACCGCGTGTTCGGCTATTATATCGAGGTTACCAAGTCCAATCTGCATCTGGTTCCCAAAGATCGGTACCACCGCAAGCAAACGCTTGCCAATTCGGAACGGTATATCACGCCGGAATTAAAAGAACGGGAACAGCTCATCTTAAATGCTTCGGAAAAATCAGTGGAACTGGAATATGAACTGTTTACAAAGGTCCGGGATCAGGTGGCGGACCAGGTTCAACGATTGCAATCTCTGGCCGAACGGGTGGCCGAGTTGGATGTATTGCACGCCTTTGCGCTGATTGCCCAAAAGTATCAATATGTCCGTCCGAAAGTGCATACAGGCAGTCAGCTGAAAATCAAAGCAGGCCGCCATCCGGTCGTCGAAGCCGTTACCAATACGGGGGAATTTGTGGCCAATGATGCGGAGTTGGATCAGGAAGACCGCCAAGTCTTATTGGTGACGGGGCCGAACATGGCCGGAAAAAGCACGTACATGAGACAAGTGGCTTTGATTGTGATTTTAAGCCAGATCGGAAGTTTCGTTCCGGCGGAAGAAGCGGAAATTTCCATTGTGGACCGCGTGTTTACCCGGATCGGGGCGGCGGATGATTTGACCGGCGGGCGGAGCACGTTTATGGTGGAGATGTCTGAAACCTGCCATGCCCTGAAGGAGGCCACCCGGAGGAGCTTGATCCTGCTGGACGAAGTGGGACGAGGAACATCCACTTATGACGGGATGGCTCTCGCGCATGCCATCGTGGAATACATTCATGACCATGTGGGAGCCAAGACGCTGTTTTCCACCCATTATCATGAGTTGACAAAACTGGAGGAATCATTGTCCCGGTTGAAGAATGTGCATGCCCAATGCATTGAGCGGAACGGAAAGGTGGTCTTTTTGCACCGGATTGTCCCGGGAGGGGCGGATCGCAGTTACGGGATTCAGGTGGCCGAGCTGGCCGGGCTGCCGAAAGAAGTGATCACAAGAGCCAAACAGTTGTTGACCGAATTGGAAAAAGGCACACCCGCACCTTCAAACACCGGTGCTTCCCCGGATTCGTCGGGGCAGTTGTCCTTGTTCGATCCGTTGGAAATTCAACCGGCAACCAGGGAGACGGCCGCTTCGTCTTTGACCTCCGAAGAACAAGAAGTTTTGGAGGCCGTCCGCAAGTGGGATTTAATGAATAAGACCCCGTTTGAATGCATGCAGTTTTTGCATGAGATCAAACAAAAATTAAATTAAACAATATGGAAAAGGAGGGGAGTGGATCATGGGCAACATTCAAGTGCTGGATGAACATTTGACGAACCAAATTGCCGCGGGAGAAGTGGTGGAACGGCCGGCGTCGGTTGTCAAAGAGTTGGTGGAAAACTCCATCGATGCCGGCGCCACCCGGGTGCAGGTCATGATAGAAGAAGGCGGGATTTCTTTTATTCAGGTGTCGGATAATGGTTCTGGCATGGACCGGGAAGATGCCGTGTTGGCCTTTTCCCGCCATGCCACCAGCAAAATCAAGCGGGAACGGGATCTGTATTCCATCCGGACACTCGGCTTTCGGGGAGAAGCTTTGCCGAGCATTGCTTCGGTTGCCAGAGTGGAATTGGTGACCACGAAGGACCCTTCACAGCTTGCGACCAAAGTGAAAATCGCCGGAGGCGACATCGAAGCGGTGGAGGAAACTTCCCGTTCCAAGGGAACCGATGTGATGGTCCGGGATTTGTTTTATAACACGCCCGCCCGTTTAAAATATTTGAAAACAGTCAATACCGAAGTCAGCCATGTTGCCGATGTTTTGGGAAGGTTGGCCTTTGCCCATCCGGAAATTTCTTTCTCCCTGACCCATAACCGGAGGAAGTTGTTTCAGACCGCGGGAGACGGGAACTTGAAGCATGTACTCCTTTCCATCTACGGGAAACAGGTGGCCGGACAGGCGGTTTCGATCGAAGGAAGCGACATGGACTTCCGGGTTTACGGATGGATCACCAAACCGGAAATCACCCGCTCCAACCGGTCTTATTTGACCATCATCTTAAACGGGCGCTATGTGCGTTCCCTGCCGATCACCCAAGCCATTTTGCGTGCTTACGGCACCTTGTTGCCGACGGGAAGGTTCCCGGTCGGGGTTTTGCATTTGGAGATGGATGCGCAATTGTTGGATGTCAATGTTCACCCTTCCAAGTTGGAAGCAAGGCTCAGCAAGGAAAAAGAATGTTGCCGGCTGGTTGAAGAAACGGTGCGGCAAGCCTTGCAAAAAGAGCGGTTGGTTGCGACACCCAAAGCGGAATCAGCGGTGAAAAAACCGGAGTATAAGCAGGAAACTTTCCAGTGGAACCAAGCGAAAAGACCTGCCGTGAATCCATATCCGTCCGACCGTCCCGATCCGTCATGGAAACTGCGCGAAAGCCCGATGTCGCTGGTCAAGAAAGAGGAGCCGGTCAAGAAAGAGGAAGAGAAGGAAACGGATCCGGCACCGGTCTTTCCGGATCCTTCCCCGTCCCAAAGCAAACCGGAGCCCAAAGATTCAAAATATCAATCCGCCTTGAGCGCATGGTCGGAACAGAATACGCCCCGGGGAGAAGAAAAGGCGACCCTTTCCGCTCCGCCGGTTTCAGAAGAAGCAGCGGCGGAAGAGCCGGAAGCGGAAGCAAACGGGGAAGACCGATTGCCGTTCATGACTCCGTTGGCACAGGTCCACGGCACTTATATCGTGGCGCAATCGGAAGACGGATTTTTTTTGTTGGATCAGCATGCGGCACATGAGCGCATTTATTACGAGATTTATTCCAAAAAGTTGGGGGAAGCAAATCATCACCAGTATTCCCTTTTGATTCCCATGACGATCGAGTGCACGCCGGCCGAAGCCGAAATATTAAACATGCATTTGGATTATCTGAATCAGTGGGGCCTGGAAATGGAGCCTTTTGGCGGAACCACGTTTGTGATCCGCGCTTACCCCGCGTGGTTTCCGAAAGAAGATCCCCAGCAATTGATTCACGAGATCATTGACTGGCTGAAAGAAAACGGGAAAGTGGAGACTGCTCAACTGAGGGATGCAACTGCCAAAATGATGTCATGCAAAGCGGCGATCAAAGCCAACCGCTATTTGCTGAAAGAGGAGATGGAAGAATTGTTAAACCAGTTGAGCCGTTGTGAAAATCCGTTTACTTGTCCGCACGGACGGCCCATTTTTGTTCATTTTTCAACTTATGAATTGGAAAAAATGTTTAAAAGGGTGATGTAATTTTGTTTGTCACAACATCTTTTCACCCGGGAGACCAGGAAACAAACGAAGCAAAAGAATTGGCCCGTGCATGGAAGGTGCCGTTCGTCCGGCGCGGGCGCCGGTCTCTTCAAGCGCTTTTCGAGCAGACGGCATCGGATCAGGTGGTCATTGTCAGCAAAGAGGGATGGCGCTATGAAGACAAAAAAGGCCATTCCTTCTTTTTTCATCCCAACATGTCGGCCCTTCGCATCAAACATTTGGCTTCCGGGCAGCCGGACAGCATGGTTTCGGTGGCCGGATTGAAAGAAGGGGATTTTGTCCTGGATTGTACCTTGGGGATGGGGGCGGATTCCATCGTGGCCAGTTATGTGGTCGGGGAAAAAGGCCGGGTTGTGGCGCTGGAGAGTGAGCCGGTCATCGCGGCGATCGCCGGACACGGCCTTCAAACATACCGGACCGGGCGCAAAGCGTTGAATGAAGCGATGCGGAGGGTTGAAATTGTTCAGGCCCGCTATCAGACCTACTTAAAACAATTGGAGGATCAATCCTTTGATGTGGTGATGTTTGACCCGATGTTCCGGGAAACGGTGAAACAGTCGTCAGCCATGCAAATGTTAAAACCCCTGGCCAACCCGGAACCCTTGGATCCGGAGTCGGTGCAAGAAGCCGTGCGGGTGGCCAGGAGGGTTGTTTTGTTAAAAGAGCGGCCGAAAAGCGGGGAGTTTGAACGGCTGGGCTTTACGATTGTTAAAGCTTCATCCAATTACGCCTGGGGTGTGATCCGGAAAGGAGGTCACCGGTGAAGGAAAAGCTTGTTGTCATTGTCGGACCGACGGCGGTCGGAAAAACCGGTTTGAGCGTACGGTTGGCCAAACGGGTTCATGGCGAAATTATTTCCGGCGATTCCATGCAGGTTTACCGGGGGATGGACATCGGGACAGCCAAAATCATGCCGGAGGAGATGGAGGGGGTTCCCCATCATCTCATTGACATTCTCCCGCCGGATCAAAATTTCTCGGTTCAGGAATATCAACAGCTGGCACGGGAAAAAATCAGCGAGATCAACCGGCGCGGGCATCTGCCCATGCTGGTGGGGGGAACCGGTTTGTACATCGAAGCGGTGGTCCATGATTATCTGATGCCCCATGTCAAAGAGGATCCAAAGCTGCGCGAAGAATTGCATGCGTTTGCCCGGCGGGAAGGCAACGAAGCGTTGCACCGGCGGTTGGAAGAAGTGGACCCGGTCCAGGCCAAAAAATTGCATCCCAATGATCTCAGACGTATGATTCGGGCTTTGGAAGTTTATCATGTTACTGGAAAGCCGTTTTCCGAGCTGAAAGGAAAAGGAACTTCGCCTTATGACGTGTTGTGGATCGGGCTGACGATGCCGCGGGAACAATTGTACGAGCGGATCAATCGGCGGGTGGACGAGATGATCCAAAAAGGCTTGGTGGATGAAGTGAAAAAACTGTGGGAGCAAGGATACGGGCTTCATTTGACTTCCATGCAGGCAATCGGGTATAAAGAGATTGTAAGTTATTTGAAAAACGAAATCACTTTGGATGAAGCGATTCATCACATCAAGCGCGGATCGCGAAAATACGCGAAACGACAGCTCTCCTGGTTTCGGCGTTTAAAAGAAATTCATTGGTTTGATGTGACAAATTCCCAATCTTTTCGAGAAATTGAAGAACTGGTGGCAGGAAAGTTTCCTTTGGCCAGAGAATAAAGTACAATAACGTTAAATGTAAATACATAGGAGGTACGGGTTTTGAAACAAACAATCAATATTCAAGATACATTCCTCAACCAAATCCGTAAAGAATCAGTACCAGTGACCATTTATTTGGTGAACGGGTTTCAATTGCGCGGCGTGATCCGCGGCTTTGACAATTTCACCATTGTGATTGACAGTGACGGCAAGCAACAAATGGTATATAAGCATGCGATCTCCACGTTCACCCCTGCACGTCCCGTTTCTTTGATGCCCGCCAGTGAAGAAAGAGAAAGCTGAGTTGTTCGTTTGACTTGTTTTGCTGCTGACCAAACCGGCCGGCAGCTTTTTTTATTGCCGCAGGTTTGACAGAGTCCGTAAAGGGGCCGGACGGCTCTTGTCACCATCAAACCCTGAAAATGCCGTATTCAACGCGATTTTTCACCAAAAAATGGGCGGATGCGTATACTGGGATCAGCGAATAAGGACGAAGAGGTGAAACGATGAGCAGAAGGGTGATGACAGTAAACGCCAAGCATCAGATTCATGTTGTTTTTGACCATCCGGAAAGAAAACCCGCGCTTTCGTCGCGGGTGGAGCCGTTGAAACCGGCTTGTCCGGGCGGTGAACCGGGGCCTTTGCAGGAGTGTTTTGAACAATTGGACCGGCTGGTGGGGATGGATAAGATCAAGGAGTTCATCAAAGAGATTTATGCATGGCTGGAAATCGGGAAACAGAGGAGAGCGGCCGGTTTAAGCGCCGAACAACAAGTGTTGCACATGATTTTTTCCGGGAGTCCGGGGACGGGGAAAACCACGGTTGCACGGATTTTAGGCCGGTTATTAAAGGAAATGGGCGTGCTGTCCAAAGGGCATTTGATCGAAGTGGAACGGGCGGATCTGGTGGGCGAGTATATCGGCCATACGGCGCAAAAAACAAGAGAACATGTGCGAAGGGCGTTGGGCGGTGTTTTGTTTATTGACGAAGCTTATTCGCTCGCGCGGGGCGGGGAAAAAGATTTCGGCAAAGAAGCCGTTGATACGATGGTCAAATCAATGGAAGACTATAAAAATGACTTCATATTAATTTTGGCGGGGTATCCCAATGAAATGGACGCATTTTTGCGTTTGAATCCCGGCCTTCCTTCCCGCTTTCCGATTCATCTTCATTTTCCGGATTTCAAATTGGATGAATTGATGAAGATAGCCGGGCAGATGCTTCAAGAGCGGGAGTATTTCTTATCCCGGCCGGCCAAAGATAAATTGCGAAACCACTTGCAAAAACAACTCCACGTTGCCAAGGCCGGTTTTGGAAATGCCCGGTACGTCCGCAATTTAATTGAAAAATCGATCCGGAATCAAGCGGTGCGGCTGATGAAAAAATCGCATGTGACCAGAGAGGAATTAATGACGTTAAGAAGTGAGGATTTGCGGTTTGACGATATGAAAAACGGTTCATGGTATAATGAAAAAATGCCTTATCAGTATTTGGTATAAGAAAGGGTTGAGTGCGTATTCAGGAGTTGGAAGGAAAAGAGAAAGCGATTTTGGTGGGGTGCGGCACGAAAAAACAGGAATGGTCCTTGCGCTCCACCTTGGAAGAGTTAAAGAGTCTGGCGGAAACGGCGCAAGCGGTTCCCGTCAGCCAGGTGCTTCAATTCAGGGACCGGATTGACCCGGCTTGGTATATCGGGCGGGGAAAAGCAGAAGAAATCGCCCGCATGGTGGAAGAAAACGAAGCGGACCTGGTTATCTTTGACCAGGAGCTTTCCCCTGCGCAGCTTCGAAACCTGGAGGGGTTGATGCCTTGCAAAGTGATTGACCGCACGCAATTAATTTTGGATATTTTTGCGCAGCGGGCCCGGACCAAAGAAGGAAAAATCCAAGTGGAACTGGCGCAGTTGAAATATTTGCTGCCCCGCCTGGCCGGAAAAGGGAAAGAAATGTCCCGTCTGGGCGGCGGCATTGGAACCCGCGGTCCCGGTGAAAAGAAACTGGAAACGGATCGCCGTCACATCCGCCGCCAAATCAGTGTTCTGACCAAACAGCTTGAGGAAATTAAAAAACACCGCCGGTTGCATCAGGAACGCCGGAAAAAGAACGGCATCCCGCAGGTGGCGTTGGTGGGATACACCAATGCCGGAAAATCGACATTGTTAAATCAACTGACGGGTGCGGGCGTGCTGTCCGAAAACCGGTTGTTTGCCACATTGGATCCGACATCCCGCCAGCTTGTGCTTCCCTCTGGCAAGGAAGTGATCCTTACCGATACCGTCGGTTTTATCCGGCAACTGCCGCACCATTTGATCGCCGCTTTCCGCTCGACGTTGGAACAGGTGAAAGAAGCGGACTTGTTGTTGCATGTGGTGGATGCTTCCCACCCGGAAGCAGAAGAGCAGGTGGAGGCCGTGGAGCAGGTGTTGGCTGATCTGAATGCGGCGGACATTCCGGTTTTAATGGTCTGGAACAAAGCGGACCGGCTGGAGCGGGAACTGCCCGAAGGATGGGATCCGGATCACCTTATGATCTCTGCGTTCAACGAACAAGATCTGGACCGGTTGAAAGCACGTGTGGAGCAGGCATTGCAACAAGAGATGATCATTGGTCAGGTGGAAATCCCGGTCATGAGGGGTGACTGGATTTCTCTGTTGCATCATAAAGCGGAAATGATAGAATCGAATACGGACGATTTGATGATGGTCATAAAGTTTCGTCTTTCTCCCGCTGATTTTGAACAGTTGCCCGGGGAGTTGAAAAGCCGGATTCAAATCCAATAAACGAGTAACAGGAATGATGACTGTGTTTGAAACTTTGAAACATGGAGCATGGATTGAAGAAAAAGTCCAAACTGCAGAGAAAAAGATTGAACCGGTGCTTAAAAAGATCGGTCAAAATGCCGATTTCATGCAGTGGAAGGTGCTGGAAGCCTTCCGGGAAATGAAAGTAAGTGAAATTCACCTTCATTCTTCAACCGGTTACGGGTATGATGATCTGGGCAGAGAAACGTTCGAAGCGGTATTTGCCCGGGTTTTCGGCGCAGAAAAAGCTTTGGTCCGTCCAAACATCATTTCCGGCACCCATGCCATTGCCGCTTGTTTGTACGGGGTGTTGCGTCCGGGGGATGAGCTGATCTATTTGACGGGCCGTCCTTATGACACGTTGCATCAGGTGATCGGTTCATGCAAAGACGGTTCCGGTTCACTCAGGGATTACGGCATTGAATACCGGGAGATCCCGCTTGCGGAAGGAAACCGGATTGATTGGGCCGCCTTTCGTCAAGCGGTCGGCCCGAAAACCAGGATGGTTGCCATCCAGCGGTCCCGGGGTTATGCCGACCGTCCGTCTTTTTCCATCCGGGAGATTGAAACGATGATCCGGAAAATCCGGGAAATCTGTCCGCAGGCGGTTGTTTTTGTGGACAATTGTTACGGGGAGTTTGTCGAGTCCAAAGAACCGACTGCAGTCGGAGCGGATCTTGTTGCCGGGTCTTTAATCAAAAATCCGGGCGGAGGGATTGCGAAGTCCGGGGGATACATCGCCGGAGCGGAAAAATGGGTGGAAAGAGCCGGATCCCGGCTGGTGGCTCCCGGCATCGGGATCGAAGGGGGAGCAACCCACGGCTATATGCACGATTATTATCAAGGATTGTTTTTGGCTCCACACGTGGTTGCCGAAGCATTGAAAGGAGCTGTTTTTGCCGCGGCATTTCTGGAGGAAGCCGGTTTTCAAACCACGCCGGGATGGGAAGAGCCGCGCACGGATATCATTCAGCAAGTGTATCTGGGAACGCCGGAGTTGCTGGTGGCTTTTTGCCAGGGGATTCAATCGGCATCGCCAGTTGATGCGCATATTTTGCCGGTGCCTTCTCTGATGCCGGGCTATGAGGACGAAGTGGTCATGGCGGCCGGAACGTTTGTGCAAGGCGCCAGCATCGAGTTGTCGGCGGACGGGCCCCTCCGTGCACCGTACATTGCTTACATGCAGGGAGGCCTCACCTATTCGCATGTGAAGATCGGTATGGCAAAAGCCATGGATTTCATGTTGGAAACCGGACAGATGCATGGGAAATTGCAGGTGTGAAGGGAGAGCTTGTTATATGGACATGCTTTGGTGGGCAATTATTATTTTGCTCTTTATTCTTGCTTATGTCGGCCTGGTTGCTCCCGCTTTGCCGGATGCCCCGCTGATGATTGCAGGGTTTGCGGTTTATCACTTTTTGATCGATTCCGAACCGTTGGGGTTCGGGTTTTGGATTACCACGTTGATCCTTGCCATCTTGCTGATTCTGGTTGATTATTTTTCCAGCAGTGTGGCGGTGAAAAAATACGGCGGCAACGCGGCTTCCGTCACCGCCGCGATTGTGGGGGTCGTTCTTTTCCCGTTTATCATGGGGCCGGTCGGCGTGATTGTCGGACCTTTTTTGTGTGTGTTTGTACTCGAATTGCTGCTTAAAAAATCTTTTTCCAAAGCACTTTATATCGCCTATGGGACCTTGGTCGGTTTTTTGGGCGGGATTTTGATGAAATTGATTGTCATGACCGGATTGATTATCTGGTTTTTGGTGTTGGTGTTGTTTTGATGAAAAATCGTTCTTCCTCCGGGGAGAACGATTTTTTTATACCCGCCTGAAATGATGTCAGAAAACCTGACATGTATTGACTAAAAAAGCTGACATGAATAAAATAGGATTATGAGGTAACAATCCCGAAAATGAAGGGAGCGTTTCCCATGGATTTTCGTGATGAAAAAAGACGGAACATGCCGCTTTTTTCGATGGGGATCGTGACGAAACTGACGGAGCTGAGTCCGAGACAAATCCGTTATTACGAACAACAAGGCTTGATCCGTCCCCAGAGAACCAAAGGGAACCAACGATTGTTTTCTTTCAATGATGTTGATCGGTTGCTGAAAATCAAGTCCTATCTGGAAAAAGGGGTCAACATCGCGGGAATCCGGGAACTCTTCAGTCAGTCTGAACGGGTTCAGGAAGCGGAGAAAGAAGAGCGTAAGGCAAGAAAAGAGCTGTCGGAAAAAGAATTGAGACGCCTCTTGAAAAGACAGATCATCCAACCCGGGCGTCCCGGGCGGCTTCCGCTTAATTACGGGGAGTTGAGCCGTTTTTTCCGACACTGAACAATTTTTCAGAAGGGAGAACGAACTGTGGCGAAGCATACCAAAGAAGACATTATGCGAATTGTGGAGAAGGAAAATGTCGCTTACATCCGTCTGATTTTTACCGACTTGCAGGGAACCATTAAAAATGTGGAAATTCCACGGAGCCAATTGGAAAAAGCATTGGACAACAAAATGATGTTTGACGGTTCCTCCATTGAAGGGTTTGTGCGAATCGAGGAGTCGGATATGCTGCTTTATCCGGATTTGGATTCATTCGTGATTTACCCGTGGGGAAATGACGTGAACGGCAGCAAAGTGGCCGGGATTATGTGTGATATCTACAATCCCGACGGAACGCCTTTTGAAGGGGATCCCCGTGGTATCTTAAAACGTGTTCTCAAAGAAGCCGAAGAAATGGGATTCACCTCTTTCAACGTGGGAACCGAACCGGAATTTTTCCTGTTCAAAACCGATGAAAACGGAGAGCCCACACTCAATTTAAATGACAAAGGCGGATACTTTGATTTGGCTCCGGTGGATCTCGGGGAAAACTGCCGTCGCGATATCGTGTTGACGCTGGACAAAATGGGCTTTGAGGTTGAAGCTTCCCACCATGAAGTGGCACCCGGGCAGCATGAAATCGACTTCAAATATGCGGATGCGGTGACAACGGCCGATCATATCCAATCATTTAAGCTGGTGGTCAAAAACGTGGCACGCCGCTATGGATTGCATGCGACCTTTATGCCCAAGCCGCTTTATGGTGTCAGCGGTTCCGGTATGCACTGCCATATGTCGCTGTTCCGCGGCAAGGAGAATGTTTTTTATGATCCTTCCGATGAATTGGGCCTGAGTGAAACCGCCCGGCATTTCCTGGCGGGGATTTTAAAGCATGCCCGTGCATTTACAGCGATCACCAACCCGATTGTCAACTCTTACAAACGGCTGGTTCCGGGTTATGAAGCGCCTTGTTACGTCGCATGGTCGCCGAAAAACCGCAGTCCCTTGGTGCGGATTCCTTCTTCCCGCGGGTTGAGCACACGTATTGAGGTTCGTTCCCCCGACCCTGCGGCCAATCCTTACCTGGCTTTGGCGGTGATGTTAAAAGCCGGATTGGACGGAATCAAAAATCAGCTCCCGCTTCCGGAACAAACGGACCGGAACATTTATGTCATGGATGAACAAGAACGGCGTGAAGCCGGCATCGAAGATCTGCCGTCCAGCTTGAAAGAAGCCTTGATTGAATTGCAAAACAATCCGGTCATGGTGGAAGCATTGGGTGAACACGCGCTTTCGCACTTTTTGGAAGCCAAGGAAATTGAATGGGATATGTTCCGGACCAATGTCCATCCTTGGGAACGCGAACAATATATGCATTTGTATTGACCGAAAGCCCCCGGTGGAACCCGGGGGCATAACATTTTTTTCTTCATTTTCGGAATAGTATATCGGGGATGTATCCGGAAATGAAGGGAGCGAAACGGTGAATGGCTTTTACGTTGTGTCTGGCAGGGGCACCGGTGACGGCAAAAGCATCTGGAAATCAGGCGGGAGCCCTGGCTTGTACGTTTCAAGGGGCTTGGCTGGAAGATGCCAATTGCACTGCCAACCGGTCCACGGTGTATACCGTTGGAAACGGCGCGCTTCGCACAGCCAGCGGTGCCGGAAATTTCACATGGGCCTATCCGGCTTCCAACAGTTTGGACAGGGCGTTCAGAGTTTGTGTTTACGATGAGGATTATCAGTTGGAAACCCAATGTTCCCCTTGGTGGTGATTTTTTCGTTTTCCGGCACCTGTTCCACCCGCGGGCAGGTGCTTTTTTTGTCGGCTCCTTGCCAAGCGGCGGGGCATAAGATTGTGCTATAATGAAAAAAATTGGAATGAAGCGAGCTGAAAGCCCTTGATCGATCTTCGCAAAATGTTTCATGGAAAAAATCTGACGGAATTGGATAAACAAATTTTGCAATACATCATTGAACATATCGACACCGTCTTGCAAATGGGCGTCCGCAAGGTGGCCAAAGCCAATTACACTTCGCCCGCCACAGTCATCCGTTTGGCCAAAAAGTTGGGTTATACCGGGTTTGTCGATATGTATTACCAGCTTCTGCCGCAAGTGAAAAAGATGGAAGTCCGGCCGGGCGGGAGAATGAAAGACTTTCCGGAAATCCGTCCGCAAGATTTTTTTCAGTTCAACACCATGGACGATATTGAACGGTTTATCGATCGGGTGCTTCATTTGCGGAAAAAATTGATTTTTATTTATGCCACCGGATTTTCTGCGATCGCGGCGGAATATTTGTATAAAAAGCTGTTGGTGTTGGGAAAGAAGACGATTATTGCCAGCGGGACGGACTCCGTCGGGGTGTTTGAAAACAATTTGGAAGACATCGGGGCGTTGGTGGCGATTTCCAAATCCGGCGAAACCCAGCTGGTGATTGATAAACTGAAGGCGGCCAGAGAACATCAAATCTTTACCGTGTCGTTCACCAAAGAAACGGAGAACCGGGCAGCCGAACTTTCGGATCTCAATTTTAAAATCGTGGATAATTATGAGCTGGATGACCGGAATATGTTGCCAAACATCTTTTTTCCCCGGTTGCTGATGCTGTTTGAACTGATTTTCAAAGAGTATTTCGACCGGATGTCACCCAAAGGCGGATGAAACGTGTTTCAACTTGTGAAACACGTTTTTTTCGTTTGTCAAAACATATACAAGAAAGCGCTTTCTCTATAAACTGGAGACAAGTCCGAACCACTGAACTGGAGGTGTCACCATGAAGGCAAAGCTGATGGAAGCCATGCAACGGTTTTCCAAAGCGATGTTTATTCCCGTCCTGATTCTGCCGATTGCGGGGATATTGATCGCACTTGGAAATTTGTTGACCAATCCCAAACTCTTGGAAACCGTTCCTTTGATGAATAACCCGGTGACAACCGGATTTGGCTCCATATTGGCCGGATCCCTGGTTTCCATTCTGGGCAATCTGGGGCTTATTTTCTGTGTGGGAATCACGGTCGGCCTGGCCCGGAAAGAAAAGCCGGTCGCAGGATTCACCGCCTTGCTGGGTTATTTGGTCTTTGTTCACGCGATGAATAAGTTTATGGAATTGAGCGGATTGTTGGTGGAGGCGGAATCGCTGCAAGGAACGGGGCAAACCCTGGTTTTGGGCGTGCAAATTCTGGATATGGGCGTTTTTTTGGGAATCATTCTCGGAATTGTAACCGCTTTTATTCATAACCGGTGGATCGATACGGAATTCAAAGGCGCTTTTCAAATTTACGGCGGATCCCGTTTCGTCTTTATCCTCCTGATTCCGGCGGTGGTGTTGCTGGCCGGGGTCTTGATGTACATCTGGCCTTTTTTCCAAAGCGGGATCAACAGCTTGGGGACGCTGATTCAACGGAGCGGGAGTTTTGGGATCTTTTTGTACGGGGCATTGGAACGCTTGCTCATCCCGACCGGATTGCATCACCTGGTATATACCCCGTTTTTGTACACTTCACTGGGGGGAGTGGCCGAAGTCGGCGGACAAATTTATGAAGGAGCCCGGAACATCTATTATGCGGAGATTGCTCATCCGGGGGTTACCCGGCTTTCGGAGAGCGTGATCTGGGATGCGCGCGGGATTTCCAAGATGTTTGGGTTGATCGGGGCGTGCCTGGCGATGTACCACACGGCCAAACCTGAAAACAAAGCCAAGGCCAAAGCGGTGTTGCTTCCGGCGGCGTTTACTTCCTTCATTGCCGGGGTGACCGAACCGATTGAATTTTCCTTTATGTTTGTCGCGCCGTTGTTGTTTGCGGTGCATGCCGTATTCAGCGTGCTCAGCATGGTGGTTCTCAACCTGCTGGATGTGCGCGCCATCGGGCCCAACGGGATGATTGACTTTTTGCTGTACAACCTGCCGCTGGGTATCCAGAAAACCAGTTGGCCAATGTATGTGTTGGTTGGACTGATGTTCTTTGTCATTTATTACGTGGTTTTCCGTTTCCTGATCGTCAAATTCAATTTTCAAACGATCGGCCGGGAAGAAAAAGGACAAGAAGTCCGGCTCTATTCCAAAAAAGATTACCAGGAGAAAAAGAAACAAACCCAAACCGTAGGACAAGCTTCAAAGGAAACATCCGATGTGGCCGGTGTCATTGTCCGCGCGCTCGGCGGGGCGGAAAACATCCAAGCGGTGACCAATTGTTACACACGTCTCCGGCTGACATTGGTTCATCCGGAAAAGGTGGATGAAACGGTTTTGAAAAATGAAACCGGTGCCAGCGGAGTGATTATTCGCGACCAAAACGTGCAGGTGGTTTACGGATTGCAGGTGACAAACATCCGGAAAGCCGTGGACCGGTATTTGGGACGCGCCGGTCAAGAATGACTCACAGAAAGGAAGAGATGGTGATGAAAAAGTTTTCCATTGTGATTGCAGGCGGAGGAAGCACTTATACACCGGGAATCGTCATGATGCTGTTGGATCATTTGGAAACGTTTCCGATCCGGAAATTGAAATTATACGATAACGATGAAAAGCGGCAACACATGATCGGGGAAGCCTTGAAAATCGTGTTGAACGAACGTGCGCCGGAGATCGAGTACACTTACACCACCGATCCGGAAGAAGCATTCACGGACATCGATTTCTGTTTTGCCCACATCCGGGTCGGGAAATATGAAATGCGCGAGCTGGATGAAAAAATTCCGCTCAAACACGGGGTGGTCGGCCAGGAAACGTGCGGTCCCGGAGGCATTGCTTATGGCATGCGTAGCATCGGACCGATGATTGAATTAATCGATTGGATGGAAAAATACTCCCCGGATGCGTGGATGTTAAACTACTCCAATCCGGCCGCCATCGTGGCCGAAGCCTGCCGGATTCTCCGCCCGAATGCCAAAGTGCTGAACATTTGCGACATGCCGGTGGGAACCCTCCGGAGAATGTCCCAGATTATCGGCAAAACGCCGAAAGAGTTGGATGTTCGTTATTTCGGGTTGAATCACTTCGGTTGGTGGACCAGCGTCAAAGACAAGGAAGGCAATGAATACCTGCCGCAAATCCGCGAATATGTGAAAGAAAACGGGTATTTGACCCAGATCGAAGTCGATACCCAGCACATGGATCCCAGCTGGCAGGAAACGCATAAAAAAGCGAAAGATTTGTTGGCGGTCGACCCGCGGTTTTTGCCCAACACGTATTTAAAATATTATTTCTATCCCGATGAAGTGGTTGAAAGTTCCAACCCCAAATACACGCGGGCCAATGAAGTGATGGACGGGAGGGAGAAGAAAGTCTTTTCCACCGCCAGAGAAATCATTGAAAAAGGGACGGCGGCAGGAAGTGAATTTGCCATCGGCAGCCATGCAACCTTCATCGTCGATCTGGCCCGGGCCATTGCATTTAACACCCATGAGCGGATGATTATGATTGTCGAAAATAACGGTGCCATTGCCAACTTTGATGATGATGCCATGGTTGAGGTCCCGTGCATTGTCGGCTCCGACGGCCCGGAACCGCTCAGCCAGGGAAAAATTCCCGAATTCCAAAGAGCTTTGATGCTTCAACAAGTTACCGTGGAAAAAATGGTGGTGCAAGCCTACATCGAAGGCAGTTACCAAAAATTGTGGCAGGCTTTGACCCTGTCCAAAACCGTCCCCAGTGCCAAAGTGGCGAAAAAAGTGCTGGATGACCTGATTGAGGCCAACCGGGGATACTGGCCGGAATTAAAATGACGTTTGGCCCGGATTTTGGATCCAAGCCTGAAAAGGCTTGGATGTTTTTTGTTTTGAAACAAACGCTGATGCGAAAGGGATGGCGATTTTCAAAGAGAGAACGGACGGAAGCAACCGAAGCGGCAACGTCCGTTCTCAAAATGCGTTTCAACTTTCTTGACATCGCAAATAAGTTTGCAACACAAACGAAATTCAAGCAGAATGATTGATGACAAGCGGGGGAAGGTGCCGGGGATGAAGAGGCGGAGTTGAGGAAACCGCCGGGGATGTGTATGATAGCATGTATGTACAAACAGGAATGAGAAAGGGGAGGGCCATGGAAAGAAACCGGGCAGAGATCAAAGCGATTGCACTGGACATGGACGGGACGATGTTAAATGTAAACAGTCAGGTGGAGCCGGAATTGGTTGATCTGTTCAGACAACTCAAGCAAAAGGGGATCCGGATTTTTGTCGCGACCGGACGGACCTTTCCCGAAGTCCGGCGGGTTCTTCCGGAACATCTGGAAATCGACGGGGTGGTGACAGCCAACGGGGCGGTGACTTATATCGGGGACCGGATCATTTCCCGCCGCACTTTGGATCCGTCCGTCGTGAGAGAAGCCGTTTTTCAAGCCGGACAAAAGAACATCTATTATGAAGTTCACACGGCAGAAGGCGTCCGTTTTGCTCTGGCAAAAGAGAAAGAGCGGATGATGGAGGAAGTGTTCCGGGAAAAGCCTGACACATTGTTTGCAAATGAATGGCTCTCCCGGAAAGCCGCTTTAAAAGAAAAAATGGTTTGGCTGGATCATTTGGTGGATGAAGATATCGTCAAAATTTATTTTTTCAGCATGGAACCGGAGAATATCCGGAAATGGCGAAATGAATTGGAGCAGATGAAAAATCGCACGCCGTTTACCCTTTTGTCTTCTTCCAAGCATAATTGTGAAATCATGGCTGACCAGGTTTCGAAGGCATACGGGTTGAACTTGGTGTTAAAAGAATATCAACTTGCGCCTGCCCATTTGATGGCCGTTGGCGACAGCGAAAATGACCTCTCGATGTTTGCATTGGCATCCCGTGCGGTGGCCATGCAAAACGCAGCGGATGAAATCAAAGAGAATGCGGATGAAGTGACGGAACTTCCGTATGACGAGAATGGATTATACCATTTTCTGCGAAAAGAATTTTTCGGTTCGTAAGCGCAGAGGGTTGCCGGCAGGAAAAGCTGCCGGCAACTTTTTGTATGTGGAGCTGTTTTTATGCGGTTTCCGGACAATAAAAAAGGGCGGCTTAAGGGCCGTCCGTTTTTTATGATCATAAACTGCAACGGGGGTTGGAGCCGATTTTAAACACCAGCAGGCTCGCATCCTGAAGCGCTTCAATGTCATGCTTTTCAAAGGGTTTCAAATGCAGCAACGATCCTTCTTTCATATGGTGCCGATTCTCTTCCACACCAAAGAGGACTTCCCCGCTTGCCACATAAACAATCACATCGGTATTGGCATGATGGCGGGGGACGGTTTCCCCGGCTTTGATTTGCAGATTGATCGCCATTCCTTGATCAAAGTCGAGTACTTTGTTGCGAGTGACTCCTTTATCAATCGGTCCTTCTTTTTTGATTTCAACCAAAGACACAGGCGGAGCCTCCTTCTTTCAAAACTTAATTTGAGTATGGCATAGATTCGGGGCATGTGATGTGATATATCTCACAAAAAAGATCACGAGAGTTTGTCTTGCAGGGTTTTCAAGTCCAGAATTTTGATTCCTTTCCGGCTCACATCAATCAAGTTCTCCTTTTTTAACTGGTTTAACCCCCGGTTGACGGATTCCCGCGACAATCCCAGCATATTGGCAAAATCCTGGTGGGTTAAAGGCAGGGAAATGAAGATGCTTTTGTCTTTGGAGACGCCGTATTCATCGACGAGCCTCAGCAAAGTCATGACCACTTTGTGATAAACATCGCCGGAAATGACTTCCTGAAGGCGCCTTTGCAGTTGCAAAATTTTAACGGCCATGATTTTCATGACGGATTTGGCGATTTGGGGATTGGCATCCAGCAGGCGGTCAAAGTCATGGATAGGGATCGCCAGGAGTTTGCTGTTTTGGATGGTTTGTGCTGTTCCGGGGTAGGGGGAGTCGTCAAAAAAACCCACGTGAGGAAACATATCGCCTTTTTGGAGGATCGAAATCACTTGTTCGTTTCCTTGATTGTCCACTTTGGTGACTTTAATGACTCCCGACTCGATGAAGTAAACCGTTGTTCTTGGTTGCGCTTCCATAAAAATGGTGGAATGTTTGGGGTATTCCTTCAAGGAGGTGATTTGTGCGATTTTCTCCAGCTCTTCGATGTTCAGGTCCCGGAAGAGAGGCACCCGTTTCAAGATTTCAACCTTGCGGTTCATAGTTACCGCCTCCAGAAATGAAAATAAATGGTGTTTAATTTCATTCAAAGGGATGCGTTCCCCGTTCGTCAACAACAAAGCGTTTTCGGTTCTGTCAACGTTCTGTGAACACCTGCATTTTTGTGATCCAGATCACATTTTGTTCATATGACAATGTTTATAATGTTTAATAAATCATTTTATATAATTTGTTATTTTTTATGTTGTCGGGATGAAGAAAAACACACGACAAGGAAGCATGAATGGTTGCCTAAAATAAAAGGGGATTTTTGCTGGAGGAGGCTTTTTATAAATGCTGGATGCAAAAACGATGGAAATCATTAAAACAACGGCTCCGGTACTGAAAGAAAACAGTGTAGCGATCGGAAAACGGTTTTATGAATTATTGTTCACCCGTCATCCGGAGTTGCTCCATATTTTTAACCACAGTAACCAAAAACGCGGTTTGCAGCAACGTTCCTTGGCGCATTCCGTCTATGTGAGCGGGGAGCATATCGACCGTTTGGAGGACATCCAACCACTCATCATGAAAATCGCCCATAAACACCGGGCGCTCGGGGTCAAACCGGAACAATATCCTGTGGTGGGCGAAACGCTGATTGAGGCTGTCAAAGATGTGTTGGGGGACGGAGCGACGGATGAGATTATCCAGGCTTGGGTGAAGGCCTATGATTACATCGCCAATCAATTCATCGGAATCGAACAAAAGTTGTATGAACAAACGGAATCGGAAGAAGGCAATTGGGAAGGTTTCCGGGAATTTGTGGTGGTGAAAAAAGTGAAGGAGAGCGAGGTGATCACCTCCTTCTATTTAAAACCCAAAGACAATAAGCCCCTTGCTTCATTCCGTCCCGGACAGTATCTGACCATCCGGGCAAAGATTCCGGGTGAAAAATACATGCATCACCGCCATTACAGTTTGTCCGATGCTCCCGGAAAGGACACCTACCGGATCAGCGTGAAACGGGAAGATGTCCGGGATGGAAATCCTCCCGGCGTGGTTTCCACCTGGTTGCATGAACAGGTGCAGGAAGGGGATGTGCTTGAATTCAGTTCGCCGGCTGGTGATTTTGTTTTGGATACCGAATCCACGAACCCGGTCGTGTTGATCAGCGGAGGTGTCGGGTTCACTCCCCTCATGAGCATGTTGAACACCTTGGTGGAAAAACAGCCGGACCGGGATGTAACCTGCATTCATGCGGCCATAAACGGCAAACACCATGCAATGAAAGAGCATGTGGCCCGTTTGGCGGCAACTTGTGATCGGCTGAAATCTTATGTGATTTATGAATCGCCGACCGAAGAGGACCGAGCGGAAAAATCCTTTGACAAAGAGGGATGGATTGACAAAAAGTGGCTGGAGTCCGTGGTGGACCATACCGGTGCCGATTTTTATTTGTGCGGCCCGGTTCCGTTTATGGAAGTTGTTTATTCGGCGCTCCGAGAGATGAATGTGGATGAAAGCCGCATTCATTATGAAGCTTTTCGCCCGCTTGACACCATGGATGCCGGTTCATGACGGGAAAATGAAAACCACCCGATACGGGTGGTTTTTTTGTTTGCAGAGCTTATAAGCCTTGGCAAAGAAAAGAAATTGACAAAGTGTCAAACATTTTGTCGCCATTTTGTGAACGACATCCGTTATGTGATGTGAATCACAAAAGCGGCGCAACGCCCCGTTTAAAGTGAAGGTGTAACAAAAAAATGAAATACAGGCAGGAGGAATTCATACCAGAGACACAAGGACATGAATCATTTTTATGAACCCTTCATTTGTGATGTTTGTCACAAAATCTGGAGAATTGGACTCATCAGCCAGTGACATTCCAAGTGTTATGGGAGGGAGAATTGTTGAAGAAATTGCGGGCATCCAACAGACTGAGAAAGCTTTTGCCGGTACCGGGATTTGTTCTCATTTTATTGCTGACAGGATGCGATGACCGTTATGTCATGTTTAATCCGTCCGGACCGGTCGCCATGACTCTGTATGATCTGATCAAGTTGTCCGTTCTCATGCTGTCTTTGATTGTGATCCCGGTGATGCTGTTCTTCTTCTATGTGGTTTACCGGTATCGGGACAAACCGGGAAATAAAGCTTCCTACCGTCCGGAGTGGGATGACAGCAAAATCATGGAGATCATCTGGTGGACCATTCCGGTGGTGATTGTCGCCATCTTGAGTGTGGCAACGGTTCGGGACACATATGGATTGACGAAAAAACCTTCACCGAACAAGCCGATCACCATTGAAGTGGTTTCATTGGACTGGAAATGGCTTTTCTTGTATCCGGAACAAAACATTGCCACGGTGAACCATGCACCCGTTCCGGCAGGGGTTCCGGTTCAATTTGTGCTCACTTCAGATGCCCCCATGAACTCCTTCTGGGTTCCGGAATTGGGCGGGCAATTGTACACCATGCCGGGAATGGAGATGCCGCTTTGGTTGCAAGCCGACAAACCCGGTGTGTATGAAGGAAAAGGAGCCAACTTTACCGGAAAAGGCTTTGCCCACATGAATTTTGAAGTGATTGCCAAACCGCAGGCGGAATTCGATGCATGGGCAGAAAGCGTGAAGAAATCCGCACCTGCTTTCACAGGAGAAAAATATGATCAATTGAAACAGCCGGGTTTGGCAAAAGAGCAGTCGTACTCGTCTTATCCGCCCAAACTTTACAAACAAATCGTGGATAAAAACGGAGGAATCTATTGGGACAAACATGATATGGACGTTCGCCATGAACCTGTTCATGAATAACGGAAAGGTGGGATCCAACAGTGATTCAGCAAATCGTTGATTACCTGAAAAACGCCCACCCCATGATTCAAATGTCAGCGGTTGCTTCCTTGGTTGTTCTGCTCGGGATCATCTTTGTGCTGACATATTTTAAAAAGTGGGGCTGGTTGTGGCGCGAATGGCTGACCACGGTGGACCATAAAAAAATCGGGGTGATGTATCTGATCTGCGCCCTGATCATGTTGTTCCGCGGTGGCGTGGATGCGCTGTTGATGCGGATTCAGCTGGCGGCGCCCAATATGCACTTTCTCGATCCGCAACATTATAACGAAATTTTTTCCACACACGGAACGATCATGATCTTGTTTGTGGCCATGCCGATGATCTTTGCCATGATGAACATTGCGGTTCCCCTTCAAATCGGGGCACGCGATGTCGCATTTCCTTTCTTGAACGCACTCAGTTTCTGGTTGTTTTTCTTCGGTGCGATGCTGTTTAATCTTTCCTTTATCCTGGGCGGTGCGCCGGACGCCGGATGGGTGGCTTATCCGCCGTTATCCGAAGTGGATTTCAGTCCGGGCCCCGGAATCAACTACTACCTGATGTCGCTCCAGATCTCAGGAATCGGAAGCATTGCAACCGGTGTCAACTTTATCGTCACGATCCTGAAAATGCGGGCACCCGGCATGACCTTGATGAAAATGCCGCTGTTTCCCTGGTCGGTTCTCGGGTCTTGCATCATCATTATTTTCGCTTTCCCGGCCTTGACCGTGGCTTTGGCGCTTCTCGGGTTGGACCGGATTTTTGATACCCATTTCTTTACGATGGATGGCGGCGGGAACCCGATGATGTATATCAACCTGTTCTGGATTTGGGGTCACCCGGAAGTATACATTGCCATCCTGCCGGTTTACGGAATTTTGTCGGAAGTGATCAGCACCTTCAGCAAGAAACGCATTTTTGGTTACAAATCGATGGTGATGTCGCTGATGCTTATCAGCGTGATCAGTTACTTCGTCTGGGCACACCATTACTTTGTGATGGGGGCCGGCCCCGGAGTCAACAGTTTCTTTGCCGTCGCTTCCATGGCGGTCGGAATTCCGACGGGTGTGAAGGTGTTTAACTGGCTGTTTACGATGTTCAGAGGACGCATCCGGCTGACCCTGCCGATGATGTGGGCACTTGCTTTCATTCCTTGCTTTGTCGTGGGCGGGGCAACCGGAATCATGCTGGCGATGGCACCGGCGGATTATCAGTTCCACAACAGTTATTTCCTCATTGCCCACTTCCATCAAGTCTTGATCGGAGGAGCCGTCTTCGGACTGTTGGCCGGAATGTATTACTGGTGGCCGAAAGTGTTTGGGTTCAAATTAAACGAGAAGCTTGGAAAATGGGGATTCTGGTTCTTTAACATCGGCTTTTATGTCTGCTTCATGCCGCAATACGCTTTGGGATTCATGGGCATGACCCGGCGCGTGTACACTTACCCGGCTGAATCGGGTTGGGGAGATCTCAACTTTGTCTCCACCATCGGTGCCTTTATGATGGGAGCCGGTTTCTTGTTCATCGGTGCTCAAATTGTCTACAGCTTGTTCCATAAAGAGTTGGACACAACCGGAGATCCGTGGGACGGCCGCACCCTCGAATGGTCGATTCCTTCCCCTGCACCGCATTACAACTTTGCCCAAATCCCGGAAGTGGGCAGTTTTGACGCCTGGTGGGAGAAAAAACGGGGCAACCGGGACAGTTTGAAAACCGGTTCAAAACTCAAACCGATCCACATGCCGAAAAATACGCCCATTCCGTTCCTCATGTCGATCGCCTTCTTTATCGGCGGATTCGGATTGGTGTTCAGTCAATTCACCATTGCGGTCATCGGGTTGATCGGGATCATTGCATGTATGTTGTGGCGCTCGTTGGAAAAGGATGAGGGTTATTTCATTCCCGTCGATGAAATCAAAAAAGCGACTGCCAAGGGGGTGTCGTAAATGGGAGCGACGGAACAACAAGCTGTTTTGGATCACCAAGCGCATGACCATGAACATCACGAGGATCAAGACCGTTATGTTCTGGGCTTTTGGGTGTTCCTTGCGTCGGATTTGGTGCTGTTTGCCAGCATCATCGCCACTTATTTTGTCTTGCGTACGCATACCGACGGGGGCCCGTCCGCGGCCGAACTGTTTGAAATTCCGCTGTTCACGCTGGAAACCATCCTGCTTTTGACCAGCAGTTTCACATGTGGTTTGGCGATGCACGCGATGAAAAACCACCGGTTGAATGCCATGATCGGGTGGATCGCCGCCACGATCGCCTTGGGATTGGCGTTTGTCGGGATTGAGGCCAGTGAGTTTGTCAAATACGCTGCCGAAGGGGCCACCATGCAGCGGAGCGCGTTTTTGTCCGGATTTTATACACTGCTTGGAACCCACGGACTTCACGTATGTCTGGGCATGGGTTGGCTTGCCTCTGTGATGGTTCAGCTGAAGCGCCAATCCATCAATCCGGCCACGGCAAGAAAATTCACAAACGCGGGACTCTACTGGCACTTCTTGGATGTGGTGTGGATCCTGATCTTCACGCTGGTTTATCTGGTGGGGGTGATTGAATGAAAAAAACAAGCAGTTTCCCCTGGTCTCATGTGATCGGATTTATCGGATCGATCGTGCTCACGTTTTTGGCCATCGGGGTTGCCGTCCGTTCCCCGCTTTCCATGGTTGGGACCTTGACGGTCTTGCTGGTGTTGGCCGCCTTGCAAATTTTGATTCAGTTGGTTTTCTTTATGCACATCAATGAAAAAAAAGGGCCGGCTTATCATACCATCGCCATTTCGCTGGGATTTGTGTTTACGTTTGCCGTGATAGCCGGATCCATTTGGGTGATGACCTTCAGTTACTTTTAATAAGGGGTGAGGAAAATGCCGGCTCCATCGGTTCATGCGGTTTCATCAGACACCCGGTTGCTTCCTGCGGTGATCAATAAGGTCAAGGCGTATGTGGCCTTGACCAAACCGCGGATTGTACTCCTGATGGTTTTTTCGGCGCTTTGTTCCGCCATCGTTGCCGAGGGGGGATGGCCGGAGGTTTGGACCTTGGTTCATATGTCGGTCGGTTTGGCGTTGTGTGCCGCGGGGGCATCCGCTTTTAACATGTGGTACGACCGTGACATGGATCAACTGATGGAACGCACCATCCATCGACCGCTTCCCACCGGGCGGATCCGTCCGCAATCCGCGCTTTGGTTCGGATTGGGTCTGGGCATGTTGTCCCTGATTTGGTTGGCCGTTTTCGTCAACGGGCTTTCCGCCCTCCTGGCGCTGGCCGGCTACTTGTATTACACCATGATCTACACGGTGTGGCTGAAACGGAGAACACCGCTCAACATTGTCATCGGGGGCGGAGCCGGCGCGCTTCCCCCCATGGTGGGATGTGCTGCGGTGACGGGGGAAATCGGTTGGACAGCCGTGTGGTTGTTTGCCATAATCTTCGTTTGGACGCCGCCTCATTTCTGGCCGCTGGCCATGGTGAGAAATGAGGAGTATCGCTTGGCGCACGTTCCCATGATGCCGGCAGTCCGGGGTTTTCGTGTCACAAAACGGCAATGTTTGGTTTATACGGTGCTTCTTTTGCCTGCCACTTTGGGATTGGCGATGACCGGAGCGGTTGGCGGTTTTTATCTGGTCGCGGCCGTCCTGGCCGGATTGGCTTTTTTGGCGGTCCAGATTCGGATGTGGCGGGAAGCGGATGATGAGATCCTGTGGGCAAGACGGGCTTTTCGGGCTTCATTGGCTTATTTGACGGTTTTGTTTGCTGCCATGGCCATGGATGTATTGATATAAAATTTCTCCTTTTTTTCTAAGAAGGGAGGGAGAGGATGCAAACGGCAACCATTTTCCGCCATCGGAAAAAACTTTATTCGTTGGCGCTCATCACGCTGATCGGGTTGTTTTTGGTCAATCTCATTGGTTTTGTCGACACCAAGACAGGGTCCACTTTTGGTTGTGGCGAAGAGTGGCCCCTCTGTAACGGGGAATGGATTCCAAGCAAATGGGATAAACATGTTGCCATTGAATACACCCACCGGGTGAGTGTCCTGGTTGACATGATTCTCTTGATCGTGTTGACCGTCCTCTCCTGGACACGATACCGGGACCGGAAAGCGATGCGAATCATTATCGGCATTTGTTTTTCGGGATTTATCGGGGAGTCGGTATTGGGCGCGTTGACGGTTCTTTTTGACAACCCGCCGTGGGTGTTGGCTTTTCATATGGGGATGGCTCTCACTTCGGTGGTGGGATTGTTTTTGTGGACGGGATGGTTGTACCGAAACGAGGTGGACCCGTCACGCCACCAAAAGGAAGCGGATCCGGCCGGGATGAAAAAGCTGGGACAATGGGCCTGGTTCGCGGTGATATATTTCTTCTTCGTGGTGTACTTCGGGGCGTATGTCTCATTTACCGGTTCCGGTTCTTTCTTTCAAGGATGGCCGGTGCCGACGGAACCTTATGAAATCGCCCGGCATGCGCTGGTGATTGACTGGGTTCACCGCCTTTCAGGTTTTGTCCTTCTTTTGATGATCCTCCGGCTGAGCGTGTTGGCTTACCGGATGCGTCAGGAAAGAAAGGATTTGTGGGGCATCTGCCGGGCCATGTTAATCCTGATCGTCTTGCAGTTTTTAAGCGGCGGTTTGTTGATTTACACCGGACTGAGCCTGGCGGCCTTCCTGCTTCATGTTACCTTTCCCAGCCTGATCATTTGCCTGTTGAGTTGGCTGGCTTTTAAAACCGTGCCACAGAGAAGAAACAGACTATGAAAGATTGACAAGAGTAAGGCCACCTTATCATGGTGGCCTGTTTTTTATTTGGGGAAAATTATCTTTCTCTTTCCATCGCATCCCGCTTGGTCCGGTGAACCGTGCCGAACGGATGCTCCGGCGGGGCGTAAATGCTGTAGAGTTTCAAAGGCTGATATCCCGTATTGATAATGTTGTGCCATGTGCCGGCCGGAACCATGATGGCATCATCTTCAAATACGGCTCTTTGGAAATTTAACCGGTCTCTTTGCGGTCCCATTTGCACCCAGCCCTGGCCTTCTTCAATGCGCAAGAACTGGTCTACATTGCGGTGGACTTCCAGGCCGATATCTTCCCCGGGGTTAATGCTCATCAAAGTCACCTGCAAATGATCTCCCGTCCAGATGGCCGTGCGAAACGTTTGGTTTTGTTCAGCCGCTTGTTCGATATTGATGACAAAAGGTTTTTTTCCCTGATCTTCGAACCGGACCCTGTTGGGGTTGGACGGATCAGGCATTTGAGCATAATAAGGATACGGATAAGGAGGATACATGTAGTGATACACATTGCTCATCCCTTTCAAAGGTATTCATGCTTTTTTCAAAGGTGATTTCACATTATACATATGGGGATCCGTGGGCAGATGTTCCGTGTTTTTGAAATCAAAGAGTGAACACAAAAGAAAAAGACCTCGTGATGTGAGGTCTTTTTTGTCAAAATGTTTCAAATTAAATACAAAATGATTAAAATGATAAGCAAAACACTATCCAGGATACTAGCCAAAATATAGGCCGATTTGGGTTCCCAATTCAACTTTTTTCCAAAAGTGATAGCCCCGAATAGGAAGATCGATGACAGTACGGCGTAGATCAAAATTAAATATAGGATTTCTATCTTTATCCCTCCGTTTCTTTTACAGATTGGATCAGCAATGAAACCAGTTCCCAAAGAGTTATTCGTAAAAGGATCGGATTTTTTTGTCAAAATATTTTCTGTTTGGGAAGAAAACCATTTTGTTCATTTTTTTGGGAGGGACTTTCGGGATGTTTATGGTGCATGGTTTACTGACTTGTTTGTTTTTCTTTTGCTAAACTGAAAGTGGCAGAATTTGCGATTGTTCACGGAACAGGGTTGTATTCATTATTTACAAAAAATACGGAATAGGAAAAATGCGGTTCGGCAATAGTCCGATTTAATCACAGGTTTGGGAGGCGGAAAGATGAAAGTGGATTTTGGCCTGACTGCCGGCGATTACCGAAAGTACCGGGCGGGTTATCCGGATGAATTATATGACCGTCTTAAAAAGTACGGAGTGGGTCTCAAGGGACAATCCGTGCTGGATCTCGGGACCGGAACCGGGTATCTCGCCCGGAAATTTGCCAAGCAAGGGGCCCGGGTAACCGGAATAGACATTTCCAAAGAGCTGATCAAAGAAGCCCGGGAGCTGGATGAACAAGAAGGGGTCCATTCCCGATATGTGATCGCCCGGGCAGAGGCGTTGCCCTTTTCGGATTCCGAATTTGATGTGGTGACCGCCGGGCAGTGCTGGCACTGGTTTCAAGCGGATCAAGTGTTAAAAGAAGTGCGCCGTGTTTTGCGCTCGCACGGAAAACTGGCGATCATCCACCTGGATTGGCTGCCGCTGAAAGGAAATATTGTGGAAAAAACGGAAAAGCTCATCTTGTCGTATAATCCGGATTGGCAAGGGGCCGGGGGAACGGGGGTTTATCCGGCGTGGTTCACGCAAGTGGCGATCGGCGGATTTTCCGGTATCGAGTCTTTCACGTTTGATCTTATGATTCCATACAGCCAAGAGGCGTGGCGGGGGCGGATCCGGGCAAGCGCAGGGGTGGGGGCCAGTCTCCCGCCGGATGCAATCGCCAGGTTTGACCATGAGTTCAAAGAGTTCCTGGAAAAGAATTATGAGGAGACGCTTAAGATACCACATCGCGTTTTTTGTTTGGTTTGCACACCGAAGGAGGAACCATGATGGTTTGCATGGGAAGAAGAGCTTTCCTGTGGGGAAGCTCTTTTTTGTTTTGTGATCTTATTCACAAAGAGAAATCGTTTTTTTGGCTAAGATGAAGCTGGAAGGGATGTGATGGGATGAAGGCATGGGCAAAACCAAGGGATTACAACGTGAATCCGTTTATTGTGATATGGGAAGTCACCCGGGCTTGTGCGTTGAAATGTTTGCATTGCCGCGCGGAGGCCCAGTACCGCCGGGATCCGAGAGAGCTCTCGTTTGAAGAGGGAAAACATTTAATTGATGAGATTGCGATGATGGATTCGCCTTTGTTGGTCTTTACGGGCGGCGATCCATTTATGCGCCCGGATTTGTTTGAATTGGCCATATATGCCATTGAAGAAAAGAACTTACCCGTTTCCATCACCCCCAGCGCCACCCCCAGGGTGACCCGCCGGGCGGTGGAAAAAGCCAAAGAAGCCAAATTGTCCCGCTGGGCCTTCAGCCTGGATGGTTCATGTGCCGAGATCCATGATTTTTTCCGGGGAACCAAAGGATCTTATGATTTGACCATGAGAGGAATTCAATATCTGAAAGAATTGGGGATTCCGCTTCAAATCAATACCACTGTTTCCAAATACAATCTGGAAGATTTGCCGGAGATTGCGGACATAGTCAAAGAGATGGAAGCGGTGTTATGGAGTGTCTTCTTCCTGGTCCCCACCGGGAGGGGATTAAAAAAAGACATGATTTCCCCCGAGGAGCATGAATCGGTGATGAAATGGCTGTACGAGCTCAAAAAAACGATGCCTTGTGATATCAAAGTGACGGAAGCGCCGCACCATCGCCGTGTCTTTTTACAGGAGTCCCGGCGACAGCAGGAAACGGGCGTTTCACAAGCAGGCAAACGAAAAGATGTTTTGGGAAGAGCGCCGAAAGCCGTGAATGACGGAGACGGATTTGTGTTTATCAGTCATATCGGCGAAGTGTATCCCAGCGGTTTTTTGCCGGTGGTTTGCGGAAATGTCCGGTGGGAACCGTTGTGGAAAATTTACCGCGAGTCGCCCGTCATGCAAAACTTGCGGGATAAATCGAAGCTGAAAGGTAAATGCGGGGTTTGTGAATTCAAGGAGATTTGCGGAGGGTCCAGGTCACGGGCTTATGCGTTGACCGGGGATTATCTGGAGAGCGATCCCTCCTGCAGTTACATTCCGGCAGCGTTTCGGCCGGAAACATGAAAAAACCAAAGAAAAAGGGACTTCCAAGGTCGGAAGTTCCTTTCTTTTTTACCGGGTTACCCGGATGATTCCTTTGGCGCCTTTGGAAGCTTGCCGGAGCTGATGGCTGATGAAAGGATAGTTTCCTTCTTCCGTGACGGTTAGTTCGATCACGGCGCCCGCGCTCGGCGAAAGTTGCACGGTTTGCAGACCATGTAGCCGGTTGCGGGGATTTCCGTCCAAATAGACAGTATCCAGGGTGGTGCCGATCACATGGAAGGAAGAAACTTCATTCGGCCCGGCGTTGAGCACATACAACCGGATCCGTTCGCCGGCTTTTGCATGGAGCGGTTGTTCTTTCAAAGCATAATCGTTGCCGTTGAACACATTGTATTCCGGCATTCCGTTGGTCATGGCACCAAGGTCATTTTCTTTATACCATTCGCTTTGGACGATGACATATTCGCGGTCGATCGTTGCATCGGTCGGATAGCCGTTTTTGGGTTCAACGATGATCATGCCGAACATTCCGTTGGCAACGTGTTGCAGGACCGGGTCGGTTCCGCAGTGGTACATGAAGACGCCGGGGGTATCGGCGGAATAGGCGAAGGTGTTTTGCTGACGGGGCATCACGTTTGCAAATTTTTTGTCGGGGGCGGCATGGACGGCATGAAAGTCCATCGAATGCGGCAGGTTCGGATCTTTGTTTTTTAAGGTGAAGAAAAGTGTGTCCCCTTCTTTGACCCGGATCACCGGGCCGGGAACCGTGCCGTTAAAAGTCCAGGCGTTGTAATTCACGCCTTTGGAGATCTCGACGTCCGTCACTTGTGCGGTCATTTCAATACGGACTGTTTTCCCGTCCCGGGTGATTTTCGGCGGGATCGGCGATGCGTTCTCCCGTTGCACGGCAGGTTCGGTTTTCTCCGGGGGTGTTTCCGGTTCTTTTTTTGGCGGAATGGAACAACCGGACAGGACAAGCGATGAAATCACAAGAACAGAGAAAAAGTTTATAGTTTTCATTGATGATCACCACGTATTCTTTGGTCGTGTCAAATTCTGCAAATGTGCATTATTTCACAAATAAGTCGTTGAGACTGCCGGGATTCCCGGATTTTTGGCGGGATGTTTCTTTTGGTGCCCCGGGAGGGGCGGGCGGCGTTTGCCGGAAGACAAATGCTGCAGTGATGTGTTGATGAAGGACGGGTCAAAAGCCAAGCCATTCTCTTCCTTCCGGGGAAATTTTCTCCGGTGTCCACGGGGGATCCCAAACCACGTCCACTTCCACGCTTTCCACGGACGGCAATGAACTTAATGCGGCTTTTACCCCGCTGACGATGGTGTCATGAAGCGGGCATCCGGGGGTGGTCAGGGTCATCTCGATGAAAATATGGCCGTTTTTGATGTCCATTCGGTAAATCAGCCCGAGATCCACCACATTGACGCCCAACTCGGGATCATAAACTTCTTTCAGTCTTTCGATGACGGTTTCACGGGTTAAACACATGCTTTTTCCTCCTTTGACCTGAAAGTGAAGACGTTGCCGATGGTGGAGATGGCATACAGGCTGGAAAGCGAAAACAGAAGGAATCCGGCTTGAAAAAGAAAGGTGGATGATGTGACAATCCCGGGCACCGTTCCCAAAAAGGCAAAGCCCACAACATAAAGTGCCCGTTTCAATTTCTTCTCCTGAATCATTTCAGAAAGAAGCGGGACTTTGTTCTTTCCGACCTGGTTGCCGTATTGAAAGGTCCACCATAAAAAAGGCACGATTTTTGACAAATAACCGATGATCGTCAGGTAGACCCATCCCCATAAACCGATCCAGACGGTCAGGATGAAAAAGGACGGTTGGGAAACGAAAAAGGAACCAACGGCAAACCCAAGAAGACAGAGCGCCGTCAATAAATTGACTCTCAAGGTGAAATGAATGCCGGCTCCCGGATTTTTCTTGATCCCGGCTTGGCGGATCTGGTGCAATGCGTAAACGTAAAGGCAAAAAGCCACGGCCAAGAGCATGATGGAAAACCGGCTCCACGGTCCAATCCCCGTCAGGTTGCTTAAAGCCCCGCCGGCCACGGCCAGATTGAAGAGAATGAATGTCCGGTTTTGCCATTGGACCGGATGGTTGTGAGAGATGGAAAACATCGGCAACAGTTTATAACTGAAACCGATAATCAGATTGCCGAACCACCCGGCAACTCCGAGCCAGATATGGGTGTAAAGAAGCTGGTCATGGCTTACACCCAACTTCCCAAGGGTAAAATCAAAACCCATGAACATGCCGCTTGAAGCGGTCAAGATCAGGCAAACCAGCGCACTTCCCGCATGAAGGGTGACCGGATTCCATTGCCCGGCCCGAAGCAGCGTGATGCCGATGTTGAGAACAAAAAGCAATATGGCCAGAAACAGGAGCGTGCCAAAAACGGCAATCCAGCGGGGGTTAAAAAAGAAAAAGCCGAAGATCAAGCCGGTATGGCCGACCAGATAAAATACATAATGGAGTCCCCCCATTCTTTGGCTGAAGAGTGATTGGTTGAGAACCACCGGAACCAGTTGGTACACCGCTCCCATGGCAATCATGGATGCCCACCCCAGGACGAAGAGATGCGTCAATCCCCATCCGTACGGGGAGCGGGGTGCCGTCACATGAATGTCAGACTGGAACAAAACCGCTGTGATGAAAAACGCGGTCATTTCCAAGATCCCCGTAATGATAAAGCACAGCGGGATTTTCAGTGATGACGAATCGGCATGGATCTTCATCCCTTTCACTTCCTGTCCGTCCGGGGCTTGAAAATGGTAACCTCTGCGGAACCATCCTCTTTTTGTTCCACTTCACAGGTGCAGCCGAGCCGGTCCAACTCTTCCAACAGATACACGGGAACCCGGTCGTTGTGAATGATCACTTGATCACCGGGGGCGGCTTTTTCCAATTGATTCAGGGTCCGGACCATCGGTTCCGGAGGTTGAAGTCCCCGGTTGTCCAAGCGGTAAACCTGAGGTTCAATGTTTTCCCCGGTTTTGAACAGAGGTTCCGCCTTTTCCTCGATCGTTTTCCCGTTTTTCTTATAAAAAAGACAAGTCCAGTGATCCGGGGCCAGCTTGGTGGCATCACACCGAAAACCTTTCATTTTTAACATCCGGATTAATGGAACGGGTTTGAAGGTTGTATGCAAAAGCAACTGTTCTTCCTCTTTCAATGAGGCCACAGCGCCCATGATTTTTTGAAAAGGCTCAGTTTTTTTCCTTAAGTCCTCACGGACATCCAATTCCACGACGGGTTGATTCGGTTGTTTCATCAGTGAATCCCTCCTTTGGTTCCCACTATATAGAAGGACGGTCCGGGAGGTTGTGACCAAAATCACATCAAAATCCGGGTTTTGTTTTTCCGGGGAAAAGTGGGAAATATCACAGAAATTCCGGAAGAAGCGGACTAAAATAAATGCGGAATCCGATCAATAAAAGGAGGGTAAAGCATTGGAACATTCCTTTGCGGCAACCGTCGATGCAAGAGAATATGAACCGAAGGACAAGCACCGGATTATTTTTGAAACCTTTGACGGGTTACAGCCCGGACAAGCGATGTTGTTATACAACGACCACGATCCGAAACCACTCTATTATCAGTTTCAAATGGAAAGGCCGGAGACTTTCGGGTGGGAGTATTTGGAGGAAGGCCCGGAGGTCTGGCGTGTGGCGATCAGCAAAAAAGGTTGACATTTCATATTTCCATCCTTCTTGATGATGTGCCAAAGGGGATCACTGCTTGAAGCGTGATCCCCTTTCAAGCGGATGTCACCCCGCTTTTAAAAATAATCACGGCTCCGGTCGCATAAAGAACGAGGATCAGTGCCGTCAACAGCAGTTGAACCGGTACGAGGTTAAATCCGCTGATTTCCACCAAGTAATTAAAAAAATCATACAAGTCGGGCGTGATTTCGATGAAGGCAATGGTATACAAGGCCAGAGCGGCATTTTTGGCAAATTTCTTTTGCGCTTCCTCTTTGCGCTGTTCAGCCCGGCGCATTTCTTCATTGGAGAGAAGTTCAATCCATGAATCGAGTTTTTCGCTCAACGCTTCCAACAATTGATGGATACGGCTTGTCTCGCTTAATCCGTCGACCATGGGTTTAAACCGCTCGGGCAAACGGGATTCGTCCGTATATTGGATGATCAGAGGGATATCATCGATTTCCACTTTTAAATTGATGCATTGATCGATGGACAAATTCAGTTGTTTGAATTCATCTTTTTCAATAGGCAGCCATTGATCCAGGCAATACAAGTAGAACCAGTAATGTTGCAAATTCAATTCAATCAATTCATAAATCTCTTTGTCTGAATCATCAAATTCTCCAAAGATGATGACGGCGGCCCAACTCATAAAAATCATGAGCTGCTTCCGGTCATCGTAGTCCACGGGTGTCGAGTGGATGTTTTCCAGGTATTCCAACACTTTCTCTTTTTTTGCATTCATTTTTGAAAGTTCATTGGAATCATCATAACCGATTAAATCCGGTTTTAAAATGGCGGGAATCCCTTTTTGCAAAAAGCGGGGCAGACGGTCAAAGCGGGAGGTGTCCACATTGCCTTGAAGCGGGCTGAAAAAAACCATGGTCATGACGTAAGAAAGTCCGTTGTATTCCCAACCCGAGGAAGCGCTGGGTCGAAGCTCTTTCTTGTGTTTTCTTACCAACTCCCTCAACTCTTTGGTAATGACATAGATGACGGGAGAAATGTCATGGGTCCAATTGAGAATTTTTTGGTGGGATTTTTTCCTTTTTTCCAGATACCTCGCGGCAAAGGTCATGTTTCCGATGTAAATTTGCGGTTTGGAATAGTATTTAAACAGCCCGATTCCGGTTTGAAAAATATATAAAAATAATTGATCGGTGATTTTGGTGCAGATACATTTTTCATACAAAATCACTTTTTCCCTTTTCGTCAACGGGGATACTTGAAAATTTTTGGACAGATAAGCGCTGATTTCTTCGAATAATTCCGGGGAAAGCCGGGCGCCGAGATCAAACGGGATGTATCGGACCGGTTCTGACAAAATTTGATAGAAAGTCAAGTCCGCTCCCCCTTTCATTTAGTTATAAAAATAAACAAAAATTGCATAATATTACCTTTGTTTATTAATTATAAATAGAATTATATAAAAGATTCAACGGATGATTTTAAAATAAAACAACATGACACAGAAAAATCCCTGCTGCATGTAGAAACAGCAGGGAGACGGGGTGGTTATACATCTTTTGTTTTCTCAGCATAAGGAAGGCGTTTTCCCGTCAGGGACCATCCGGTTTTCAGCCCCAGGACAAACCAGCCGAGGACAAGTGCACCGATGGCAAAGAGGGTGTCTCCGATGGTCCGGAGCCAGACAAAGGTGTGAATGATCCCCTGCGACATGAATTGGGCGGAACGGGCGTACCAGGTTCCGTGTTCGACGCTGGCCCATGTTTGCATCAGGCCGATGGGGAGCAGGCTTAAAAGAACCATGAGCGCCAGTCCAATGTTGATGGTCCAAAACGAGAACGACAACGCTTTGGTCTTCCAAATCGATGGTTGGGTCAAACCGCGCAAACAGAAGAGCATCAATCCGATCCCGAGCATGCCGTACACACCGAACAAAGCGGTATGCCCGTGTACGGAGGTGGTGTTCAAACCTTGCATGTAATAAAGGGCGATCGGGGGATTGATAAAAAACCCGAACAATCCGGCACCGGTCAGGTTCCAGAAAGCAACGGCAATGAAGTAGTATATGGGCCATCGATAGGCTTGCACCCAGGGGCGGACCCGGGTGAGGGTTAAATTTTGATACGCTTCAAACCCGATCAGCACGAGCGGAACGACCTCAAGGGCACTGAAGGTTGCACCCAACGCCAAAACCCCGATCGGCGTTCCGCTGAAATACAGGTGGTGGAATGTGCCGATAATCCTGCCGAACAAGAAAACAATGGTAGAAAATAAAACGGAAGCGGTTGCGGTGGCTGTTCTCAAAAGTCCCATGCGAGTGAACAGGAAGGCGATGACAACGGTCGCAAACACTTCAAAAAATCCTTCCACCCACAGATGGACCACCCACCAGCGCCAGTATTCGGCGATGGCCAAATGCGTGTGTTGACCCCACATCAGGGCGGGGATGTAAAACAAGGGAATGGCGGTGGATGCAATCATGAACAAAATGAGCAAATGTCTGTCGTTTTGTTTCTTTTTCAGTGCCGGCCAAATTGCCCGCAACATCAAAAACAACCAAAGGAAAAGTCCGATGGTCAAAAATAACTGCCAAAACCTTCCCAGATCGGTATACTCATATCCCTGATGACCGAACCAGAAGTTGGACTTCAGCCCGAACCGTTCTTGAACAGCATACCACTGTCCGGCCATTGAGCCGATGACAATGATCACTAAGCAGACAAATAAAATGTTGACTAACAGACGTTGGTGTTTCGGTTCACGGCCGGAGACCGCCGGGGCCACAAACAAGCCGGTGGCGATCCAAGCCGTGGCAATCCAGAAGATGGCCAGTTGGGTATGCCATGTGCGGGTGATGGAATAGGGAATCCATTCGGCAATGGGAATCCCGTAAAAACCGCTTCCTTCAATCTGGTAATGCGCGGTGACGGCTCCCAACGCCACTTGAATCACCCATAATATACCGACCACCCAGAAATATTTCAGCGTTGCTTTCATGGATGGAGTGGGAGACAAGGCAAGCAAAGGATCTTTCTCGGGAAAATGATCTTCCGATCCAATTTCATCGTGTTGTTGCGTGGCATAATACCAAGCCAGAGCGCCTACGCCGGCCAGCAACAGGACGAAGCTGACCACCGACCAGAGTACCATTTCCCCGGTGGGATGATTGCCGATGAGGGGTTCATGCGGCCAGTTATTGGTGTAAGTAATGTCACTTCCCGGACGGTTCGTCGCACATGCCCAGGTTGACCAGAATAAAAAGGCGTTTAAGGATTTCATCCGCCCGGGATTTTTGACGGTCCCTTCGGGAATGGCATATTTTTCGCGCAAATCATCCAGTTTCGGATCGCTGCCAAACAATGATGAATAGTGCCGGCTGATGTGGGAAACCGCCTCGGCGCGCAAAGGGGAAATGACGAGATTGCCGGTTTCCGGTTGATAGGTGTTGGTTCTGATTTCTTTTTTCAATCGGGCTTGAAGCATGGCTTTTTTCTCATCGTCCAGTTGCTGATACGGTTTTTTGAATTGATCCGCGGCATATCGGTTTAACATCCACACGGCTTCCCGGTGCAGCCAATCCGCATTCCAATCCGGGGCCACATAAGCGCCGTGTCCCCAGATGGTTCCCAATTCCTGGCCGCCGATGGATTGCCAAACGTTTTGCCCTTCTTTGATATCTTTTTCGGTGAATAAAACCTTGCCGGTTGTGGTCACGACCTTTTTGGGAACCGGGGGCATGGTTTGATAAATTCTTCCCCCGTAATATCCCAAAATGGCGAATGAAATCAGAATCACCAGTCCAAGACCGGTCCAAAGCCGGGAATATCGCATGTCAAAATCCTCCTGATCCGTTTTTAAGCTGGTACTATTTTTTGGAACCAAGTATTTTTATTCAAAAACAAAAGAAGAAAAAGAACGGAAGGGAAGACAGAATCTGGACATTTTATGAAGAAAATCGGGAAACGCTTGGCAAAAAAATATATTGTGATATCATTAACAATGTGCAGAGGCAAACACTCTTTCGCTATGTTTTCATCAGGAAAATATTGGGATGGATGTTCAGGGAATATATAATCTGTTATTGCCGTTTTACTTATGTGAAAGAGTTGGTTTGGGTAAAAACTTAGTAAAGAGGTTTGCAGTATTGAACCATAAAGAGGTAGATAAATATGGGTCAAATGAATGATGTCTTTTTGAGAGCTTGTCGGAAAGAGGACACCCCTTATGTTCCTGTCTGGTATATGCGCCAAGCAGGAAGGTATCAGCCGGAGTACCGGGAAATCCGCCGGAAATATTCTTTTTTTGAGATGAGCGAAAATCCCGAGGTTTGTGCGAAAGTAACCTCGCTTCCGGTCGAACAGCTGGGCGTGGATGCCGCCATTTTGTTCGCGGATATTATGACGCCGCTGAAACATATCGGGGTGGAAGTTGCCATTCAACCGGGAACCGGTCCCGTGATTTCCAACCCGGTCCGGGGGGAAGAGGAGATCGGCCGTTTGGGAGAGCTGGATCCGGAGAAACATGTGCCTTACATTTTAGAAGCGGTTCAGATTTTAAATGAGCAGCTTTCCGTTCCTTTGATCGGTTTTGCCGGTGCGCCGTTTACGCTTGCCAGTTACATGGTGGAAGGCGGACCTTCCAAAAATTATCACAAAACGAAAGCATTGATGTATTCGGCACCTTCTGCTTGGGATCAGTTGATGGCGAAACTCGGCGACATGACCGTGAATTACCTGAAAGGACAAATTGAAGCAGGTGCCCATGCCGTACAAGTGTTTGACTCCTGGGTCGGTGCCTTGAACCAACAGGACTACCAAACCTATGTGGCTCCGGTCATGGAGCGGATCTTCCGCAAATTAAAGGAAGAAACTGATGTTCCCGTCATCTATTTCGGAGTCGGAGCCGGTCATCTCCTGGAGGCATGGAATCAACTGCCGGTGGATGTGATCGGTCTGGATTGGCGGACGCCGATCCGTTTTGCGAGGGAACAAGGGGTCGAAAAAGCTTTGCAAGGAAATCTTGACCCGTCGATTCTCATGGGCCCTTGGGAATTGATCGAAGAAAGGACCAAAGAAATTTTAGACCAGGGAATGAAACAGCCGGGCTATATTTTCAATTTGGGCCACGGTGTGTTTCCGGATGTTCGCGTGGAAACGCTTCAAAAATTGACTGAATTTGTCCATCAATATACAAGAAGGTGATGACCATCGCTAAAAAAACCGGTTTGCTCGTCATGGCGTACGGAACTCCGAGAAGTCCTGAGGAAATTGAACCTTATTACACCCACATCCGCCACGGCAGGAAGCCGCCTGCCGAACTGCTGGAAGACCTGAAACAACGTTATGAAATGATTGGCGGGGTGTCCCCGCTGGCACGCATTACGGAAGAACAAATGAAAGCTTTGGAAGAGGAGTTAAATTCCAGGTCTACGGACCGGACTTTTCAAGCGTACTTGGGATTAAAACACACGTACCCGTTCATTGAAGAGGCGGTCCGGAAAATGAAAGAGGATCAGATTGAGGAAGCGGTCAGCATTGTGTTGGCGCCACACTATTCCACCCTCAGCATTCAAACTTACAACGACCGCGCAAACCAGGAAGCCGAAAAACTGGGCGGTCCGAAGATTAAAACGGTGGACAGTTGGCATGACCATCCGGGATTTATCCGTTTATGGGCAAAACGGGTTCAAGAAACTTTGTCGTCCATCCCGGAAGAAGAGCGTGACCAAACGGTGGTGATTTTCACGGCGCACAGCCTGCCGGAGAAGATTCTTAAAACGGGGGATCCCTATCCGGAACAATTGGCGGAAACCGCCAAGCTGATTGCCGAAGAAGCGAAAATTCCACACTATGCGGTGGGCTGGCAAAGCGCGGGCCGGACGCCGGAGCCTTGGCTGGGACCGGATGTGCTGGATTTGACCCGGGAATTGTACGAACAAAAAGGATATCGTTCGTTTGTCTATTGCCCGGTCGGCTTTGTGGCGGATCATTTGGAAGTGCTTTATGACAACGATATTGAGTGCAAACAATTGACGGATGAGTTAAACGCCAACTATTACCGTCCGCCGATGCCCAATGTCGATCCGGAATTTATCCGTTGCCTTGCCGATGTGGTGGAGAAGCGCCTGACCACTTGAGGAGGAGATTTGTCATGAACGTGCAAACACCACATGTCGTCATAGCCGGAGCAGGGATGACCGGTTTGTCCGCGGCTTATTATTTACAAAAAACATTAAAAGAGAAGGGGCAAAAGTGGCAAATCACTTTGTTGGAAGCTTCCGGCCGTCTGGGCGGAAAAGTGAAAACGTTGGTCCGGGACGGCTTTGTCATGGAGCAGGGGCCCGACTCCTTTTTGGAGCGAAAGAAAAGCGCGTTCGAATTGGCCAAAGATTTGGGGCTCGAAGATCAATTGGTCCGGAATCAAACCGGACAAGCATATATCTTGCACAAGGATGAGTTGTTGCCGATTCCGGAAGGGGCAGTGATGGGAGTCCCGACCCGGATCATGCCGTTTGCCCTGACTCCTTTGGTGTCCCCGGCCGGCAAAGTCCGTGCCGCGGCCGACCTGGTTCTTCCCCGTTCCAATGTGGCGGAAGGAGCTGACCGGTCCGTGGGGTCGTTTTTCCGCCACCGCCTGGGAAGCGAAGTGGTGGACCGGATCATCGAACCTCTGCTATCGGGGATTTATGCAGGCGACATTGACCAGATGAGCTTGATGTCCACCTTCCCGCAATATGCTGAGATGGAGAAAAAATACCGCAGCATGATCCTGGGAATGAAATCGATGCGTCCGCCGAAAGCCAAATCCGACAAACCCAAAGGCGCGTTTCTCACGTTGAAAAACGGGCTTCAATCCATGGTCGAAGAATTGGAAGCACAATTGCAGGAAGTCCGGGTGATCAAAGGAGAACAATTGAAGCGGGTGGAAAAACAGGAGCAAAAGTACCGGTTGTATTTGGAGCAAGGAGAGCCCTTGGAAGCGGATGCGGTGATTTTGGCATTGCCGCATGAAGTGAACCGGGAAATGTTGGGGCAAGCCGACTTTTTGAAGCCGCTGAATGACCGGCCCACTTCAGTGGCGACGGTGCTGCTGGCTTTTTCGGAGGAAGAAGCCCCCAAAACCAAAGAAGGAACCGGCTTTTTGATTCCGCGTGTCGAGCCTTACACCATCACCGCCTGCACATGGACGCACAAAAAATGGCCGCACACCACCCCGCCGGGCAAAGCGTTGCTTCGTTGTTATGTGGGGCGTGCCGGCGATGAAGAGATCGTGTACAAGTCGGATGAGGAAATCGTTGAGGTGGTTCTCTCCGATCTGAAACGGATCACCGCCTTTCACGGAGAGCCCGAGTTCACTCATGTGACACGTTGGAAGAAGGCCATGCCGCAATTTGTCGTCGGTTTTCCGGATTGGTTGAAGCAATTAAAGGAACAGATGTCCGTCCATTATCCGGGAGTTTTCATGGCAGGATCCAGTTATGGTGCATCCGGGATTCCCGATTGCATCAGCCACGGAAAAAAAGCCGTTCAAGATGTGATAGAATATTTATCAGCTTGATCAGATGAGGAGTGAAAAAAGATGGCGCAAGCAGTGGAAACGTATGAAGGCTGGTATGCTTTTCATGATTTTCGCTCAATCGACTGGAATCAATGGAGATCACTCAGTGCCGGCGAAAGAGAAGAAGCCGTGCGCGAGCTGAAGGAAATCCTGAAGCAATATGAATCCGTTGAAGAAAATAAACAAGGAAGCTTTGCCTTATTTGCCATTTCCGGTACCAAAGCGGATTTGTTGTTTGTTCATTTCCGTCCCACGTTGGAAGAGTTGACCGAAGTCAAAAATCAATTTAACAAAACGCGTTTTGCGGGAGTCACCACCACCCCTTATTCCTATATTTCCGTTGTGGAACTTGGCGCTTACACCATTCCGCCCCATGTCGATCCGGAAACGGATCCGTCGGTGCAAAGACGCTTAAAACCGATGATGCCCAAATCCGGTTATGTCTGCTTCTATCCGATGAACAAAAAAAGAGACGGGGAAGACAACTGGTACATGCTCTCCATGGAAGACCGGAGAGAAATGATGAAAAGCCACGGCATGATCGGCCGTTCTTATGCCGGGAAAGTGCGGCAAATCATTACCGGTTCGGTCGGTTTCGATGATTGGGAATGGGGCGTCACCTTATTCGGCGAAGACCCGTTGCAATTCAAAAAGCTTGTCTACGAAATGCGTTTTGACGAAGCGAGCGCACGTTTCGGTGAATTTGGCCCGTTTTACGTGGGAAATTACTTGACACAGGAAGATTTGAACGCTTTGCTGTTGGAAGGTTTAAACGGATAAACCCGACCCGCTTGATCTTGTTATAAGGATGATGGACAAAGAGGTCCGTCATCCTTTTTTTGATGATGAAATGACTTCGGCCGCAATGGGTGGTTATGATAGAATAAAAAAGAATTTCTCTAAAAGGTGGTACGGATGAAGAAAGCAGACAAGTCCGCCTCCTGAATTTACACCCGATAAAAATTCAGGAGGCGGATTCAATGAACTTTCATAAAGTGAATTGGAATGAATGGGAAAGAAAAGAAACATTTCATCATTTTTTGAACCAACAAACGACATTCAGCATGACCACGGAAATAGATATCACTGCTTTGTATGCAAGGATCAAACAAAAGGGATTTAAATTTTACCCTGCGTTTCTTTACGTGGTGACCCGCGTGGTCAATTCACATCCTGCTTTCCGAATGGGTTACAACCATAAGCGGGAATTTGGCTGTTGGGATCAATTACATCCGCTGTACACGATTTTCGACCGGGAGTCGGAAATGTTTTCGGGCATTTGGACAATGGCAGAAGGTGATTTCAAAGCATTTTACCGTTTATACCTGACTGATGTGGAGCGATATGGCGGTTCGGGAAAATTGTTTCCCAAAACACCGATCCCGGAAAACGCTTTTTCTGTTTCGATGATTCCATGGACTTCCTTTACAGGGTTTAACTTGAATATCCATAATCAGCGGGATTATCTTCTGCCGATTGTCACGGCGGGAAAATGGATTCGTCACGGCCGTTCCATCCGCTTGCCGGTGGCATTGCAAGTTCATCATGTGGTATGTGATGGATATCATGCGGGGATGTTTATGAACGCCGTTCAGGAATGGGCGGATCACCCGGAGGAATGGCTTTGATCTTTTCCAAAAAACCGGCTGTACTTTTCACAGCCGGTTTTTTTCTCCTGAATCCCGGGCACACAGAAGAGTTTTACAGAGGGGGATTTTCCACGCCCAAACGGATCCAATCTTCTTTGGGCGGACCATAAACGCCAAAAGGTTTTAATCCGGCTTGCCGGATCAGAACCGTCAATTGGCCGCGATGATGGATTGTGTGTTTGATGAGTCCCATTAAAATTTGGGCATTGGTCTCTTCTCTTCCGAAAGCGATTTGCACTTCTTTTAATGTCTCGTCATTCCACTGCTGCAAAATGGCTTTGGCGGCATTGGCGCTTACGTTTTTAAAGGTTTCGGCGATTTCTTTGGCCGATGGCGGAACTTGATCGGCATTTTCCACTGGATCGATTTTCAAGCCGAATTCAGACAAATATTCCGGGATGCAGGTGGTTAAATGCCACGCGATTCTCCCCAGTGTGCGGCCTCCGGGATAAACTTGTTGTTTCAAGGAGGCATCGGTCAGCCCGTCCAACACGTTTTGTGTCAAGACGGCTTCTCTGTTCCATTCTTTGATGAAGTCTGAAACGGTTACGTACATCCAATCATTCCTCCATTGTTGATTGACTGCTGTTATTATTTATCTGCCAACCATGATATATTATGATGGTGACAACCCTTGTCACCGTTCCACAGAATTTGGAGGTTTTCATGTCCAAGGCGAGAAGATTGAATGAAATGATCATGATGGTGAACCGGAAAAAAAGATTTACCGTCCGGGAACTGGCACAGGAATTCGGGGTTTCCAAACGGACCATTTTGCGGGATTTGCAAGAGTTGAGTGCGATGGGGGTGCCGTTGTACTCCGAGACCGGGCCGAACGGAGGGTATCGGGTCTTAAAGGAGAGAGTGCTTCCTCCCATTGCTTTTACCGGGGAAGAAGTGATGGCCGTCTTTTTTGCCATTCACGCATTGAGGCATTATCTCTCACTTCCTTTTGATGTGGAGTATGAATCCGTCATCAAGAAGTTTTATTTAAATCTCTCCGGAGACCTGCGGGACACCATTGACAATCTGAAAGACCGGATGGACTTTGTCACTGTCCATCAACAAGAAGAGATCCCTTTTTTGAAACCACTGCTGAAAGCCGCGGTTCAACAGGAAGTGTTAAACATCCGGTATCAAACGGGTGAAAAAACCAGCTGTCGGAAAATTCAACCGATCGGGGTTTATGCAAGAGACGGAAAATGGTATTGCCCGGCTTATTGCTTTTTGAGAAAAGATTACCGGGTTTTCCGGTGTGACCGGATTCAATCGGTGGAACATGACGAAAACACAAAGCCGGTCGATTTATCGGGGATCCGTTTGAAAAATCGTTTTTCGCTGATGAACCGGAAGCGGGAAATGATGGAAATGGTTGTGGAGCTCACCCAAGACGGAACAGAAAAATTTCAACCGGTTCAGTGGCCGGATCTGTCGTTGGAAAAACGGGAAGATGGCTCAGGTTTGATCACCGGGAGAATCGCCAAAGACGATCTCCCTTTTTTCGCAGACTATTTTCTTAAATACGGCGAGCATGCCCTGGTCAAGAAGCCGCTTGCCTTAAAAAAGCTTCTGAAAGAAAGATTGGCAAAAACGCTTGAACAATACCGCCCGCTTTAGGTTCTTGCGGGCGTTGACGCCTGAGAGGACGGATGCAGCGAAGGGCACGTTGTGTGGAAAGAAGGGGTTGCTTGCTCTCACATTGACTCCTTAAAATAAGCGGATGTATAATAAAATTCAATGTGATTCTTATCGGATGGAGGGTACACAGGTGTTGACGACAATTAAAGAAATAGCCGGCCATGTGGGAAAAGAAGTCAAATTGGGCGGATGGCTTTACAATAAACGTTCGAGCGGAAAAATCCAATTTTTGCAGCTTCGGGACGGAACCGGATTCATTCAGGGGGTTCTGGTCAAAAGTCAAGTGGCTCCGGAAGTGTGGGACGCTGCCAAACAATTGACACAAGAAAGCTCATTGTACATATGGGGAACCGTACGGGAAGATGAACGGGCACCCGGCGGTTTTGAGTTGGATGTGACCAACCTCGAAGTGATTCAGGTGGCGGAAGAATACCCGATTGCGAAAAAGGAACACGGGGTGGATTTTTTGTTGGACCACCGCCATTTATGGATCCGTTCCCCCCGGCAACGTGCCATTTTGACCATTCGGGCTGAAATTATCAAAGCCTTGCAACAATACTTGAATGATGAAGGCTTTACATTGGTGGATCCACCGGTTTTGACGCCGTCTTCCTGCGAAGGAACCACCAACCTGTTTCACACCAAGTATTTCGATGAAGACGCCTACTTGACGCAAAGCGGCCAGTTGTACATGGAAGCCGCCGCGATGACGTTGCGAAAAGTTTATTCATTCGGCCCGACGTTCCGTGCGGAAAAATCCAAAACCAGACGCCATCTGATTGAATTTTGGATGATCGAGCCGGAAATGGCCTTTGTGGAACATGAAGAAAGCCTGAAAATCCAAGAGAATATGGTTGCCCATGTTGTCCGGCATGTGTTGGACAACTGCGAAGCGGAATTAAAAACATTGGGCCGGGATATCAGCCGCCTGGAAAAAGTGACGGCTCCGTTCCCGCGCATCACTTATGATGAAGCCGTGGAACTTCTTCACAAAGAAGGATTTTCCGATTTTGAATGGGGGGAAGACTTCGGGGCGCCCCATGAAACCAAAATCGCCGAAAGCTTTGAGAAACCGGTCTTTATCACACATTATCCGACAGAGATCAAGGCTTTCTATATGAAGCCCGATCCGAACCGGCCGGAAGTGGTTTTGTGTGCGGATTTGATTGCTCCCGAAGGCTATGGGGAAATCATTGGCGGAAGCCAGCGGATTGATGATCCGGAACTGATGCGCCAACGCTACGAAGAACATAATTTGCCGAAAGAAGCTTATCAGTGGTATATGGATTTGAGAAAATACGGTAGCGTCCCCCATTCCGGATTCGGCTTGGGATTGGAAAGAACGGTGGCCTGGATTTGCGGATTGGATCATGTCCGGGAAACCATCCCGTTCCCGCGGATGCTCAATCGGTTATATCCATAGAAAAGGATAAAGCATCGGTTTTTTCTGACCGGTGCTTTTATCCATTTTTCAAGTCTGTTTCACCAAAGTGGATCATGGTATAGTGGAGGATAAGAGAGGTGTGAAAGAGCATGGACCGGAAGGAAACGGTGCAAGCGGCAATGGTTCATCTCCTGAAACAAGGATTCGTGTCTTTGCCCGTTCTTTTGTTTACGGAGTATAAACGGCTCGGATTGACGGAAACCGAAGCCATGTTGCTGATTCATCTTTATATTTTTCAAGAAAAAGAAAACAAGACGTTCCCGACCGTGGCGGAATTGGAAGAGCGCATGAGCCTCAGCCAACATCAAATCGTGGACTTGATTCAGCGCCTGTTACATGGCGGATTTATAAGGATTGAAGAAGGTCACGAACACGGGATCCGGAGTGAAAGTTACGAGTTGACCCCGCTTTTTCAACAATTGGTCTCATCGTATCTGGATCATAAACAGAAGAAACCGGAATCGGACGGTGAAACATATCAGCAAATATTCCGCGTGTTTGAACTGGAATTCGGACGGGCCCTGTCTCCGATGGAATGCCAGATGTTGTCCCAATGGATTGATGAGGACGGCTATTCAAAGGAGATCATCGAAGCCGCTCTCCGGGAAGCCGTTTTTTGCGGGAAAGTGAATTTTCGTTATATTGACCGGATTTTGCTGGAATGGCATAAAAACAATGTGCGCACGGCGGATGAGGCGGCCGAGTATTCTCGCAAATTCAGGCAAAAAGGAAGCTTATACCAGACCAATGTGAAGGAAAAACCGGTTCTGAGCGAAAATGGTTTTTCTTTTTACAACTGGGTGAATCAAGAGTAGCGGAAGAAACCTTCTTCCGTTCTTTTTTTGCCCGGATGGCGAAATGGAATTTTATATGTAATTAATTTATATTGAAATAATATTTCACATCAGATATAATGAAAGCGCATTCAAGGAAGTCTTGATTTGGAGGGTATAACATGGAAGAAGAGCTGAGTCGTCTGACGACCGAGTCCCGAAATGAACGAAGCAAACAGATTGACAAAATGGATACGATAGAGATTTTAAGACTGATCAATGCTGAAGATCAAAAAGTTGCTCAAGCCGTCAAGGAGGTGATTCCGGACATTCAGGTGGCGGTGGAGTATGTTTGCAATTCTTTTTCCCGCGGAGGGCGTCTGATTTACATGGGAGCCGGAACCAGCGGACGGTTGGGGGTTCTCGATGCCGTTGAGTGCCCGCCCACCTTCAGCACGTCTCCGGAACAGGTTGTCGGTTTGATGGCGGGGGGCGAAAAAGCCTTTGTGAAAGCCGTTGAAGGGGCGGAAGATAACGAAAAACTGGGGGAAGAAGATTTAAAGGCGATTGGCTTGACGGAAAAGGACACAGTCATCGGCATCGCCGCCAGCGGAAGAACCCCGTATGTGATTGGAGCGCTCCGGTATGCGCGAAGCATCGGAGCCCACACGGTGGCCCTTTCCAGCAATTCCGGTGCACGTATCAGCCAGGTAGCTGGTCATGCCATTGAAGTCATTGTGGGTCCCGAGGTGTTGACGGGTTCGACCCGGATGAAAGCAGGAACGGCCCACAAAATGATTTTAAACATGATTTCCACCACTGCGATGATACGCATGGGAAAAGTGTACGAAAATCTGATGGTGGATGTCAAAATCAGCAATTACAAATTGCAGAAACGGGCCGTCAACATCATCCGCACCATTACCGGCTTGTCTGACCGGGAAGCGGAAGACTTGCTCCGGCGTGCCCAAAATGAAGTGAAAACGGCCATTGTGATGGATTTGTCTGAAACCGGCTACGAAGAAGCCAAAAGATTGCTTGAAAAGTCCAGAGGGCATGTCCGCAAAGCGATTGCGCTCGGGATTCGGTCCCGTTAACAGGAGGGGAGGAGGGTGTCTGCAGGAGGACTTCATCGTTTGCGTTTGATGTTGGATCAATTGACGGTTGCCGAGCGGAAAATCGCCGAATATATTTTGGCTCATCCACAGGAAGCGGTTCACGAGACAGCGGCCGGATTGGGGAAACGGACGGGCACAAGCGGAGCGACGGTGGTCCGTTTATGCAAGTCCCTGCAATTAAACGGATTTCAAGATTTAAAAATGCGAATCGCCGGCGATCTGGCCAAGCCCGAAGAGAGCGGCTACCGGGATATCAGGCCCGAAGAGCCGGCCGGGTTGATTTTGCAAAAGATGACCAGTAACAGTGTGCAAAGTTTGCGCGACACGGCCGAGGTTCTTGACTTGAAAGAGTTGGAAAGAGCGGTGGATGCTTTATTAAAGGCAAACCAAGTCCACTTTTTCGGAGTGGGGGCTTCCCACATCATCGCTTTGGATGCCAGGCAAAAGTGGCTTCGAATCAACAAAGGGGTGACTGCTTTCTCCGACTTGCATCTGGTTGCCACCCAAATTGCCAATGCCCGGCCCGAGGATGTGGTGTTTGCCATTTCATATTCGGGGGAGACCCCGGAAGTTGTGAGGATTTTGCGCTTGGCCAAAGAGTATGGAGTCAAAACCATCAGCTTGACCAAATATGGTTCGTCCACTGTGGCCGATCTTTCAGAAATCCGCTTGCATATCGCTCCTTCCGGTGAAGCCGTTTTTCGCAGCGGGGCGACTTCCTCCCGGATTGCGCAACTTCATGTGATTGATATTTTGTTTTTGGCGATGGCCGGAAAAGAGTATGATCAAACGGTTCGTTTGTTGGATAAAACCCGGAAAGCCACCCGGCGGCTGAAGGAAATTTAACCCAGGAAGGAGAATTGGGATGGGAAAAGACAACAGCGTGCTTGCGCAAGAGATTTTGCAGCATTTGGGGGGAGCCTCCAATATCGCTTCTTACACCCACTGCATGACGCGGCTTCGCGTCAAACCCAAAAATGAAAGCCTTGTCAATAAAGAGGCATTGAAGGAAATCGAAGGCGTCATCGGTGTGGTGGAGGAAGAAACGCTGCAAATTATATTGGGGCCCGGCAAAGTGGCCAAGGTATACGAGGAATTCAAAATATTAATGAAAAACACGGGCGAATGGGATTTAAACGAACAAGCGAAGCAAAAAAGGGAAGAAATCAAGGAGAAAAACAAAACGCCGTTTAAGCTGTTTTTAAGAAGAATCGCCAACATTTTCATCCCCTTGATTCCGGCTCTGGTGGCTTCAGGGCTCATTACGGGGGTTACGAAAAGTGTGGTGCAGGCCGGTTGGATTGCGGAAACTTCCATGATCGCAACCGTCTTGACCGTGATGGGATCCGGCCTGTTTTCATTCCTGGGGATTTTGGTCGGGTTAAATGCCGCCAAAGAGTTCGGAGGTTCCCCGGCTTTGGGCGCGTTAGCCGGCATTTTGGTGATCAACCCCGAAATTGCAAACATCAGTTTGTTCGGAGAAGAATTGGTGCCGGGGCGCGGCGGTCTGATCGGTGTCATGTTTGCGGCCATTTTTATCGCTTTTTTGGAACAACGGATCCGCCGCCTCGTCCCGCAAGCGCTGGATATCATTGTTACACCGACACTGGCCTTGCTGATTACCGGAATCATCACCTATGTGGTCTTTATGCCGATTGGCGGTATGATTTCCGACGGAATTACCAAAGGCTTGCTGGCCATCTTGGATACCGGCGGAGTGATTGCCGGATTTGTGTTGGGAGCCACCTTCCTCCCGTTGGTGGTTACCGGTTTACACCAAGGCTTGACACCGGTTCATCTGGAACTGATCCAATCCATCGGCAACGACCCGTTGCTCCCCATTCTGGCCATGGGAGGCGCGGGGCAAGTCGGGGCGGCCTTTGCCATCTATGTGAAAACAAAGAAGAAAAGGTTGAAAAGGGCAATTGGCGGGGCTCTTCCGGCCGGCTTGCTCGGCATCGGGGAACCGCTTATTTTCGGGGTGACCTTGCCTTTGGGAAGGCCCTTTTTGACAGCATGTCTGGGAGCAGGCGTGGGCGGAGCTTTTCAAGCCGTGTTTAAAGTGGCCACCATTGCCATCGGGGTTTCCGGCCTGCCGCTCACCTTTTTGGTGCTGCCCGGCCAAATCCTTTTGTATATAACCGGATTGGTATTATCTTATGCGGCAGGGTTTTTGTTCACCTATTGGTTTGGTTTCCGCGATGAAATGGCCGGCGGATTTGAATGATCCCGTCAAAAATACAGCTCCGAAGCGGGCTGTATTTTTTCTTGCCATCGGTTGCTGTATAGATTGCATCTTCCTTTCGGATACTAAACAGGACAATCAAGAATTTGCGGAGGAAGATTTCAAATGAGCTGGTATTTGGTTGTGGTCAAAACTTTTTTTACGGTTGTGGTCCTTTTGGTGCTTACGAGATTGATCGGAAAAAGACAAGTTTCCCAACTTTCCCTGTTTGAATACATCACGGGTATCACCTTGGGAAGCATCGCGGCATATATTCCGTTGGAATCGGAACCGAACTCATGGTATTTGGGGGCGCTTTCCCTGTTGGTGTGGTTGATCATGGCACTCGGCATCGAATATTTGGGGATCAAAAGCCAAAAAATAAGAAAGTGGATTGTCGGGACGGAAACGGTGTTGATCAAACAGGGAAAGGTGTTGGAGGAAAATTTAAAGAAAGAGCGGATGACGGCGATTGAATTGATGAGCAAATTGCGGGCGAAAAATATTTTCAACTTGTCGGAAGTGGAGTTTGCCATCATTGAGCCGGACGGATCGGTCAATGTGATGCTGAAAAAAGAGCATCAACCGCTTACGGCCAACATGCTTCATCTTCCTGTGGATCATGAACGGGAACCCGAAATGGTCGTTATCGACGGCCGGATTGAAAAAGAAGTGTTGTCGAGCATCGGGAAAAACGAAAAATGGTTATTGAAAGAACTGGAAAAACAAGGGTTGAAACTGGAAGATGTGTTTTTGGCCCAGGTGGATTCGTCCGGGAAATTGTGGACCGACCTTTATCAAGACCGGTCTTCTTCCCGTCCCCGGCCCCACTGGCGGGCAAACCTCATACAGGCATTGGAAAAATGTGAAGCCGAGTTAAAAAAGTCGGGACAATTGGCCGGGGATCCGGATGAAAAACAAATGTACCTGAACACCGCCCGCAATCTCAAGGAGATCACCAAAGACATCCAGTCCCGTTTCTTATCGCCATCATGAGAATCATTGCATTTTCTGTTGGATTTCTATTATTATTTTTACAGGCCCATTTATAAAAAAAGAGGTGCAAACCATTGGAACAATTTACATTTTATAACCCAACCCAAGTGTTCTTTGGCAAAGGCACTTTGTCAGCGATCAAAGAACAAATCCCCCGTTACGGCAAAAAAGTATTGTTGGTTTACGGGGGCGGAAGCATTAAAAGAAACGGGATCTATGATCAGATCATGAAATATTTGCAAGAGATCGGGGCAGAAGTCCTGGAATTGTCCGGGGTAGAACCCAATCCGCGCCTGTCCACGGTACATAAAGGCGTGGAAATGTGCAAGAAAGAAAAGGTGGACTTTTTATTGGCGGCCGGTGGCGGCAGTGTGATTGACGCCACCAAAGCCATTTCTGCCGGAGCCAAATTTGACGGGGATGTGTGGGACATCATCACCAAAAAAGCCGTTCCCAAAGATGCCCTGCCGTTTGGAACGGTTTTGACCTTGTCGGCGACCGGTTCGGAAATGAACAGCGGTTCGGTGATCACCAATTGGGAAACCCAGGAAAAGCTCGGCTGGGGACATCCGTTGGTCTTTCCGAAGTTTTCGGTTCTCGATCCGGTTCATACCTTTACGGTTCCCAAAGATCAAACCGTTTACGGAATTGTGGACATGATGTCCCATGCGCTGGAACATTATTTCAGTCCTGCCCCCAACGTTCCTTACAACGAACGAATGGTGGAAGGATTGCTGTCAACGGTTATCGAGACGGCACCCAAGCTGCTTGAGGACCTGGAAAATTATGAAGCCAGAGAGACGATCATGTTTTGTGGAATGATGGCATTAAACGGAGTCGTAAACCAGGGGATGCGAGGCGATTGGGGAACGCATGGAATCGAGCATGCTGTTTCCGCCGTTTATGATATCCCGCATGGCGGAGGTTTGGCAATTTTATTCCCCAACTGGATCCGGTATAATCTGGATGTGACCGAAGCCAAAATGAAGCAATTGGCGATTCGTGTGTTCGGTGTGGATCCCGAAGGAAAATCGGACCGTGAAATCGCTGAAGAAGGAATTGACCGGCTGCGGGAATTCTGGAGTTCCATCGGAGCCCCCAGCCGTCTGGCTGATTACGGAATCGATGATTCCAAACTGGAACTGATGGCAGAAAAAGCGGTGGCTCACGGTTCGTTGGGGCGGATGAAAAAATTAACCAAAGAAGATGTTTATGAGATTTATAAAATGTCATTATAAGGGCAAAGGCCGGACAAAACCGGCCTTTGTCAAAGCATGTTTTGTAAGCGCTTTAGCACAGGGAGTGTGATTTTAAGTTTTGCTAAAAAATGGTTTGTTGACAGCTGGGAATAAAAGTTTTTATAATGCATTGTACTGTTAAATTATAATCTATAAATAATAAATATACTGAAAATGCACAGAAGAAAAAAAGTTTGCCTGTGCATTTCAAAATACGGCAATGGTTGCAACAGAACAAAAAGGAGACATCTTCAATGCTTAAGAACACCAAATTACTCACAATCGGTTTGATGTTGTTTTCATTATTTTTTGGCGCCGGAAATTTAATTTTTCCGCCGTTGTTGGGACAAGGGGCTGGAGACCAGTTCTTCACGGCGATGCTTGGTTTTGCGGTCACAGGAATCGGCCTTCCATTGTTGGCCGTGATCGCAGTCGGTTTGTCTGACGGCGGTCTGCAAGCGATCGGAAGCCGCGTCCATCCGGTTTTTGGCCTGATTTTAAGTTTGGTTGTATATTTGTCCATTGGTCCTTTCTTTGGAATTCCCCGGACCGGAACGGTTTCTTTTGAAATGGGCTTGTCTTTCCTGCCGGATGCCATCCGGGAAGCGGTATGGGCTTTGCCTGTTTTTACAATTATTTATTTTCTGATTACATTGTTGATCAGTTTGAACCCTTCCAAAGTCATTGATCGTGTCGGAAAGATCCTGACCCCGGTCTTGTTGACATTGATTGTCGTGCTCTTTCTCCAAAGCTTGTTTCACCCGGTTGGAGATTTGGGAACCCCGACGGATGTTTATGCGCAATTTCCTTTTTTCCAGGGGTTTCTTGACGGCTATTTAACCTTGGATGCGCTGAGTGCATTGGTGTTTGGGGGAATGGTGGTGGCAGCCATTCGCGATGACGGAATCCGTGACCGGAAGAAGGTGGCCGCCACGACATTGAAAGCCGGTTTGATAGCGGTTACTTGTCTGGCTTTTGTGTATTTTTCACTGGCTTATATGGGAGCCACCAGCCAGCAGTTGGGAATGATGGATAACGGTGGTGAAATTTTAACGGCGATTGCTTTTGAACTTTTTGGCCCATACGGTTCCGTCCTCCTGGGGGTCGTTGTTTTGCTCGCTTGTTTGACGACTTCGGTCGGGCTTTCGACATCCTGTGCGCAATTTTTAAACCGGGTTTTCCCGAAACTGTCCTATAAAAAATGGTTGTTGGTTGTAACCGTATTCAGTGCCTTTGTTGCCAATGTCGGATTAACCCAGTTGATTGCGATTTCCGTGCCGGTTCTCAGCATGATTTATCCGGCGGTGATTGTCTTGATCTTGTTGTCTTTCCTGCACTCATTCTTTCATGGTTATCGTGCGGTGTATGCCGGAGGAATTGCAGGCGCCTTTATCATCAGTGTTTTGGATACGCTTACCGGTACATTCGGATTCGATTTTTCATGGCTTGTGGTTTTGCCGCTTTATGACCAGGGAATCGGCTGGCTGGTGCCGGCTGTGATCGGAAGCTTGCTGGGATATGTCATTGGAAAAGCATCCGAAAAAAAAGCTGCCCTTACGTAAGGAAAGGGCGGCTTTTTGCCGTTACAGCCTTTTTAATTGCTTGACGAGCTTTTCGAAATGGAGGGAACGGGAGGCTTTAAAATAGATGACTGCATTTTTCGGGGTTTTTGCCAAGTGGTGCAATGCTTGCCGGAAATCTTTACAAATCAGTACCCTTTTGCGGCTCATTCCATTGGCGATGGCAGATTGGGCAATGATTGCCCCGCGTTTGCCCAAGGTGATGAGTTGCGTCAACCCCAAGCGCGCCGCATATTTCCCTGATTGCATGTGAGCTTGATGCGAATATTCCCCGAGCTCCAGCATGTCGCCTAATACGGCAATCTTGGGGCGGGATCCGCTGACATTTTTCAGGACGTCCAATCCGGCTTTCAGTGCTGTGGGATTGGCGTTCCAAGCATCATTGATCAGGATGCGCCCGGATTTTGTGCGAATGAATTCCAAGCGCATTTTCGGCGCTTTAAAGGTGGCGAGCCCTTTTTGAATTTCCGGAATCGTGGCTCCATAAGCCCAGGCAACCCCGATGGCGGTCAGGGCGTTATAAACATTGTGTGTGCCGAAGACAGGGATCCGGACGGGGAACTGGCGTCCCTGAAGCACGGCGTCAAAGACCATCCCGTTTCGGGTGTAACGGATGTTTTTCCCCCGGATGTGGGCCGGATTGCGGATGCCGAAGGTAAGGATTTTTCCCTGGAATCGGTTGGTTTTCAGTTTTCTTGACCGCGGGTCGTCCGCATTTAAGATCAAGGTTCCGTTTTTGCGGACACCATCCACCATTTCCTGTTTGGCTTTTACGATGAGATGAAGTCCGCCCAAGCTGCCCGCATGCGCTTCTCCGACATTGGTGACGGCACCGATCTCGGGCCGGACGATTTGGCATTGTTTGCGGATGTTATTGAGCGATTTCATTCCCATTTCCAAGAGGAGCAATTTTTCTTTGCCGGTTAACCGGGTCAGATATGTTGGCAAAAAAGAAAAAGTGTTCAGGTTGCGATGGGTTTTCACCATCGGTGCACGATATTTCAAAATGGAAGCCACCATTTCGGTCGTGGTCGTTTTGCCCGCACTTCCCGTGATGCCGATCACTCTGGCCGGGCATTGTTTCCAGTTCCAAAGACCGGCTTTAAAAAAAGCCTGATAAGCATCTTTCACCCGGATGATCGAAGTGCCCGGCGGGATTCCCCGGGCAAAAAGGTGAGCGGGGAGCACGACGGCGAGCGGGCGGATCCGTTGAATGGCCGGCAGTTGCTTTTCGTGCCACCGAATTTTGCGGGTGTAAAAGTAGACTTGGTGCGGTTTCAATTGTTTTGGATTTCCGAAATTGAGGGACTGGACGATTTTGGACGGGGAACCTGCAGTCAATCTTCCGCCCATGATTTGGGCCAGTTTGTTTAATTGGATTGCTTTCATGTATATATCCTCCCATGGCGAGACGATCTCATGGATCCATGTGTGATTATCCTATTCAGGAGGCAATAAACCGGCTTGGATGAAAGCCCATAAAAAGAAACCGGTCATTTTTGCCGCATATTTTGAGGACAAAAAAAGCAGTGCCTTTTTCACGGCACTGCTTTTTGAAGATAAGTTATTCATTGGTGGAGACGACAGGCTTTTTGCTCATCTTCACCCGGGTGTCGCCCATGAACAGAATGGCTATGGCTGCCAGCACGATTGGGATCAAAGCCAGGGCAAAGATATGGGTGATCGATGAAGACATGGCGTCCACGATTTTATCCAGAATGAAATCGGGAATCGCGGCGCGGGCACCAGGCTCAAAGAGCCCTTGCGGGTCGTTGAGGTCAAAGGATGGCCCGTGTCCTCCCTGCATCCCGCCAAAATCCAATTGATCGGTAAAAAAGCGGTTTTGCAGGGAGCCGAATAAGGTGATCCCGATGGTCATGCCAAGAGAGCGGAAGAATGAATTGGTTGAATTTGCCGAACCGCGGTGTTTGGCATCCATGTTGTGCACTGATGCAATGGGCAACAACGAAAAAGAAAATCCGACCCCAAATCCGGCGATGACCATGTAGATGGTGACCAGCCAACGTGCCGTTTCGGGCGTCATGGTACTGAGCAAGGTCATTCCGGCGGCATAAGCAATGATCGAGAGGATCATCAAATTACGATAACTGGTCTTGGTTTGGAAGATGCCGCCGATCGCGCTTCCGACAACCGACCCGAGCATCATCGGGGTCAGGATCAGTCCCGCATTGGTGGCGGACCCGCCGTACACCGCTTGCACAAAGATCGGGATATATACGGTCAACACGACAAAAGTGGCGCCATATAAGAAAACGGCGATTTGGCTGGCGGCAAATAACCGGCGCTTAAACATCCAGAAGGAAATAATCGGTTCTTTTGCTTTGGTTTCAACGTACAAGAACGCAAGAAAAAAGACCGCAAAGATGACAAATAAACTGATGATGGGAAGTGAATTCCAAGCATACTCTTTGCCTCCCAGTTCGAGGGCAAACATGAGGCTGACGACGGCAGTCACCAGGGTGACGGCTCCCCACCAGTCGATTGCTTGTTTTTCATGCTTATTCGACTCTTTGTAAGCCGTGAGAATCAGAACAAGGGAAATGATCCCGATGGGTAAATTGACATAAAAAATCCAATGCCAGCTGATGTAATCGGTAATATAAGCACCGAGCAGGGGACCGGCGACACTGGATGAACCAAAAACCGCCCCGAGCAATCCGGTCATTTTCCCTCTTTGTTCCGGTGGAAAGATGTCAAAAACAATGGTAAAGGCAATGGGCATCAAGGCTCCGCCGCCGATTCCCTGGATGGCCCGGTAAATGCTTAACTGGACAATGCTTTGTGCGGTTCCGCAAAGTGCCGAACCGATCAAGAAGATGGTAAGACCGAAGATAAAGAAACGTTTTCGCCCGTACATGTCCGACAGTTTTCCGAAGATGGGCATTCCGGCCATATATGTGACCATGTAGGCGGAGGTGACCCAAACGAATTGGTCCAATCCCCCCAATTCCCCGACGATCGTCCCCATGGCTGTCGCAACAATGGTATTGTCCATGGCCGACATGAAGATCGCCAGCAATAAAGATGCGACGACAAGTTGAAAGTTGCTTTTTTTTGAGTCCATGATGTTCCTCCTTTGTTGTGGATCACAACAACATTATAATCCCAACCGGTTATTTAATATAAGTAATATTTAACATACGAAATAATGATGAAAATTTCTGTCAAAAACAAAGGTTTTGCGTCGGACAGATTGGTTTTATTAAAAATAAAAAGGTTACCAACTATTTTTAACAGATGGTAACCTTTTTATAATAATTATGACAATTCTAAATTATGTATTTAAACAGCAGGGGGCTCGCCGCAACAAAGTTTGTGGGACCGCATATTTTCACCTCACTTGATCTTTTTCTATTACTTTATTTGCGATGTATATTTAAAATCCTGCTAATCTGATAAATTTTTTAAGCTGATTTCTCCGCGGATTCGCAAAATCGCTTCAGTCAGACCGTGGTAGGATGCGGATTTTTCGTTGAGCAATTCCGATAACCCGCACTTTTCCGCGCATTTTTTGCAGACTTTGACCCCGTCTTCAAAAAGCCCCTCGGGATCTTCAACGATGCGTATATGTAAACGGTGTGTTTTTTTGTAACAAAGAGAGCAGTTGTCCATCACTTCAGTCAGATGGGGTTGAGGAGCATAATTTAATAATTTTTGCAAAATCTTTTCTTCTTTATCCGCAATGGATTGGATTTTTGCTTTCCATTGTGAATTCACGGTCCATCTCCCTTCTTGAATCAGAAATCTTCATTCAAAATTCTATCGCTTTCGTCACTATTATACAACCGATTTTAACCAAATTTTTTAAAAAAACCGGGTTCTTTACCCGGCGAGATATTTGGTGTTTTTAAATTTATTTTTGAGATGTTTTTTCTTTAATATTTTTGGCAGGGTCCGCTTTCAAAAAAATCTCTTCATCGGCCGGCGCCGGTGGCATCGGATGTTCATTCATGATGACAATGACATCATGTCGGAGAATGTTTTTATGAAGGCGAAAGAATGCATGAGAAAAGAAAACGGAGGGATATTATAGACAGGGTGATTCAAATGAAAAAAATTTTTATTGTGATGTTTTCTGCACTGATTGCGTTGACGGGATGTGCCAAAAACCAAGGAAGTCCGGAGCATGATGCCACTCCCCGGCAAGTGGTGGACCAAAACGGTCAGCCCGGGAATGAGCCCATTTCAAGCCGGGGAAGGGTCTTGCCGGATGAAAGAGGAGAAAATGAGCTGAATCTGAAAGGAAAAACCAAACGCATCGAAAGTATTGGCTTTTTGGAAGCCACTTCCCCGGATCAGGCGGTGGAGGATGCATTGGAAGCGGTCCGCGATTTAACCTACGTAGCCTTTTTCAGCTACCAGGTTCGACCGGATGGGAGTTTAATCCCCCTGAAGGATGAAAAACCGCTTCGGGCCATTCAAAACAAGGCCGTTCCGATGCTGGTGCTGACCAATTTTGCTGACGGAAATTTCCAACCCGATTTGGCTCATCAAATTTTTACAAACCGGCAATCCCTCAACCGGTTGATGAATCAAGTCGAACAGGTGATGAAGGAAAAAGGGTACAAGGCGATCAACATCGATTTTGAACACATTTACGAAAAAGATCGTGAGCTGTACCACGGTTTTTTGGAAACCTTTATACCCCGGATGAAAAAACACGGATATGTGGTATCGACTGCCTTGGCTCCCAAAAGCAGTGACGAACAGGCCGGAGCTTGGCATGGGGCGCATGATTATGCGTTTCACGGGAAGATTGCCGACTTTGTGATCTTAATGACGTATGAGTGGGGATGGACCGGCGGACCTCCGATGGCTGTGGCTCCACTTCCCCAGGTTCGGAAAGTGGTGGATTATGCTGTCCAAAAAATTCCCCCGGAAAAAATTGTGATGGGAGCACCGCTCTACGGTTATGACTGGACGCTCCCCTATAAAAAAGGAGGCCCCCCGGCGAAACGGATTGCTCCGCATGAAGCCGAAACATTTGCCAAAAAACGAGGGTTGAAGGTGGAGTATAACAACCGGGATGAAGCTCCTTACTTTTACTACCGGGATGAACAAGGGAAAAAGCATGTGATTTGGTATGAAAATGAACAAAGCATGCAGGCGAAGTATAATCTGATTAAAGAATACGGTCTCCGGGGTGTCAGCTATTGGGTGTTGGGTGAAGATTTCCCGGAGAACTGGAAGCTTCTGCGCCAAAATTTCAAGATCAACAAATACTGATGTGAACGCTCCGTGAAAGCAGCCTGGATGGGAAATCCGGGCTGCTTGTTGCTTCCCGGGAGTTTGAAAATTTGTCAAAGGGATGGTTCCTTGACAAGAATCAGATCTGACAAAATGGAAGGAAAATGGAGCTACCGGTTGCATACGGTGGGGGGGTATCCTATAATAAAGATGTAAGAAGATGTAAAAAAACTTCGTTACAGCAGGAGGAAAAGATAAAATGAAGGAACAAATTCGCGTTGCCGGAATGTCCTGTGAGCATTGCGTCAAATCGATCGAGGAAGCATTAAAACAAATCGGGGTTGCAGCCAAAGTTGATTTGTCCAAAGGAATGGTCGATGTGGATTTTGATGAACAGACGGCCGATTTGGACAAAATCAAACAAACCATTGAAGATCAGGGATATGATGTCCTTTCATAAATTGCATCACAGCAGGAGGCGTTTTCGTTGTCCAGTGAACTCAGCTCGATTCGGTTGCCGGTGGAAGGGATGTCTTGTGCGGCTTGTGCCAACCGGATCGAAAAACGTCTTTCAAAGTTAAATGGTGTTGAATCGGTCCATGTACACTTGGGCATGGACGAAGCCCATATCGTTTATGACCCGAAGCAAGTCCGGTTGCCGGAGATGGAGAAGATCATCCAAGATCTTGGCTACCGCGTTCCCAAAGAAGAAGTGGATATGGAGATTACCGGGATGACCTGTGCGGCTTGTGCCAGCCGGATTGAAAAAGTGTTGAACCGGATCCCGGAAGTTGCCAATGCTTCCGTCAACCTGGCAACTGAATCGGCGCGGGTCGCTTATTACCCGGGGACCTTGGCCGTATCCGACGTGGTGCAACGGGTGGAGCGGCTGGGGTACGGGGCAAAGCCGAAAAGTGCGGAGTCAGGGAAACGGAAAGAGGAAGAATGGGAGAAAAAAAGAGTCCGGTTTTTTGCGGCTTTGGCATTTTCCCTTCCGCTTCTGTGGACCATGGCCGCCCATTTCTCTTTTTTTCATTGGATCCCGGTTCCGTCTGTTCTCATGAACCCTTGGGTCCAATGGATTTTGGCCACACCGGTGCAATTTGTGATCGGGGCTCCGTTTTACCGCGGGGCATGGAAGGCACTGCGCAACCGCAGCGCCAATATGGATGTGCTGGTGGCACTCGGCACCTCGGCGGCCTATTTTTACAGTTTGTATGTAATGTTGACCCAACCGGCTTCCCGCATTCATGAGCAGGGATTGTACTTTGAAACCAGTGCCGTTTTAATCACACTCATTTTGCTTGGCAAATTGCTGGAATCCCGGGCCAAAGGAAAAGCCTCGGATGCCATATCCAAGTTGATGCGGTTGAAAGCCAAAAAAGCATTGGTCTTGAAAGACGGCAAGGAGGTAGCCGTCCCGGTGGAAGAAGTCAAGCCGGGGGATCTGATTCTTGTGAAGCCGGGCGAGCAGATCCCGGTGGACGGAGAGGTGGTCAAGGGCCGGTCATCCGTCGATGAATCCATGTTGACCGGTGAAAGCATCCCGGTGGAAAAACAGTCGGGTGACCCGGTGATCGGCGCCACCATCAACGGGCAAGGACGGCTTCTTTTCCGGGCAACCAAAGTCGGGGAAGAGACGGCGCTGGCACAAATTATCCGGGTGGTTCAGGAAGCGCAAGGCTCCAAGGCGCCGATCCAACGGATTGCAGACCGGATTTCGGGGGTTTTTGTTCCCGTTGTTTTGGGAATCAGCGTGCTTACGTTTATGCTTTGGTTTTTCGGTCTGGAGCCGGGGCAATTTCAAGGCGCCTTGGAGAAAGCGATGGCCGTGTTGGTGATAGCCTGTCCTTGTGCCTTGGGGCTGGCCACGCCCGTTTCGATTATGGCCGGTTCCGGCCGGGCTGCGGAATGGGGCATTTTGTTTAAAGGCGGCGAACATTTGGAAAAGGCGCAGCAAATCACGATGGTTTTGTTTGATAAAACCGGAACGATCACCCACGGGAAACCCGAATTGACCGATACCATCATCTTTCATCCGGAAGAGAGAGAAAACGTTTTGCGCCGGGCCGGTTCGGCGGAAAAAAATTCCGAGCACCCGTTGGCGCAGGCCATTGTGAACGGGATTGCGAAAGAAGGAGTGACATTGCAAGATCCCGACTCCTTTGAAGCATTACCCGGCCACGGGGTCAAAGCCGTGCTGGACGGACAGGAAGTTTTGCTGGGAACCCGCCGGTTGATGGCACAAAACGGAATTCAAACGGATTCAATTGAGGAGGAATTGGTCCGGCTCGAAAAACAAGGGAAAACAGCCGTGATCATCGCAATAGACCAAGAATGCGCCGGGATTTTGGCCGTTGCCGACACGGTGAAAGAAACTTCCCGCACAGCGATCCAAAAGTTGAAACAAATGGGGCTAAAAGTGGGGATGATCACGGGAGATAATGAACGGACGGCCCGTGCCATCGCCAAACAGGTCGGCATTGAGGATGTTTCGGCCGAAGTGTTGCCGGAAGGAAAGGCCGAAGTGGTTTATCAGTGGCAGAGACAAGGGGAAAAAGTGGCGATGGTCGGGGACGGAATCAATGATGCGCCGGCTCTCGCGGCGGCCGATCTGGGCATTGCAATGGGAAACGGAACCGATGTCGCGATGGAAACCGCAGATGTCACCTTGATGCGCGGGAATCTTGAGCAGGTGGCCGATGCGCTTTGGATGAGCCGGAAAACCATGGCCAATATCAAACAAAATCTGTTCTGGGCTTTGGCGTACAACAGCTTGGGAATTCCCGTCGCCGCGGCCGGATTTCTGGCTCCCTGGCTTGCAGGGGCGGCGATGGCTTTCAGTTCGGTCTCCGTGGTGTTGAATGCCCTCCGGTTGCAACGGGTCAAATCTTTGTCATAAGTTGTCCAGTCCATCGGAAAGATGGGCTGTTTTTTATGAATTAATTTGGTGAATTTTCATTTTTTATATTATGATTGAAATAGATTCTATTAACAAAAAATAATAATGCAGCTGTTTGGAGGGGTTTAAATGGAAGAAAAAACCGTTGTCCTGACCATACAAGACGGCGTCGGCCTGATCCGGCTGAACCGTCCCCAAGTGTATAATGCCATCAACCGACAATTGGCCGAAGAATTGACAGCAACTTTGCAAAAGGTAAACGAAGATCCGAAAGTACGGGCCGTGGTTTTGACCGGGGAAGGAAAAGCGTTTTGCTCGGGACAAGATTTGAATGACCGGTCAGCCATTGTCACCAAGGGGGATGAAATTTCACTGGGCGAGAGTGTGCGTTCCCGTTATAATCCTTTGATTCAAGCCATCATGGATCTGAAAAAACCCGTTATTGCCGCGGTAAATGGCGTGGCGGCCGGGGCCGGTTGCAGTTTGGCGCTGGCTTGCGACCTGCGTGTGATCACTCCCCGTACCCGTTTTGTCGAAGCATTTGTCAGGATTGGATTGGCACCCGACAGCGGTTCCAGCTACTTTTTGCCCCGGTTGGTCGGGCTGGGAAGGGCATTGGAGATCGCGATGACCGGACGGGATGTGGAAGCGGAAGAAGCCGTCCGGATCGGGTTGGCCAACCAATTGGCGGAGGAAGACAAATTGCTGGATGAGGCGATGGCACTGGCCAAGCAGTTGGCGCAAGGCCCCACGGTGGCCATCGGATTAACGAAAAAAGCGATTTACCGCGGGATGGAAACGACGATCGAAGAAGCGCTGGAATATGAAGCTGTGGTTCAGGAACAAGCCGGGAAAACCAAAGACTTCCTGGAAGGCATACAAGCTTTCGCCGAAAAAAGAAAACCGAATTATCGCGGAGAATAGCATCCTTTTCAAACAAAAGCCGGTTTTGTTGATAAATCGGCTTTTTGTGTTTACCATCTAAAGAACGGTTAAAGACTTTGGCACCACTTTGACCGTGATGGGGGTTTCATCCAGAATTTCGCCATCCACATGGGCCAGACAAGAGCCTTGCGGCGTGATGGTGAATTCTTTGCCGCGGTGAAAAATCACGGATTTGTGCCGGGTGTGTTTGCCGGAATAAGCCGTCGGGAACAGGCGGAGAAACTCAAAGGAGCTGAGGTTTTGAATGCAACAGAGATCCAGCAGGCCATCATTTTTTTCCGCTTGGGGGCATATTTTGATTCCGCCGGCAAAGTTCGGGATGTTTGATACCGCAATCAGCCAGACATGTTCGTAATCATAGCGCACACCATCCACGGTCAGGGTGACGGGCTGCGGGGTGTACGAACGGAAGACTTTCAAAGCCTCCAGGCCGTAAGTCAAGCGCCCGAACCACTGCTTTTCCCATCTTTTCAGGCGAACCGCATTGGCCACTTTGGCATCAAATCCCGCGCCGCCGAAGCTGATCAGGCAACGGTTGTTCATTTTGGCGGTGTCCACCTGGTAGGTTTGGTGCCGCAAAATTCGTTTTAATGCAAATTCCGGATCCAGGGGGATCTGATGCCCCAAGGCAAAATCATTTCCGGTTCCTGCCGGGATATAACCCATGGGGATATGGGTCCCGATGAGCCCGCTTCCCACTTCATTTACGGTGCCGTCCCCACCGACGGCGACAACGGCTTTGACCGGGTGATGTTGTTTGATGTCATTGACAATCCGGGTGGCATCTCCCGCTTCTTGTGTGAAGACTGCCTGGTACTCCACCCCCAGTTCATTTAATCTGCGATGGATATAAGACCATACTGTTTTCCCCCGTCCATTGCCGGCCAGTTGATTGACCACGAAAAAATACATGGTGGCACTCCCCCTTAAAATTCAGATATGGAATGAGATGGTCTTTTGTGCGGGTTTTGGTCCAGTTTGCGGTAAATCCGCCGGGTGGCCCAAAAATAAAGGGAGGGAAACCATTTGCGCAATTGAAGCGCCCATTTTAACTTTCCGGGAATCACCACGGTTTTGGGACGCTTCATTATTTTTAAGGCACAACGGGCGACTTCCTCAGCCTCCAACATTTGGCCGGCGATCAGGGGAGGAAAATGGCGGCGGGGATTTTCTCCCCTGAAAAAAGGCGTCATGACCGGTCCAGGGCAAAGAACCCCCACCTGAATCACCGGTGCGTACTCCAATTTCAGAGCTTCGGAAAAGCCGATCAAGGCAAATTTGCTTGCACAATATCCGGCGAGGTTTGGAATGCCGGTCAATCCGGCCAGAGAAGCGATGTTGATGATTCGTCCGTGTCCGGCGCGCAGCATGTGCGGCAAGACGTGACGAACCATGCGGACGGCGCCCAAATAATTGGTTTCCATCATCCCCCGGTAGTCATCCGGCGAAACATCCAAAAATCCGCCAAAACACCCATAGCCGGCGTTATTGATCAACACGTCAATGCGGCCGTAAGTTTGGATGATTTCCTCCACGACCTGTTTGACTTCCGCTTCTTTAGTGATGTCACAGGAATAATAAGATCCCCGGTTGAGTTCGCGATGGATTTCTTTTAGACGGGAGGATCTCCTTCCGAGCAGAATCGGGACATATCCCTGGTCCGGCAACTTTCGGGCAATGGCTTCTCCGATTCCGCTGGTTGCGCCGGTCACCAGAACAACGGGGTTTTTCATTCCATTTCCTCCTTTTAACTCATCGGAACCTGCGGAATGATTTTGCCTTCAATCCGTTCAAAAATATCGTCAATGTCTCTTCCGGTAATGGTCAAGCCGTGGTTTTTCAACCCGATCACGGCCCGGGAAGGATCTTCTGCTTCCCGGATCAAGCCGGCAACCGCTTCGGCCAGCTGTACGGTTCCGCATGGATAATTGATTTCCGTGGAAGGGACTCCTTCCATCCAGGCGTGAATATGCACAATGGCTCCGACTTCAGGGTGCTCGTTATAAATCATCCAATGCTCGATGGCATCGACCGAAACGCGGTTGGGCTTGATATTGGAAGGAACGCTCAATTCCATGGCCGACTGGTCTTCATCATACCCTTTCACCAGCAAAATATCCTTTCCGATCCGGCTCAGGTTTCCTTTGTTGACGCCGCTGGCGCTCATCCAGAATTCATTTTTGTCCCGCCGGGCACTCAAGTTGCCGTAACTGAGCCCGCCGATGCCGTATAAACGCTTGATATGGCGGAATTCTCTGGGAGGCAATAATTCCTGGATCGGAAACGGCGCGGGAAGCAGATTCATTTCATCCAGTTTTTTGCCCGCACGGGAGATGCTTTCGCTGGTTTCATCCCCTTCCCACAGGTCTTCGGGAAGATCGGGGTGAAAAATATTGTTGATGACCAGACGGGAAGTGGCCAAAGGACGCAAGCGTTCGAAAACTTCTTGGAAATATTGGTCATCCTCTTCGGGACGGGAGACGGCATAACATCCCTGTTCCAGGGTGATGAAATGCGTGGACGACACGGGACGGGAACGATCAATGAAAATCATCAGGTTTCCCAGTGAACGGATCAAGATCGGATAACCGGTTTTCAGGATATTGGACACGGGCTCATCAACCTCGGTGATGGACACGACAAACGTTCCGTTTCCTTTGCGGCGAAAAGGTTTCGGACGGTTTGGATCCACGAAATTCAACACCAGTTGAAGTTGCGGAAAATCGGTCGGCACCCGTTGAAAACCGTGTTTTTCCATCTCTTGTTGGATGCCTTCGGCAAAGCGTTTTAAATCCGGTGTTCTTTCATAATCTCCAAACAATGCAAAGTGAATCATTGTGCTAAAACCCTCCTTTGATACAAGACCTAGTTTCCCGAAGGATTTGGAAAAATATACATCTTGGCGCCGGGTCTTTCCGGGGATTCAGAAAGTTGAACCGCAGGACCGGAAAAAATGGTATGTTAAATTTCGAGGATTCCTTACTGGGGAGGATTGTTTATGTATACCATCCGAAATATTTATTGCATCGGCAGAAACTACCGGTTGCACGCGAAAGAGTTGGGCAATGAAGTGCCGGAGGCCCCGTTGGTTTTTTTAAAGCCGACCCATTCACTGGCCAAAGCGGACGGAAGGGTTCTTTCGCTTCCGGGAACCAAAGGGGAAGTGCATTATGAAGCGGAGTGGGTTTTAAGGATTTCCCGCCCGGTTGCGTCAGGGACACCCGTGGATGAAGTGGTGAGTCACATGGGGCTCGGCATCGATTTTACCTTGCGGGATGTACAGAGTGGGTTAAAGAAAAAAGGACATCCCTGGCTTCCAGCGAAAGGGTTTCCTAATGCAGCGGTGTTAACGGATTTGAAACCGTTCCCGGGGCTTGCAGCATGCGCATCGAAATCGTTTTCGTTGTTTAAAAATCAAGAAAGAGTGCAAAAGGGAACCATTGATGACATGGTTTTTTCCCCGCAACAATTGGTGGATTACTGCGCGGCACACTTTGGGCTGGGCGAAGGAGACATTATTTTTACCGGTACGCCGGCCGGTGTGGGAAAAGTGGAGGACAACGATCATTTCAGGCTGTATTGGGAAAACGAACCATGGGGAGAATGCCGAATCCGGTTGAAATAAAAAGGAAACCGCCTTGGAGCAAGGCGGTTTTTCAGTGTGAAAACAAGTCAGTTTCCGTTTTTTTGTAACCACTTTTGTTCAGCCCGTGCAAAGTTGGCGGGGCCGTCATGCTCCAAGGCATCATAAACGAATTCATATGCATGGGAATCAAAGGCGATACCGAGGTGAGCGGCCAGATCGGCCGGCACTTGATCCTGAATCGTAATATTGGACACCTGTTCTTCGGGGCCATCCAAAAAGGCGGAGGTGTAGGGAACGACAATTTCATCGTATTTGGTGCTGATCACGGTGTATGAAACATCTCCCGGGGTTTCATCGCCGGCGTTTAAATCATTCAGGAAGTCAGAGCCGGCTTTTTGTTGATCGCATGCCGTGCATCCCAAAAGACCCGGAAGAATCATAATGGGTTCAATGCCGAGAACCCCGATGGTCCCGTGGTTGGATGGAGCCAGTCCAATCAGGTCTTCGACTTTTTCCGCACCGCCAAGGTATTTGATGTAGTAGCGCGGCATCATGCCTCCCTGGCTGTGCCCGACAATGGAAACTTTCTCCGCGCCGGTTTGCTCCAACACCTGATCGACAAACGCTTTCAATTCTTCAGCAGATTTTTCAATCGGTCCGGTACTGAAGCCGAGATGGGTGATGCCGTAGTTTAAAGCATACACGCAATAACCTTTCTTCGCCAAAAGGGGTGACAGTTTGCTCCAGTTAAATGCCATGCTTTCAAAAGTGCCCGGCACCAGGATGACCGGATAAGGATGTTCCTCGCTCGGCTTGCAATCGGGAAGATTGGCGCCGGGAGGTGACAAAGCGGCTTCGCGCAGGATGTCTTCATCCGTTTGGGCATGGGCGGATGAGATCCCAAAGAGACAAGGAGCCAACGCTAACACTGAAATAATGAATATTCTGAAAATAAATCGGAAGGCAGACATCGTCTCTCCCCCTTCTGTGGATTGTTAATACTTATCTATATTAAATTTTATCTTACTGAAACAAATCAGGGAAGGGGAGAGAGATAAAAAATCAGATTATTCAATCATTTTTTGCGACATGGTTCCTTGGAAGATTGGAACCATTTCATTTTCGGGGCGATTCACCGGGAACGGGGACAAAGTCGAAGATTTCTCCGCTTTTAAAGGTGTTTGCCAACTGGCGGAGCCAGGCATAATACCGTTTGGCATTGGACAGTTTTTCAAGGTCTTGTTCCGCTTGTTTTTTCTCTTCATCTTTGACCCCGGAATCATGAAGCAGTTTTGTCAGCTTTTCTTCACTGGCACGAAGCGCTTTCTCATTCAGCTCAATTTCTGTTTCCCACAGGTGGACGAAAAACTCGATAAAAGACCGGAACTGGTCTTGTTCGGCCTGGTACAAATCTTTCCGGACGCCTTTGATCCATTTTTTTTGCACCAGTTTTAAACGGGATAATTCCCGTACCCCCGTACTCATGCTTGTTTTGCTCATGCCGGTGCGCTGGCGCATTTCATCCAGTGTCATCGGTTCCTGATGGAAAAACATGATGCCGAACAGCCGGCCCATGGAGGGACTGAGTCCGTACATCCGCATCGTTTCAGCCAATGATTCGATGTAATCGCTTCTTGCCTCTTCCAATTGATCCATCGCACATTCCTCCAATCGCTTGCGTGTCCTTTTGTTTAATCCAATCATCCGAAGCAGGTGCCCACATCTTAAACATAAGGAATGATTTTGTCAAACAAAAGGGAAGGTTGCGGAAGAAAAAAATTCATGTTAACGTAATTTATATACAGTTTGTACAGAAAAAACTGTACAAAAGGAAATTGATGGTTAAAGGGGGAGGGGAAATGTTCAAGCGCATGTTCATCGGCGGAAAGTGGACCGAAGCCGTCACGGGGGGAGTGCGGTCCATTTTCAATCCGTTTAACCAGGAAGTGATCGGAGAAGTGCCGGAAGGCGGGCGCGAAGATGCGAAAAGAGCCATCCAAGCCGCCCGTCAGGCGTTTGATAAAGGAAGCTGGCCGGATACGCCGGCTGCTGAACGGGGAGAGAAGTTGTTCCGGCTGGCGGAGGAGATACAAAGGAACAAACCGGAACTGGCCCGGTTGGAAACGTTGAATACCGGAAAGACACTGACGGAAAGTGAGGCAGATATGGACGATATCGCCAACGTGTTCCGGTATTACGCGGGGCTGGCGGATAAGGACGGGGGAGAAATGCTGTTTCCTCCGGCCAAGCATGCCGAAAGCAAAGTGGTTCGTGAACCGGTCGGCGTTTGCGGAATGATCACCCCTTGGAATTATCCGTTGCTACAGGCGGCTTGGAAGCTGGCTCCGGCTTTGGCTGCCGGTTGCACGATGGTGTTGAAACCGAGTGAACTGACTCCGCTGACCACGTTGAAGTTGGCGGAATTGATCGAAAAGGCGGGCATTCCTGAAGGTGTGGTAAACATCGTGACCGGACCGGGGGAAACCGTGGGGGCCGAACTGTCCGAATCGCCGGACGTGGACCTGATTTCGTTTACCGGCGGAATCGAAACCGGCAGAGCCATCATGCAAGCTGCCGCAAGCAATATGAAGAAAATCACTTTGGAATTGGGAGGAAAAAACCCGAACATCGTTTTTGCCGACAGCGACTTTGAGGCGGCGGTGGATTATGCGCTGAACGCGGTCTTTTTCCATGCCGGACAGGTGTGCTCGGCCGGGGCCCGGCTGCTGATCGAAGAAACGTGGCATGATGCCTTTGTCGGGGAACTGGTGAAAAGAGCCAAGCAAATCCGGTTGGGCAACGGGTTGGATCCAAAAACACAAATGGGACCGCTCATTTCAGCCGAACACCGGGCCAAAGTGGAGAAATATATCGGGATCGGGCAAAAGGAAGGAGCCGTTTTGTTAACAGGTGGAAAACGGCCTGCATCAAAGGAACTGGCCGGCGGCTTTTTTGTGGAACCGACGGTGTTTGACCATTGCACAAGTTCCATGCGCATTGTACAGGATGAAGTCTTCGGCCCGGTTTTGACGGTGGAAACTTTCCGTACGGAAGAAGAGGCGGTGGCTTTGGCCAATGACACCCGTTACGGGTTGGCCGGGGCGGTGTGGACCGGCGATATGAACCGGGCGGAAAGAGTGAGCCGGGCATTAAGGATGGGAACGGTTTGGATCAATGATTTTCATCCGTACTTCCCGCAAGCGCCCTGGGGTGGATATAAACAGTCGGGAATCGGAAGGGAACTGGGGAAAACGGGATTGGAGGAATATACGGAACAAAAGCATATTTACAAAAATTTGCAACCTGCCCCCATGCGTTGGTTCGGATAAAAAAGACAGGAGGGAAGCACCATGCTGCGAACGGAACAAATGTTGGAGAAGAAAGAGTTTGTCATGCCCACCAAAGTGATTTCGGGCATCGGCGTCTTAAAGCACGTGGGAGAGGAAGCGAAGGCGTTGGGCATCCGGAAAGCGATGATCGTCACCGACCAAGGTCTTTACCGGAATGGACTGATCCAACCAGTTGCCGAACGGTTCAAACAAGCCGGCATTGATGTTCATGTGTTTCATGAAATCGCGGGTGAACCGGACACCTGTTTAATCGAATTGGGAGCGAAAACGTTTCAGGAGCAAAAATGTGACGGATTGGTTGCGGTGGGCGGCGGAAGTTCCATGGATACCGCCAAAGCGATCGGAGTGGAAGCCGTTCACGGAGAACCGGTGATCCGGTTTGAAGCTTCGGAACGGGGCAAGCCACTGGAAAAACGGATCCCGCCGCTGGTGACCATCCCGACCACGGCCGGCACCGGAAGTGAGGTCACGCAATGGGCCGTGATCAAAGACCCCGAACGGCAAATTAAATTTAATGTGGGAGGACCTTTGATCCCTGCCCATCTTGCGGTGATTGATCCGGAACTGCACCTGACGATGCCCAAAACGATTACGGCGGTCACCGGGATTGATGCCTTATCCCATGCCATCGAGTGTTACACCATGAATCAATCGCAACCGTTGACGGACGCAGTGGCATTGATGGCCATTGAGTATGTGGCCAAATACATTCGCCGGGCATATGCCAACGGTCACGACATCGAGGCGCGTTATCACATGGCCCAAGCGGCGATGTTGGCGGGCCTTTCGTATGGAAGTGATTCAGCGGGAGCGGCCCACGCGATGGCCCAAACATTGGGAGGCATGGTGCCGGTCATGCATGGACAATGTGTTTCGGCCATCCTTCCGGCCGTGATGGAATATAACTGGATGGGAGCTCCGCAGAAATTTGCCCGGATCGCCCAAGCGATGGGGGTTGATATCCGGGGCATGAGCACCGGGGAGGCGGCAAAGGCCGCGGTGAAAGAAGTGGATCGTCTTGTCCGTGATTTAAACATTCCGTCTCTTCAAGAACAAGGGGCAGATCCCGGACAAATCGACCGCTATGCCGAAGCGGCACTGGAAGACCCGCAAACCTTCGGCAACCCGCGTGATTTGGATCTGGATGCTTACCGCTGGATTTACCGTCGCGCGTTCGGGTTGGAAAAATCTGTTTTATAAATCAAATACACCCCTGGTGATTCGTTACCAGGGGTGTTTGTTATTTTTAAATCGATCTGGAGAGGACATATTCCACGACGGAGATAATCTCCTTTTTTTGCTGTTCGTCCAACACCCTCCCTTTCCACAGAGCTGTTTCGTTTTCGGAGGACAAGATTTGCCAGATGTCGCATGGGTTGCTGTCCCCGTTTGTGTTTTTCTGTAACAAGTCTTCATACCCGCTGGCCCGCATCAAATCTTCAAAGGGGTATTGGTATGCATCGGCCAATTTTTTCAATACATGGGCCGAAGGTTTGATCGGTTTGCCTGTGGCGCGGCGGATGCCAAGTTCCAAATCCCGGATGTAGGTGTGGGAAAGTCCGGTTTTTTCCGAAACATCACGCAATGTTCTTTTGCCGCGGAGTTTTTTCAGTAAAATCCCCAATTCGCTGCGCATGGTTGAATTCGTTTCCTTTCTGAACAGGGAATATTCCACTTTCATTATAAGGGAAACCGGGCTTGAAAATAAAAACTGATGGCCGCTGAAACCGTGAAGAAGGGATCAGATTGGGGAGTGGGAGAGGGAAAGGGCATGATCCTGAAGTGCAGAATGAAAAAAAGGGGCTTAAAGGGCGGTAAATCCCTTGCCGGACACCCAAGGTTACAAAAGTTTGGTTTTGTAACGGTATAAATCAGAAGCAAAAAACGCGAAAACCCTTGATTTATCTATGGTTTTGGCGTTTTTTCATTTACGCAGGGCATGGGAGGGGGTCTTTTGTAAACTCGGTACAACAAAACATCGCCTGAAAAAACGGCCTTCTTATTTTGGTTTTAAGGGTGGTTTATTTTCACATCCATCCAATGAGTCGAAAAAGGGGGATGAGCGCTGTATGACCCCTTGGAGTCCGTCTCATGTGTTTGCCAATGATTTCCACGAAAAAACCGGACAAATGCGTCCGGTTTTTTCAGAAAATATTATTCTTTGCTCACGGTTTCCAACGCCATGATGATCATGTCGTTAAAAGTGGTTTGCCGTTCTTCGGCGGTGGTTTCCTCTCCGGTGAAGATGTGGTCGCTGACCGTTAAAATCGAGAGGGCTTGCGCCTGGTGTTTGGCAGCCAGGGTGTAAAGGGCGGTGGTTTCCATCTCGACGGCCAGAACGCCGTGTTCTCCCAGTTTTTTCACCATATCCATGTTGTCCCGGTAGAAGACATCGGACGTCAAAATATTTCCGACGCGAACGGATAACCCTCTTTCCACCCCGGTGTCATACGCTTTTTTGAGCAATTCAAAATCGGCGCACGGGGCGAAATCAAAACCAGGGAAAGTTTGCCGGTTCATGTTGGAATCCGTGCAGGATGTCATGGCGATAATCACATCACGGACCCGGATGTCTTTTTGTATTCCGCCGCAGGTTCCGACGCGGATCAATTGCTTGACGCCGTATTCCCGGATCAGCTCATTGACATAAATGGAAATGGAAGGCACCCCCATGCCGGTTCCTTGTACCGAGACGCGGGTTCCTTTGTATGTACCGGTAAAGCCGAGCATGCCGCGAACCTCGTTGTAACAGGTGACATCTTCCAGGAACGTTTCCGCAATATATTTGGCACGAAGGGGATCGCCGGGCAAAAGGATTTTTTCGGCAATCTCTCCTTGTTTGGCTCCAATGTGAACACTCATGTTGACACCGTTTCCGTCAATTCCGGACCGGTGTTCACCTCCTTTATTCTCGATCTAAATATAACTATAACAGGAGAAAGAGGCATTTGAAAGAAAAAGGACTTGTGAATTGCATACAGATTTAATATATAAACAAGTTTGCTTTGCAAACAAAATAAAACCACAAAAGCGATCCGTGCCCCAGACGAAATCTTTTTTCAATCAAAGGATGGCATCCGGCCGGTTGATATAAAATGATAATATGGGAATGAACCTTTTTTTAGCGATGCAGTTTTTGGAGGGGTACAGATGAACGAAGTGATCAAAACGATAATGAACCATCGCTCCATTCGTCGATATGAAGACCGTCCTTTGACGGAAGAACAAATCCGGACCATTGTGGAAGCGGCCCAATCTGCTTCGACGTCCAGTTATATCCAAGCATACACGATCATTGGCGTTAAAGATCCGGCCAAAAAGAAAAAGCTGGCGGAGTTGGCGGGAAACCAACCTTATGTGGAGAAAAACGGCCATTTGCTGATTTTCTGCGCGGATTTGCACCGGCATGATTTAATCGCGGAGATGGAAGAGGCCGATTTGACCACCAGTCTGGAAAGCACCGAAAAATTCATGGTGGCCGTGGTCGATGCGGCACTGGCGGCCCAAAACGCAGCCATCGCCGCGGAGTCGATGGGCTTGGGCATTTGTTACATCGGAGGGCTTCGCAACAACCTGGAAGAGGTGACGAAGCTGCTCAACACCCCCCGGCGGGTTTTGCCGCTTTTTGGCATGACGATTGGTTATCCTTTGCACGAATCATCGAAAAAACCCCGTTTGCCCTTGGAACACGTTTATCATGAAGAAGAATATGAGCAGGATGAACAGCGCTTGAAAGAACAACTGAAAGAATACAACGAAACGGTCCAATCCTACTATATCGAGCGCACCGGAGGAAAACGGGAGGACACTTGGACGGGACAAATGGCGCAAATGCTCTCCCACCCGCGAAGAATGTACATGAAAGAATTTGTGGAGAAAAAAGGCTTTAACAAACAATAACGGTGAAGACCAAAATGGCTTGAGTGCATGGAGGGTGCTCAAGTCATTTTGGTTTCATCGCTGAACAACAACCGGGCCATGTAATATTCATCCGCATACCGGTCCTGGATAAACAAGGATTTTTTTCGCATCCCCTCGATTTGGAACCCCATTTTTTTATAAAAGTAGACGGCTTTCAGATTGGGACGCAGGATGGAGATTTCCAACCGCTTAATATCTTGTTCCCGGGCCCACTGAATGGCTTTTTCCAGCAACCGGGTGCCCACCCGTTGGTTTTGGTACTTTTTCAAAACCCCTGTCTCCGGACGCGCGGTATGCCGGTTGTGCGGATCATCTCCACGCCGGATGGCAACATGGCCCGTCAACTTTCCGTCCGTTTCCGCTACGAAAACGTGCCGGTTGCCGTCGCTGATCTTGCCCGGGTCCGCATCGCCGCAAGGAAACAAAAGGTACGGACTTTCCTCATTCAATTGGTTCAACAGCCGAAGGTAAGCCTCATGATCCTCCGGGCAAATTTCGCGTATCTGCAAGCGCCTCCCCCCCTCTTAAGCACAGATCCTTGTCTCCCAACCATCATATGATCGTCTTACCCGGTTAATTCGAGTCTGTGGTGAAACCTATTTCCATTTTGAGTTGGTGACTGGTTGGACGATGATCCTTTGTGGCCGGCAGGGGAGCTGTTGCAGGAAAACCAGCCGGAATTGAGTTAATAACGGTTTGAAATTTTTCAGGTGGGTTAAGGCTGATTATCAGGTGGAGGTTTATTCCGATTGTGTCAATGCAGCAATGCATGCTTCCCGTATTTCCCACACTTTTCTCCAGTCCTTAATGGGTACACTCGGGGATGTCACCCAGTGTTTCAAATAATCTTGATGAAACCGGTTTTTTCCTCCCCGATTCCTTCGGGTGTAATCTCTTTTTCAACCAAAAATGATAGGGGGGTCCCATTTGTTTGACCGGTCAGACGCGTGAAATCCAAATACTGTCCCTCAAACCGCAAATAACAATTTGCTTCCGGGAGCTCCTTAAGGCCGTAACGTTCCAACACCTTGCCGACACCGGGGGTGTTGTCCTCATTCATCAAATAAATGCCGATCATCAACCGGACGGGCATGTTGTTTTCCTCCGCCAAGATGGCCAACAGCGCGTGCTTCATACTACAGGTCCCTTTCCCCTCGCGCAAGACCGATCGCGCATCTGCCCGGTTGGCATTTCGCCCGTAAGGCAACGCCTGCAAATAACAGGCGGCTTTTTGGAACGTTTGAACACCCAAAGAGTGAAACGCTTGGGACATTTCTCCTGCCGGTTGCAGACAAAAATCCAAACGTTGTTTAGCTATTCATTTCCTCACCCTCACAAATAAGATCCATTTGATTTAATCATACTTTTTCGATATTCATTCTAAAAAGTTCCTTGAATAAAAATATTCTCGTTAACCTGATAGGGATAAATTGGAACGAAGTGATCTAAATCATCGCTTGATCCGGAAATGCGAGGATTGTCCGTTTTCCGTTTGTTGATTTTCTGTGCAGGGGCATTGTCATGAATTGATCAGGGAGATGAGAGGGGGAAGAGATTTGACCGCCAGTCTGGAAAGCACCGGGGAATTTTTGGCAACCGTAGCAAAGAATCCTGAACGTGGTTTTGAATTTAACATACCGTCCGATCTTTAGTCTCTGAGATGGGTCAGTTTCTCTTAAGGTGCAACAATGAGTAAAGCAAATGGACATTAAAAAAGGAGCCGTTTGCGGCTCTTTTTTAGTTAAGAGGTTCCACAATGGATGCAATTAAACAGATTCGTTCGTTCCCGTTCCTAAAAGGGGACGTGGGTTTCAATTCCACAATGGATACAATCAAACCCCTTGCGTAAACACCAGTTATATCAAGCACTTTAAGTGTATCAAAAATCAAGATTTGCAGCAAACCACCGGTTGTGTCCGGCAAAGTTTCATGCGGAGGGCGATTGATGTTTAAAGAAGCTGCCAGACGAACGGGCAGCTTCTTTAAAATCAAAGGTTTGATATGTTCTGCTGTCTCAGCCGGATAAAAACTTTCCGCCACACCACCCATGAAGCAGCTCCCAGGATCACTGAGGTGAGAATGCTTCCTTCAATCCCGAAAGGGCCGCCGTGGAACCATTCGGGGCCGTGTGTCGTCGCGTGAAGAAGCGGGAAGGACAGGCTTTGTCCGCTCACAGCCAATCCGAAAACCGAGCCTTGTGCCCAGTTCCAAACAGAGTGAATGGCGCAAATTCCCCACAGGCTTCCTTCGTTTAAAACGTGACCGACGGCAAACAAGCCGAACAGACTGATGTTCAAGAAAGAAATCACGTTGATCCCGTCATTTAACAAATGCATGGCGGCAAAGAACAGAAAGGAGGTGCCCACCGCAATCCAGAATGGTGTGCGCATGGCCATGACAGGGAACAACCAACCGCGGACCACGATTTCTTCCGTTGCGCCCTGGATGATGAAGGTGATCATGGAAAGCAGTAAAATGAGAAGCGCGGGGAGATGGATTTTCGTGATCTCAAAGGTCAGAGAGCCGGTGATCCAAAGGAGGAACACAGGGGCGGAAATGAGCAAAAAACCCCACATCCACCCGGTTATGTACTTTTTAAAAGCCCTCTCTTTCGTAAAGCCGATGCTGGAGACCGGCCGTTTCTCCACCAACCGGACCCAGGCGAAAGTGAACAGTGCGGTTCCGCTGAAGGAAACGATCAGTTCCAAATGAAACCAAACACTTTGCATCAGCGGGGAAACAGGGGGCGGAAACAAAATGAGAAAGAAGATCCCGAGCCCACCGAAAAAAAGGAAAGAAAAGGCGATGACCGGGGCCAAAACCGGATGAATTTTTCTTTCGCCGGTTGCGGCCAGTTTTAACAGGGTAAACGTTTGGAAGTTCATAATCGGTCCTTTCGAAAAGTAAAGTTTTCAATATAATCTATCATATATGAAAACGACAACAAAAACCATCGGTGATCCTTTTTCTGTCTAATCGGTCAAAAAAAGTGTATACTAGTAGGGGATAGAAGAGAAGATTTTTTCTGACAACTGGGGGACTATCAGTGTTTAAAAAACAAAAGACGAGTTTGTATTCCAAAACGTTGATGGATATTACATCCTTGATCGTGGAGGCGGCTGAGCTGTTTCGCGAAAATGTGGAAAACCTTGAACAACGGGAGCAATATGGAGAGAGAATCAAACGCCTCGAGTCCAAAGGGGATGAATTTGTCCAGCTTTTGATTTCCCAATTGAACAAAGAATACTTCCCGCCGATGGACCGGGAAGATATTTTCCAATTGGTGGCCAAATTGGATGACATTTTGGATGGCATTGAGGCTTGTGCCGAACGTTCGTTGTATATCGAGAAGACGACACCGCAGCTGATCCGGTTTGCGGATATTTTGGTGCAAACGGCCAAAATTTTGCAGGAAGCATTCACCTCATTAAACGACCGCCGGTTCGAAGCGCTTCATAAGGCGACCATTGAAGTCAATTCCTTGGAAAGTGAAGCGGATCAATTGTTGCGGGACAGTCTGAGCGAATTGTTTTTTGATCCGAAAGATGTGGTCTTGCTGATCAAAATGAAGGATATCTATGAGCGGCTGGAAAAAACAACCGATGACGCCGAAGACATTGCGGATGTTTTTGAAAGCATGATCATTAAATACGCATAAGGAACGGATAAAAAAACACCGGCTTTACGGCCGGTGTTTTTTATATAAACAGGCGGACAATTTGGGTGATGGTGAAGGTGACGGCAACCGCGATGGCGGTGGGAATCAGTGCGGCCAGGAGAGACCATTTGACGCTTTTGGTTTCTTTATAAATGGTGAACAAAGTGGTCCCGCACGGATAATGAAGCAGGGAAAAAAGCATCATATTGAGTGCCGTCAGCCATGTCCATCCGTGGTCGAGCAGGATTTGTTTCAGATCGGCCAACTGATCAACTTCCACCATGGAGCCGGTTGCTAAATAACCCATAAGCAGGATGGGAAGAACCACTTCATTTGCCGGCAATCCGAGGATAAAGGCGAGCAAAATGAAACCATCCAGTCCCAGCGCATGTGCAAACGGGTCCAAAAAGCGGGCGGCATGTTCCAGAATGCTGGCCGAACCCATATCGATATTGGCCAGGATCCAGGTGATCAAGCTGGCCGGTGCGGCGATGATGACGGCCCGTTTTAACACAAACAAGGATTTATCCATGGTGGCCCGGTAAATGGTGTTCCAAAATTTCGGCCGCCTGAACGGCGGGAGTTCCAACGTGTAATGTGTGGGAACCCCGCGAAGTGCGGTTTTGGACAAGGTCCATGACACAAAGAGCGTGACGACAATGCCGAACAAAACCAGTCCCATCACCACGGCGGCGGTGACCATGGTGCCGATGCCGGAGCTGAAACCGGCTGCCATGAATAGTGATGCCAAAAGGATCAGAAGGGGCCACCGGCCATTGCAGGGAACAAAATTGTTGGTCAAAATGGCCAGCAAACGTTCCCTCGGGGATTCAATGATCCGGGTGGAAACGATGGCCGCTGCATTGCATCCAAATCCCATGGCCATGGTGAGGGCTTGCTTGCCGTGGCCGCCGGATTTTTGGAAGACCCGGTCCATATTGAAAGCCACTCTGGGCAGAAATCCGAAGTTCTCCAACAGTGCAAACACAGGAAAGAAGATGGCCATCGGCGGCAACATGACGCTCACCACCCAAGTGGTTCCCCGATAAAGCCCGAACACCAAAAGATCATGAAGCCAAACGGGGGAGCCCAAAGCCTGAAGGACTTGGGAAAGAACCGGTTCCCCCGCGCCGAACAGACGGGCCAGGGCTTGGGAGGGAAGATTCGCTCCCCAGATGGTCATATAAATAATCAAGCCCAGGATGGCCAGCATGATCGGAAATCCCCAAGTCCGGGAAGTCAAAATCCGGTCCAGTCTGGCGTGGGAGGGGGGGATCCGGTGTTGCCGGGTGTAGCGAACGCTTTCCTCGCACAGTTGATGTGAGTTTTGATAAATCCGGCTGACAATCTCGTCGCGGATATTTTCGCTTCGTAAGGATTTGCTCTGTTCCATCAAATGATCCAATTCGCCCAGAGGAAATGCGGATGAATTTGCCATGATCCTTTCCTCCTTTTGTTTAGCCGTGAGCACGGGTGGCGCGGGCAGGTGGCTGTTTTCGGGCAAATTTTTGTTTTAACACCCGAAGCAAAGACTCATCGCCCTCCAACAAGCGCAAAGCGAGCCAGCGGGCGGGGAGGGAGTCGCCGAATATTTCTTTGATTTTCGGTTCGATGCGTTGGATATGTCTTTCAATATTCTCGCTGTACTCAATCCGGTAAGGTCGGGTGATGATTTGTCCGCTTACCATCTTGTGCAAGGTGTCAAGCAACCGGGTGATGCCTTCACCGTTGCGGGCGGACATTTTGACCACCGGAATGCCCAGTTTGGACGCGATTTTTTGGTCATCGATGACAATGCCCTGCTTGGAAGCCTCGTCGATCAGGTTAATGCACAAAATGACCCGTTCGGTCATCTCCAAAACTTGCAAGGCCAAATTCATGTTCCGCTCTAATGAAGTGGCATCGACGACGACCATGGTCACATCGGGTTGTTCGAAAAAGATATAATCGCGGGCCACTTCTTCATCGGCGGAGTTTGAGAACAGGGAATATGTTCCGGGCAAATCGATCAAGCGATAGTCGTTTCCTTTATGGTGAAAGGTTCCTTCGGCAAGTGACACGGTTTTTCCCGGCCAATTTCCGGTATGCTGGTTGAGTCCGGTCAGGGCATTAAAAAGAGTGCTTTTTCCCGTGTTTGGATTTCCGGCCAGTGCAATCCGGTATTCACTCATTGGTTTCATCTCCCAATTTTTCACCCCATATTTTTTGGCTCTCTTCTTTTCGGAGTGCGATGGTGGATTGGCTCACCCGATAGGCGACCGGATCGCCCAAAGGACTTTGATACAACACTTCGACCAACGCCCCCGGAATGAAGCCGAGATCCAGCAGTCTGCGCCGGAGGACGCCTTCCACACGGATTTCCCGGATGCGGAAACGATCCCCCGGCTTTGTATCAAAAAGAGTATGATGATTCATTTTTTCACACCTGCTCTTTTTTTCTATTGAGACAAATTTGTTGGTTTAGGGCAACATTTAATTTAAAGAAAAACAATTATACTATATAAAGTATATATAACTGTAGATATAAAATCAATATGGTTTTGCAAGATATGTATCGTCTTTCACGCCGTTTGTCAGGAGGTTTGCGTTTGGTGCGATTCGAGTGATACCATTTTATGTGGTATGATGACAATGTGTGTCACATCCCTTTCACTTTTGTGAAGATTATCCATAAGGGATTTCATGGTAAGATAAAATACGGAAACAGGCTCTTGTTCAGAATGGAGGGATTCCGTGTCTGCGTCACAAATTTTATTCCAAATCTTTTCGTATCCCAGCAATTGGAACTATCAATTGAATCTGCTTTTCATCCTGATTGCGGTGGTTTATTTGTTGATCACCGGTCCCTTGCGGCATCGGTTCAAAGATGCAACTGAGGTGAAAGGATCGAAGAAAGCGGTGTTTCTGACCGGCTTGATTGTTTATTATTTTGCCGTGGGAAGCCCGCTGGCCCTTTTGACGCATGAGTTGTTTACCATGCATATGCTGCAAATGGCTTTGATGTTTTTTGTGGTTTCACCTCTTTTGTTGATCGGGATTCCGGTTTATTTATATCGTGCTGTTTTTCGCATCAAGCCGTTGAAAGCGGTGTTTTCGTTTTTGTCCCGTCCGTTGTTGACGGTGTTCCTTTTTAACGGTTTGTTGTCGTTGTACCATGTGCCGGCGGTTTTTGATACGATCATGAACAGCGAGGCGTTGCACGTGATCTCGCACACGCTCCTGATGGTTGCGTCCCTTTTCATGTGGTGGTCGATTGTATCGCCGGTCCCGGAAATGGATACCTTAAGAGACAGGCATTTAATCAAAATTGCGTTGATTCTCGGGGGGACGGTCTTGATCACTCCGGCTTGTGCACTGATTATTTTCACGGATACGGTTTTATTTGAAAGATTTCAAGAGACCTCCCAAATGCTGCCGCTGTTTGCTCCGCTGATCGACCAGCAATTGGGCGGGGTCATCATGAAAATCATCCAGGAGATTTCCTTTATGGCGGCCATCGGCACCGTTTTGGCCGGCTGGTTTAAAAGCGAACGGAAAAAAGACCAGGAAGATTTGATGCAAATTGAAAAGGAGCGGCAACTCAGATTCTCCGAAAATCAAGTGTGAAGGTGTGAAACATGAAGAAGATCCGAATCGCAGGTTTGATCGCGGGAAGTGCCATCTTGCTGGCTGCCTGCGGATCAAACGGAAATGGGCCCAATCAAAGCGGACAAGTGACGGAAGGGCCGGAAAAAGTGTATATGCAGCACTGCGCCAATTGTCACGGTGAACAATTGCAAGGAGGGTACGGCCCGGCACTCAAAGAAGCGGGAAAAAAATATTCTGAAGCTGAAATCTTGACCATTATTCAAAAGGGAAAAGGAAGCATGCCTTCGCAAGGTTTTATCAATGAAGAAGACCAAAAACAGCTGGCCAAGTGGCTGTCGGAGCAATAGGGACAACGTGTCTTTCATCAACCTGTTAGGGGTTGATGTTTTTTTATGCGCAGATGGAAGCCGGATGAATATAATGGTACGGAATGACAAGAAAGAGCAAAGGTCGATCGCAATGGAAGAAAAAAAGAATTCCTTGTTGATCCGGTTTCCCTCTTCGATTCCTTCCGCATACATGGCGCAGTTTCCGGCCGGACAAGGGATCCCGGCAGCCGACAGTCGGGAAGCATGGTCCTTGATCAGCCGGGAGCCGGAGTGGACGAAGGTGGAATTGGTGAATCCCCCGGCGGCGGTGTTAAAGCGGTTGCTGGAGGAAGGCCGGAATCCTCAAAACATCGTCTTGTATTATGATCATTTGCCCCAAGCGGAAGAGGGAACGTTTCTCCGTTCTTTCCATGTCCGGGTTCCGTCGCTTTGTTTTTATTATTTCTTCCGGCAACGGGAGGTGGACGTGGAGCTGATTCATCCACAGCCGGTTGAACCGCTGAAAGGCTCCGGGGACGGGGAAGAGGCCCCAAAAGACTTGAAGATGAAAAACAAAGTATGCTTGTGGATCGATGACCGGTTGGTTGCCCCGCGTTATCGCAAAGGATTGGCGCTGACGGTTTTGAAAGTGCTCAAGCAGTCGGATGATCTTGAAGTGATTTGGGCCGGGCCGCACCGGGAGGATTTTGATCAGCGGATCCGGGTGGTTCCTCCGGAAGAAGCAGACCGGAAACATCTTTGGCTGGCGGCAGATATCGTGGTGACATTGAGCCCGGTTGCCCAGTCGATGGCCCCGCTTCATGTCCGGTGTTTGGCGAATCAAAAAGCGGTGATCACGGATGACCGGGGGGATCATGGGGAGTGGGTGAATCATGCATTGACCGGCTTCTTATTGAGTCAAAAAGGAATGTTTCCGGAGTTGCGCAGGTATTTGCTGCGGTTGTTGCATCAGCGGGATATGCTGATGCGCTTTCAGCAAAACGGACCGCTTATGGTTGAAAAGGTGCTGAAGAAAAGATGAAACCCGAAGTCAGTTTGATCATTCCCCACCAAAACGATTGGAAACGCATGCGGGTTTGTCTGGAAGGGATTTTGGCGTATACCCATGCGGAATATGAAGTGATCATCGTGGATCATTGCCCGGGGAAGATCCCGGAAAAATTCCTGCAACGCCCTCCGTTTACGGTCATTCAACATCAACCGGATTTGGGGCGGGCCGCTTCACTCAACTTGGGAATGGAAAGGGCGAGGGGAGAGTACATTGTCTGGCTGAGCCCTTATGCCGTTCCTTCACACCGTTGGTTGGCACAGCTGCTTGCCGTTTTCAAAGCGAGGGAGTGGACGGGGATGGCGGGGCCGGTTACCAATCGCGGAAATGCCAAGCAAAAATTAAGCATTCCCTTTAAAACCATCTCCAAAATTCACCGGTTTTCCAACCAATTTAACCACAGCAATCCCACGAGGTGGAAAGAGACCGAGGTGTTGTCGGGATTTTGTGTGTTGTTCCCGCGAAGGCTGGTGGATGAAATCGGATGGCTGGATGAACGGTTTGGAGATGGTATCCACGCGGAGGCGGATTATTGCCGGCGGGTGCGGAGAGCGGGGTATTCTTTGATCATCGCCGCCGATACGTATGTTCATCAGCTGGCAAAAAAACCGTTGAGCAAAGAAGAAGTGCGGGATCAGATCAAAATCCGCAAACAAAACCAGAAATATTATGAGTACAAGTGGTTTTATTTACAAGATCACAATGGAGGCCAATCAACGCTTCATCCGTAAAATACAAACCTTGGCTGGTTTGTATTTTTTTTATCGGGTTATATATATTTCGGGGTTTGCAATCAGCCGGAAAATGGCTGCTGGACAGGCTGTGTCGAAAGGGGGATCGGGGGATGATTGGTGTTTTCATGGTCGTGATCGTGTTATTGGGCGGAATTCTTTATCTTTTCCTGAGTCCGAAGGTTCCGTCCATCAGTCCGGATGATTTGAAGAAGGTCATGGAAAACAACCAAAACATATTAATCATTGATGTCCGCGAAAAACGGGAATATGAGTCCGGACACATCCCCGGAGCAGTCCATGTTCCGTTGCGCCAGCTGGAACGTCTCAAAAAAGAGGCAGATCAACAAGAAAACGGCGGGGGAGTGTATATCTACTGCCGCAGCGGTTCCCGCAGTGCGATTGCCGTGCGTGAATTACGGAAGCTCGGATACCGGAATGTGTACTTCCTCCGGGGCGGGCTCATAAATTGGAACGGTCCGGTCCAAAAAGCTGAGGAAAAAACAGATGAATAAAGGGGCGAGTCTGCGGGAGGGGTGTATGATGGAGGAAGACACAGTCCGATATTCCGTGATTTCAGCCAGGCAGACTTTGATATTGGCGTGGTGAATTGATGTGGATTGCGGAGGGATGCATTGCAGTTATTTAAAACTTGTTTGTAACACAAACTATCTGTTGAAACAAAATTTTTATTGAAACATTACCGGTCGTTTGTGAGGTTTTAAAAACACTGGTTAAATTGAAAAACCTCTGGTTTTCACAACCAGAGGTTGATATTCTAAAGAATCGTTTACCACCAGGAGCTGCATTGATATGATGTATCACTGAGGATACATACGCGGAAGCGGCGGTTCGGGTCGCTGGATGCCGGATATGCCCAAGTGGTAATGGTGGATGTTCCGGAAGTGATATAATCATACGTGCCAATGGTTTGTCCGCCATCAAAGCTGACATCAAAGAAAAGGGTATAGCCGGAGCTGGAAGTCGTGAGCCTTCCCCAACCGTATTGTTGTCCGTTGTAGTAGCCCACTCTTAATTCCGCATTACTGATGGTGCGGACGGTTGACCGGTTTGTCGTGCAATCAGGGTCCGAAAGCCAGGCATTGTAAATTTCACACGCTTGCGCTCCAACGTCTGCCGCATGCGATACTGCCGGCACTCCCAAAAATCCCATCACCGCCACCATGGCAATCGCAAACAGTCGTTTCAATCCAAACGCCCCCCGTTAAATTATACTGAAAATTCAAAACACACTTATAATTATAATCTTTATCGGATGGATTGTATAGCATAATTTTCAAATTTCTGAAAAAATGTCAGAAAAGGTTGCATCAGCCGCCATATTAAAAGAGCGTCTGTTTACTTCAACGCGGTTGGATTTTCCGGACTATGTCATTGAACCGGAAGCGAAATCGATTATACTATACAAAGATGTAAAGATTTTCTAAGGAGGATATGATCATGAATCATTTAATGAATGGCAAAAATGTTGTCATCATGGGGGTGGCGAACCAAAGGAGTTTGGCGTGGGGGGTTACCAAATCCCTGCAAGCCGCGGGGGCTAATTTGATCTTTACATACCGGAAAGAACGTTCTTATCAAAAGCTGACTTCTCTGTTGTCAGAGGCTGGAATCGATCCTTTGCTCATTGTTCCGTGTGATGTTTCCGACGATGCCAGTATTGAAAACGCCTTTTCCGAAATCGAACAAAAAGTCGGGACAATCCACGGCTTGGTTCACTCGATTGCATTTGCCGACAAGGATGAGTTGTGCGGGGAGTATGTGGAAACTTCCCGTGACGGGTACCTGATGGCGCAAGAGATGAGCGCATATTCACTGGTTGCAGTGGCCCGGGAAGCAAAAAAACTCATGAGTGACGGTGGGAGCATTGTGACGCAGTCCTATCTCGGGGCCGAGCGGGTCGTGAAAAACTATAACGTGATGGGCGTTGCCAAAGCGGCATTGGAAGCCAGCGTAAGGTACTTGGCAGAGGATCTGGGGAAATATAACATCCGGGTGAATGCCGTATCCTCGGGGCCGATTCGGACCCTTTCCGCAAAAGGCGTTTCCGGTTTCAACGACCTGATGGGGATCATTGCCGAAAAAGCGCCGCTGAGAAGAAACGTGGATCAAAGCGAAGTCGGGGATGCCACATTATTTTTCTTAAGCCCCTTGTCCAGAGGGATTACCGGGGAAGTGCTCCATGTCGATGCGGGTTATCATATCATCGGGGTATAGACGGATTTGCGGTTAAAAGTTAACCGTGTCATATCAATTAAAACCAGTTTGCGATGCAAACAACATAAAAATACCCAAATCTTCTTTTATAAGAAGAAAACCTACAAACCGGCTGTCCATCGGCACAGGAAAGCCGGTTTGTTTTTTGGAAAAAAGATTAACCGGCAGAGACTCCACCGTCAACAATCAGGATGTGTCCCGTTACATAGCCGGCTTCGTCGGAGCACAACCAAGTTACAGACCGGGCGATGTCATCCGGGTCACCGATCCGGTGGAGGGGGATTTTGGATTCATATTGGTTCGGGGTTAATCCTGTTTGCTTGAAGACTTGTTCCAGCATCGGGGTGCGAACCGCTCCCGGGCAGATGGCGTTGATCCGGATGTTGAATTGGGCATATTCCAAGGCGGCAGATTTGGTTAAACTGATCACTCCGCTTTTGCTTGCCGAATAAACGGCTGCGTTCGGATGGCCATCCAATCCGTTGATGGAAGAGATGTTTACAATGGTTCCGGATCCTTGTTTTCGCATCTGGCGGATCTGATGTTTCATGCAAAGCCATATTCCCTTCAGATTGATTGCCATCGTGAGATCAAAATCTTCTTCCGTGATGTCGGCCAGAGGGGCCAATTTCCCGCCGGTACCCGCCGCATTGCATGCGAAGTCCAAGCGCCCGAAGCGCTCCATGGTTGTTTGGATCATTTTCCGGACTTGCTTGTCTTGCGAAACGTCGGCTTGAACCGCAAGGGATTCTCCGCCCAAACCCCTGATCATTTCTTGTGTTTCCTCAATGTTCTCTTTCGTCCGCCCGACGACCACGACTTTTGCGCCTTTTTTGGCAAAAGCCAGGGAGGTGGCTCTTCCGATTCCCGACCCCCCGCCGGTCACCATGGCCACTTTTCCGGCAAATGGTTTCATCTGTTCATCCCCTTCAAAAAACAAATGATTAGCAATTATAAAAATCGTTATACTGATTGTATCTTATCATGTATGAAAAAACCTGAATGAAAAAAGTACCCTTTTTGGATCCAGGAAAAAGAGCAAAACTGCCTTATACTCATTCACAGGAAAGAGAGGGGACGGATTTGGTGAAAGACGATTGGCATAATCCGGAGTTTGCGCTTTACTGGGATCAAACCGCTTTGGCAGGAAACCCGACCCGGAAGGAACAGATGGATCTGCTCATCTTTCTGCTTGAGCAAAAACTTTCTTCAAACGTAACCCTTCTGGATCTGGGGATCGGATCGGGACTGATCGAGGAGAAATTGTTGGCCCGGAGGCCGGATATCACGGTTATCGGAATCGATGCTTCCAAAGCGATGCTGGACTTGGCCCGACCACGGTTGGATCCGGCGCGTTGCCAATTGATTCAGCATGATATCGGCGACACCGATACACTTGCCGTTCCAATGGTGCAAGCAGTCATAAGCGTTCAGATGCTTCACCACCTGCCGCATGACAAACAAAAGGCAGTGTACCGCTTCGTCCATCAACATCTTGAGCCAGGGGGGGCTGTTCCTGATCATGGACCGCATCCAATTGCCGGATGAGGCTTTGCATCCCGACTGCGCGGACATGTGGGACTGGCTTGAACGGAAAACGGATTATCCCGGCGGAACAACGGGGGAGGAATTTCTTGATAAATTCCAACACAAACATGATCACCCGGCAGGATTGGAAGAGCATCTTCGTTGGCTGCGTGAGTCCGGATTTACCGGTGCCTGTTTGCATTTAGCTTTAAATCGCGCTTTGTGGGTCGGGGTGAAAGTGTGAGAAAAGCATTGGAGAAGGAAGGACGAAGTTCCGCATGGATTGCAGAGAAGGCATCACAGACGAACGGTTACCGAATCGTTTTGGCCTCAGACAGAAAATCATGAAGAAACCATCGGATTAATTCCAAACAAGTTTGTAACACAAATAAACCAAAACGCTTTTCTTTCTGCAATCTGCAAGTTAAATGCTCACGCAACCGATCAAGTCGGACAATCCCATGATATATGAAAAGCAATGAACATCTTGCCGGGAAAATTGACTGTTCACCTGACAATGAAACGCCCGCCATGTAAAAAAGGACTTGGGAAAGTATCCAAGTCCTTTAAAAATGCCTAAAGAATAAGTAACACAGAATTCATGATCTACGACCCGCTCTGCCAGAAGGAAAAAGATATGGAAAGAAAGATATGGAAAGAATGAATGTAAATACGAAAGTTATGGCGATTGCAGGTCCTGTAATGGAAGAAGTTGAGCAAATGGAAAAACAATTCAGGCAGGTTACCAGATGAAGAAGAGAGTTAAAAAAAGAAAGGCAAAATAGAAGTTCAATTCACTTTATGAAATAAAAAAACACACCAAAAGAACCCAATAAAGCTTTATTGGGTTCTTTTTGAATAATTAAAAACGCTTTATTTCATTAATCGGGATTACAATGATTTTTCTATATTCTAACTCATAATACAAATAAGCCAACATTTCCTCGGGAAATTCAATATCCTTTTCATATAACATATATTCAATATAGGTTTCAGCCCATTCATGATGATCTTCAAAAAGCTCCCGTACTCTATTTTTAAACAATTCATCCATTTTATCTTTTTCATAACGAGGCTTCGGAACGCCTTGCCAACTAAGAAGGTCATACCCAAAATGCTCGACTAAACCCATATTTATTGACCGATTATAGACATCCTCCAAAAATAACCGATCATGGATGCCGACTTTTCGAATAAAAAATTGTTTTGCCTCTTGTGAGCTATTCGCTTCTACAATAAAGCATTGATTGTGTATTGTGTTTTGATTGACTTGTTCCCCTTCTATAACCAGATAAGAAGCCATCAAATTCACTCCTCAACAGAATTTAAATAAAATCTGAAAATGATAATTACATTTTATACAATGATATAATGAGAGTATATCCGAAATGCCAATAAGAAGCGATAAGAATGCCTAGAGTCAAACCAAGACCAGAATGGATCGAATGGAGTTAAAAGAAGAAGGAAAGCCAGTCAGCAGAAATATCTCTTGCTGACTATCTTGGTGTATCGAAGCGATGATCAGCTTCGATATGGTCCTAATATAATTGATCGGTACTTGGCTGCAACGGATAAAGGTGGAACGTCCAAGCGCCAGGACAAAAACCTTTTTGGGACTTATCTCGAACTTTCCCCTTTGTTTATTTTCAGCCATTTTTCCAGTTCCATATTGGGGAATTCATCTGCGGTATCGATTTGAAGCGGAGAAATTAGTTCTAAAAGATTTCCATCAGGATCTTTAAAATAGACAGCTGCATGTGCGTGAGGAACGTTTGACAATACAAGAGGTTGTCTATCGGGTGAAAATTCAAAGAAAGGACTCACTTCAACCCCAATCGATTGTAACCATTCTTTTGCTTTTTTAATATCCTCTACATAAAAGGCAATATGACGTAAAGAAACATGATAAGGCGTATGAACTTGTTCACATTGCCATAACCCTAACCAACTCTTGCTTTTTTGAATCCAAAAAAAAGCTAAATTTTCACTACGGTGAGCCAATTCAAGACCTAAGTTTTTATAAAACTCAATTGAACGATCTAAATCACTGACAGGCAAATGGGCTTCATATAATCCTTGAATCATTTTGTCCCCTCCCTCAATATCCGGAATAGCCGAAGGTAATAAAAATCAGAAAAATGAGGTTACTCAATCGATAACATATTTACTCACCCAGTCTTATGATATTCTAATACCCCCTTAAATATATTTTATAAACATATTTATTTATTAAATATAAAAAGAAAAGAGAGCAACGCTGTCTTCCATCCCACGATTCAAACACTTCCCGGGGAATGCCACATTCACGATCCACGTGACCATGAAGTCATCCACCAGATCCTCGTTAATGAACGTCCTTAGAAGGAACAAAAAAGCTGCCTTATTCAGGCGGAGGGGGGATTTACATGAGACACCCGAAAATTCAAGGAGGGCCGACAAAACGAGAGAAGGGAGTTGCTGATGCTCACTTCATCTAAGGAGTTGTCCGATTTTGAAAAAATGACTATAAAACTTTATATTATTGCGAATAGTTTATTACGTGAATAATTAAATTATTCAAATGTTATTTGTTATGGGATTTTTGAAGAAAAGATCATCCGTGATGTGTCTTCTTGCAGCAATGGAGATAAATCTGTTTAATAACTCACCTGCTAAAACAGTAAAGAACGTTTTTTGAAGCAATACCCAAAACAGGTTGATCTCTTCTCCTTTCCAAAGTAGCTACATCGTTGATGACTTAAAGGAGATGATAATTTTTGCTAAAACGCGTTTATCGCCCATGGGGCAGAATAGGAATGGTTCTTTTCGTATCTCTGGCACTGGTTGCAGCACTATATTTTGAAAAAGATACAATGGCACATATACTGGGCGTGGTTCTTGCGGTTGCAGCTTGGTTTGCAACAGCCAGCTTTTTTCCTGAAAGAGTTTCTGATTATTCAACTATACAAAAAATCGTTTTCATATTAATAACATTGATCATAATCATTCTTACGCTATTCTTGTTTTAAAGGTAGGAAGGTGTTTTTATGAGTAAATGGGAACTAATTCTTTGCAAGCCAAGTGGGGAAGAAAAGATTAAAAATCCGGATTGGAATCAAATCAAAAGTTACTTGGATCAAGTGGATGGAGATCGACGTCGTGATCATAATTTCCTTGATGCTTTATGTTTAATCAACCCGGATATTGGTGAAATGAACGTCTTCGGGGGAAATGAAATCGATGGTCGTCGCCTTTACGCGATCATTTTTTTCCCAGCAGAATGTGATTATGAAAAACAATATATGTTACTCAATCCTGAGGTTCAAACACGAGGTGAAGAGTATGAACTGATCACAAACGAAGGAGTGGGAATAGATTTCAACAAAGAATGGTTGGTGGAATATGATCCGATGATTCAAGCTTTTAAACATTTCTTTGAAACAGGAAAACTTGACGATACATTGGAATGGGATGAGTGGTAGGATACCAGGATACTTATGGGTTCGTTTCCGCTATATTTGGCGTAATATATGAAGGGGATTTCCCCTTTTAATCGTCCCGATTGCACAGAAGCAATTGAGAAAAAAGACTTTTAGATGATTTCAAAAGCCGAGATTCTGAAATCATCAAACCCTTTCCCATGGCCATTTTTGGCTGTGGGATTCTTTATTTTGTTAAAGCACAGTTCATTTCACAGCTCAAACCACAGTTACACAGACAACATTTTAAATAACTATAAAAAGAAAAATATAAATAATTAAGACTAAAAAACAAAATGTTTTTTAGCCTTGAATCAAGGTTTATTTAACAAGTCTTTTTATAAGAAATCAATCACGGAGAGAAAAGGGTTAAAATAAAAATTCTTCATTCATTCAACTTTTTTGCTGTGAGAATCTTCAAGTCAAAAAATATATAAGTCAAAGTTTCACCAGCGAAGAGGTGGCCGTCACCTGCACGAACGTGACACAGTGGTATTTTCGTTCACTGTGTAAGTGGCGTGCCAGGAGATTGACTAATGAAATAGGAGGTTATCATATGAAAAAGTTAGACGAGATACAAATCCGGTTCATTGAATCCAGTTTGGAATAAAAAAAGACCGTCCGATATGGACGGTCTTTTTTATGCATGCAAAAATACATGCTGATCCAATATTAAAGTTCCCGGGTATTTTTCATAACCACTCGACATTTGAATTTCAAGAGGATTCGGTGGTTATCTGGACTTTCCTCTTTTGAAAAGTAACGTAAGATTTTTTGTTACTTATTAAATCTAGCCAATCCGTTCCATCATTCTGACGGAACCTTGGCATCAATTGAAAGCCAAGCCAACATCGTTTCAATCCTCTTGATGAGATGCAATTATTCTGACGGCATATGTCTCAAACCCTTGACACGCTTGAGGTTCAATATGGTTTTTCGAGCACCTTCGTTTTTTAAAGAGCATTTAAGTTATTTTTTATGTATCAGCTCAAAACGAAGTGGCTTTTTCCTTTTTGACGATTGGGCAAAGGTCAATGTGGAGTTGACCGAGAAAAAGTTTCAATCCTCTAGAAGAGGTGTAGTCACTTTGACCCAACTTCCAGATCAAATGAAGACGTAACATCATGAGTTTCAATCCTTTTGATGAGGTGTAATCATTCTGACCATGAAGAACAAGTTTCTCAAATTTTATACGACGAAGATGGTTTCAATCCTCTTGATGAGGTGTAATCATTCTGACAGTAACAAATCGGATGAAGTGAAATCGAACAACATCGTTTCAATCCTCTTGATGAGGTGTAATCATTCTGACGGCATGTGTCTGAAACCCTTGGCACTCTTGAGGTTCAGCACCGTTTTTCGAGCACCTTCGTTTTTTAAAGAGCATTTAAGTTATTTTTTATGTATCAAGTCAAAACGAAGGTGGCTTTTTCCTTTATTTCATGCGGGTTTCAGCTTTTGCGAGCAACCCCGGGGATTTTTGAGATATCAAGGTGCTCGAAAAATAACTTCTGTTACAAGTTTGAACATCAGGAATATCAATTATTTTCAGTTTAGCAAAAAGATAAAAAGACTGTAAACCTTCAAAGTCTAACATTTTAAAGGCAAGAGATAATCTCTATTAAAACATCTCTCCGGTGATGGAGAGAGCTGTTTTTGTAATCCTCTAGAAGAGGTGTAATCATTGTGACCACAGCAACGGTTGACAGCATTTGCGTGATAGGTGGTGTTTCAATTCTCAAAATGAGATGCAATCAAGTTCTTTGTTGAAAATAATAACCCGTCTAACCTCTGATTTCAAGAGGTTTTTCGGCGGTTGTATTGATTATCAATATTCGACTAAAAATGGATCTTGAGAAACTCGAACACCTGAAAATTCAATATACACAAAAACTGAAGCACATTGCTTGACCTGCAAAAGAGCAGGTTTATTTTTTTAATTAAAAATCAAATGCACATCTCGGCAGGAACAGGTTTCTGGGTTCATTCTATTGTTCATGCTGACGGGGGGATTAATGTTCCTGAGATTAATGAGGATTTCAATCAATTTGAACAAAAAAACCCGGTTCAGAAGGAGCAACCGGTCTCATCCCCTCCGGTTGAAGAAAAGAAAGGATTTGTGGATAAGTTTACCGAATCATTGTCAAAGGTAGGAGATTGGTTAGGGGACAAAATTTCCGGAGCATGGGAATGGACCAAGGAAAAAGCGTCTGCCTTTTGGGATTGGTTTACTGAAATTTGCTCCAAAATAGCAGAAGTCGTAATCGATACACTGTCTGCAGTTTGGGACTGGATCGTCAAATTTAAAGAATACATAGCGTTTGCCGGCGTCCTGATTTTGGGCATTGTCTTGTGCATCTTTGCCACACCGTTAGGGATTGCCGTACTCAGTGGTATGGCTTTGTCATTTGTGATCAGTATGGCCCTGAATGGTTGGGAAATTAACAAAATGACCTTCTTGAAGCAGCCATTGGCGGCCTCTTGGGATTGATCGGAGGAGGCATCACGGCAGGAGCTTCCCGGGGCTTGGCCAGCGGCATCGGGCAAAAACTGATCATGGGTGCCAAAAATTCCAAATTCTTGGGTCCTGTTCTTCGCGGAGGACAGAAACTGGTTT
